# Synthesis and Anti-Inflammatory Evaluation of a Library of Chiral Derivatives of Xanthones Conjugated with Proteinogenic Amino Acids

**DOI:** 10.3390/ijms241210357

**Published:** 2023-06-19

**Authors:** Sara F. Vieira, Joana Araújo, Virgínia M. F. Gonçalves, Carla Fernandes, Madalena Pinto, Helena Ferreira, Nuno M. Neves, Maria Elizabeth Tiritan

**Affiliations:** 1 3B’s Research Group, I3BS—Research Institute on Biomaterials, Biodegradables and Biomimetics, University of Minho, Headquarters of the European Institute of Excellence on Tissue Engineering and Regenerative Medicine, AvePark, Parque de Ciência e Tecnologia, Zona Industrial da Gandra, Barco, 4805-017 Guimarães, Portugal; sara.vieira@i3bs.uminho.pt (S.F.V.); nuno@i3bs.uminho.pt (N.M.N.); 2 ICVS/3B’s–PT Government Associate Laboratory, 4806-909 Braga/Guimarães, Portugal; 3 Laboratório de Química Orgânica e Farmacêutica, Departamento de Ciências Químicas, Faculdade de Farmácia da Universidade do Porto, Rua Jorge de Viterbo Ferreira 228, 4050-313 Porto, Portugal; m.araujo@gmail.com (J.A.); cfernandes@ff.up.pt (C.F.); madalenakijjoa@gmail.com (M.P.); 4 TOXRUN—Toxicology Research Unit, University Institute of Health Sciences, CESPU, CRL, 4585-116 Gandra, Portugal; virginia.goncalves@cespu.pt; 5 UNIPRO—Oral Pathology and Rehabilitation Research Unit, University Institute of Health Sciences (IUCS), CESPU, CRL, 4585-116 Gandra, Portugal; 6 Interdisciplinary Centre of Marine and Environmental Research (CIIMAR), University of Porto, Terminal de Cruzeiros do Porto de Leixões, Avenida General Norton de Matos, S/N, 4450-208 Matosinhos, Portugal

**Keywords:** anti-inflammatory activity, chirality, chiral pool, enantioselectivity, xanthones

## Abstract

In recent decades, the relationship between drug chirality and biological activity has been assuming enormous importance in medicinal chemistry. Particularly, chiral derivatives of xanthones (CDXs) have interesting biological activities, including enantioselective anti-inflammatory activity. Herein, the synthesis of a library of CDXs is described, by coupling a carboxyxanthone (**1**) with both enantiomers of proteinogenic amino esters as chiral building blocks (**2**–**31**), following the chiral pool strategy. The coupling reactions were performed at room temperature with good yields (from 44 to 99.9%) and very high enantiomeric purity, with most of them presenting an enantiomeric ratio close to 100%. To afford the respective amino acid derivatives (**32**–**61**), the ester group of the CDXs was hydrolyzed in mild alkaline conditions. Consequently, in this work, sixty new derivatives of CDXs were synthetized. The cytocompatibility and anti-inflammatory activity in the presence of M1 macrophages were studied for forty-four of the new synthesized CDXs. A significant decrease in the levels of a proinflammatory cytokine targeted in the treatment of several inflammatory diseases, namely interleukin 6 (IL-6), was achieved in the presence of many CDXs. The amino ester of L-tyrosine (X1AELT) was the most effective in reducing IL-6 production (52.2 ± 13.2%) by LPS-stimulated macrophages. Moreover, it was ≈1.2 times better than the D-enantiomer. Indeed, enantioselectivity was observed for the majority of the tested compounds. Thus, their evaluation as promising anti-inflammatory drugs should be considered.

## 1. Introduction

**Figure 1:**
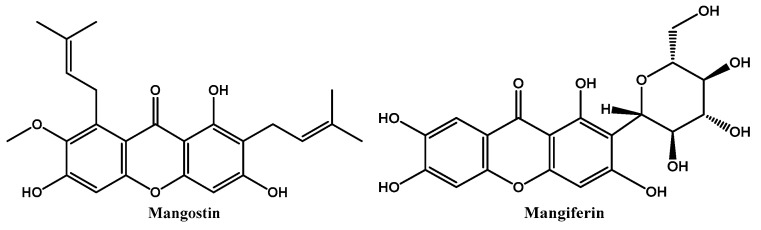
Naturally occurring biologically active xanthone derivatives.

The World Health Organization recognized that natural organisms, such as plants and fungi, are a source of pharmacologically active compounds, which may be used in basic and high levels of health care practice [1]. However, natural products are not easy to commercialize due to their structural complexity and the difficulty to produce them on a large scale. Indeed, most of the time, the purification processes are very laborious and their synthesis is unattainable. Nevertheless, their unique structures inspire the design of new analogues with similar biological actions. Among the natural products with different therapeutic uses, xanthone derivatives, representing a great diversity of compounds, possess a broad spectrum of biological and pharmacological activities [2,3,4,5], such as anticancer [6,7], antimicrobial [8,9], and anti-inflammatory [10,11] activities. For example, disease-preventative and therapeutic properties of mangosteen (*Garcinia mangostana*), also known as the “queen of fruits”, have been ascribed to xanthone derivatives that are present in several parts of the tree, including the pericarp, fruit rind, peel, stem bark, root bark, and leaf [12]. Mangostin (Figure 1), the most abundant xanthone derivative in the *G. mangostana* pericarp [12], has been reported to demonstrate anti-inflammatory activity. Recently, the ethanolic extract of this plant part was revealed to be a promising formulation to treat MRSA-induced superficial skin infection due to its antibacterial, anti-inflammatory, and wound healing effects [13]. Mangiferin (Figure 1) is another example of naturally occurring biologically active xanthone derivatives with diverse pharmacological properties, including anti-inflammatory activity [4].

Xanthone derivatives are very attractive in drug development since they are considered privileged structures. Indeed, they present endless different patterns of substitution that allow for generating a library of derivatives able to interact with several biological targets [2,14]. Moreover, as biological systems have high selectivity towards chiral compounds, the research concerning chiral derivatives of xanthones (CDXs) and their pharmacological/biological properties constitutes a very interesting and promising field. Biological systems are structurally chiral, as the building blocks amino acids, sugars, and nucleic acids of the proteins, glycoproteins, and deoxyribonucleic acid (DNA) are chiral. Therefore, enzymes, receptors, or other binding molecules are intrinsically chiral and able to discriminate enantiomers by exhibiting different biological responses (enantioselectivity) [15,16]. In fact, the metabolic and regulatory processes mediated by biological systems are sensitive to stereochemistry as differences in pharmacodynamics and pharmacokinetics can frequently be observed for each enantiomer [2,16,17]. Even if the enantiomers possess similar pharmacodynamics, but one is more potent than the other, the administration of the pure and more effective enantiomer may have advantages, such as the requirement of lower therapeutic doses, higher safety margins, fewer side effects and drug interactions, as well as lower variability among individuals [16,17]. Beyond the well-known enantioselectivity in the pharmacodynamics, pharmacokinetics, and toxicological effects, the importance of the chirality feature led to regulatory authorities recommending that chiral drugs should be marked as pure enantiomers [18,19,20]. Additionally, the introduction of chirality in the development of new molecular entities increases the structural diversity and complexity, which can lead to more selective drugs and improve the affinity between the drug and the biological target [16,21]. Thus, the preparation of new CDXs is important not only because they will lead to the improvement in pharmacological selectivity of xanthone derivatives but also as a good strategy to provide a large library of compounds with a broad spectrum of biological activities for enantioselective studies and structure–activity relationships (SARs). Consequently, the research of new CDXs and their biological/pharmacological properties constitutes a promising field that has already shown relevant results [2,3]. Examples of synthesized CDXs with enantioselectivity that have been explored in our research group include CDXs as cell growth inhibitors [6,22,23], sciatic nerve blockers [24], antimicrobial resistance mechanism inhibitors [25], and cyclooxygenase inhibitors [10]. A great contribution in this field was also made by Professor Marona’s research team, describing a variety of CDXs with diverse activities, such as anticonvulsant [26], local anesthetic [26], cardiovascular [27], antifungal and antibacterial [28], antiarrhythmic [29], antiplatelet aggregation [30], antiadrenergic receptors [31], and more recently antioxidant [32,33] and anticancer potential [34,35]. Although the strategy to conjugate amino acids with xanthone derivatives has been explored in the screening of different biological activities, the enantioselectivity was frequently neglected [2,3,36]. This work describes the synthesis and structural elucidation of a new library of proteinogenic amino ester and amino acid derivatives of xanthones and their anti-inflammatory activity evaluation. Both enantiomers were evaluated in order to assess the enantioselectivity and, consequently, to determine the more effective enantiomer.

## 2. Results and Discussion

### 2.1. Synthesis and Structural Elucidation of a New Library of CDXs

The carboxy xanthone 2-((9-oxo-9*H*-xanthen-3-yl)oxy)acetic acid—XCAR-1 (**1**)—was synthesized according to the procedure previously established by our research group [6]. Then, it was used as the chemical substrate for the synthesis of the new library of CDXs. The sixty new CDXs were synthesized according to the chiral pool strategy, using the coupling reagent (1-cyano-2-ethoxy-2-oxoethylidenaminooxy)dimethylamino-morpholino-carbenium hexafluorophosphate (COMU) and/or *O*-(benzotriazol-1-yl)-*N*,*N*,*N*’,*N*’-tetramethyluronium tetrafluoroborate (TBTU). The commercial enantiomerically pure amino esters were bonded to XCAR-1 (**1**) to afford the library of CDXs, as shown in Table 1. The amino ester derivatives were then hydrolyzed to achieve the derivatives of amino acids, as shown in Table 2. The structures of all CDXs of amino esters and amino acids were elucidated by infrared (IR) spectroscopy and ^1^H and ^13^C nuclear magnetic resonance (NMR), while the enantiomeric purity was evaluated by chiral liquid chromatography (cLC). The data from IR are presented in the Materials and Methods section and cLC and NMR analyses are presented in Tables S1 and S2 and Figure S1 (Supplementary Materials—SM).

#### 2.1.1. Synthesis of the CDXs of Amino Esters

The synthesis procedure of all CDXs of amino esters (compounds **2**–**31**;**Table 1**) was firstly performed by the activation of the carboxylic acid of XCAR (**1**), using the coupling agent COMU, or TBTU in some cases. The reactions were conducted at room temperature in the presence of triethylamine (TEA) in catalytic amounts and using dry tetrahydrofuran (THF) as the solvent of the reaction. After reaction with the amino esters, it was possible to conclude that both COMU and TBTU were efficient as coupling reagents with low or non-existent tendency towards racemization. Additionally, as COMU and TBTU are water-soluble, they were eliminated through a simple extraction [37,38]. The reaction times of CDXs of amino esters with COMU as coupling reagent were in the range of 30 min to 30 h, affording high yields (44 to 98%). Only for three CDXs (compounds **20**, **21**, **25,** Table 1) TBTU reveled better results as coupling reagent than COMU, with reaction times from 27 to 30 h and yields from 50 to 67%. The purification procedure of all synthesized CDXs involved only a chemical liquid–liquid extraction and crystallization to avoid racemization and to reduce the production of waste generated by preparative chromatography.

The reaction time changed according to the structure of the amino ester employed but among stereoisomers they were quite similar. Interestingly, even though the esters of leucine and isoleucine have similar structures, the reaction times were quite different. The ester of leucine enantiomers (**6** and **7**) reacted from 30 min to 1 h, while the coupling reaction with isoleucine derivative (**5**) was only accomplished after 24 h. This is because of the space occupied by the ramification of the isoleucine side chain that increases the steric hindrance with the coupling reagent. All CDXs of amino esters were firstly prepared using the coupling reagent COMU, however, as the purification of CDXs of serine (**20** and **21**) and glutamic acid (**25**) enantiomers was difficult, an additional reaction with TBTU as coupling reagent was required. In these first attempts, the preparation of the CDXs with the amino esters of serine using COMU was accomplished in 2 h, yielding 42% of the enantiomer X1AELSer (**20**) and 64% of the enantiomer X1AEDSer (**21**). Although COMU provided better yields in a shorter time, TBTU showed a significant decrease in side products, which simplified the purification process. In the case of the synthesis of X1AEDGlutAcid (**25**) performed with COMU as coupling reagent, the impurities also complicated the purification of the product (a yellow oil). Conversely, the reaction with TBTU allowed for obtaining a solid yellow product with fewer impurities, which were eliminated by crystallization.

The enantiomeric purity of all CDXs was evaluated by cLC using a commercial chiral column. The enantioseparation of the synthesized CDXs was optimized in a cellulose carbamate derivative chiral stationary phase (CSP) in normal elution mode. The optimized conditions, chromatographic parameters, and peak purity are described in Tables S1 and S2 (SM). All CDXs of amino esters revealed high enantiomeric purities with enantiomeric ratio (e.r.) near 100% (Table 1). The lowest e.r. value was found for CDX **18** (95.9%), demonstrating that synthesis and purification procedures allowed a low degree of racemization for all synthesized CDXs. ^1^H and ^13^C-NMR of all CDXs are given in Figure S1 (SM)

#### 2.1.2. Synthesis of CDXs of Amino Acids

All CDXs of amino esters (**2**–**31**) were hydrolyzed under mild alkaline conditions to afford the amino acids analogues (Table 2).

All CDXs of amino acids (**32**–**61**) were obtained by the hydrolysis of the respective amino ester derivative (**2**–**31**). The mild alkaline conditions avoided racemization in the hydrolyses of all CDXs of amino esters, except for the phenylalanine derivatives. Indeed, X1AALPG (**42**) and X1AADPG (**43**) were partially racemized (e.r. = 60.3 and 55.5, respectively).

The hydrolysis of the *tert*-butyl amino ester of the CDXs from alanine enantiomers (**32** and **33**) required the addition of 1 mL of a more concentrated base solution (NaOH 1 M) than the other hydrolysis reactions. According to the results shown in Table 2, the hydrolysis reactions occurred from 5 min (0.0833 h) to 24 h. The time of the reactions changed according to the structure of the amino ester. Additionally, in some cases, the enantiomers presented different times to complete the hydrolysis. For example, for the CDX with the L-enantiomer of the phenylalanine amino acid (X1AALPA, **44**) the hydrolysis was completed in only 1 h, while for the analogue D-enantiomer (**45**) the reaction took 4 h. Additionally, the L-valine derivative (**40**) production was accomplished in 24 h, while for the D-valine (**41**) the reaction was finished in 5 h. Moreover, L-isoleucine amino ester (**35**) needed twice the time of amino ester of leucine enantiomers to hydrolyze (**36** and **37**). The enantiomeric purity of the amino acid derivatives was also evaluated by cLC. The optimized conditions, chromatographic parameters, and peak purity are described inTable S2 (SM). The enantiomeric purity of CDXs of amino acids was also high, except for that of phenylglycine derivative (**42** and **43**) enantiomers (Table 2).

In order to evaluate the ability of all synthesized CDXs to behave as potentially useful drugs, the physicochemical descriptors, as well as absorption, distribution, metabolism, and excretion (ADME) parameters related to pharmacokinetic properties, drug-like nature, and medicinal chemistry friendliness, were predicted using the SwissADME program [39]. Some of the most relevant data are summarized in Table S3 (SM). Since in silico results were similar for both enantiomers, data for only one of the enantiomers were included. The topological polar surface area (TPSA) and logarithm of *n*-octanol/water partition coefficient (Log *Po*/*w*) values for all CDXs ranged from 86.05 to 144.64 and from 1.41 to 3.48, respectively. High gastrointestinal (GI) absorption is expected with 0 violation according to Lipinski’s rule of five. The predicted bioavailability score for all CDXs was about 0.55. Regarding P-glycoprotein (P-gp) substrates, among all CDXs, the derivatives of the amino ester of tryptophan are the only substrate of this drug efflux pump. All CDXs presented good drug likeness, with only the exception of the amino acid derivatives of cysteine, which do not comply with the parameters of Veber’s rule (TPSA value higher than 140 Å²).

### 2.2. Cytotoxicity and Anti-Inflammatory Activity

**Figure 2:**
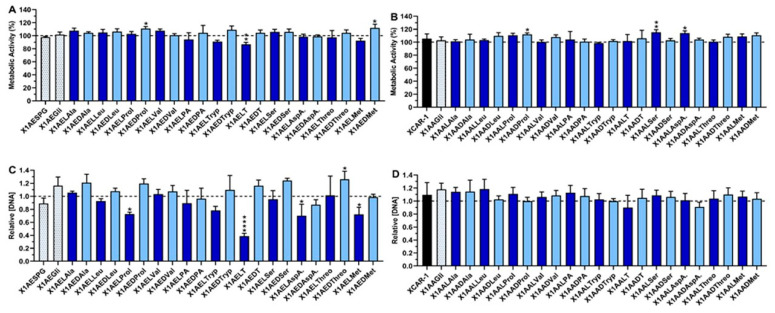
Metabolic activity (**A**,**B**) and relative DNA concentration (**C**,**D**) of LPS-activated macrophages cultured in the presence of CDXs of amino esters (**A**,**C**) and CDXs of amino acids (**B**,**D**) at 20 μM for 22 h of culture. Statistically significant differences are * (*p* < 0.0435), ** (*p* < 0.084), **** (*p* < 0.0001) in comparison to the non-treated LPS-stimulated macrophages for each tested compound.

The cytocompatibility and anti-inflammatory activity of XCAR-1 and twenty-three CDXs of amino acids and twenty-four CDXs of amino esters were evaluated in the presence of lipopolysaccharide (LPS)-stimulated macrophages. Figure 2 and Figures S2–S31 (SM) present the metabolic activity and DNA concentration obtained for LPS-stimulated macrophages in the absence or presence of the tested CDXs and clinically used anti-inflammatory drugs (indomethacin and dexamethasone). As can be observed in Figure 2A,B, with more detail in Figures S2–S15 (SM), the metabolic activity of macrophages was not negatively affected in the presence of the studied compounds, even at the highest tested concentration (20 μM). An exception was observed for the enantiomer X1AELT (**18**), the CDX of the amino ester of L-tyrosine. X1AELT (**18**) significantly decreased the metabolic activity of LPS-stimulated macrophages in comparison with the control (non-treated LPS-stimulated macrophages) for the highest tested concentration. Conversely, this was not observed for the enantiomer X1AEDT (Figure 2). The results in Figure S16 (SM) also demonstrated that indomethacin and dexamethasone did not affect the metabolic activity of the M1 macrophages at the tested concentration.

The DNA content of M1 macrophages was preserved in the presence of most of the compounds at the highest tested concentration (20 μM, Figure 2C,D). However, the CDX of amino ester of the L-enantiomers proline, tyrosine, aspartic acid, and methionine (X1AELProl (**8**), X1AELT (**18**), X1AELAspA. (**22**), and X1AELMet (**30**), respectively) significantly diminished the DNA content (Figure 2C), while this was not observed for the respective CDX with the D-enantiomer. Although, for concentrations up to 10 μM, the DNA content in the presence of these compounds was not significantly affected (Figures S22, S26, S28, and S30). The same behavior was observed for the anti-inflammatory drugs used as control (Figure S31). In conclusion, most of the CDXs are cytocompatible at all tested concentrations, but some CDXs of amino ester are more toxic than their analogue derivatives of the amino acids. Additionally, among the amino ester derivatives, the CDXs with L-enantiomers are more toxic than the derivatives with D-enantiomers.

**Figure 3:**
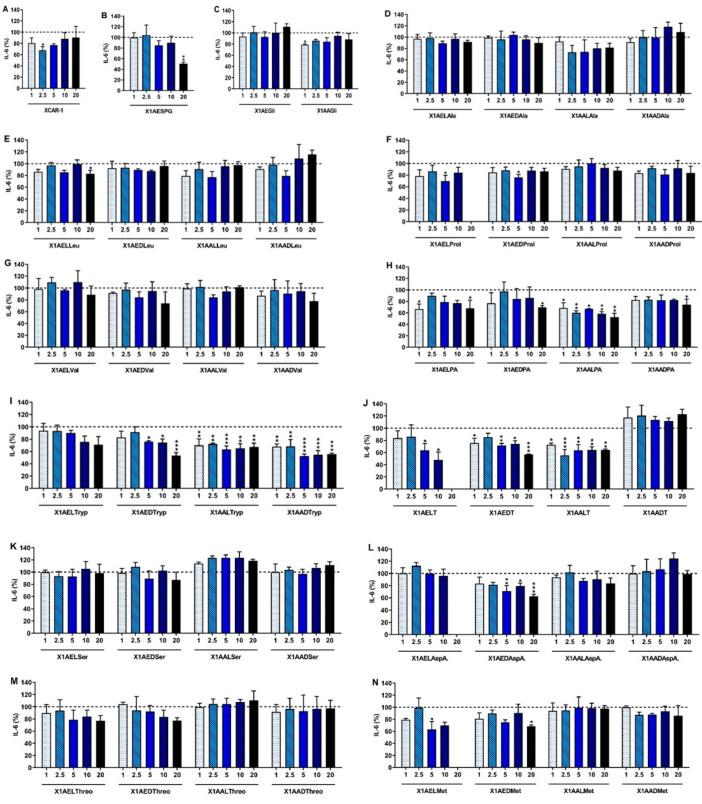
IL-6 (%) production by LPS-activated macrophages cultured in the presence of the tested compounds (**A**–**N**) at different concentrations (1, 2.5, 5, 10, and 20 μM) for 22 h of culture. Statistically significant differences are * (*p* < 0.0469), ** (*p* < 0.0090), *** (*p* < 0.0007), and **** (*p* < 0.0001) in comparison to the non-treated LPS-stimulated macrophages for each tested compound.

The anti-inflammatory activity of the tested compounds was evaluated based on their capacity to decrease the production of the proinflammatory cytokine IL-6 by LPS-stimulated macrophages (Figure 3). As can be seen in Figure S32 (SM), non-stimulated macrophages did not produce measurable amounts of IL-6. An inflammatory scenario, presenting high IL-6 levels, was successfully obtained by the addition of LPS to the macrophages. Dexamethasone (10 μM), a corticosteroid, was the most efficient control in reducing the IL-6 production (Figure S32), as expected. However, in this in vitro inflammatory model, indomethacin, being a non-steroidal anti-inflammatory drug (NSAID), did not show the ability to reduce the IL-6 concentration in the culture media.

In Figure 3, the compounds at cytotoxic concentrations were excluded in order to avoid false positives in the anti-inflammatory activity results. XCAR-1 (2.5 μM, Figure 3A), X1AESPG (20 μM, Figure 3B), X1AAGly (1 μM, Figure 3C), X1AELLeu (20 μM, Figure 3E), X1AELProl (5 μM, Figure 3F), X1AEDProl (5 μM, Figure 3F), X1AELPA (1 and 20 μM, Figure 3H), X1AEDPA (20 μM, Figure 3H), X1AALPA (1–20 μM, Figure 3H), X1AADPA (20 μM, Figure 3H), X1AEDTryp (5–20 μM, Figure 3I), X1AALTryp (1–20 μM, Figure 3D), X1AADTryp (1–20 μM, Figure 3D), X1AELT (5 and 10 μM, Figure 3J), X1AEDT (1, 5–20 μM, Figure 3J), X1AALT (1–20 μM, Figure 3J), X1AEDAspA (5–20 μM, Figure 3L), X1AELMet (5 μM, Figure 3N), and X1AEDMet (20 μM, Figure 3N) demonstrated significant ability to reduce the IL-6 production by LPS-stimulated macrophages.

As can be observed, CDXs of amino esters and amino acids presented differences in effectiveness. For instance, the CDX of amino acid of glycine (X1AAGli) demonstrated anti-inflammatory activity at 1 μM, but this was not observed for its amino ester (X1AEGli) (Figure 3C). The CDX of amino ester of proline had a strong anti-inflammatory activity, which was not observed for its corresponding amino acid (Figure 3F). Besides the different activities between CDXs of amino acids and amino esters, enantioselectivity was also observed. For example, only the L-enantiomer of the CDX of amino ester of leucine (X1AELLeu) led to a significant reduction of the IL-6 production at 20 μM (Figure 3E). Both L- and D-enantiomers (X1AELProl and X1AEDProl) displayed bioactivity at 5 μM, but the D-enantiomer was more efficient. In general, all CDXs with phenylalanine (Figure 3H) and tryptophan (Figure 3I) were able to reduce the IL-6 production. The CDX of amino acid of phenylalanine (X1AALPA and X1AADPA) and tryptophan (X1AALTryp and X1AADTryp) were more efficient than their amino esters (X1AELPA and X1AEDPA versus X1AELTryp and X1AEDTryp, respectively). In the case of CDX of phenylalanine, for both amino ester and amino acid, the derivatives with the L-enantiomer were the most potent at 1 μM (Figure 3H). In addition, this compound at 2.5 and 20 μM displayed similar bioactivities, highlighting its efficacy at low concentrations. Conversely, for CDXs of tryptophan, the D-enantiomers of both amino ester and amino acid were more efficient (Figure 3I). X1AADTryp efficiently reduced IL-6 production at all tested concentrations, but 5 μM of this compound induced stronger IL-6 reduction than 20 μM. The modification of XCAR-1 with tyrosine also improved the anti-inflammatory activity for both D- and L-amino esters and L-amino acid derivatives (Figure 3J). However, the derivatives with the L-enantiomers displayed greater bioactivity (X1AELT and X1AALT) than their enantiomers. Indeed, X1AALT led to a similar reduction of IL-6 with lower concentrations than the most effective D-enantiomer-(2.5 μM for X1AALT versus 20 μM for X1AEDT). The CDX of the D-amino ester of aspartic acid exhibited anti-inflammatory activity for concentrations higher than 5 μM, while its L-enantiomer did not inhibit the production of the proinflammatory cytokine IL-6 at any tested concentration (Figure 3L). The molecular modifications of XCAR-1 with alanine (Figure 3D), valine (Figure 3G), serine (Figure 3K), and threonine (Figure 3M) did not induce any anti-inflammatory effect. These results highlight that the molecular modification of XCAR-1 with a specific amino acid (mainly with phenylalanine, tryptophane, tyrosine, and aspartic acid) impacts the anti-inflammatory activity at different levels. The most efficient compound to decrease the studied cytokine was X1AELT (10 μM, 52.2 ± 13.2%), followed by X1AESPG (20 μM, 49.6 ± 3.2%), X1AALPA (20 μM, 47.6 ± 6.8%), X1AADTryp (5 μM, 47.5 ± 3.2%), X1AEDTryp (20 μM, 46.6 ± 4.6%), X1AALT (2.5 μM, 44.9 ± 9.8%), X1AEDT (20 μM, 43.0 ± 0.5%), X1AEDAspA (20 μM, 37.8 ± 3.4%), X1AALTryp (5 μM, 36.7 ± 5.5%), X1AELMet (5 μM, 36.7 ± 12.8%), X1AELPA (1 μM, 33.6 ± 8.9%), X1AEDPA (20 μM, 32.0 ± 14.0%), XCAR-1 (2.5 μM, 32.1 ± 6.2%), X1AEDMet (20 μM, 31.7 ± 2.6%), and X1AELProl (5 μM, 30.3 ± 9.9%). A low anti-inflammatory activity was observed for X1AADPA (20 μM, 25.9 ± 9.9%), X1AEDProl (5 μM, 23.9 ± 4.9%), X1AAGly (1 μM, 21.3 ± 1.8%), and X1AELLeu (20 μM, 17.0 ± 5.4%). Nonetheless, these compounds showed better anti-inflammatory activity than the clinical NSAID indomethacin at 10 μM.

## 3. Materials and Methods

Ethanol for bioassays was obtained from Fisher Scientific, Portugal. Roswell Park Memorial Institute (RPMI) 1640 medium, fetal bovine serum (FBS), antibiotic/antimycotic solution, Dulbecco’s phosphate-buffered saline (DPBS), and Quant-iT PicoGreen dsDNA Kit were purchased from Thermo Fisher Scientific, Lisbon, Portugal. DMSO was obtained from VWR, Radnor, PA, USA. alamarBlue^®^ was purchased from Bio-Rad (Hercules, CA, USA). Human IL-6 DuoSet enzyme-linked immunosorbent assay (ELISA) kits and DuoSet ELISA Ancillary Reagent Kit 2 were purchased from R&D Systems, Minneapolis, MN, USA. The remaining commercially available reagents and solvents used in this work were purchased from Sigma Aldrich Co., Lisboa, Portugal, and were used without purification. Ultra-pure water was obtained from a Milli-Q^®^ Direct Water Purification System (Milli-Q Direct 16, Millipore, Molsheim, France).

All the reactions were monitored by thin-layer chromatography (TLC) (Merck silica gel, 60 F_254_ plates, Lisboa, Portugal), with appropriate mobile phases, and UV detection at 245 and 365 nm. Melting points were obtained in a Köfler microscope. IR spectra were obtained in KBr disc in a Nicolet iS10 FTIR spectrometer from Thermo Scientific (Waltham, MA, USA) with a Smart OMNI-Transmisson accessory (Software 188 OMNIC 8.3). NMR spectra were measured on a Bruker Advanced 300 and 500 MHz (300.13 MHz for ^1^H and 75.47 MHz for the ^13^C), Bruker, Germany. All samples were dissolved in deuterated dimethyl sulfoxide (dDMSO) and the chemical shifts are expressed in δ (ppm) values relative to tetramethylsilane (TMS), used as an internal reference. The ^13^C NMR assignments were made by 2D (HSQC and HMBC) NMR experiments (long-range ^13^C–^1^H coupling constants were optimized to 7 Hz). Liquid chromatography analyses were performed on a LaChrom Merck Hitachi HPLC, Lisboa, Portugal, equipped with an L-7100 pump, an L-7200 auto-injector, an L-7455 diode array detector, and a D-7000 interface. The chiral column used was Lux^®^ 3 µm Cellulose-2 (150 × 4.6 mm) from Phenomenex and data analysis was performed using HPLC System Manager HSMD-7000 software, version 3.0. High-resolution mass spectroscopic analysis was performed using an LTQ OrbitrapTM XL hybrid mass spectrometer (Thermo Fisher Scientific, Bremen, Germany) controlled by LTQ Tune Plus 2.5.5 and Xcalibur 2.1.0. The capillary voltage of the electrospray ionization source (ESI) was set to 4.0 kV. The capillary temperature was 350 ºC. The sheath gas was 6 0 (arbitrary unit as provided by the software settings). The capillary voltage was 49 V and the tube lens voltage 90 V. The capillary voltage of the ESI was set to 3.0 kV. The capillary voltage was −35 V and the tube lens voltage −110 V. MS data handling software (Xcalibur QualBrowser software, Thermo Fisher Scientific) was used to search for predicted peptides by their m/z value.

### 3.1. General Procedure for the Synthesis of Chiral Derivatives of Xanthones of Amino Esters

The 2-((9-oxo-9*H*-xanthen-3-yl)oxy)acetic acid—XCAR (**1**) (100 mg, 0.37 mmol)—was dissolved in dry THF (20 mL) and then TEA (100 µL, 0.72 mmol) was added. Then, the coupling reagent (1.2 eq. mmol) was added, and the solution was stirred for about 30 min. Afterward, the appropriate chiral reagent amino ester (1.7 eq.) was added, and the mixture was stirred at room temperature for 30 min up to 30 h. The reaction was followed by TLC using chloroform:methanol:acetic acid (9:1:0.1, *v*/*v*/*v*) as the mobile phase. After finishing the reaction, the solvent was evaporated, and the crude product was dissolved in dichloromethane. The solution was then washed with a 5% HCl solution (2 × 13 mL), 5% NaHCO_3_ solution (2 × 15 mL), and water (3 × 25 mL). The organic layer was dried with anhydrous sodium sulfate, filtered, and the solvent was evaporated. The products were then crystallized to afford the CDXs of amino esters (**2**–**31**). The HRMS (ESI) data of the analyzed CDXs are in Table S4 (SM).

#### 3.1.1. *tert*-Butyl (2-((9-Oxo-9H-xanthen-3-yl)oxy)acetyl)-L-alaninate (**2**)

The coupling reaction finished in 1 h. After extraction, the product was recrystallized with ethanol containing a few drops of THF and water to afford compound **2** as a white crystal solid (120.25 mg, 81.4%). m.p.: 130–132 °C; IR (KBr): ṽ 3401, 1745, 1649, 1621, 1523, 1466, 1449, 1257, 871, 833, 757, 670 cm^−1^. ^1^H NMR (500.13 MHz, DMSO-*d*_6_): *δ =* 8.54 (1H, *d*, *J* = 7.3 Hz, N-H), 8.17 (1H, *dd*, *J* = 7.9 and 1.1 Hz, H-8), 8.12 (1H, *d*, *J* = 8.5 Hz, H-1), 7.86 (1H, *m*, H-6), 7.63 (1H, *d*, *J* = 8.4 Hz, H-5), 7.48 (1H, *m*, H-7), 7.12 (1H, *dd*, *J* = 8.5 and 2.3 Hz, H-2), 7.11 (1H, *d*, *J* = 2.3 Hz, H-4), 4.81 (1H, *d*, *J* = 6.4 Hz, -OCH_2_), 4.77 (1H, *d*, *J* = 6.4 Hz, -OCH_2_), 4.22 (1H, *m*, H-2′); 1.39 (9H, *s*, -OC-(CH_3_)_3_), 1.31 (3H, *d*, *J* = 7.3 Hz, H-3′) ppm. ^13^C NMR (75.5 MHz, DMSO-*d_6_*): *δ =* 174.9 (C-9), 171.5 (C-1′), 166.7 (C=O amide), 163.4 (C-3), 157.3 (C-4a), 155.6 (C-10a), 135.2 (C-6), 127.6 (C-1), 125.9 (C-8), 124.4 (C-7), 121.2 (C-8a), 117.9 (C-5), 115.4 (C-9a), 114.1 (C-2), 101.6 (C-4), 80.6 (-OC-(CH_3_)_3_), 66.9 (-OCH_2_), 48.2 (C-2′), 27.6 (-OC-(CH_3_)_3_), 17.0 (C-3′) ppm.

#### 3.1.2. *tert*-Butyl (2-((9-Oxo-9H-xanthen-3-yl)oxy)acetyl)-D-alaninate (**3**)

The coupling reaction finished in 30 min. After extraction, the product was recrystallized with ethanol containing a few drops of THF and water to afford compound **3** as a white crystal solid (130.01 mg, 87.7%); m.p.: 127–129 °C; IR (KBr): ṽ 3401, 1745, 1649, 1621, 1522, 1507, 1466, 1450, 1257, 871, 833, 756, 669 cm^−1^. ^1^H NMR (500.13 MHz, DMSO-*d_6_*): *δ* = 8.54 (1H, *d*, *J* = 7.3 Hz, N-H), 8.17 (1H, *dd*, *J* = 7.9 and 1.1 Hz, H-8), 8.12 (1H, *d*, *J* = 8.5 Hz, H-1), 7.86 (1H, *m*, H-6), 7.63 (1H, *d*, *J* = 8.4 Hz, H-5), 7.48 (1H, *m*, H-7), 7.12 (1H, *dd*, *J* = 8.5 and 2.3 Hz, H-2), 7.11 (1H, *d*, *J* = 2.3 Hz, H-4), 4.76 (1H, *d*, *J* = 2.6 Hz, -OCH_2_), 4.74 (1H, *d*, *J* = 6.4 Hz, -OCH_2_), 4.23 (1H, *m*, H-2′); 1.39 (9H, *s*, -OC-(CH_3_)_3_), 1,31 (3H, *d*, *J* = 7.3 Hz, H-3′) ppm. ^13^C NMR (75.5 MHz, DMSO-*d_6_*): *δ =* 174.9 (C-9), 171.5 (C-1′), 166.7 (C=O amide), 163.4 (C-3), 157.3 (C-4a), 155.6 (C-10a), 135.2 (C-6), 127.6 (C-1), 125.9 (C-8), 124.4 (C-7), 121.2 (C-8a), 117.9 (C-5), 115.4 (C-9a), 114.1 (C-2), 101.6 (C-4), 80.6 (-OC-(CH_3_)_3_), 66.9 (-OCH_2_), 48.2 (C-2′), 27.6 (-OC-(CH_3_)_3_), 17.0 (C-3′) ppm.

#### 3.1.3. Methyl (2-((9-Oxo-9H-xanthen-3-yl)oxy)acetyl)glycinate (**4**)

The coupling reaction finished in 23 h. After extraction, the product was recrystallized with dichloromethane and *n*-hexane to afford compound **4** as a white solid (72.3 mg, 71.6%). m.p.: 155–157 °C; IR (KBr): ṽ 3367, 1741, 1665, 1619, 1538, 1508, 1464, 1434, 1256, 851, 837, 757, 704, 689, 776 cm^−1^. ^1^H NMR (400.14 MHz, DMSO-*d*_6_): *δ =* 8.68 (1H, *t*, *J* = 5.9 Hz, N-H), 8.18 (1H, *dd*, *J* = 7.9 and 1.6 Hz, H-8), 8.14 (1H, *d*, *J* = 8.6 Hz, H-8), 7.86 (1H, *ddd*, *J* = 8.5, 7.1 and 1.5 Hz, H-6), 7.65 (1H, *dd*, *J* = 8.4 and *J*=1.5 Hz, H-5), 7.45 (1H, *m*, H-7), 7.13 (1H, *dd*, *J* = 8.7 and 2.4 Hz, H-2), 7.12 (1H, *d*, *J* = 2.4 Hz, H-4); 4.79 (2H, *s*, -OCH_2_), 3.94 (2H, *d*, *J* = 5.9 Hz, H-2′) and 3.65 (3H, *s*, -OCH_3_) ppm. ^13^C NMR (100.6 MHz, DMSO-*d_6_*): *δ* = 175.4 (C-9), 170.5 (C-1′), 168.0 (C=O amide), 163.7 (C-3), 157.8 (C-4a), 156.1 (C-10a), 135.6 (C-6), 128.1 (C-1), 126.4 (C-8), 124.9 (C-7), 121.7 (C-8a), 118.4 (C-5), 115.9 (C-9a), 114.6 (C-2), 102.2 (C-4), 67.6 (-OCH_2_), 52.3 (-OCH_3_), 40.9 (C-2′) ppm.

#### 3.1.4. Methyl (2-((9-Oxo-9H-xanthen-3-yl)oxy)acetyl)-L-isoleucinate (**5**)

The coupling reaction finished in 24 h. After extraction, the product afforded compound **5** as a white solid (150.80 mg, 98.9%). IR (KBr): ṽ 3354, 1748, 1668, 1648, 1621, 1525, 1466, 1448, 1433, 1256, 870, 834, 760, 672 cm^−1^. ^1^H NMR (400.14 MHz, DMSO-*d*_6_): *δ =* 8.53 (1H, *d*, *J* = 8.2 Hz, N-H), 8.17 (1H, *dd*, *J* = 7.9 and 1.6 Hz, H-8), 8.11 (1H, *d*, *J* = 8.5, H-1), 7.84 (1H, *ddd*, *J* = 8.5, 7.1 and 1.6 Hz, H-6), 7.62 (1H, *dd*, *J* = 8.4 Hz and *J* = 1.1, H-5), 7.47 (1H, *m*, H-7), 7.09 (1H, *dd*, *J* = 8.5 and 2.4 Hz, H-2), 7.08 (1H, *d*, *J* = 2.4 Hz, H-4), 4.85 (1H, *d*, *J* = 14.7 Hz, -OCH_2_), 4.80 (1H, *d*, *J* = 14.7 Hz, -OCH_2_), 4.29 (2H, *dd*, *J* = 8.1 and 6.7 Hz, H-2′), 3.65 (3H, *s*, OCH_3_), 1.86 (1H, *m*, H-3′), 1.43 (1H, *m*, H-4′), 1.22 (1H, *m*, H-4′), 0.84 (3H, *t*, *J* = 7.3 Hz, H-5′), 0.86 (3H, *d*, *J* = 6.8 Hz, H-6′) ppm. ^13^C NMR (100.6 MHz, DMSO-*d*_6_): *δ =* 174.9 (C-9), 171.7 (C-1′), 167.1 (C=O amide), 163.5 (C-3), 157.3 (C-4a), 155.6 (C-10a), 135.1 (C-6), 127.6 (C-1), 125.9 (C-8), 124.4 (C-7), 121.2 (C-8a), 117.9 (C-5), 115.3 (C-9a), 113.9 (C-2), 101.4 (C-4), 66.8 (-OCH_2_), 56.2 (C-2′), 36.2 (C-3′), 24.8 (C-4′), 15.5 (C-6′), 11.2 (C-5′) ppm.

#### 3.1.5. Methyl (2-((9-Oxo-9H-xanthen-3-yl)oxy)acetyl)-L-leucinate (**6**)

The coupling reaction finished in 1 h. After extraction, the product was recrystallized with dichloromethane and *n*-hexane to afford compound **6** as a white crystal solid (113.34 mg, 76.7%); m.p.: 111–112 °C; IR (KBr): ṽ 3387, 1746, 1672, 1622, 1528, 1465, 1438, 1258, 826, 792, 754, 665 cm^−1^. ^1^H NMR (400.14 MHz, DMSO-*d*_6_): *δ =* 8.62 (1H, *d*, *J* = 7.9 Hz, N-H), 8.18 (1H, *dd*, *J* = 8.0 and 1.5 Hz, H-8), 8.13 (1H, *d*, *J* = 8.5, H-1), 7.85 (1H, *ddd*, *J* = 8.6, 7.0 and 1.6 Hz, H-6), 7.63 (1H, *dd*, *J* = 8.5 and 1.0 Hz, H-5), 7.48 (1H, *ddd*, *J* = 8.0, 7.1 and 0.9 Hz, H-7); 7.11 (1H, *dd*, *J* = 8.5 and 2.4 Hz, H-2), 7.10 (1H, *d*, *J* = 2.4 Hz, H-4), 4.82 (1H, *d*, *J* = 14.9 Hz, -OCH_2_), 4.77 (1H, *d*, *J* = 14.9 Hz, -OCH_2_), 4.39 (1H, *m*, H-2′), 3.63 (3H, *s*, OCH_3_), 1.60 (3H, *m*, H-3′ and H-4′), 0.88 (3H, *d*, *J* = 6.2 Hz, H-5′), 0.82 (3H, *d*, *J* = 6.2 Hz, H-6′) ppm. ^13^C NMR (100.6 MHz, DMSO-*d_6_*): *δ =* 174.9 (C-9), 172.5 (C-1′), 167.1 (C=O amide), 163.3 (C-3), 157.2 (C-4a), 155.6 (C-10a), 135.1 (C-6), 127.5 (C-1), 125.9 (C-8), 124.4 (C-7), 121.2 (C-8a), 117.9 (C-5), 115.3 (C-9a), 114.1 (C-2), 101.5 (C-4), 66.9 (-OCH_2_), 51.9 (-OCH_3_), 50.0 (C-2′), 24.2 (C-3′), 22.7 (C-4′), 21.1 (C-5′ and C-6′) ppm.

#### 3.1.6. Methyl (2-((9-Oxo-9H-xanthen-3-yl)oxy)acetyl)-D-leucinate (**7**)

The coupling reaction finished in 30 min. After extraction the product was recrystallized with dichloromethane and *n*-hexane to afford compound **7** as a white solid (142.84 mg, 93.5%); m.p.: 110–111 °C; IR (KBr): ṽ 3387, 1746, 1672, 1622, 1528, 1466, 1438, 1258, 827, 754, 666cm^−1^. ^1^H NMR (400.14 MHz, DMSO-*d*_6_): *δ =* 8.62 (1H, *d*, *J* = 7.9 Hz, N-H), 8.18 (1H, *dd*, *J* = 8.0 and 1.5 Hz, H-8), 8.13 (1H, *d*, *J* = 8.5 H-1), 7.85 (1H, *ddd*, *J* = 8.6, 7.0 and 1.6 Hz, H-6), 7.63 (1H, *dd*, *J* = 8.5 and 1.0 Hz, H-5), 7.48 (1H, *ddd*, *J* = 8.0, 7.1 and 0.9 Hz, H-7); 7.11 (1H, *dd*, *J* = 8.5 and 2.4 Hz, H-2), 7.10 (1H, *d*, *J* = 2.4 Hz, H-4), 4.82 (1H, *d*, *J* = 14.9 Hz, -OCH_2_), 4.77 (1H, *d*, *J* = 14.9 Hz, -OCH_2_), 4.39 (1H, *m*, H-2′), 3.63 (3H, *s*, OCH_3_), 1.60 (3H, *m*, H-3′ and H-4′), 0.88 (3H, *d*, *J* = 6.2 Hz, H-5′), 0.82 (3H, *d*, *J* = 6.2 Hz, H-6′) ppm. ^13^C NMR (100.6 MHz, DMSO-*d*_6_): *δ =* 174.9 (C-9), 172.5 (C-1′), 167.1 (C=O amide), 163.3 (C-3), 157.2 (C-4a), 155.6 (C-10a), 135.1 (C-6), 127.5 (C-1), 125.9 (C-8), 124.4 (C-7), 121.2 (C-8a), 117.9 (C-5), 115.3 (C-9a), 114.1 (C-2), 101.5 (C-4), 66.9 (-OCH_2_), 51.9 (-OCH_3_), 50.0 (C-2′), 24.2 (C-3′), 22.7 (C-4′), 21.1 (C-5′ and C-6′) ppm.

#### 3.1.7. Methyl (2-((9-Oxo-9H-xanthen-3-yl)oxy)acetyl)-L-prolinate (**8**)

The coupling reaction finished in 24 h. After extraction the product was recrystallized with dichloromethane and *n*-hexane to afford compound **8** as a white solid (117.6 mg, 83.3%); m.p.: 132–134 °C. IR (KBr): ṽ 1742, 1671, 1623, 1559, 1540, 1498, 1465, 1434, 1259, 834, 822, 765, 668 cm^−1^. ^1^H NMR (400.14 MHz, DMSO-*d*_6_): *δ =* 8.18 (1H, *dd*, *J* = 7.9 and 1.5 Hz, H-8), 8.10 (1H, *d*, *J* = 8.8 Hz, H-1), 7.85 (1H, *ddd*, *J* = 8.6, 7.0 and 1.6 Hz, H-6), 7.63 (1H, *dd*, *J* = 8.4 and 1.0 Hz, H-5), 7.47 (1H, *ddd*, *J* = 7.9, 7.1 and 0.9 Hz, H-7); 7.06 (1H, *dd*, *J* = 8.8 and 2.4 Hz, H-2), 7.02 (1H, *d*, *J* = 2.4 Hz, H-4), 5.06 (2H, *s*, -OCH_2_), 4.36 (1H, *dd*, *J* = 8.6 and 4.6 Hz, H-2′), 3.66 (2H, *m*, H-5′), 3.62 (3H, *s*, OCH_3_), 2.21 (1H, *t*, H-3′), 1.97 (2H, *m*, H-4′) and 1.87 (1H, *m*, H-3′) ppm. ^13^C NMR (100.6 MHz, DMSO-*d_6_*): *δ =* 174.9 (C-9), 172.1 (C-1′), 165.3 (C=O amide), 163.7 (C-3), 157.3 (C-4a), 155.6 (C-10a), 135.1 (C-6), 127.4 (C-1), 125.9 (C-8), 124.4 (C-7), 121.2 (C-8a), 117.9 (C-5), 115.2 (C-9a), 114.0 (C-2), 101.5 (C-4), 66.1 (-OCH_2_), 58.6 (C-2′), 51.8 (-OCH_3_), 45.3 (C-5′), 28.4 (C-3′) and 24.5 (C-4′) ppm.

#### 3.1.8. Methyl (2-((9-Oxo-9H-xanthen-3-yl)oxy)acetyl)-D-prolinate (**9**)

The coupling reaction finished in 22 h. The extraction afforded a yellow oil product. Crystallization was not possible. IR (KBr): ṽ 1742, 1654, 1623, 1569, 1498, 1465, 1443, 1433, 1260, 834, 822, 765, 690, 668, 602 cm^−1^. ^1^H NMR (400.14 MHz, DMSO-*d*_6_): *δ =* 8.18 (1H, *dd*, *J* = 7.9 and 1.5 Hz, H-8), 8.10 (1H, *d*, *J* = 8.8 Hz, H-1), 7.85 (1H, *ddd*, *J* = 8.6, 7.0 and 1.6 Hz, H-6), 7.63 (1H, *dd*, *J* = 8.4 and 1.6 Hz, H-5), 7.47 (1H, *ddd*, *J* = 7.9, 7.1 and 0.9 Hz, H-7); 7.06 (1H, *dd*, *J* = 8.8 and 2.4 Hz, H-2), 7.02 (1H, *d*, *J* = 2.4 Hz, H-4), 5.06 (2H, *s*, -OCH_2_), 4.36 (1H, *dd*, *J* = 8.6 and 4.6 Hz, H-2′), 3.66 (2H, *m*, H-5′), 3.62 (3H, *s*, OCH_3_), 2.21 (1H, *m*, H-3′), 1.97 (2H, *m*, H-4′) and 1.87 (1H, *m*, H-3′) ppm. ^13^C NMR (100.6 MHz, DMSO-*d*_6_): *δ* = 174.9 (C-9), 172.1 (C-1′), 165.3 (C=O amide), 163.7 (C-3), 157.3 (C-4a), 155.6 (C-10a), 135.1 (C-6), 127.4 (C-1), 125.9 (C-8), 124.4 (C-7), 121.2 (C-8a), 117.9 (C-5), 115.2 (C-9a), 114.0 (C-2), 101.5 (C-4), 66.1 (-OCH_2_), 58.6 (C-2′), 51.8 (-OCH_3_), 45.3 (C-5′), 28.4 (C-3′) and 24.5 (C-4′) ppm.

#### 3.1.9. Methyl (2-((9-Oxo-9H-xanthen-3-yl)oxy)acetyl)-L-valinate (**10**)

The coupling reaction finished in 4 h. After extraction, the product was recrystallized with dichloromethane and *n*-hexane to afford compound **10** as a white solid (102.76 mg, 73.5%); m.p.: 110–112 °C; IR (KBr): ṽ 3421, 1727, 1662, 1620, 1539, 1466, 1437, 1258, 863, 833, 751, 669 cm^−1^. ^1^H NMR (400.14 MHz, DMSO-*d*_6_): *δ* = 8.5 (1H, *d*, *J* = 8.2 Hz, N-H), 8.17 (1H, *dd*, *J* = 7.9 and 1.5 Hz, H-8), 8.12 (1H, *d*, *J* = 8.9 Hz, H-1), 7.85 (1H, *ddd*, *J* = 8.6, 7.0 and 1.6 Hz, H-6), 7.63 (1H, *dd*, *J* = 8.5 and 1.6 Hz, H-5), 7.47 (1H, *ddd*, *J* = 8.0, 7.1 and 0.9 Hz, H-7); 7.10 (1H, *dd*, *J* =8.8 and 2.4 Hz, H-2), 7.10 (1H, *d*, *J* = 2.4 Hz, H-4), 4.86 (1H, *d*, *J* = 14.8 Hz, -OCH_2_), 4.81 (1H, *d*, *J* = 14.8 Hz, -OCH_2_), 4.25 (1H, *dd*, *J* = 8.2 and 6.4 Hz, H-2′), 3.66 (3H, *s*, OCH_3_), 2.11 (1H, *m*, H-3′), 0.92 (3H, *d*, *J* = 7.0 Hz, H-4′) and 0.90 (3H, *d*, *J* = 7.0 Hz, H-5′) ppm. ^13^C NMR (100.6 MHz, DMSO-*d*_6_): *δ* = 175.4 (C-9), 172.2 (C-1′), 167.7 (C=O amide), 164.0 (C-3), 157.8 (C-4a), 156.1 (C-10a), 135.6 (C-6), 128.1 (C-1), 126.4 (C-8), 124.9 (C-7), 121.7 (C-8a), 118.4 (C-5), 115.8 (C-9a), 114.4 (C-2), 102.0 (C-4), 67.3 (-OCH_2_), 57.8 (C-2′), 52.3 (-OCH_3_), 30.3 (C-3′), 19.4 (C-4′) and 18.7 (C-5′) ppm.

#### 3.1.10. Methyl (2-((9-Oxo-9H-xanthen-3-yl)oxy)acetyl)-D-valinate (**11**)

The coupling reaction finished in 2 h. After extraction, the product was recrystallized with dichloromethane and *n*-hexane to afford compound **11** as a white solid (108.5 mg, 75.1%); m.p.: 112–113 °C; IR (KBr): ṽ 3446, 1747, 1652, 1621, 1540, 1489, 1465, 1457, 1436, 1256, 756, 668 cm^−1^. ^1^H NMR (400.14 MHz, DMSO-*d*_6_): *δ* = 8.5 (1H, *d*, *J* = 8.2 Hz, N-H), 8.17 (1H, *dd*, *J* = 7.9 and 1.5 Hz, H-8), 8.12 (1H, *d*, *J* = 8.8 Hz, H-1), 7.85 (1H, *ddd*, *J* = 8.6, 7.0 and 1.6 Hz, H-6), 7.63 (1H, *dd*, *J* = 8.5 and 1.0 Hz, H-5), 7.47 (1H, *ddd*, *J* = 8.0, 7.1 and 0.9 Hz, H-7); 7.10 (1H, *dd*, *J* =9.5 and 2.4 Hz, H-2), 7.10 (1H, *d*, *J* = 2.4 Hz, H-4), 4.86 (1H, *d*, *J* = 14.8 Hz, -OCH_2_), 4.81 (1H, *d*, *J* = 14.8 Hz, -OCH_2_), 4.25 (1H, *dd*, *J* = 8.2 and 6.4 Hz, H-2′), 3.66 (3H, *s*, OCH_3_), 2.11 (1H, *m*, H-3′), 0.92 (3H, *d*, *J* = 7.0 Hz, H-4′) and 0.90 (3H, *d*, *J* = 7.0 Hz, H-5′) ppm. ^13^C NMR (100.6 MHz, DMSO-*d_6_*): *δ =* 175.4 (C-9), 172.2 (C-1′), 167.7 (C=O amide), 164.0 (C-3), 157.8 (C-4a), 156.1 (C-10a), 135.6 (C-6), 128.1 (C-1), 126.4 (C-8), 124.9 (C-7), 121.7 (C-8a), 118.4 (C-5), 115.8 (C-9a), 114.4 (C-2), 102.0 (C-4), 67.3 (-OCH_2_), 57.8 (C-2′), 52.3 (-OCH_3_), 30.3 (C-3′), 19.4 (C-4′) and 18.7 (C-5′) ppm.

#### 3.1.11. Methyl (S)-2-(2-((9-Oxo-9H-xanthen-3-yl)oxy)acetamido)-2-phenylacetate (**12**)

The coupling reaction finished in 1 h. After extraction, the product was recrystallized with methanol containing a few drops of THF and water to afford compound **12** as a white solid (99.17 mg, 64%); m.p.: 148–150 °C; IR (KBr): ṽ 3340, 1735, 1664, 1621, 1537, 1497, 1464, 1442, 1255, 853, 823, 760, 728, 697, 668 cm^−1^. ^1^H NMR (300.13 MHz, DMSO-*d*_6_): *δ* = 9.12 (1H, *d*, *J* = 7.2 Hz, N-H), 8.18 (1H, *dd*, *J* = 7.9 and 1.5 Hz, H-8), 8.11 (1H, *d*, *J* = 8.8 Hz, H-1), 7.86 (1H, *ddd*, *J* = 9.0, 7.0 and 1.6 Hz, H-6), 7.64 (1H, *dd*, *J* = 8.4 and 0.9 Hz, H-5), 7.48 (1H, *ddd*, *J* = 8.0, 7.1 and 0.9 Hz, H-7), 7.42 (2H, *m*, H-5′), 7.41 (2H, *m*, H-4′), 7.36 (1H, *m*, H-6′), 7.09 (1H, *dd*, *J* = 8.8 and 2.4 Hz, H-2), 7.08 (1H, *d*, *J* = 2.4 Hz, H-4), 5.52 (1H, *d*, *J* = 7.2 Hz, H-2′), 4.89 (1H, *d*, *J* = 15.0 Hz, -OCH_2_), 4.83 (1H, *d*, *J* = 14.8 Hz, -OCH_2_), 3.65 (3H, *s*, OCH_3_) ppm. ^13^C NMR (75.5 MHz, DMSO-*d*_6_): *δ* = 174.9 (C-9), 170.7 (C-1′), 167.0 (C=O amide), 163.5 (C-3), 157.3 (C-4a), 155.6 (C-10a), 136.0 (C-3′), 135.2 (C-6), 128.7 (C-4′), 128.4 (C-5′), 127.8 (C-6′), 127.6 (C-1), 125.9 (C-8), 124.4 (C-7), 121.2 (C-8a), 118.0 (C-5), 115.3 (C-9a), 114.0 (C-2), 101.5 (C-4), 66.7 (OCH_2_), 55.1 (C-2′), 52.4 (OCH_3_) ppm.

#### 3.1.12. Methyl (R)-2-(2-((9-Oxo-9H-xanthen-3-yl)oxy)acetamido)-2-phenylacetate (**13**)

The coupling reaction finished in 30 min. After extraction, the product was recrystallized with methanol containing a few drops of THF and water to afford compound **13** as a white solid (117.56 mg, 75.9%); m.p.: 152–154 °C; IR (KBr): ṽ 3297, 1738, 1663, 1621, 1540, 1497, 1464, 1442, 1256, 851, 760, 728, 697, 667 cm^−1^. ^1^H NMR (300.13 MHz, DMSO-*d*_6_): *δ* = 9.12 (1H, *d*, *J* = 7.2 Hz, N-H), 8.18 (1H, *dd*, *J* = 7.9 and 1.5 Hz, H-8), 8.11 (1H, *d*, *J* = 8.8 Hz, H-1), 7.86 (1H, *ddd*, *J* = 9.0, 7.0 and 1.6 Hz, H-6), 7.64 (1H, *dd*, *J* = 8.4 and 0.9 Hz, H-5), 7.48 (1H, *ddd*, *J* = 8.0, 7.1 and 0.9 Hz, H-7), 7.42 (2H, *m*, H-5′), 7.41 (2H, *m*, H-4′), 7.36 (1H, *m*, H-6′), 7.09 (1H, *dd*, *J* = 8.8 and 2.4 Hz, H-2), 7.08 (1H, *d*, *J* = 2.4 Hz, H-4), 5.52 (1H, *d*, *J* = 7.2 Hz, H-2′), 4.89 (1H, *d*, *J* = 15.0 Hz, -OCH_2_), 4.83 (1H, *d*, *J* = 14.8 Hz, -OCH_2_), 3.65 (3H, *s*, OCH_3_) ppm. ^13^C NMR (75.5 MHz, DMSO-*d*_6_): *δ* = 174.9 (C-9), 170.7 (C-1′), 167.0 (C=O amide), 163.5 (C-3), 157.3 (C-4a), 155.6 (C-10a), 136.0 (C-3′), 135.2 (C-6), 128.7 (C-4′), 128.4 (C-5′), 127.8 (C-6′), 127.6 (C-1), 125.9 (C-8), 124.4 (C-7), 121.2 (C-8a), 118.0 (C-5), 115.3 (C-9a), 114.0 (C-2), 101.5 (C-4), 66.7 (OCH_2_), 55.1 (C-2′), 52.4 (OCH_3_) ppm.

#### 3.1.13. Methyl (2-((9-Oxo-9H-xanthen-3-yl)oxy)acetyl)-L-phenylalaninate (**14**)

The coupling reaction finished in 5 h. The extraction afforded compound **14** as a white crystal solid (130.84 mg, 80.1%); m.p.: 152–154 °C; IR (KBr): ṽ 3306, 1733, 1655, 1620, 1544, 1496, 1464, 1439, 1258, 844, 817, 768, 746, 695, 667 cm^−1^. ^1^H NMR (400.14 MHz, DMSO-*d*_6_): *δ* = 8.64 (1H, *d*, *J* =7.9 Hz, N-H), 8.18 (1H, *dd*, *J* = 7.9 and 1.6 Hz, H-8), 8.10 (1H, *d*, *J* = 8.8 Hz, H-1), 7.86 (1H, *ddd*, *J* = 8.5, 7.0 and 1.6 Hz, H-6), 7.64 (1H, *dd*, *J* = 8.4 and *J* = 1.0 Hz, H-5), 7.48 (1H, *ddd*, *J* = 8.1, 7.1 and 1.1 Hz H-7); 7.21 (2H, *m*, H-5′), 7.20 (2H, *m*, H-6′), 7.14 (1H, *m*, H-7′), 7.04 (1H, *dd*, *J* = 8.8 and 2.4 Hz, H-2), 6.99 (1H, *d*, *J* = 2.3 Hz, H-4), 4.74 (1H, *d*, *J* = 15.1 Hz, -OCH_2_), 4.69 (1H, *d*, *J* = 15.1 Hz, -OCH_2_), 4.59 (1H, *m*, H-2′), 3.64 (3H, *s*, OCH_3_), 3.12 (1H, *dd*, *J* = 13.8 and 5.1 Hz, H-3′), 2.99 (1H, *dd*, *J* = 9.6 and 5.1 Hz, H-3′) ppm. ^13^C NMR (100.6 MHz, DMSO-*d*_6_): *δ* = 174.9 (C-9), 171.6 (C-1′), 167.1 (C=O amide), 163.2 (C-3), 157.2 (C-4a), 155.6 (C-10a), 137.1 (C-4′), 135.2 (C-6), 129.0 (C-6′), 128.2 (C-5′), 127.6 (C-1), 126.4 (C-7′), 125.9 (C-8), 124.4 (C-7), 121.2 (C-8a), 117.9 (C-5), 115.4 (C-9a), 114.0 (C-2), 101.5 (C-4), 66.9 (-OCH_2_), 53.2 (C-2′), 52.0 (-OCH_3_), 36.3 (C-3′) ppm.

#### 3.1.14. Methyl (2-((9-Oxo-9H-xanthen-3-yl)oxy)acetyl)-D-phenylalaninate (**15**)

The coupling reaction finished in 5 h. The extraction afforded compound **15** as a white crystal solid (119.11 mg, 73.97%); m.p.: 151–153 °C; IR (KBr): ṽ 3306, 1733, 1655, 1620, 1544, 1496, 1464, 1439, 1258, 844, 817, 768, 746, 695, 667 cm^−1^. ^1^H NMR (400.14 MHz, DMSO-*d*_6_): *δ* = 8.64 (1H, *d*, *J* =7.9 Hz, N-H), 8.18 (1H, *dd*, *J* = 7.9 and 1.6 Hz, H-8), 8.10 (1H, *d*, *J* = 8.8 Hz, H-1), 7.86 (1H, *ddd*, *J* = 8.5, 7.0 and 1.6 Hz, H-6), 7.64 (1H, *dd*, *J* = 8.4 and and *J* = 1.0 Hz, H-5), 7.48 (1H, *ddd*, *J* = 8.1, 7.1 and 1.1 Hz, H-7); 7.21 (2H, *m*, H-5′), 7.20 (2H, *m*, H-6′), 7.14 (1H, *m*, H-7′), 7.04 (1H, *dd*, *J* = 8.8 and 2.4 Hz, H-2), 6.99 (1H, *d*, *J* = 2.3 Hz, H-4), 4.74 (1H, *d*, *J* = 15.1 Hz, -OCH_2_), 4.69 (1H, *d*, *J* = 15.1 Hz, -OCH_2_), 4.59 (1H, *m*, H-2′), 3.64 (3H, *s*, OCH_3_), 3.12 (1H, *dd*, *J* = 13.8 and 5.1 Hz, H-3′), 2.99 (1H, *dd*, *J* = 9.6 and 5.1 Hz, H-3′) ppm. ^13^C NMR (100.6 MHz, DMSO-*d*_6_): *δ* = 174.9 (C-9), 171.6 (C-1′), 167.1 (C=O amide), 163.2 (C-3), 157.2 (C-4a), 155.6 (C-10a), 137.1 (C-4′), 135.2 (C-6), 129.0 (C-6′), 128.2 (C-5′), 127.6 (C-1), 126.4 (C-7′), 125.9 (C-8), 124.4 (C-7), 121.2 (C-8a), 117.9 (C-5), 115.4 (C-9a), 114.0 (C-2), 101.5 (C-4), 66.9 (-OCH_2_), 53.2 (C-2′), 52.0 (-OCH_3_), 36.3 (C-3′) ppm.

#### 3.1.15. Methyl (2-((9-Oxo-9H-xanthen-3-yl)oxy)acetyl)-L-tryptophanate (**16**)

The coupling reaction finished in 1 h. After extraction, the product was recrystallized with dichloromethane and *n*-hexane to afford compound **16** as a white solid (98.92 mg, 56.7%); m.p.: 215–216 °C; IR (KBr): ṽ 3310, 1739, 1646, 1617, 1540, 1507, 1497, 1489, 1464, 1436, 1328, 1258, 852, 761, 740, 668 cm^−1^. ^1^H NMR (300.13 MHz, DMSO-*d*_6_): *δ* = 10.89 (1H, *d*, *J* = 2.4 Hz, N-H_arom_), 8.56 (1H, *d*, *J* = 7.7 Hz, N-H), 8.18 (1H, *dd*, *J* = 8.0 and 1.7 Hz, H-8), 8.10 (1H, *d*, *J* = 8.9 Hz, H-1), 7.86 (1H, *ddd*, *J* = 8.6, 7.1 and 1.6 Hz, H-6), 7.63 (1H, *dd*, *J* = 8.4 and 1.0 Hz, H-5), 7.51 (1H, dd*, H-10′), 7.46 (1H, *m*, H-7), 7.32 (1H, *dt*, *J* = 7.9 and *J* = 1.0 Hz, H-7′), 7.18 (1H, *d*, *J* = 2.4 Hz, H-5′), 7.07 (1H, *dd*, *J* = 8.9 Hz and *J* = 2.3, H-2), 7.07 (1H, *d*, *J* = 2.3 H-4), 7.03 (1H, *m*, H-8′), 6.95 (1H, *m*, H-9′), 4.74 (1H, *d*, *J* = 15.1 Hz, -OCH_2_), 4.70 (1H, *d*, *J* = 15.2 Hz, -OCH_2_), 4.62 (1H, *m*, H-2′), 3.62 (3H, *s*, OCH_3_), 3.25 (1H, *dd*, *J* = 14.7 and 5.5 Hz, H-3′), 3.16 (1H, *dd*, *J* = 14.6 and 8.4 Hz, H-3′) ppm. ^13^C NMR (75.5 MHz, DMSO-*d*_6_): *δ* = 175.0 (C-9), 171.9 (C-1′), 167.1 (C=O amide), 163.3 (C-3), 157.3 (C-4a), 155.7 (C-10a), 136.1 (C-6′), 135.2 (C-6), 127.7 (C-1), 127.1 (C-9′), 125.9 (C-8), 124.5 (C-7 and C-11′), 123.8 (C-5′), 121.2 (C-8a), 121.0 (C-8′), 118.5 (C-9′ and C-10′), 118.0 (C-5), 115.4 (C-9a), 113.9 (C-2), 111.5 (C-7′), 109.3 (C-4′), 101.6 (C-4), 66.9 (-OCH_2_), 52.1 (-OCH_3_), 47.0 (C-2′), 37.9 (C-3′) ppm.

#### 3.1.16. Methyl (2-((9-Oxo-9H-xanthen-3-yl)oxy)acetyl)-D-tryptophanate (**17**)

The coupling reaction finished in 2 h. After extraction, the product was recrystallized with methanol and water to afford compound **17** as a white solid (135.68 mg, 77.4%); m.p.: 214–215 °C; IR (KBr): ṽ 3279, 1749, 1647, 1621, 1540, 1506, 1496, 1489, 1465, 1447, 1323, 1284, 867, 838, 815, 786, 769, 754, 741, 670 cm^−1^. ^1^H NMR (300.13 MHz, DMSO-*d*_6_): *δ* = 10.89 (1H, *d*, *J* = 2.4 Hz, N-H_arom_), 8.56 (1H, *d*, *J* = 7.7 Hz, N-H), 8.18 (1H, *dd*, *J* = 8.0 and 1.7 Hz, H-8), 8.10 (1H, *d, J* = 8.9 Hz, H-1), 7.86 (1H, *ddd*, *J* = 8.6, 7.1 and 1.6 Hz, H-6), 7.63 (1H, *dd*, *J* = 8.4 and 1.0 Hz, H-5), 7.51 (1H, dd*, H-10′), 7.46 (1H, *m*, H-7), 7.32 (1H, *dt*, *J* = 7.9 and *J* = 1.0 Hz, H-7′), 7.18 (1H, *d*, *J* = 2.3 Hz, H-5′), 7.07 (1H, *dd*, *J* = 8.9 and *J* = 2.3 Hz, H-2), 7.07 (1H, *J* = 2.3 Hz, H-4), 7.03 (1H, *m*, H-8′), 6.95 (1H, *m*, H-9′), 4.74 (1H, *d*, *J* = 15.1 Hz, -OCH_2_), 4.70 (1H, *d*, *J* = 15.2 Hz, -OCH_2_), 4.62 (1H, *m*, H-2′), 3.62 (3H, *s*, OCH_3_), 3.25 (1H, *dd*, *J* = 14.7 and 5.5 Hz, H-3′), 3.16 (1H, *dd*, *J* = 14.6 and 8.4 Hz, H-3′) ppm. ^13^C NMR (75.5 MHz, DMSO-*d*_6_): *δ* = 175.0 (C-9), 171.9 (C-1′), 167.1 (C=O amide), 163.3 (C-3), 157.3 (C-4a), 155.7 (C-10a), 136.1 (C-6′), 135.2 (C-6), 127.7 (C-1), 127.1 (C-9′), 125.9 (C-8), 124.5 (C-7 and C-11′), 123.8 (C-5′), 121.2 (C-8a), 121.0 (C-8′), 118.5 (C-9′ and C-10′), 118.0 (C-5), 115.4 (C-9a), 113.9 (C-2), 111.5 (C-7′), 109.3 (C-4′), 101.6 (C-4), 66.9 (-OCH_2_), 52.1 (-OCH_3_), 47.0 (C-2′), 37.9 (C-3′) ppm.

#### 3.1.17. Methyl (2-((9-Oxo-9H-xanthen-3-yl)oxy)acetyl)-L-tyrosinate (**18**)

The coupling reaction finished in 4 h. After extraction, the product was recrystallized with dichloromethane and *n*-hexane to afford compound **18** as a white solid (82.05 mg, 49.4%); m.p.: 194–197 °C; IR (KBr): ṽ 3550–3300, 3014, 1740, 1649, 1623, 1543, 1465, 1435, 1401, 1255, 847, 836, 804, 762, 717, 671, 663 cm^−1^. ^1^H NMR (400.14 MHz, DMSO-*d*_6_): *δ* = 9.23 (1H, *s*, O-H), 8.56 (1H, *d*, *J* = 7.9 Hz, N-H), 8.18 (1H, *dd*, *J* = 8.0 and 1.6 Hz, H-8), 8.12 (1H, *d*, *J* = 8.8 Hz, H-1), 7.86 (1H, *ddd*, *J* = 8.6, 7.0 and 1.6 Hz, H-6), 7.65 (1H, *dd*, *J* = 8.4 and 1.0 Hz, H-5), 7.48 (1H, *ddd*, *J* = 7.9, 7.2 and 0.9 Hz, H-7), 7.05 (1H, *dd*, *J* = 9.5 and 2.4 Hz, H-2), 7.05 (1H, *d*, *J* = 2.2 Hz, H-4), 7.00 (2H, *d*, *J* = 8.5Hz, H-5′), 6.63 (2H, *d*, *J* = 8.5 Hz, H-6′), 4.97 (1H, *d*, *J* = 14.8 Hz, -OCH_2_), 4.74 (1H, *d*, *J* = 15.5 Hz, -OCH_2_), 4.49 (1H, *m*, H-2′), 3.62 (3H, *s*, OCH_3_), 2.98 (1H, *dd*, *J* = 13.9 and 5.3 Hz, H-3′), 2.88 (1H, *dd*, *J* = 13.9 and 9.2 Hz, H-3′) ppm. ^13^C NMR (100.6 MHz, DMSO-*d*_6_): *δ* = 174.9 (C-9), 171.7 (C-1′), 167.0 (C=O amide), 163.3 (C-3), 157.3 (C-4a), 156.0 (C-7′), 155.6 (C-10a), 135.2 (C-6), 130.0 (C-5′), 127.6 (C-1), 127.0 (C-4′), 125.9 (C-8), 124.4 (C-7), 121.2 (C-8a), 117.9 (C-5), 115.4 (C-9a) 115.0 (C-6′), 114.0 (C-2), 101.6 (C-4), 66.9 (OCH_2_), 52.0 (-OCH_3_), 47.0 (C-2′), 35.7 (C-3′) ppm.

#### 3.1.18. Methyl (2-((9-Oxo-9H-xanthen-3-yl)oxy)acetyl)-D-tyrosinate (**19**)

The coupling reaction finished in 30 min. After extraction, the product was recrystallized with dichloromethane and *n*-hexane to afford compound **19** as a white solid (99.89 mg, 59.1%). m.p.: 193–196 °C; IR (KBr): ṽ 3550–3300, 3014, 1740, 1649, 1622, 1541, 1464, 1435, 1255, 847, 835, 803, 762, 716, 663 cm^−1^. ^1^H NMR (400.14 MHz, DMSO-*d*_6_): *δ* = 9.23 (1H, *s*, O-H), 8.56 (1H, *d*, *J* = 7.9 Hz, N-H), 8.18 (1H, *dd*, *J* = 8.0 and 1.6 Hz, H-8), 8.12 (1H, *d*, *J* = 9.5 Hz, H-1), 7.86 (1H, *ddd*, *J* = 8.6, 7.0 and 1.6 Hz, H-6), 7.65 (1H, *dd*, *J* = 8.4 and 0.6 Hz, H-5), 7.48 (1H, *ddd*, *J* = 7.9, 7.2 and 0.9 Hz, H-7), 7.05 (1H, *dd*, *J* = 9.5 and 2.4 Hz, H-2), 7.05 (1H, *d*, *J* = 2.2 Hz, H-4), 7.00 (2H, *d*, *J* = 8.5Hz, H-5′), 6.63 (2H, *d*, *J* = 8.5 Hz, H-6′), 4.97 (1H, *d*, *J* = 14.8 Hz, -OCH_2_), 4.74 (1H, *d*, *J* = 15.5 Hz, -OCH_2_), 4.49 (1H, *m*, H-2′), 3.62 (3H, *s*, OCH_3_), 2.98 (1H, *dd*, *J* = 13.9 and 5.3 Hz, H-3′), 2.88 (1H, *dd*, *J* = 13.9 and 9.2 Hz, H-3′) ppm.^13^C NMR (100.6 MHz, DMSO-*d*_6_): *δ* = 174.9 (C-9), 171.7 (C-1′), 167.0 (C=O amide), 163.3 (C-3), 157.3 (C-4a), 156.0 (C-7′), 155.6 (C-10a), 135.2 (C-6), 130.0 (C-5′), 127.6 (C-1), 127.0 (C-4′), 125.9 (C-8), 124.4 (C-7), 121.2 (C-8a), 117.9 (C-5), 115.4 (C-9a) 115.0 (C-6′), 114.0 (C-2), 101.6 (C-4), 66.9 (OCH_2_), 52.0 (-OCH_3_), 47.0 (C-2′), 35.7 (C-3′) ppm.

#### 3.1.19. Methyl (2-((9-Oxo-9H-xanthen-3-yl)oxy)acetyl)-L-serinate (**20**)

Firstly, compound **20** was synthesized using COMU. The coupling reaction finished in 2 h with a yield of 42%. However, the product incorporated a lot of impurities and was difficult to purify. Thus, TBTU was used in a coupling reaction that finished in 27 h. The extraction afforded compound **20** as a white crystal solid (38.32 mg, 49.9%). m.p.: 166–167 °C; IR (KBr): ṽ 3550–3300, 3318, 1740, 1661, 1645, 1617, 1558, 1498, 1465, 1443, 1262, 1235, 836, 761, 669 cm^−1^. ^1^H NMR (400.14 MHz, DMSO-*d*_6_): *δ* = 8.46 (1H, *d*, *J* = 7.8 Hz, N-H), 8.18 (1H, *dd*, *J* = 8.0 and 1.5 Hz, H-8), 8.13 (1H, *d*, *J* = 8.7 Hz, H-1), 7.85 (1H, *ddd*, *J* = 8.6, 7.0 and 1.6 Hz, H-6), 7.63 (1H, *dd*, *J* = 8.4 and 1.0 Hz, H-5), 7.47 (1H, *ddd, J* = 8.0, 7.2 and 1.0 Hz, H-7); 7.12 (1H, *dd*, *J* = 8.7 and 2.4 Hz, H-2), 7.10 (1H, *d*, *J* = 2.4 Hz, H-4), 5.74 (1H, *s*, OH), 4.84 (1H, *d*, *J* = 15.4 Hz, -OCH_2_), 4.80 (1H, *d*, *J* = 15.6 Hz, -OCH_2_), 4.46 (1H, *m*, H-2′), 3.79 (1H, *dd*, *J* = 11.1 and 5.2 Hz, H-3′), 3.70 (1H, *dd*, *J* = 10.9 and 4.3Hz, H-3′), 3.65 (3H, *s*, OCH_3_) ppm. ^13^C NMR (100.6 MHz, DMSO-*d*_6_): *δ* = 174.9 (C-9), 170.7 (C-1′), 167.1 (C=O amide), 163.3 (C-3), 157.3 (C-4a), 155.6 (C-10a), 135.2 (C-6), 127.6 (C-1), 125.9 (C-8), 124.4 (C-7), 121.2 (C-8a), 117.9 (C-5), 115.4 (C-9a), 114.0 (C-2), 101.6 (C-4), 66.9 (OCH_2_), 61.0 (C-2′), 54.5 (C-3′), 52.0 (-OCH_3_) ppm.

#### 3.1.20. Methyl (2-((9-Oxo-9H-xanthen-3-yl)oxy)acetyl)-D-serinate (**21**)

Compound **21** was first synthesized using COMU. The coupling reaction finished in 2 h with a yield of 64%. However, the product incorporated a lot of impurities and was difficult to purify. Thus, TBTU was used in a coupling reaction that finished in 27 h. The extraction afforded a white crystal solid (40.39 mg, 53.3%). m.p.: 166–167 °C; IR (KBr): ṽ 3550–3300, 3319, 1741, 1662, 1645, 1617, 1558, 1498, 1466, 1443, 1262, 1236, 836, 761, 670 cm^−1^. ^1^H NMR (400.14 MHz, DMSO-*d*_6_): *δ* = 8.46 (1H, *d*, *J* = 7.8 Hz, N-H), 8.18 (1H, *dd*, *J* = 8.0 and 1.5 Hz, H-8), 8.13 (1H, *d*, *J* = 8.7 Hz, H-1), 7.85 (1H, *ddd*, *J* = 8.6, 7.0 and 1.6 Hz, H-6), 7.63 (1H, *dd*, *J* = 8.4 and 1.0 Hz, H-5), 7.47 (1H, *ddd*, *J* = 8.0, 7.2 and 0.8 Hz, H-7); 7.12 (1H, *dd*, *J* = 8.7 and 2.4 Hz, H-2), 7.10 (1H, *d*, *J* = 2.4 Hz, H-4), 5.74 (1H, *s*, OH), 4.84 (1H, *d*, *J* = 15.4 Hz, -OCH_2_), 4.80 (1H, *d*, *J* = 15.6 Hz, -OCH_2_), 4.46 (1H, *m*, H-2′), 3.79 (1H, *dd*, *J* = 11.1 and 5.2 Hz, H-3′), 3.70 (1H, *dd*, *J* = 10.9 and 4.3Hz, H-3′), 3.65 (3H, *s*, OCH_3_) ppm. ^13^C NMR (100.6 MHz, DMSO-*d*_6_): *δ* = 174.9 (C-9), 170.7 (C-1′), 167.1 (C=O amide), 163.3 (C-3), 157.3 (C-4a), 155.6 (C-10a), 135.2 (C-6), 127.6 (C-1), 125.9 (C-8), 124.4 (C-7), 121.2 (C-8a), 117.9 (C-5), 115.4 (C-9a), 114.0 (C-2), 101.6 (C-4), 66.9 (OCH_2_), 61.0 (C-2′), 54.5 (C-3′), 52.0 (-OCH_3_) ppm.

#### 3.1.21. Dimethyl (2-((9-Oxo-9H-xanthen-3-yl)oxy)acetyl)-L-aspartate (**22**)

The coupling reaction finished in 30 h. After extraction, the product was recrystallized with dichloromethane and *n*-hexane to afford compound **22** as a white solid (85.8 mg, 55.9%). m.p.: 114–115 °C; IR (KBr): ṽ 3406, 1758, 1741, 1661, 1641, 1622, 1526, 1466, 1436, 1261, 826, 761, 666 cm^−1^. ^1^H NMR (400.14 MHz, DMSO-*d*_6_): *δ* = 8.73 (1H, *d*, *J* = 8.1 Hz, N-H), 8.16 (1H, *dd*, *J* = 7.9 and *J* = 1.6 Hz, H-8), 8.11 (1H, *d*, *J* = 9,1 Hz, H-1), 7.84 (1H, *ddd*, *J* = 8.7, 7.1 and 1.6, H-6), 7.62 (1H, *dd*, *J* = 8,7 Hz and *J* = 0.9, H-5), 7.46 (1H, *ddd*, *J* = 8.0, J = 7.2 and 1.0 Hz, H-7), 7.09 (1H, *dd*, *J* = 7.1 and 1.3 Hz, H-2), 7.07 (1H, *d*, *J* = 1.30 Hz, H-4), 4.78 (2H, *s*, OCH_2_), 4.78 (1H, *m*, H-2′), 3.64 (3H, *s*, OCH_3_), 3.60 (3H, *s*, C-5′), 2.90 (1H, *dd*, *J* = 16.5 and 6.0 Hz, H-3′), 2.79 (1H *dd*, *J* = 16.6 and 7.2 Hz) ppm. ^13^C NMR (100.6 MHz, DMSO-*d*_6_): *δ* = 175.0 (C-9), 171.0 (C-1′), 170.6 (C-4′), 167.1 (C=O amide), 163.2 (C-3), 157.3 (C-4a), 155.7 (C-10a), 135.2 (C-6), 127.7 (C-1), 126.0 (C-8), 124.5 (C-7), 121.2 (C-8a), 118.0 (C-5), 115.5 (C-9a), 114.1 (C-2), 101.6 (C-4), 67.0 (-OCH_2_), 52.4 (-OCH_3_), 51.7 (C-5′), 48.3 (C-2′), 35.4 (C-3′) ppm.

#### 3.1.22. Dimethyl (2-((9-Oxo-9H-xanthen-3-yl)oxy)acetyl)-D-aspartate (**23**)

The coupling reaction finished in 23 h. After extraction, the product was recrystallized with dichloromethane and *n*-hexane to afford compound **23** as a white solid (97.88 mg, 62.7%); m.p.: 118–119 °C; IR (KBr): ṽ 3406, 1758, 1741, 1661, 1641, 1622, 1526, 1466, 1436, 1261, 827, 761, 665 cm^−1^. ^1^H NMR (400.14 MHz, DMSO-*d*_6_): *δ* = 8.73 (1H, *d*, *J* = 8.1 Hz, N-H), 8.16 (1H, *dd*, *J* = 7.9 and *J* = 1.6 Hz, H-8), 8.11 (1H, *d*, *J* = 9,1 Hz, H-1), 7.84 (1H, *ddd*, *J* = 8.7, 7.1 and 1.6, H-6, H-6), 7.62 (1H, *dd*, *J* = 8,3 and *J* = 0.9 Hz, H-5), 7.46 (1H, *ddd*, *J* = 8.0, J = 7.2 and 1.0 Hz, H-7), 7.09 (1H, *dd*, *J* = 7.1 and 1.3 Hz, H-2), 7.07 (1H, *d*, *J* = 1.30 Hz, H-4), 4.76 (2H, *s*, OCH_2_), 4.78 (1H, *m*, H-2′), 3.64 (3H, *s*, OCH_3_), 3.60 (3H, *s*, C-5′), 2.90 (1H, *dd*, *J* = 16.5 and 6.0 Hz, H-3′), 2.79 (1H *dd*, *J* = 16.6 and 7.2 Hz) ppm. ^13^C NMR (100.6 MHz, DMSO-*d*_6_): *δ* = 175.0 (C-9), 171.0 (C-1′), 170.6 (C-4′), 167.1 (C=O amide), 163.2 (C-3), 157.3 (C-4a), 155.7 (C-10a), 135.2 (C-6), 127.7 (C-1), 126.0 (C-8), 124.5 (C-7), 121.2 (C-8a), 118.0 (C-5), 115.5 (C-9a), 114.1 (C-2), 101.6 (C-4), 67.0 (-OCH_2_), 52.4 (-OCH_3_), 51.7 (C-5′), 48.3 (C-2′), 35.4 (C-3′) ppm.

#### 3.1.23. Dimethyl (2-((9-Oxo-9H-xanthen-3-yl)oxy)acetyl)-L-glutamate (**24**)

The coupling reaction finished in 24 h. The extraction afforded compound **24** as a yellow solid (119 mg, 75.4%). m.p.: 100–103 °C; IR (KBr): ṽ 3276, 1754, 1732, 1664, 1620, 1562, 1463, 1441, 1264, 845, 760, 668 cm^−1^. ^1^H NMR (400.14 MHz, DMSO-*d*_6_): *δ* = 8.64 (1H, *d*, *J* = 7.8 Hz, N-H), 8.18 (1H, *dd*, *J* = 7.9 and 1.6 Hz, H-8), 8.13 (1H, *d*, *J* = 9.5 Hz, H-1), 7.85 (1H, *ddd*, *J* = 8.5, 7.1 and 1.6 Hz, H-6), 7.63 (1H, *dd*, *J* = 8.0 and *J* = 1.1 Hz, H-5), 7.47 (1H, *ddd*, *J* = 8.1, 7.1 and 0.9 Hz, H-7), 7.12 (1H, *dd*, *J* = 9.5 and 2.4 Hz, H-2), 7.10 (1H, *d*, *J* = 2.4 Hz, H-4), 4.82 (1H, *d*, *J* = 15.0 Hz, -OCH_2_), 4.77 (1H, *d*, *J* = 15.0 Hz, -OCH_2_), 4.39 (1H, *m*, H-2′), 3.63 (3H, *s*, OCH_3_), 3.54 (3H, *s*, H-6′), 2.36 (2H, *t*, *J* = 7.5 Hz, H-4′), 2.07 (1H, *m*, H-4′), 1.91 (1H, *m*, H-3′) ppm. ^13^C NMR (100.6 MHz, DMSO-*d*_6_): *δ* = 174.9 (C-9), 172.6 (C-1′), 171.7 (C-5′), 167.3 (C=O amide), 163.3 (C-3), 157.3 (C-4ª), 155.6 (C-10ª), 135.1 (C-6), 127.6 (C-1), 125.9 (C-8), 124.4 (C-7), 121.2 (C-8ª), 117.9 (C-5), 115.4 (C-9ª), 114.1 (C-2), 101.6 (C-4), 67.0 (-OCH_2_), 52.0 (OCH_3_), 51.3 (C-2′), 50.9 (C-6′), 30.1 (C-4′), 26.1 (C-3′) ppm.

#### 3.1.24. Dimethyl (2-((9-Oxo-9H-xanthen-3-yl)oxy)acetyl)-D-glutamate (**25**)

Compound **25** was first synthesized using COMU. The obtained product was a yellow oil due to the presence of many impurities, according to the TLC control. This product was then hydrolyzed to obtain compound **55**. The coupling reaction with TBTU finished in 30 h. The extraction afforded compound **25** as a yellow solid (49.23 mg, 66.7%), m.p.: 98–101 °C IR (KBr): ṽ 3275, 1754, 1732, 1664, 1620, 1561, 1463, 1441, 1264, 845, 759, 668 cm^−1^. ^1^H NMR (400.14 MHz, DMSO-*d*_6_): *δ* = 8.64 (1H, *d*, *J* = 7.8 Hz, N-H), 8.18 (1H, *dd*, *J* = 7.9 and 1.6 Hz, H-8), 8.13 (1H, *d*, *J* = 9.5 Hz, H-1), 7.85 (1H, *ddd*, *J* = 8.5, 7.1 and 1.6 Hz, H-6), 7.63 (1H, *dd*, *J* = 8.0 and *J* = 1.1 Hz, H-5), 7.47 (1H, *ddd*, *J* = 7.9, 7.1 and 0.9 Hz, H-7), 7.12 (1H, *dd*, *J* =9.5 and 2.4 Hz, H-2), 7.10 (1H, *d*, *J* = 2.4 Hz, H-4), 4.82 (1H, *d*, *J* = 15.0 Hz, -OCH_2_), 4.77 (1H, *d*, *J* = 15.0 Hz, -OCH_2_), 4.39 (1H, *m*, H-2′), 3.63 (3H, *s*, OCH_3_), 3.54 (3H, *s*, H-6′), 2.36 (2H, *t*, *J* = 7.5 Hz, H-4′), 2.07 (1H, *m*, H-4′), 1.91 (1H, *m*, H-3′) ppm. ^13^C NMR (100.6 MHz, DMSO-*d*_6_): *δ* = 174.9 (C-9), 172.6 (C-1′), 171.7 (C-5′), 167.3 (C=O amide), 163.3 (C-3), 157.3 (C-4ª), 155.6 (C-10ª), 135.1 (C-6), 127.6 (C-1), 125.9 (C-8), 124.4 (C-7), 121.2 (C-8ª), 117.9 (C-5), 115.4 (C-9ª), 114.1 (C-2), 101.6 (C-4), 67.0 (-OCH_2_), 52.0 (OCH_3_), 51.3 (C-2′), 50.9 (C-6′), 30.1 (C-4′), 26.1 (C-3′) ppm.

#### 3.1.25. Methyl (2-((9-Oxo-9H-xanthen-3-yl)oxy)acetyl)-L-threoninate (**26**)

The coupling reaction finished in 23 h. The extraction afforded compound **26** as a yellow solid (60.4 mg, 44.1%). m.p.: 125–127 °C; IR (KBr): ṽ 3550–3300, 3447, 1749, 1653, 1617, 1540, 1465, 1445, 1231, 852, 756, 668 cm^−1^. ^1^H NMR (400.14 MHz, DMSO-*d*_6_): *δ* = 8.22 (1H, *d*, *J* = 8.6 Hz, N-H), 8.18 (1H, *dd*, *J* = 8.0 and 1.6 Hz, H-8), 8.13 (1H, *d*, *J* = 8.8 Hz, H-1), 7.85 (1H, *ddd*, *J* = 8.5, 7.1 and 1.6 Hz, H-6), 7.63 (1H, *dd*, *J* = 8.4 and *J* = 1.0 Hz, H-5), 7.48 (1H, *ddd*, *J* = 8.1, J= 7.1 and *J* = 1.0 Hz, H-7), 7.15 (1H, *d*, *J* = 2.4 Hz, H-4), 7.12 (1H, *dd*, *J* = 8.8 and 2.4 Hz, H-2), 5.17 (1H, *d*, *J* = 5.5 Hz, OH ), 4.89 (2H, *s*, OCH_2_), 4.37 (1H, *dd*, *J* = 8.6 and 3.2 Hz, H-2′), 4.18 (1H, *m*, H-3′), 3.65 (3H, *s*, OCH_3_), 1.09 (3H, *d*, *J* = 6.4 Hz, H-4′) ppm. ^13^C NMR (100.6 MHz, DMSO-*d*_6_): *δ* = 174.9 (C-9), 170.8 (C-1′), 167.8 (C=O amide), 163.3 (C-3), 157.3 (C-4a), 155.6 (C-10a), 135.2 (C-6), 127.7 (C-1), 125.9 (C-8), 124.4 (C-7), 121.2 (C-8a), 117.9 (C-5), 115.4 (C-9a), 113.9 (C-2), 101.6 (C-4), 66.9 (OCH_2_), 57.6 (C-2′), 51.9 (-OCH_3_), 46.9 (C-3′), 20.2 (C-4′) ppm.

#### 3.1.26. Methyl (2-((9-Oxo-9H-xanthen-3-yl)oxy)acetyl)-D-threoninate (**27**)

The coupling reaction finished in 24 h. The extraction afforded compound **27** as a yellow solid (68.6 mg, 47.5%), IR (KBr): ṽ 3550–3300, 3397, 1749, 1662, 1621, 1542, 1465, 1444, 1231, 854, 833, 757, 668 cm^−1^. ^1^H NMR (400.14 MHz, DMSO-*d*_6_): *δ* = 8.22 (1H, *d*, *J* = 8.6 Hz, N-H), 8.18 (1H, *dd*, *J* = 8.0 and 1.6 Hz, H-8), 8.13 (1H, *d*, *J* = 8.8 Hz, H-1), 7.85 (1H, *ddd*, *J* = 8.5, 7.1 and 1.6 Hz, H-6), 7.63 (1H, *dd*, *J* = 8.4 and *J* = 1.0 Hz, H-5), 7.48 (1H, *ddd*, *J* = 8.1, J= 7.1 and *J* = 1.0 Hz, H-7), 7.15 (1H, *d*, *J* = 2.4 Hz, H-4), 7.12 (1H, *dd*, *J* = 8.8 and 2.4 Hz, H-2), 5.17 (1H, *d*, *J* = 5.5 Hz, OH ), 4.89 (2H, *s*, OCH_2_), 4.37 (1H, *dd*, *J* = 8.6 and 3.2 Hz, H-2′), 4.18 (1H, *m*, H-3′), 3.65 (3H, *s*, OCH_3_), 1.09 (3H, *d*, *J* = 6.4 Hz, H-4′) ppm. ^13^C NMR (100.6 MHz, DMSO-*d*_6_): *δ* = 174.9 (C-9), 170.8 (C-1′), 167.8 (C=O amide), 163.3 (C-3), 157.3 (C-4a), 155.6 (C-10a), 135.2 (C-6), 127.7 (C-1), 125.9 (C-8), 124.4 (C-7), 121.2 (C-8a), 117.9 (C-5), 115.4 (C-9a), 113.9 (C-2), 101.6 (C-4), 66.9 (OCH_2_), 57.6 (C-2′), 51.9 (-OCH_3_), 46.9 (C-3′), 20.2 (C-4′) ppm.

#### 3.1.27. Methyl (2-((9-Oxo-9H-xanthen-3-yl)oxy)acetyl)-L-cysteinate (**28**)

The coupling reaction finished in 19 h. The extraction afforded compound **28** as a yellow solid (133.5 mg, 91.6%); m.p.: 129–132 °C; IR (KBr): ṽ 3292, 1743, 1668, 1621, 1540, 1497, 1489, 1464, 1442, 1255, 848, 757, 668 cm^−1^. ^1^H NMR (400.14 MHz, DMSO-*d*_6_): *δ* = 8.64 (1H, *d*, *J* = 7.8 Hz, N-H), 8.18 (1H, *d*, *J* = 7.9 and 1.6 Hz, H-8), 8.13 (1H, *d*, *J* = 9.5 Hz, H-1), 7.85 (1H, *ddd*, *J* = 8.6, 7.0 and 1.6 Hz, H-6), 7.64 (1H, *dd*, *J* = 8.2 and *J* = 1.0 Hz, H-5), 7.47 (1H, *ddd*, J = 8.1, *J*= 7.1 and *J* = 1.1, H-7), 7.11 (1H, *dd*, *J* = 9.5 and 2.4 Hz, H-2), 7.12 (1H, *d*, *J* = 2.4 Hz, H-4), 4.83 (2H, *s*, OCH_2_ ), 4.56 (1H, *m*, H-2′), 3.65 (3H, *s*, OCH_3_), 2.89 (2H, *s*, H-3′), 2.60 (1H, *t*, *J* = 8.6 Hz, -SH) ppm. ^13^C NMR (100.6 MHz, DMSO-*d*_6_): *δ* = 174.9 (C-9), 170.4 (C-1′), 167.2 (C=O amide), 163.3 (C-3), 157.3 (C-4ª), 155.6 (C-10ª), 135.1 (C-6), 127.6 (C-1), 125.9 (C-8), 124.4 (C-7), 121.2 (C-8ª), 117.9 (C-5), 115.4 (C-9ª), 114.0 (C-2), 101.6 (C-4), 66.9 (OCH_2_), 54.3 (C-2′), 52.2 (-OCH_3_), 37.9 (C-3′) ppm.

#### 3.1.28. Methyl (2-((9-Oxo-9H-xanthen-3-yl)oxy)acetyl)-D-cysteinate (**29**)

The coupling reaction finished in 19 h. The extraction afforded compound **29** as a yellow solid (130.8 mg, 90.6%). m.p.: 129–132 °C; IR (KBr): ṽ 3423, 1742, 1662, 1621, 1540, 1464, 1442, 1257, 833, 756, 668 cm^−1^. ^1^H NMR (400.14 MHz, DMSO-*d*_6_): *δ* = 8.64 (1H, *d*, *J* = 7.8 Hz, N-H), 8.18 (1H, *d*, *J* = 7.9 and 1.6 Hz, H-8), 8.13 (1H, *d*, *J* = 9.5 Hz, H-1), 7.85 (1H, *ddd*, *J* = 8.6, 7.0 and 1.6 Hz, H-6), 7.64 (1H, *dd*, *J* = 8.2 and *J* = 1.0 Hz, H-5), 7.47 (1H, *ddd*, J = 8.1, *J*= 7.1 and *J* = 1., H-7), 7.11 (1H, *dd*, *J* = 9.5 and 2.4 Hz, H-2), 7.12 (1H, *d*, *J* = 2.4 Hz, H-4), 4.83 (2H, *s*, OCH_2_ ), 4.56 (1H, *m*, H-2′), 3.65 (3H, *s*, OCH_3_), 2.89 (2H, *s*, H-3′), 2.60 (1H, *t*, *J* = 8.6 Hz, -SH) ppm. ^13^C NMR (100.6 MHz, DMSO-*d*_6_): *δ* = 174.9 (C-9), 170.4 (C-1′), 167.2 (C=O amide), 163.3 (C-3), 157.3 (C-4ª), 155.6 (C-10ª), 135.1 (C-6), 127.6 (C-1), 125.9 (C-8), 124.4 (C-7), 121.2 (C-8ª), 117.9 (C-5), 115.4 (C-9ª), 114.0 (C-2), 101.6 (C-4), 66.9 (OCH_2_), 54.3 (C-2′), 52.2 (-OCH_3_), 37.9 (C-3′) ppm.

#### 3.1.29. Methyl (2-((9-Oxo-9H-xanthen-3-yl)oxy)acetyl)-L-methioninate (**30**)

The coupling reaction finished in 25 h. After extraction, the product was recrystallized with dichloromethane and *n*-hexane to afford compound **30** as a white crystal solid (78.9 mg, 50.1%); m.p.: 116–117 °C; IR (KBr): ṽ 3302, 2949, 2920, 1735, 1651, 1619, 1540, 1507, 1466, 1434, 1256, 866, 832, 758, 703, 669 cm^−1^. ^1^H NMR (400.14 MHz, DMSO-*d*_6_): *δ* = 8.66 (1H, *d*, *J* = 7.8 Hz, N-H), 8.18 (1H, *dd*, *J* = 7.9 and 1.7 Hz, H-8), 8.13 (1H, *d*, *J* = 9.4 Hz, H-1), 7.85 (1H, *ddd*, *J* = 8.6, 7.0 and 1.7 Hz, H-6), 7.64 (1H, *dd*, *J* = 8.6 and 1.0 Hz, H-5), 7.48 (1H, *ddd*, *J* = 7.9, 7.1 and 1.0 Hz, H-7); 7.12 (1H, *dd*, *J* = 9.4 and 2.4 Hz, H-2), 7.10 (1H, *d*, *J* = 2.4 Hz, H-4), 4.83 (1H, *d*, *J* = 14.9 Hz, OCH_2_), 4.77 (1H, *d*, *J* = 14.9 Hz, OCH_2_), 4.49 (1H, *m*, H-2′), 3.65 (3H, *s*, OCH_3_), 2.47 (2H, *m*, H-4′), 2.01 (3H, *s*, H-5′), 1.99 (2H, *m*, H-3′) ppm. ^13^C NMR (100.6 MHz, DMSO-*d*_6_): *δ* = 174.9 (C-9), 171.9 (C-1′), 167.3 (C=O amide), 163.3 (C-3), 157.2 (C-4ª), 155.6 (C-10ª), 135.1 (C-6), 127.6 (C-1), 125.9 (C-8), 124.4 (C-7), 121.2 (C-8ª), 117.9 (C-5), 115.4 (C-9ª), 114.1 (C-2), 101.5 (C-4), 67.0 (OCH_2_), 52.0 (-OCH_3_), 50.7 (C-2′), 30.1 (C-4′), 29.5 (C-5′), 14.5 (C-3′) ppm.

#### 3.1.30. Methyl (2-((9-Oxo-9H-xanthen-3-yl)oxy)acetyl)-D-methioninate (**31**)

The coupling reaction finished in 25 h. After extraction, the product was recrystallized with dichloromethane and *n*-hexane to afford compound **31** as a white crystal solid (83.8 mg, 54.7%). m.p.: 111–113 °C; IR (KBr): ṽ 3306, 2951, 2920, 1736, 1650, 1620, 1541, 1466, 1435, 1256, 866, 833, 759, 703, 670 cm^−1^. ^1^H NMR (400.14 MHz, DMSO-*d*_6_): *δ* = 8.66 (1H, *d*, *J* = 7.8 Hz, N-H), 8.18 (1H, *dd*, *J* = 7.9 and 1.7 Hz, H-8), 8.13 (1H, *d*, *J* = 9.4 Hz, H-1), 7.85 (1H, *ddd*, *J* = 8.6, 7.0 and 1.6 Hz, H-6), 7.64 (1H, *dd*, *J* = 8.4 and 0.6 Hz, H-5), 7.48 (1H, *ddd*, *J* = 7.9, 7.1 and 0.9 Hz, H-7); 7.12 (1H, *dd*, *J* = 9.4 and 2.4 Hz, H-2), 7.10 (1H, *d*, *J* = 2.4 Hz, H-4), 4.83 (1H, *d*, *J* = 14.9 Hz, OCH_2_), 4.77 (1H, *d*, *J* = 14.9 Hz, OCH_2_), 4.49 (1H, *m*, H-2′), 3.65 (3H, *s*, OCH_3_), 2.47 (2H, *m*, H-4′), 2.01 (3H, *m*, H-5′), 1.99 (2H, s, H-3′) ppm. ^13^C NMR (100.6 MHz, DMSO-*d*_6_): *δ* = 174.9 (C-9), 171.9 (C-1′), 167.3 (C=O amide), 163.3 (C-3), 157.2 (C-4ª), 155.6 (C-10ª), 135.1 (C-6), 127.6 (C-1), 125.9 (C-8), 124.4 (C-7), 121.2 (C-8ª), 117.9 (C-5), 115.4 (C-9ª), 114.1 (C-2), 101.5 (C-4), 67.0 (OCH_2_), 52.0 (-OCH_3_), 50.7 (C-2′), 30.1 (C-4′), 29.5 (C-5′), 14.5 (C-3′) ppm.

### 3.2. General Procedure for the Synthesis of Chiral Derivatives of Xanthones of Amino Acids

The synthesized CDXs of amino ester (50 mg) were hydrolyzed in methanol (10 mL) with NaOH 0.25 M (3.75 mL) at room temperature. After finishing the reaction, the solvent was evaporated, 10 mL of water was added, and it was acidified with concentrated HCl. The solid was collected by filtration under reduced pressure, washed with cold water, and recrystallized to afford the CDX of amino acid (**32**–**61**). The chromatographic control was performed by TLC, using silica gel and a mixture of chloroform:methanol:formic acid (9:1:0.01, *v*/*v*/*v*).

The HRMS (ESI) data of the analyzed CDXs are in Table S4 (SM).

#### 3.2.1. (2-((9-.Oxo-9H-Xanthen-3-yl)oxy)acetyl)-L-alanine (**32**)

This reaction required an additional mL of NaOH (1 M) to initiate due to the presence of the *tert*-butyl group in the ester. The hydrolysis finished in 20 h and the product was crystallized with ethanol containing a few drops of THF and water to afford compound **32** as a white crystal solid (37.98 mg, 70.36%). m.p.: 215–216 °C; IR (KBr): ṽ 3100–2900, 3395, 1739, 1647, 1619, 1553, 1465, 1449, 1423, 1257, 865, 838, 761, 671 cm^−1^. ^1^H NMR (400.14 MHz, DMSO-*d*_6_): *δ* = 8.51 (1H, *d*, *J* = 7.5 Hz, N-H), 8.17 (1H, *dd*, *J* = 7.9 and 1.6 Hz, H-8), 8.13 (1H, *d*, *J* = 8.7 Hz, H-1), 7.85 (1H, *ddd*, *J* = 8.5, 7.0 and 1.6 Hz, H-6), 7.63 (1H, *dd*, *J* = 8.4 and 1.0 Hz, H-5), 7.47 (1H, *ddd*, 8.1; and 1.1 Hz, H-7); 7.12 (1H, *dd*, *J* = 8.5 and 2.4 Hz, H-2), 7.11 (1H, *d, J* = 2.4 Hz, H-4), 4.77 (1H, *d*, *J* = 14.8 Hz, -OCH_2_), 4.73 (1H, *d*, *J* = 14.8 Hz, -OCH_2_), 4.32 (1H, *m*, H-2′), 1.34 (1H, *d*, *J* = 7.3 Hz, H-3′) ppm. ^13^C NMR (100.6 MHz, DMSO-*d*_6_): *δ* = 174.9 (C-9), 173.7 (C-1′), 166.7 (C=O amide), 163.3 (C-3), 157.3 (C-4a), 155.6 (C-10a), 135.1 (C-6), 127.6 (C-1), 125.9 (C-8), 124.4 (C-7), 121.2 (C-8a), 117.9 (C-5), 115.3 (C-9a), 114.0 (C-2), 101.6 (C-4), 67.0 (-OCH_2_), 47.4 (C-2′), 17.0 (C-3′) ppm.

#### 3.2.2. (2-((9-.Oxo-9H-Xanthen-3-yl)oxy)acetyl)-D-alanine (**33**)

This reaction required an additional mL of NaOH (1 M) to initiate due to the presence of the *tert*-butyl group in the ester. The hydrolysis finished in 20 h and the product was crystallized with methanol containing a few drops of THF and water to afford compound **33** as a white crystal solid (38.08 mg, 68.71%). m.p.: 210–212 °C; IR (KBr): ṽ 3100–2900, 3395, 1739, 1648, 1619, 1553, 1465, 1449, 1423, 1257, 865, 838, 761, 671 cm^−1^. ^1^H NMR (400.14 MHz, DMSO-*d*_6_): *δ* = 8.51 (1H, *d*, *J* = 7.5 Hz, N-H), 8.17 (1H, *dd*, *J* = 7.9 and 1.6 Hz, H-8), 8.13 (1H, *d*, *J* = 8.7 Hz, H-1), 7.85 (1H, *ddd*, *J* = 8.5, 7.0 and 1.6 Hz, H-6), 7.63 (1H, *dd*, *J* = 8.4 and 1.0 Hz, H-5), 7.47 (1H, *ddd*, 8.1; and 1.1 Hz, H-7); 7.12 (1H, *dd*, *J* = 8.5 and 2.4 Hz, H-2), 7.11 (1H, *d*, *J* = 2.4 Hz, H-4), 4.77 (1H, *d*, *J* = 14.8 Hz, -OCH_2_), 4.73 (1H, *d*, *J* = 14.8 Hz, -OCH_2_), 4.32 (1H, *m*, H-2′), 1.34 (1H, *d*, *J* = 7.3 Hz, H-3′) ppm. ^13^C NMR (100.6 MHz, DMSO-*d*_6_): *δ* = 174.9 (C-9), 173.7 (C-1′), 166.7 (C=O amide), 163.3 (C-3), 157.3 (C-4a), 155.6 (C-10a), 135.1 (C-6), 127.6 (C-1), 125.9 (C-8), 124.4 (C-7), 121.2 (C-8a), 117.9 (C-5), 115.3 (C-9a), 114.0 (C-2), 101.6 (C-4), 67.0 (-OCH_2_), 47.4 (C-2′), 17.0 (C-3′) ppm.

#### 3.2.3. (2-((9-.Oxo-9H-Xanthen-3-yl)oxy)acetyl)glycine (**34**)

The hydrolysis finished in 10 min to afford compound **34** as a white solid (44.54 mg, ~99%). m.p.: 200–202 °C; IR (KBr): ṽ 3100–2900, 3063, 1741, 1649, 1620, 1559, 1466, 1422, 1259, 868, 837, 763, 671 cm^−1^. ^1^H NMR (300.13 MHz, DMSO-*d*_6_): *δ* = 8.55 (1H, *t*, *J* = 5.9 Hz, N-H), 8.16 (1H, *dd*, *J* = 8.0 and 1.6 Hz, H-8), 8.12 (1H, *d*, *J* = 8.8 Hz, H-1), 7.85 (1H, *ddd*, *J* = 8.6, 7.0 and 1.6 Hz, H-6), 7.62 (1H, *dd*, *J* = 8.4 and 1.0 Hz, H-5), 7.46 (1H, *ddd*, *J* = 8.1, 7.1 and 1.1 Hz, H-7), 7.12 (1H, *dd*, *J* = 8.7 and 2.4 Hz, H-2), 7.11 (1H, *d*, *J* = 2.4 Hz, H-4), 4.76 (2H, *s*, -OCH_2_), 3.84 (2H, *d*, *J* = 5.9 Hz, C-2′) ppm. ^13^C NMR (75.5 MHz, DMSO-*d*_6_): *δ* = 175.0 (C-9), 171.0 (C-1′), 167.4 (C=O amide), 163.3 (C-3), 157.4 (C-4a), 155.7 (C-10a), 135.2 (C-6), 127.7 (C-1), 126.0 (C-8), 124.5 (C-7), 121.2 (C-8a), 118.0 (C-5), 115.5 (C-9a), 114.2 (C-2), 101.7 (C-4), 67.2 (OCH_2_), 40.6 (C-2′) ppm.

#### 3.2.4. (2-((9-.Oxo-9H-Xanthen-3-yl)oxy)acetyl)-L-isoleucine (**35**)

The hydrolysis finished in 2 h to afford compound **35** as a white crystal solid (28.72 mg, 41.3%). m.p.: 140–142 °C; IR (KBr): ṽ 3100–2900, 3355, 1729, 1662, 1621, 1545, 1464, 1438, 1256, 835, 759, 668 cm^−1^. ^1^H NMR (400.14 MHz, DMSO-*d*_6_): *δ* = 8.33 (1H, *d*, *J* = 8.5 Hz, N-H), 8.17 (1H, *dd*, *J* = 7.9 and 1.6 Hz, H-8), 8.12 (1H, *d*, *J* = 8.3 Hz, H-1), 7.85 (1H, *ddd*, *J* = 8.7, 7.1 and 1.8 Hz, H-6), 7.62 (1H, *dd*, *J* = 8.4 and 1.0 Hz, H-5), 7.47 (1H, *J* = 8.1, 7.1 and 1.1H-7), 7.10 (1H, *dd*, *J* = 8.3 and 2.4 Hz, H-2), 7.08 (1H, *d*, *J* =2.4 Hz, H-4), 4.85 (1H, *d*, *J* = 14.7 Hz, OCH_2_), 4.80 (1H, *d*, *J* = 14.7 Hz, OCH_2_), 4.25 (1H, *dd*, *J* = 8.4 and 6.0 Hz, H-2′), 1.85 (1H, *m*, H-3′), 1.44 (1H, *m*, H-4′), 1.20 (1H, *m*, H-4′), 0.88 (3H, *d*, *J* = 6.9 Hz, H-6′), 0.85 (1H, *t*, *J* = 7.4 Hz, H-5′) ppm. ^13^C NMR (100.6 MHz, DMSO-*d*_6_): *δ* = 174.9 (C-9), 172.6 (C-1′), 166.9 (C=O amide), 163.5 (C-3), 157.3 (C-4a), 155.6 (C-10a), 135.1 (C-6), 127.6 (C-1), 125.9 (C-8), 124.4 (C-7), 121.2 (C-8a), 117.9 (C-5), 115.3 (C-9a), 113.9 (C-2), 101.5 (C-4), 66.9 (-OCH_2_), 56.1 (C-2′), 36.3 (C-3′), 24.7 (C-4′), 15.5 (C-6′), 11.2 (C-5′) ppm.

#### 3.2.5. (2-((9-.Oxo-9H-Xanthen-3-yl)oxy)acetyl)-L-leucine (**36**)

The hydrolysis finished in 1 h to afford compound **36** as a white solid (37.12 mg, 67.74%). m.p.: 195–197 °C; IR (KBr): ṽ 3302, 3000–2800 1732, 1660, 1611, 1563, 1466, 1446, 1244, 843, 751, 670 cm^−1^. ^1^H NMR (400.14 MHz, DMSO-*d*_6_): *δ* = 8.48 (1H, *d*, *J* = 8.2 Hz, N-H), 8.18 (1H, *dd*, *J* = 7.9 and 1.6 Hz, H-8), 8.12 (1H, *d*, *J* = 8.3 Hz, H-1), 7.85 (1H, *ddd*, *J* = 8.7, 7.0 and 1.8 Hz, H-6), 7.62 (1H, *dd*, *J* = 8.4 and 1.0 Hz, H-5), 7.47 (1H, *ddd*, *J* = 7.9, 7.1 and 0.9 Hz, H-7); 7.12 (1H, *dd*, *J* = 8.3 and 2.5 Hz, H-2), 7.10 (1H, *d*, *J*= 2.5 Hz, H-4), 4.80 (1H, *d*, *J* = 14.8 Hz, OCH_2_ ), 4.75 (1H, *d*, *J* = 14.8 Hz, OCH_2_ ), 4.31 (1H, *m*, H-2′), 1.60 (3H, *m*, H-3′ and H-4′), 0.88 (3H, *d*, *J* = 6.2 Hz, H-5′), 0.83 (3H, *d*, *J* = 6.2 Hz, H-6′) ppm. ^13^C NMR (100.6 MHz, DMSO-*d*_6_): *δ* = 174.9 (C-9), 173.7 (C-1′), 167.0 (C=O amide), 163.4 (C-3), 157.3 (C-4a), 155.6 (C-10a), 135.1 (C-6), 127.5 (C-1), 125.9 (C-8), 124.4 (C-7), 121.2 (C-8a), 117.9 (C-5), 115.3 (C-9a), 114.1 (C-2), 101.5 (C-4), 67.0 (-OCH_2_), 50.0 (C-2′), 24.3 (C-3′), 22.9 (C-4′), 21.1 (C-5′ and C-6′) ppm.

#### 3.2.6. (2-((9-.Oxo-9H-Xanthen-3-yl)oxy)acetyl)-D-leucine (**37**)

The hydrolysis finished in 1 h to afford compound **37** as a white solid (35.51 mg, 63.5%). m.p.: 196–197 °C; IR (KBr): ṽ 3380, 3304, 1731, 1662, 1621, 1551, 1465, 1438, 1257, 835, 756, 669 cm^−1^. ^1^H NMR (400.14 MHz, DMSO-*d*_6_): *δ* = 8.48 (1H, *d*, *J* = 8.2 Hz, N-H), 8.18 (1H, *dd*, *J* = 7.9 and 1.6 Hz, H-8), 8.12 (1H, *d*, *J* = 9.5 Hz, H-1), 7.85 (1H, *ddd*, *J* = 8.6, 7.0 and 1.6 Hz, H-6), 7.62 (1H, *dd*, *J* = 8.4 and 1.0 Hz, H-5), 7.47 (1H, *ddd*, *J* = 7.9, 7.1 and 0.9 Hz, H-7); 7.12 (1H, *dd*, *J* = 9.5 and 2.5 Hz, H-2), 7.10 (1H, *d*, *J*= 2.5 Hz, H-4), 4.80 (1H, *d*, *J* = 14.8 Hz, OCH_2_ ), 4.75 (1H, *d*, *J* = 14.8 Hz, OCH_2_ ), 4.31 (1H, *m*, H-2′), 1.60 (3H, *m*, H-3′ and H-4′), 0.88 (3H, *d*, *J* = 6.2 Hz, H-5′), 0.83 (3H, *d*, *J* = 6.2 Hz, H-6′) ppm. ^13^C NMR (100.6 MHz, DMSO-*d*_6_): *δ* = 174.9 (C-9), 173.7 (C-1′), 167.0 (C=O amide), 163.4 (C-3), 157.3 (C-4a), 155.6 (C-10a), 135.1 (C-6), 127.5 (C-1), 125.9 (C-8), 124.4 (C-7), 121.2 (C-8a), 117.9 (C-5), 115.3 (C-9a), 114.1 (C-2), 101.5 (C-4), 67.0 (-OCH_2_), 50.0 (C-2′), 24.3 (C-3′), 22.9 (C-4′), 21.1 (C-5′ and C-6′) ppm.

#### 3.2.7. (2-((9-.Oxo-9H-Xanthen-3-yl)oxy)acetyl)-L-proline (**38**)

The hydrolysis finished in 1 h to afford compound **38** as a white solid (56.6 mg, 49.97%). m.p.: 104–106 °C; IR (KBr): ṽ 3400–3000, 1719, 1659, 1615, 1584, 1466, 1443, 1254, 833, 765, 669 cm^−1^. ^1^H NMR (400.14 MHz, DMSO-*d*_6_): *δ* = 8.17 (1H, *dd*, *J* = 7.9 and 1.6 Hz, H-8), 8.09 (1H, *d*, *J* = 8.8 Hz, H-1), 7.85 (1H, *ddd*, *J* = 8.8, 7.1 and 1.7 Hz, H-6), 7.62 (1H, *dd*, *J* = 8.4 and 1.1 Hz, H-5), 7.47 (1H, *ddd*, *J* = 8.1, 71 and 1,1, H-7); 7.11 (1H, *d*, *J* = 2.4 Hz, H-4), 7.07 (1H, *dd*, *J* = 8.9 and 2.4 Hz, H-2), 5.04 (2H, *s*, OCH_2_), 4.29 (1H, *dd*, *J* = 8.7 and 4.2, H-2′), 3.63 (2H, *m*, H-5′), 2.18 (1H, *m*, H-3′), 1.97 (2H, *m*, H-4′) and 1.88 (1H, *m*, H-3′) ppm. ^13^C NMR (100.6 MHz, DMSO-*d*_6_): *δ* = 174.9 (C-9), 173.1 (C-1′), 165.1 (C=O amide), 163.8 (C-3), 157.3 (C-4a), 155.6 (C-10a), 135.1 (C-6), 127.4 (C-1), 125.9 (C-8), 124.3 (C-7), 121.2 (C-8a), 117.9 (C-5), 115.1 (C-9a), 114.0 (C-2), 101.6 (C-4), 66.2 (-OCH_2_), 58.7 (C-2′), 45.2 (C-5′), 28.5 (C-3′) and 24.4 (C-4′) ppm.

#### 3.2.8. (2-((9-.Oxo-9H-Xanthen-3-yl)oxy)acetyl)-D-proline (**39**)

The hydrolysis finished in 1 h to afford compound **39** as a white solid (64.77 mg, 47.21%). Compound **9** was obtained in the form of a yellow oil, which was hydrolyzed to achieve compound **39.** The calculated yield corresponds to the coupling reaction of XCAR (**1**) using COMU, followed by the hydrolysis. m.p.: 103–105 °C; IR (KBr): ṽ 3500–3000, 1718, 1659, 1615, 1584, 1466, 1443, 1254, 833, 765, 669 cm^−1^. ^1^H NMR (400.14 MHz, DMSO-*d*_6_): *δ* = 8.17 (1H, *dd*, *J* = 7.9 and 1.4 Hz, H-8), 8.09 (1H, *d*, *J* = 8.9 Hz, H-1), 7.85 (1H, *ddd*, *J* = 8.5, 7.0 and 1.6 Hz, H-6), 7.62 (1H, *d*, *J* = 7.9 Hz, H-5), 7.47 (1H, *ddd*, *J* = 8.1, 71 and 1,1 H-7); 7.11 (1H, *d*, *J* = 2.4 Hz, H-4), 7.07 (1H, *dd*, *J* = 8.9 and 2.4 Hz, H-2), 5.04 (2H, *s*, OCH_2_), 4.29 (1H, *dd*, *J* = 8.7 and 4.2, H-2′), 3.63 (2H, *m*, H-5′), 2.18 (1H, *m*, H-3′), 1.97 (2H, *m*, H-4′) and 1.88 (1H, *m*, H-3′) ppm. ^13^C NMR (100.6 MHz, DMSO-*d*_6_): *δ* = 174.9 (C-9), 173.1 (C-1′), 165.1 (C=O amide), 163.8 (C-3), 157.3 (C-4a), 155.6 (C-10a), 135.1 (C-6), 127.4 (C-1), 125.9 (C-8), 124.3 (C-7), 121.2 (C-8a), 117.9 (C-5), 115.1 (C-9a), 114.0 (C-2), 101.6 (C-4), 66.2 (-OCH_2_), 58.7 (C-2′), 45.2 (C-5′), 28.5 (C-3′) and 24.4 (C-4′) ppm.

#### 3.2.9. (2-((9-.Oxo-9H-Xanthen-3-yl)oxy)acetyl)-L-valine (**40**)

The hydrolysis finished in 24 h to afford compound **40** as a white solid (30.8 mg, 59.16%). m.p.: 103–105 °C; IR (KBr): ṽ 3404, 3300–2900, 1733, 1653, 1619, 1539, 1465, 1439, 1256, 836, 758, 668 cm^−1^. ^1^H NMR (400.14 MHz, DMSO-*d*_6_): *δ* = 8.31 (1H, *d*, *J* = 8.5 Hz, N-H), 8.16 (1H, *dd*, *J* = 7.9 and 1.5 Hz, H-8), 8.10 (1H, *d*, *J* = 8.6 Hz, H-1), 7.83 (1H, *J* = 8.7, 7.1 and 1.7, H-6), 7.60 (1H, *J* = 8.6 and 1.0, H-5), 7.46 (1H, *J* = 8.0, 7.1 and 1.0, H-7); 7.10 (1H, *dd*, *J* = 8.6 and 2.3 Hz, H-2), 7.08 (1H, *d*, *J* = 2.3 Hz, H-4), 4.87 (1H, *d*, *J* = 14.7 Hz, OCH_2_), 4.82 (1H, *d*, *J* = 14.7 Hz, OCH_2_), 4.22 (1H, *m*, H-2′), 2.11 (1H, m, H-3′), 0.9 (6H, *d*, *J* = 5.9 Hz, H-4′ and H-5′). ^13^C NMR (100.6 MHz, DMSO-*d*_6_): *δ* = 175.0 (C-9), 172.7 (C-1′), 167.1 (C=O amide), 163.5 (C-3), 157.3 (C-4a), 155.7 (C-10a), 135.2 (C-6), 127.6 (C-1), 125.9 (C-8), 124.4 (C-7), 121.2 (C-8a), 118.0 (C-5), 115.3 (C-9a), 113.9 (C-2), 101.6 (C-4), 66.9 (-OCH_2_), 57.1 (C-2′), 29.9 (C-3′), 19.1 (C-4′) and 18.0 (C-5′).

#### 3.2.10. (2-((9-.Oxo-9H-Xanthen-3-yl)oxy)acetyl)-D-valine (**41**)

The hydrolysis finished in 5 h and the product was crystallized with methanol and water to afford compound **41** as a white crystal solid (30.37 mg, 66.6%). IR (KBr): ṽ 3401, 3500–2900, 1718, 1659, 1624, 1539, 1498, 1467, 1423, 1258, 854, 838, 762, 741, 667 cm^−1^. ^1^H NMR (400.14 MHz, DMSO-*d*_6_): *δ* = 8.31 (1H, *d*, *J* = 8.5 Hz, N-H), 8.16 (1H, *dd*, *J* = 7.9 and 1.5 Hz, H-8), 8.10 (1H, *d*, *J* = 8.6 Hz, H-1), 7.83 (1H, *J* = 8.7, 7.1 and 1.7, H-6), 7.60 (1H, *J* = 8.6 and 1.*0* H-5), 7.46 (1H, *J* = 8.0, 7.1 and 1.0 H-7); 7.10 (1H, *dd*, *J* = 8.6 and 2.3 Hz, H-2), 7.08 (1H, *d*, *J* = 2.3 Hz, H-4), 4.87 (1H, *d*, *J* = 14.7 Hz, OCH_2_), 4.82 (1H, *d*, *J* = 14.7 Hz, OCH_2_), 4.22 (1H, *m*, H-2′), 2.11 (1H, m, H-3′), 0.9 (6H, *d*, *J* = 5.9 Hz, H-4′ and H-5′). ^13^C NMR (100.6 MHz, DMSO-*d*_6_): *δ* = 175.0 (C-9), 172.7 (C-1′), 167.1 (C=O amide), 163.5 (C-3), 157.3 (C-4a), 155.7 (C-10a), 135.2 (C-6), 127.6 (C-1), 125.9 (C-8), 124.4 (C-7), 121.2 (C-8a), 118.0 (C-5), 115.3 (C-9a), 113.9 (C-2), 101.6 (C-4), 66.9 (-OCH_2_), 57.1 (C-2′), 29.9 (C-3′), 19.1 (C-4′) and 18.0 (C-5′).

#### 3.2.11. (*S*)-2-(2-((9-Oxo-9H-Xanthen-3-yl)oxy)acetamido)-2-phenylacetic acid (**42**)

The hydrolysis finished in 2 h and the product was crystallized with methanol and water to afford compound **42** as a white solid (23.79 mg, 55.69%). m.p.: 218–220 °C; IR (KBr): ṽ 3300–2900, 3300, 1728, 1661, 1610, 1544, 1497, 1466, 1446, 1232, 854, 754, 722, 694, 669, 650 cm^−1^. ^1^H NMR (400.14 MHz, DMSO): *δ* = 8.94 (1H, *d*, *J* = 7.4 Hz, N-H), 8.17 (1H, *dd*, *J* = 7.9 and 1.6 Hz, H-8), 8.10 (1H, *d*, *J* = 9.2 Hz, H-1), 7.85 (1H, *ddd*, *J* = 8.6, 7.0 and 1.6 Hz, H-6), 7.63 (1H, *dd*, *J* = 8.4 and 1.0Hz, H-5), 7.47 (1H, *ddd*, *J* = 7.9, 7.0 and 1.0 Hz, H-7), 7.43 (2H, *m*, H-5′), 7.38 (2H, *m*, H-4′), 7.30 (1H, *m*, H-6′), 7.11 (1H, *dd*, *J* = 9.2 and 2.4 Hz, H-2), 7.10 (1H, *d*, *J* = 2.4 Hz, H-4), 5.41 (1H, *d*, *J* = 7.5 Hz, H-2′), 4.88 (1H, *d*, *J* = 14.8 Hz, OCH_2_) and 4.84 (1H, *d*, *J* = 14.8 Hz, OCH_2_) ppm. ^13^C NMR (100.6 MHz, DMSO): *δ* = 174.9 (C-9), 171.5 (C-1′), 166.7 (C=O amide), 163.5 (C-3), 157.3 (C-4a), 155.6 (C-10a), 136.9 (C-3′), 135.1 (C-6), 128.6 (C-4′), 128.1 (C-6′), 127.6 (C-1 and C-5′), 125.9 (C-8), 124.4 (C-7), 121.2 (C-8a), 117.9 (C-5), 115.3 (C-9a), 114.0 (C-2), 101.5 (C-4), 66.8 (-OCH_2_), 56.1 (C-2′) ppm.

#### 3.2.12. (R)-2-(2-((9-Oxo-9H-Xanthen-3-yl)oxy)acetamido)-2-phenylacetic acid (**43**)

The hydrolysis finished in 2 h and the product was crystallized with methanol and water to afford compound **43** as a white solid (28.95 mg, 60.71%). m.p.: 218–219 °C; IR (KBr): ṽ 3310, 3400–2900, 1717, 1662, 1615, 1543, 1497, 1466, 1446, 1232, 855, 755, 722, 695, 669, 651 cm^−1^. ^1^H NMR (400.14 MHz, DMSO): *δ* = 8.94 (1H, *d*, *J* = 7.4 Hz, N-H), 8.17 (1H, *dd*, *J* = 7.9 and 1.6 Hz, H-8), 8.10 (1H, *d*, *J* = 9.2 Hz, H-1), 7.85 (1H, *ddd*, *J* = 8.6, 7.0 and 1.6 Hz, H-6), 7.63 (1H, *dd*, *J* = 8.4 and 1.0 Hz, H-5), 7.47 (1H, *ddd*, *J* = 7.9, 7.0 and 1.0 Hz, H-7), 7.43 (2H, *m*, H-5′), 7.38 (2H, *m*, H-4′), 7.30 (1H, *m*, H-6′), 7.11 (1H, *dd*, *J* = 9.2 and 2.4 Hz, H-2), 7.10 (1H, *d*, *J* = 2.4 Hz, H-4), 5.41 (1H, *d*, *J* = 7.5 Hz, H-2′), 4.88 (1H, *d*, *J* = 14.8 Hz, OCH_2_) and 4.84 (1H, *d*, *J* = 14.8 Hz, OCH_2_) ppm. ^13^C NMR (100.6 MHz, DMSO): *δ* = 174.9 (C-9), 171.5 (C-1′), 166.7 (C=O amide), 163.5 (C-3), 157.3 (C-4a), 155.6 (C-10a), 136.9 (C-3′), 135.1 (C-6), 128.6 (C-4′), 128.1 (C-6′), 127.6 (C-1 and C-5′), 125.9 (C-8), 124.4 (C-7), 121.2 (C-8a), 117.9 (C-5), 115.3 (C-9a), 114.0 (C-2), 101.5 (C-4), 66.8 (-OCH_2_), 56.1 (C-2′) ppm.

#### 3.2.13. (2-((9-.Oxo-9H-Xanthen-3-yl)oxy)acetyl)-L-phenylalanine (**44**)

The hydrolysis finished in 1 h and the product was crystallized with ethyl acetate and methanol containing *n*-hexane to afford compound **44** as a white solid (30.78 mg, 62.26%). m.p.: 229–231 °C; IR (KBr): ṽ 3400–2900, 3395, 1736, 1660, 1619, 1556, 1497, 1465, 1436, 1257, 855, 839, 758, 739, 703 cm^−1^. ^1^H NMR (400.14 MHz, DMSO-*d*_6_): *δ* = 8.47 (1H, *d*, *J* = 8.2 Hz, N-H), 8.19 (1H, *dd*, *J* = 7.9 and 1.6 Hz, H-8), 8.10 (1H, *d*, *J* = 8.8 Hz, H-1), 7.86 (1H, *ddd*, *J* = 8.5, 7.0 and 1.6 Hz, H-6), 7.64 (1H, *dd*, *J* = 8.1 and 1.0 Hz, H-5), 7.48 (1H, *ddd*, *J* =7.9, 7.0 and 0.9 Hz, H-7); 7.21 (4H, *d*, *J* = 4.4, H-5′ and H-6′), 7.13 (1H, *m*, H-7′), 7.04 (1H, *dd*, *J* = 8.8 and 2.4 Hz, H-2), 7.00 (1H, *d*, *J* = 2.4 Hz, H-4), 4.72 (1H, *d*, *J* = 15.0 Hz, OCH_2_), 4.68 (1H, *d*, *J* = 15.0 Hz, OCH_2_), 4.53 (1H, *m*, H-2′), 3.13 (1H, *dd*, *J* = 13.8 and 4.6, H-3′), 2.97 (1H, *dd*, *J* = 13.8 and 9.7 Hz, H-3′) ppm. ^13^C NMR (100.6 MHz, DMSO-*d*_6_): *δ* = 174.9 (C-9), 172.6 (C-1′), 166.9 (C=O amide), 163.2 (C-3), 157.2 (C-4a), 155.6 (C-10a), 137.5 (C-4′), 135.1 (C-6), 129.0 (C-6′), 128.1 (C-5′), 127.5 (C-1), 126.3 (C-7′), 125.9 (C-8), 124.4 (C-7), 121.2 (C-8a), 117.9 (C-5), 115.4 (C-9a), 114.0 (C-2), 101.5 (C-4), 66.9 (-OCH_2_), 53.2 (C-2′), 36.3 (C-3′) ppm.

#### 3.2.14. (2-((9-.Oxo-9H-Xanthen-3-yl)oxy)acetyl)-D-phenylalanine (**45**)

The hydrolysis finished in 4 h and the product was crystallized with ethyl acetate and methanol containing *n*-hexane to afford compound **45** as a white solid (37.50 mg, 74.97%); m.p.: 234–236 °C; IR (KBr): ṽ 3500–2900, 3395, 1736, 1661, 1620, 1556, 1466, 1435, 1257, 856, 840, 757, 739, 703, 670 cm^−1^. ^1^H NMR (400.14 MHz, DMSO-*d*_6_): *δ* = 8.47 (1H, *d*, *J* = 8.2 Hz, N-H), 8.19 (1H, *dd*, *J* = 7.9 and 1.6 Hz, H-8), 8.10 (1H, *d*, *J* = 8.8 Hz, H-1), 7.86 (1H, *ddd*, *J* = 8.5, 7.0 and 1.6 Hz, H-6), 7.64 (1H, *dd*, *J* = 8.1 and 1.0, H-5), 7.48 (1H, *ddd*, *J* =7.9, 7.0 and 0.9 Hz, H-7); 7.21 (4H, *d*, *J* = 4.4, H-5′ and H-6′), 7.13 (1H, *m*, H-7′), 7.04 (1H, *dd*, *J* = 8.8 and 2.4 Hz, H-2), 7.00 (1H, *d*, *J* = 2.4 Hz, H-4), 4.72 (1H, *d*, *J* = 15.0 Hz, OCH_2_), 4.68 (1H, *d*, *J* = 15.0 Hz, OCH_2_), 4.53 (1H, *m*, H-2′), 3.13 (1H, *dd*, *J* = 13.8 and 4.6, H-3′), 2.97 (1H, *dd*, *J* = 13.8 and 9.7 Hz, H-3′) ppm. ^13^C NMR (100.6 MHz, DMSO-*d*_6_): *δ* = 174.9 (C-9), 172.6 (C-1′), 166.9 (C=O amide), 163.2 (C-3), 157.2 (C-4a), 155.6 (C-10a), 137.5 (C-4′), 135.1 (C-6), 129.0 (C-6′), 128.1 (C-5′), 127.5 (C-1), 126.3 (C-7′), 125.9 (C-8), 124.4 (C-7), 121.2 (C-8a), 117.9 (C-5), 115.4 (C-9a), 114.0 (C-2), 101.5 (C-4), 66.9 (-OCH_2_), 53.2 (C-2′), 36.3 (C-3′) ppm.

#### 3.2.15. (2-((9-.Oxo-9H-Xanthen-3-yl)oxy)acetyl)-L-tryptophan (**46**)

The hydrolysis finished in 2 h and the product was crystallized with methanol containing a few drops of THF and water to afford compound **46** as a white solid (32.91 mg, 70.82%). m.p.: 231–232 °C; IR (KBr): ṽ 3400–2900, 3306, 1690, 1644, 1618, 1586, 1477, 1466, 1437, 1329, 1258, 765, 742, 669 cm^−1^. ^1^H NMR (300.13 MHz, DMSO-*d*_6_): *δ* = 10.87 (1H, *d*, *J* = 2.5 Hz, N-H_arom_), 8.37 (1H, *d*, *J* = 7.9 Hz, N-H), 8.18 (1H, *dd*, *J* = 7.9 and 1.5 Hz, H-8), 8.09 (1H, *d*, *J* = 8.8 Hz, H-1), 7.85 (1H, *ddd*, *J* = 8.5, 7.0 and 1.6 Hz, H-6), 7.63 (1H, *dd*, *J* = 8.4 and 1.0 Hz, H-5), 7.55 (1H, the calculation of *dd* was not possible, *J* = 7.8 Hz, H-10′), 7.47 (1H, *ddd*, *J* = 8.1, 7.1 and 0.9 Hz, H-7), 7.31 (1H, dd*, *J* = 7.9 Hz, H-7′), 7.17 (1H, *d*, *J* = 2.3 Hz, H-5′), 7.06 (1H, dd*, *J* = 8.8 Hz, H-2), 7.03 (1H, *m*, H-8′), 7.02 (1H, d*, H-4), 6.95 (1H, *m*, H-9′), 4.70 (2H, *s*, OCH_2_), 4.55 (1H, *m*, H-2′), 3.26 (1H, *dd*, *J* = 15.1 and 5.1 Hz, H-3′), 3.14 (1H, *dd*, *J* = 14.6 and 8.3 Hz, H-3′) ppm. ^13^C NMR (75.5 MHz, DMSO-*d*_6_): *δ* = 175.0 (C-9), 172.8 (C-1′), 166.9 (C=O amide), 163.2 (C-3), 157.3 (C-4a), 155.7 (C-10a), 136.1 (C-6′), 135.2 (C-6), 127.6 (C-1), 127.2 (C-9′), 125.9 (C-8), 124.4 (C-7), 123.7 (C-5′), 121.2 (C-8a), 121.0 (C-8′), 118.4 (C-9′), 118.2 (C-10′), 118.0 (C-5), 115.4 (C-9a), 113.9 (C-2), 111.4 (C-7′), 109.7 (C-4′), 101.6 (C-4), 67.0 (-OCH_2_), 52.8 (C-2′), 26.8 (C-3′) ppm.

#### 3.2.16. (2-((9-.Oxo-9H-Xanthen-3-yl)oxy)acetyl)-D-tryptophan (**47**)

The hydrolysis finished in 1 h and the product was crystallized with methanol and water to afford compound **47** as a white solid (30.45 mg, 63.53%). m.p.: 233–234 °C; IR (KBr): ṽ 3500–2900, 3379, 1690, 1617, 1586, 1477, 1465, 1436, 1328, 1258, 764, 742, 670 cm^−1^. ^1^H NMR (300.13 MHz, DMSO-*d*_6_): *δ* = 10.87 (1H, *d*, *J* = 2.5 Hz, N-H_arom_), 8.37 (1H, *d*, *J* = 7.9 Hz, N-H), 8.18 (1H, *dd*, *J* = 7.9 and 1.5 Hz, H-8), 8.09 (1H, *d*, *J* = 8.8 Hz, H-1), 7.85 (1H, *ddd*, *J* = 8.5, 7.0 and 1.6 Hz, H-6), 7.63 (1H, *dd*, *J* = 8.4 and 1.0 Hz, H-5), 7.55 (1H, dd*, *J* = 7.8 Hz, H-10′), 7.47 (1H, *ddd*, *J* = 8.1, 7.1 and 0.9 Hz, H-7), 7.31 (1H, dd*, *J* = 7.9 Hz, H-7′), 7.17 (1H, *d*, *J* = 2.3 Hz, H-5′), 7.06 (1H, dd*, *J* = 8.8 Hz, H-2), 7.03 (1H, *m*, H-8′), 7.02 (1H, d*, H-4), 6.95 (1H, *m*, H-9′), 4.70 (2H, *s*, OCH_2_), 4.55 (1H, *m*, H-2′), 3.26 (1H, *dd*, *J* = 15.1 and 5.1 Hz, H-3′), 3.14 (1H, *dd*, *J* = 14.6 and 8.3 Hz, H-3′) ppm. *The calculation was not possible. ^13^C NMR (75.5 MHz, DMSO-*d*_6_): *δ* = 175.0 (C-9), 172.8 (C-1′), 166.9 (C=O amide), 163.2 (C-3), 157.3 (C-4a), 155.7 (C-10a), 136.1 (C-6′), 135.2 (C-6), 127.6 (C-1), 127.2 (C-9′), 125.9 (C-8), 124.4 (C-7), 123.7 (C-5′), 121.2 (C-8a), 121.0 (C-8′), 118.4 (C-9′), 118.2 (C-10′), 118.0 (C-5), 115.4 (C-9a), 113.9 (C-2), 111.4 (C-7′), 109.7 (C-4′), 101.6 (C-4), 67.0 (-OCH_2_), 52.8 (C-2′), 26.8 (C-3′) ppm.

#### 3.2.17. (2-((9-.Oxo-9H-Xanthen-3-yl)oxy)acetyl)-L-tyrosine (**48**)

The hydrolysis finished in 4 h to afford compound **48** as a white solid (29.06 mg, 65.08%). m.p.: 239–240 °C; IR (KBr): ṽ 3500–2000, 3439, 1733, 1671, 1608, 1563, 1465, 1444, 1231, 849, 826, 759, 698, 668 cm^−1^. ^1^H NMR (300.13 MHz, DMSO-*d*_6_): *δ* = 9.21 (1H, *s*, Arom-OH), 8.38 (1H, *d*, *J* = 8.1 Hz, N-H), 8.18 (1H, *dd*, *J* = 7.9 and 1.5 Hz, H-8), 8.11 (1H, *d*, *J* = 9.3 Hz, H-1), 7.86 (1H, *ddd*, *J* = 8.6, 7.0 and 1.6 Hz, H-6), 7.65 (1H, *dd*, *J* = 7.5 and 1.0 Hz, H-5), 7.47 (1H, 8.1, 7.1 and 1.1 H-7), 7.06 (1H, dd*, *J* = 9.3Hz, H-2), 7.04 (1H, d*, H-4), 7.01 (2H, *d*, *J* = 8.6 Hz, H-5′), 6.63 (2H, *d*, *J* = 8.5 Hz, H-6′), 4.70 (2H, *s*, -OCH_2_ ), 4.43 (1H, *m*, H-2′), 3.00 (1H, *dd*, *J* = 13.9 and 4.7 Hz, H-3′), 2.86 (1H, *dd*, *J* = 13.9 and 9.2 Hz, H-3′) ppm. * The calculation was not possible. ^13^C NMR (75.5 MHz, DMSO-*d*_6_): *δ* = 175.0 (C-9), 172.8 (C-1′), 166.8 (C=O amide), 163.3 (C-3), 157.3 (C-4a), 155.9 (C-7′), 155.7 (C-10a), 135.2 (C-6), 130.1 (C-5′), 127.6 (C-1), 127.5 (C-4′), 125.9 (C-8), 124.4 (C-7), 121.2 (C-8a), 118.0 (C-5), 115.4 (C-9a), 115.0 (C-6′), 114.0 (C-2), 101.6 (C-4), 66.9 (-OCH_2_), 53.6 (C-2′), 35.7 (C-3′) ppm.

#### 3.2.18. (2-((9-.Oxo-9H-Xanthen-3-yl)oxy)acetyl)-D-tyrosine (**49**)

The hydrolysis finished in 1 h to afford compound **49** as a white solid (28.97 mg, 69.17%). IR (KBr): ṽ 3500–2400, 3420, 1733, 1652, 1618, 1558, 1465, 1444, 1232, 836, 759, 698 cm^−1^. ^1^H NMR (300.13 MHz, DMSO-*d*_6_): *δ* = 9.22 (1H, *s*, Arom-OH), 8.38 (1H, *d*, *J* = 8.1 Hz, N-H), 8.18 (1H, *dd*, *J* = 7.9 and 1.5 Hz, H-8), 8.11 (1H, *d*, *J* = 9.3 Hz, H-1), 7.86 (1H, *ddd*, *J* = 8.6, 7.0 and 1.6 Hz, H-6), 7.65 (1H, *dd*, *J* = 7.5 and 1.0 Hz, H-5), 7.47 (1H, 8.1, 7.1 and 1, H-7), 7.06 (1H, dd*, *J* = 9.3Hz, H-2), 7.04 (1H, d*, H-4), 7.01 (2H, *d, J* = 8.6 Hz, H-5′), 6.63 (2H, *d*, *J* = 8.5 Hz, H-6′), 4.70 (2H, *s*, -OCH_2_ ), 4.43 (1H, *m*, H-2′), 3.00 (1H, *dd*, *J* = 13.9 and 4.7 Hz, H-3′), 2.86 (1H, *dd*, *J* = 13.9 and 9.2 Hz, H-3′) ppm. * The calculation was not possible. ^13^C NMR (75.5 MHz, DMSO-*d*_6_): *δ* = 175.0 (C-9), 172.8 (C-1′), 166.8 (C=O amide), 163.3 (C-3), 157.3 (C-4a), 155.9 (C-7′), 155.7 (C-10a), 135.2 (C-6), 130.1 (C-5′), 127.6 (C-1), 127.5 (C-4′), 125.9 (C-8), 124.4 (C-7), 121.2 (C-8a), 118.0 (C-5), 115.4 (C-9a), 115.0 (C-6′), 114.0 (C-2), 101.6 (C-4), 66.9 (-OCH_2_), 53.6 (C-2′), 35.7 (C-3′) ppm.

#### 3.2.19. (2-((9-.Oxo-9H-Xanthen-3-yl)oxy)acetyl)-L-serine (**50**)

The hydrolysis finished in 2 h to afford compound **50** as a white solid (39.03 mg, 72.05%). m.p.: 199–200 °C; IR (KBr): ṽ 3500–2400, 3306, 1740, 1665, 1625, 1556, 1478, 1465, 1442, 1261, 837, 759, 670, 650 cm^−1^. ^1^H NMR (400.14 MHz, DMSO-*d*_6_): *δ* = 8.28 (1H, *d*, *J* = 8.0 Hz, N-H), 8.18 (1H, *dd*, *J* = 7.9 and 1.6 Hz, H-8), 8.12 (1H, *d*, *J* = 8.8 Hz, H-1), 7.85 (1H, *ddd*, *J* = 8.7, 7.1 and 1.6 Hz, H-6), 7.63 (1H, *d*, *J* = 8.3 Hz, H-5), 7.47 (1H, *ddd*, *J* = 7.9, 7.1 and 0.8 Hz, H-7), 7.16 (1H, *d*, *J* = 2.4 Hz, H-4), 7.12 (1H, *dd*, *J* = 8.8 and 2.4 Hz, H-2), 4.81 (2H, *s*, OCH_2_), 4.37 (1H, *m*, H-2′), 3.79 (1H, *dd*, *J* = 10.9 and 5.2 Hz), 3.71 (1H, *dd*, *J* = 11.0 and 3.9 Hz, H-3′) ppm. ^13^C NMR (100.6 MHz, DMSO-*d*_6_): *δ* = 174.9 (C-9), 171.6 (C-1′), 166.9 (C=O amide), 163.3 (C-3), 157.3 (C-4a), 155.6 (C-10a), 135.1 (C-6), 127.6 (C-1), 125.9 (C-8), 124.4 (C-7), 121.2 (C-8a), 118.0 (C-5), 115.4 (C-9a), 114.0 (C-2), 101.7 (C-4), 67.0 (-OCH_2_), 61.1 (C-2′), 54.4 (C-3′) ppm.

#### 3.2.20. (2-((9-.Oxo-9H-Xanthen-3-yl)oxy)acetyl)-D-serine (**51**)

The hydrolysis finished in 2 h to afford compound **57** as a white solid (58.80 mg, 70.81%). m.p.: 199–202 °C; IR (KBr): ṽ 3400–2500, 3306, 1740, 1665, 1625, 1556, 1478, 1465, 1442, 1261, 837, 759, 670, 649 cm^−1^. ^1^H NMR (400.14 MHz, DMSO-*d*_6_): *δ* = 8.28 (1H, *d*, *J* = 8.0 Hz, N-H), 8.18 (1H, *dd*, *J* = 7.9 and 1.6 Hz, H-8), 8.12 (1H, *d*, *J* = 8.8 Hz, H-1), 7.85 (1H, *ddd*, *J* = 8.5, 7.1 and 1.6 Hz, H-6), 7.63 (1H, *d*, *J* = 8.3 Hz, H-5), 7.47 (1H, *ddd*, *J* = 7.9, 7.1 and 0.8 Hz, H-7), 7.16 (1H, *d*, *J* = 2.4 Hz, H-4), 7.12 (1H, *dd*, *J* = 8.8 and 2.4 Hz, H-2), 4.81 (2H, *s*, OCH_2_), 4.37 (1H, *m*, H-2′), 3.79 (1H, *dd*, *J* = 10.9 and 5.2 Hz), 3.71 (1H, *dd*, *J* = 11.0 and 3.9 Hz, H-3′) ppm. ^13^C NMR (100.6 MHz, DMSO-*d*_6_): *δ* = 174.9 (C-9), 171.6 (C-1′), 166.9 (C=O amide), 163.3 (C-3), 157.3 (C-4a), 155.6 (C-10a), 135.1 (C-6), 127.6 (C-1), 125.9 (C-8), 124.4 (C-7), 121.2 (C-8a), 118.0 (C-5), 115.4 (C-9a), 114.0 (C-2), 101.7 (C-4), 67.0 (-OCH_2_), 61.1 (C-2′), 54.4 (C-3′) ppm.

#### 3.2.21. (2-((9-.Oxo-9H-Xanthen-3-yl)oxy)acetyl)-L-aspartic acid (**52**)

The hydrolysis finished in 5 min to afford compound **52** as a white crystal solid (29.58 mg, 69.59%). m.p.: 129–130 °C; IR (KBr): ṽ 3600–2500, 3352, 1718, 1647, 1618, 1539, 1465, 1442, 1257, 829, 760, 668 cm^−1^. ^1^H NMR (300.13 MHz, DMSO-*d*_6_): *δ* = 8.54 (1H, *d*, *J* = 8.1 Hz, N-H), 8.18 (1H, *dd*, *J* = 8.0 and 1.6 Hz, H-8), 8.13 (1H, *d*, *J* = 8.7 Hz, H-1), 7.86 (1H, *ddd*, *J* = 8.6, 7.0 and 1.6 Hz, H-6), 7.64 (1H, *dd*, *J* = 8.4 and 1.0 Hz, H-5), 7.48 (1H, *ddd*, *J* = 8.0, 7.1 and 1.1 Hz, H-7), 7.13 (1H, *dd*, *J* = 8.7 and 2.4 Hz, H-2), 7.09 (1H, *d*, *J* = 2.4 Hz, H-4), 4.77 (2H, *s*, -OCH_2_ ), 4.65 (1H, *m*, H-2′), 2.79 (1H, *dd*, *J* = 16.8 and 5.9 Hz, H-3′), 2.69 (1H, *dd*, *J* = 16.7 and 6.8 Hz, H-3′) ppm. ^13^C NMR (75.5 MHz, DMSO-*d*_6_): *δ* = 175.1 (C-9), 172.2 (C-1′), 171.9 (C-4′), 166.9 (C=O amide), 163.3 (C-3), 157.4 (C-4a), 155.7 (C-10a), 135.2 (C-6), 127.7 (C-1), 126.0 (C-8), 124.5 (C-7), 121.2 (C-8a), 118.1 (C-5), 115.5 (C-9a), 114.0 (C-2), 101.7 (C-4), 67.1 (-OCH_2_), 48.4 (C-2′), 35.9 (C-3′) ppm.

#### 3.2.22. (2-((9-.Oxo-9H-Xanthen-3-yl)oxy)acetyl)-D-aspartic acid (**53**)

The hydrolysis finished in 5 min to afford compound **53** as a white solid (16.80 mg, 37.96%). IR (KBr): ṽ 3600–2400, 3406, 1727, 1648, 1618, 1538, 1465, 1445, 1256, 835, 760, 668 cm^−1^. ^1^H NMR (300.13 MHz, DMSO-*d*_6_): *δ* = 8.54 (1H, *d*, *J* = 8.2 Hz, N-H), 8.18 (1H, *dd*, *J* = 8.0 and 1.6 Hz, H-8), 8.13 (1H, *d*, *J* = 8.7 Hz, H-1), 7.86 (1H, *ddd*, *J* = 8.6, 7.0 and 1.6 Hz, H-6), 7.64 (1H, *dd*, *J* = 8.4 and 1.0 Hz, H-5), 7.48 (1H, *ddd*, *J* = 8.0, 7.1 and 1.0 Hz, H-7), 7.13 (1H, *dd*, *J* = 8.7 and 2.4 Hz, H-2), 7.09 (1H, *d*, *J* = 2.4 Hz, H-4), 4.77 (2H, *s*, -OCH_2_ ), 4.65 (1H, *m*, H-2′), 2.79 (1H, *dd*, *J* = 16.8 and 5.9 Hz, H-3′), 2.69 (1H, *dd*, *J* = 16.7 and 6.8 Hz, H-3′) ppm. ^13^C NMR (75.5 MHz, DMSO-*d*_6_): *δ* = 175.1 (C-9), 172.2 (C-1′), 171.9 (C-4′), 166.9 (C=O amide), 163.3 (C-3), 157.4 (C-4a), 155.7 (C-10a), 135.2 (C-6), 127.7 (C-1), 126.0 (C-8), 124.5 (C-7), 121.2 (C-8a), 118.1 (C-5), 115.5 (C-9a), 114.0 (C-2), 101.7 (C-4), 67.1 (-OCH_2_), 48.4 (C-2′), 35.9 (C-3′) ppm.

#### 3.2.23. (2-((9-.Oxo-9H-Xanthen-3-yl)oxy)acetyl)-L-glutamic acid (**54**)

The hydrolysis finished in 1 h to afford compound **54** as a yellow solid (92.40 mg, 84.1%). m.p.: 79–83 °C; IR (KBr): ṽ 3500–2000, 3437, 1717, 1618, 1552, 1465, 1437, 1260, 849, 793, 752, 668 cm^−1^. ^1^H NMR (400.14 MHz, DMSO-*d*_6_): *δ* = 8.48 (1H, *d*, *J* = 7.9 Hz, N-H), 8.17 (1H, *dd*, *J* = 7.9 and 1.5 Hz, H-8), 8.12 (1H, *d*, *J* = 9.3 Hz, H-1), 7.84 (1H, *ddd*, *J* = 8.6, 7.0 and 1.6 Hz, H-6), 7.62 (1H, *dd*, *J* = 8.6 and 1.1 Hz, H-5), 7.47 (1H, *ddd*, *J* = 7.9, 7.1 and 0.9 Hz, H-7), 7.11 (1H, *dd*, *J* = 9.3 and 2.4 Hz, H-2), 7.10 (1H, *d*, *J* = 2.4 Hz, H-4), 4.80 (1H, *d*, *J* = 14.9 Hz, OCH_2_), 4.75 (1H, *d*, *J* = 14.8 Hz, OCH_2_), 4.28 (1H, *m*, H-2′), 2.30 (2H, *t*, *J* = 7.5 Hz, H-4′), 2.04 (1H, *m*, H-3′), 1.87 (1H, *m*, H-3′) ppm. ^13^C NMR (100.6 MHz, DMSO-*d*_6_): *δ* = 174.9 (C-9), 173.8 (C-5′), 172.9 (C-1′), 167.1 (C=O amide), 163.3 (C-3), 157.3 (C-4a), 155.6 (C-10a), 135.1 (C-6), 127.6 (C-1), 125.9 (C-8), 124.4 (C-7), 121.2 (C-8a), 118.0 (C-5), 115.4 (C-9a), 114.0 (C-2), 101.6 (C-4), 67.0 (OCH_2_), 51.1 (C-2′), 30.1(C-3′), 26.1 (C-4′) ppm.

#### 3.2.24. (2-((9-.Oxo-9H-Xanthen-3-yl)oxy)acetyl)-D-glutamic acid (**55**)

The hydrolysis finished in 1 h to afford compound **61** as a yellow solid (65.47 mg, 43.03%). m.p.: 82–84 °C; IR (KBr): ṽ 3600–2500, 3447, 1717, 1644, 1618, 1552, 1465, 1437, 1260, 849, 753, 669 cm^−1^. ^1^H NMR (400.14 MHz, DMSO-*d*_6_): *δ* = 8.49 (1H, *d*, *J* = 7.9 Hz, N-H), 8.17 (1H, *dd*, *J* = 7.9 and 1.5 Hz, H-8), 8.12 (1H, *d*, *J* = 9.3 Hz, H-1), 7.84 (1H, *ddd*, *J* = 8.6, 7.0 and 1.6 Hz, H-6), 7.62 (1H, *dd*, *J* = 8.6 and 1.1 Hz, H-5), 7.47 (1H, *ddd*, *J* = 7.9, 7.1 and 0.9 Hz, H-7), 7.11 (1H, *dd*, *J* = 9.3 and 2.4 Hz, H-2), 7.10 (1H, *d*, *J* = 2.4 Hz, H-4), 4.80 (1H, *d*, *J* = 14.9 Hz, OCH_2_), 4.75 (1H, *d*, *J* = 14.8 Hz, OCH_2_), 4.28 (1H, *m*, H-2′), 2.30 (2H, *t*, *J* = 7.5 Hz, H-4′), 2.04 (1H, *m*, H-3′), 1.87 (1H, *m*, H-3′) ppm. ^13^C NMR (100.6 MHz, DMSO-*d*_6_): *δ* = 174.9 (C-9), 173.8 (C-5′), 172.9 (C-1′), 167.1 (C=O amide), 163.3 (C-3), 157.3 (C-4a), 155.6 (C-10a), 135.1 (C-6), 127.6 (C-1), 125.9 (C-8), 124.4 (C-7), 121.2 (C-8a), 118.0 (C-5), 115.4 (C-9a), 114.0 (C-2), 101.6 (C-4), 67.0 (OCH_2_), 51.1 (C-2′), 30.1(C-3′), 26.1 (C-4′) ppm.

#### 3.2.25. (2-((9-.Oxo-9H-Xanthen-3-yl)oxy)acetyl)-L-threonine (**56**)

The hydrolysis finished in 30 min to afford compound **56** as a white solid (58 mg, ~99%). Crystallization was still required. m.p.: 157–159 °C; IR (KBr): ṽ 3500–2000, 3407, 1769, 1648, 1620, 1537, 1465, 1447, 1423, 1255, 864, 831, 763, 669 cm^−1^. ^1^H NMR (300.13 MHz, DMSO-*d*_6_): *δ* = 8.22 (1H, *d*, *J* = 8.6 Hz, N-H), 8.18 (1H, *dd*, *J* = 8.0 and 1.7 Hz, H-8), 8.12 (1H, *d*, *J* = 8.8 Hz, H-1), 7.85 (1H, *ddd*, *J* = 8.6, 7.0 and 1.8 Hz, H-6), 7.62 (1H, *dd*, *J* = 8.4 and 1.0 Hz, H-5), 7.47 (1H, *ddd*, *J* = 8.0, 7.1 and 1.0 Hz, H-7), 7.16 (1H, *d*, *J* = 2.4 Hz, H-4), 7.12 (1H, *dd*, *J* = 8.8 and 2.4 Hz, H-2), 5.17 (1H, *d*, *J* = 5.5 Hz, C_3′_-OH), 4.86 (2H, *s*, OCH_2_), 4.27 (1H, *dd*, *J* = 8.8 and 3.1 Hz, H-2′), 4.19 (1H, *m*, H-3′), 1.07 (3H, *d*, *J* = 6.3 Hz, H-4′) ppm. ^13^C NMR (75.5 MHz, DMSO-*d*_6_): *δ* = 175.0 (C-9), 171.9 (C-1′), 167.4 (C=O amide), 163.4 (C-3), 157.4 (C-4a), 155.7 (C-10a), 135.2 (C-6), 127.7 (C-1), 126.0 (C-8), 124.5 (C-7), 121.2 (C-8a), 118.0 (C-5), 115.4 (C-9a), 114.1 (C-2), 101.7 (C-4), 67.0 (OCH_2_), 66.2 (C-3′), 57.5 (C-2′), 20.5 (C-4′) ppm.

#### 3.2.26. (2-((9-.Oxo-9H-Xanthen-3-yl)oxy)acetyl)-D-threonine (**57**)

The hydrolysis finished in 30 min to afford compound **57** as a white solid (41.02 mg, ~99%). Crystallization was still required. m.p.: 162–164 °C; IR (KBr): ṽ 3500–2000, 3408, 1769, 1648, 1620, 1537, 1465, 1446, 1255, 863, 831, 763, 669 cm^−1^. ^1^H NMR (300.13 MHz, DMSO-*d*_6_): *δ* = 8.22 (1H, *d*, *J* = 8.6 Hz, N-H), 8.18 (1H, *dd*, *J* = 8.0 and 1.5 Hz, H-8), 8.12 (1H, *d*, *J* = 8.8 Hz, H-1), 7.85 (1H, *ddd*, *J* = 8.6, 7.0 and 1.6 Hz, H-6), 7.62 (1H, *dd*, *J* = 8.4 and 1.0 Hz, H-5), 7.47 (1H, *ddd*, *J* = 8.0, 7.0 and 0.9 Hz, H-7), 7.16 (1H, *d*, *J* = 2.4 Hz, H-4), 7.12 (1H, *dd*, *J* = 8.8 and 2.4 Hz, H-2), 5.17 (1H, *d*, *J* = 5.5 Hz, C_3′_-OH), 4.86 (2H, *s*, OCH_2_), 4.27 (1H, *dd*, *J* = 8.8 and 3.1 Hz, H-2′), 4.19 (1H, *m*, H-3′), 1.07 (3H, *d*, *J* = 6.3 Hz, H-4′) ppm. ^13^C NMR (75.5 MHz, DMSO-*d*_6_): *δ* = 175.0 (C-9), 171.9 (C-1′), 167.4 (C=O amide), 163.4 (C-3), 157.4 (C-4a), 155.7 (C-10a), 135.2 (C-6), 127.7 (C-1), 126.0 (C-8), 124.5 (C-7), 121.2 (C-8a), 118.0 (C-5), 115.4 (C-9a), 114.1 (C-2), 101.7 (C-4), 67.0 (OCH_2_), 66.2 (C-3′), 57.5 (C-2′), 20.5 (C-4′) ppm.

#### 3.2.27. (2-((9-.Oxo-9H-Xanthen-3-yl)oxy)acetyl)-L-cysteine (**58**)

The hydrolysis finished in 10 h to afford compound **58** as a yellow solid (52.67 mg, 76.22%). m.p.: 130–132 °C; IR (KBr): ṽ 3500–2600, 3400, 1733, 1651, 1619, 1537, 1464, 1443, 1255, 835, 758, 669 cm^−1^. ^1^H NMR (400.14 MHz, DMSO-*d*_6_): *δ* = 8.57 (1H, *d*, *J* = 7.6 Hz, N-H), 8.17 (1H, *d*, *J* = 7.9 and 1.4 Hz, H-8), 8.10 (1H, d*, H-1), 7.84 (1H, *m*, H-6), 7.62 (1H, *dd*, *J* = 8.5 and 0.6 Hz, H-5), 7.46 (1H, *m*, H-7), 7.08 (1H, dd*, H-2), 7.08 (1H, d*, H-4), 4.91 (2H, *s*, OCH_2_), 4.53 (1H, *m*, H-2′), 2.91 (2H, *m*, H-3′) ppm. *The calculation was not possible. ^13^C NMR (100.6 MHz, DMSO-*d*_6_): *δ* = 174.8 (C-9), 171.7 (C-1′), 167.1 (C=O amide), 163.2 (C-3), 157.2 (C-4a), 155.2 (C-10a), 135.0 (C-6), 127.5 (C-1), 125.8 (C-8), 124.3 (C-7), 121.1 (C-8a), 117.9 (C-5), 115.3 (C-9a), 113.9 (C-2), 101.6 (C-4), 66.9 (OCH_2_), 51.8 (C-2′), 29.2 (C-3′) ppm.

#### 3.2.28. (2-((9-.Oxo-9H-Xanthen-3-yl)oxy)acetyl)-D-cysteine (**59**)

The hydrolysis finished in 10 h to afford compound **59** as a yellow solid (50.10 mg, 69.5%). m.p.: 131–133 °C; IR (KBr): ṽ 3500–2600, 3408, 1735, 1649, 1619, 1534, 1464, 1443, 1255, 831, 758, 668 cm^−1^. ^1^H NMR (400.14 MHz, DMSO-*d*_6_): *δ* = 8.57 (1H, *d*, *J* = 7.6 Hz, N-H), 8.17 (1H, *d*, *J* = 7.9 and 1.4 Hz, H-8), 8.10 (1H, d*, H-1), 7.84 (1H, *m*, H-6), 7.62 (1H, *dd*, *J* = 8.5 and 0.6 Hz, H-5), 7.46 (1H, *m*, H-7), 7.08 (1H, dd*, H-2), 7.08 (1H, d*, H-4), 4.91 (2H, *s*, OCH_2_), 4.53 (1H, *m*, H-2′), 2.91 (2H, *m*, H-3′) ppm. *The calculation was not possible. ^13^C NMR (100.6 MHz, DMSO-*d*_6_): *δ* = 174.8 (C-9), 171.7 (C-1′), 167.1 (C=O amide), 163.2 (C-3), 157.2 (C-4a), 155.2 (C-10a), 135.0 (C-6), 127.5 (C-1), 125.8 (C-8), 124.3 (C-7), 121.1 (C-8a), 117.9 (C-5), 115.3 (C-9a), 113.9 (C-2), 101.6 (C-4), 66.9 (OCH_2_), 51.8 (C-2′), 29.2 (C-3′) ppm.

#### 3.2.29. (2-((9-.Oxo-9H-Xanthen-3-yl)oxy)acetyl)-L-methionine (**60**)

The hydrolysis finished in 30 min to afford compound **60** as a white crystal solid (31.58 mg, 77.9%). m.p.: 164–166 °C; IR (KBr): ṽ 3300–3000, 3323, 1742, 1658, 1618, 1556, 1464, 1442, 1255, 861, 754, 666 cm^−1^. ^1^H NMR (400.14 MHz, DMSO-*d*_6_): *δ* = 8.52 (1H, *d*, *J* = 8.0 Hz, N-H), 8.18 (1H, *dd*, *J* = 7.9 and 1.6 Hz, H-8), 8.13 (1H, *d*, *J* = 9.5 Hz, H-1), 7.85 (1H, *ddd*, *J* = 8.5, 7.0 and 1.6 Hz, H-6), 7.63 (1H, *dd*, *J* = 8.4 and 1.0 Hz, H-5), 7.47 (1H, *ddd; J =* 8.8, 7.1 and 1.1, H-7), 7.12 (1H, *dd*, *J* = 9.5 and 2.4 Hz, H-2), 7.11 (1H, *d*, *J* = 2.4 Hz, H-4), 4.82 (1H, *d*, *J* = 14.8 Hz, OCH_2_), 4.76 (1H, *d*, *J* = 14.8 Hz, OCH_2_), 4.41 (1H, *m*, H-2′), 2.45 (2H, *m*, H-4′), 2.01 (3H, *s*, H-5′), 1.94 (2H, *m*, H-3′) ppm. ^13^C NMR (100.6 MHz, DMSO-*d*_6_): *δ* = 174.9 (C-9), 172.9 (C-1′), 167.1 (C=O amide), 163.3 (C-3), 157.3 (C-4a), 155.6 (C-10a), 135.1 (C-6), 127.6 (C-1), 125.9 (C-8), 124.4 (C-7), 121.2 (C-8a), 117.9 (C-5), 115.3 (C-9a), 114.1 (C-2), 101.6 (C-4), 67.0 (OCH_2_), 50.7 (C-2′), 30.3 (C-4′), 29.7 (C-5′), 14.5 (C-3′) ppm.

#### 3.2.30. (2-((9-.Oxo-9H-Xanthen-3-yl)oxy)acetyl)-D-methionine (**61**)

The hydrolysis finished in 30 min to afford compound **61** as a white crystal solid (27.70 mg, 71.1%). m.p.: 157–16o °C; IR (KBr): ṽ 3300–3000, 3324, 1743, 1658, 1618, 1541, 1465, 1442, 1249, 861, 821, 754, 666 cm^−1^. ^1^H NMR (400.14 MHz, DMSO-*d*_6_): *δ* = 8.52 (1H, *d*, *J* = 8.0 Hz, N-H), 8.18 (1H, *dd*, *J* = 7.9 and 1.6 Hz, H-8), 8.13 (1H, *d*, *J* = 9.5 Hz, H-1), 7.85 (1H, *ddd*, *J* = 8.5, 7.0 and 1.6 Hz, H-6), 7.63 (1H, *dd*, *J* = 8.4 and 1.0 Hz, H-5), 7.47 (1H, *ddd; J =* 8.8, 7.1 and 1.1, H-7), 7.12 (1H, *dd*, *J* = 9.5 and 2.4 Hz, H-2), 7.11 (1H, *d*, *J* = 2.4 Hz, H-4), 4.82 (1H, *d*, *J* = 14.8 Hz, OCH_2_), 4.76 (1H, *d*, *J* = 14.8 Hz, OCH_2_), 4.41 (1H, *m*, H-2′), 2.45 (2H, *m*, H-4′), 2.01 (3H, *s*, H-5′), 1.94 (2H, *m*, H-3′) ppm. ^13^C NMR (100.6 MHz, DMSO-*d*_6_): *δ* = 174.9 (C-9), 172.9 (C-1′), 167.1 (C=O amide), 163.3 (C-3), 157.3 (C-4a), 155.6 (C-10a), 135.1 (C-6), 127.6 (C-1), 125.9 (C-8), 124.4 (C-7), 121.2 (C-8a), 117.9 (C-5), 115.3 (C-9a), 114.1 (C-2), 101.6 (C-4), 67.0 (OCH_2_), 50.7 (C-2′), 30.3 (C-4′), 29.7 (C-5′), 14.5 (C-3′) ppm.

### 3.3. Anti-Inflammatory Activity Evaluation

The anti-inflammatory activity of the compounds was evaluated according to the method reported by Vieira et al. [40]. Briefly, a human peripheral blood monocyte cell line (THP-1) was cultured in complete RPMI (cRPMI; RPMI 1640 medium supplemented with 10% FBS and 1% antibiotic/antimycotic solution) at 37 °C in an atmosphere of 5% CO_2_. Then, the monocytes (5 × 10^5^ cells/well) were differentiated into macrophages in the presence of 100 nM of phorbol myristate acetate (PMA) added in the medium for 24 h of culture. After this period cells were gently washed and fresh medium was added. LPS (100 ng/mL in fresh medium) was added after 48 h of macrophage culture and incubated for 2 h to induce the inflammatory process. By diluting the stock sterilized (with 0.22 µm filter) solution of each compound in DMSO (20 mM) with cRPMI, different concentrations were obtained. Then, they were added, without removing the stimulus, to LPS-stimulated macrophages (concentrations in the wells of 1, 2.5, 5, 10, and 20 μM). After 22 h of incubation, the cell culture medium was harvested, centrifuged, and stored at −80 °C until the determination of IL-6 concentration. The metabolic activity and DNA quantifications were performed after cell washing. Non-LPS-stimulated and LPS-stimulated macrophages without treatment were used as controls. Dexamethasone (10 µM), a strong corticosteroid, was used as a positive control of reduction of IL-6 production. The NSAID indomethacin (10 µM) was also tested. The maximum percentage of DMSO in the wells was 0.1% and did not affect the cell viability.

#### 3.3.1. Metabolic Activity and DNA Quantification

The metabolic activity of the macrophages stimulated with LPS or not was determined by using the alamarBlue assay, as previously described [41]. The results are expressed in percentages related to the control (untreated LPS-stimulated macrophages).

The quantification of the DNA content was assessed using a fluorimetric dsDNA quantification kit, as previously described [42]. DNA content is expressed in relative concentrations to the control (untreated LPS-stimulated macrophages).

#### 3.3.2. IL-6 Quantification

The amount of IL-6 in the culture medium was determined using an ELISA kit, as previously described [43]. The IL-6 concentration (pg/mL) of each sample was obtained from a standard curve of cytokine concentration versus absorbance intensity. Then, the cytokine concentration was normalized by the respective DNA concentration. The results are expressed in percentages in relation to the control (untreated LPS-stimulated macrophages).

#### 3.3.3. Statistical Analysis

Results are expressed as mean ± standard deviation (SD) of 3 independent experiments, with a minimum of 3 replicates for each condition. Statistical analyses were performed using GraphPad Prism 8.0.1 software. One-way or two-way analysis of variance (ANOVA) and Dunnett’s multiple comparison method were used for cell assays. Differences between experimental groups were considered significant with a confidence interval of 95% whenever *p* < 0.05.

## 4. Conclusions

A library of sixty new CDXs was synthesized and their structures elucidated. The amino esters of the CDXs as well as their respective amino acid derivatives showed high enantiomeric purities with e.r. above 95.9%, except for CDXs of phenylglycine. The evaluation of the anti-inflammatory activity demonstrated that X1AELT (≈52%), X1AESPG (≈50%), X1AALPA (≈48%), X1AADTryp (≈48%), X1AEDTryp (≈47%), X1AALT (≈45%), and X1AEDT (≈43%) have a strong ability to reduce the IL-6 production at cytocompatible concentrations. In addition, differences in efficacies were observed between the amino esters of the CDXs and the amino acid derivatives, and enantioselectivity was also detected in many cases. The strategy to couple proteinogenic amino ester/acid to XCAR-1 proved to be adequate to obtain compounds with a better anti-inflammatory activity (depending on the amino acid) than the respective precursor. Thus, the development of this library of CDXs was important as a first approach in the search for new potential anti-inflammatory agents based on proteinogenic amino acids and amino ester derivatives of a carboxyxanthone. In order to explore the mechanism of action, other assays, such as inhibition of the enzyme cyclooxygenase, will be further evaluated.

## Figures and Tables

**Table 1:** Reaction time (h), yield (%), coupling reagent, and enantiomeric ratio (e.r.) of the synthesis of CDXs of amino esters (**2**–**31**) by coupling reactions of the amino ester with XCAR (**1**).

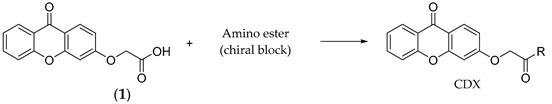					
CDX of Amino Esters	R	Reaction Time (h)	Yield (%)	Coupling Reagent	e.r. (%)
X1AELAla **2**	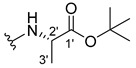	1	81	COMU	~100.0
X1AEDAla **3**	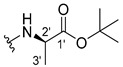	0.5	88	COMU	99.9
X1AEGli * 4	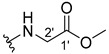	23	72	COMU	
X1AELIsoLeu **5**	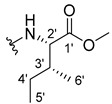	24	99	COMU	~100.0
X1AELLeu **6**	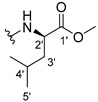	1	77	COMU	~100.0
X1AEDLeu **7**	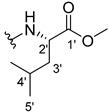	0.5	94	COMU	~100.0
X1AELProl **8**	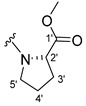	24	83	COMU	~100.0
X1AEDProl **9**	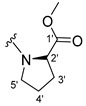	22	**	COMU	~100.0
X1AELVal **10**	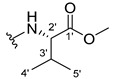	4	74	COMU	~100.0
X1AEDVal **11**	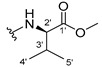	2	75	COMU	99.9
X1AELPG **12**	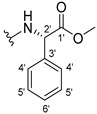	1	64	COMU	98.6
X1AEDPG **13**	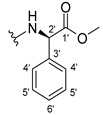	0.5	76	COMU	99.1
X1AELPA **14**	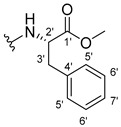	5	80	COMU	~100.0
X1AEDPA **15**	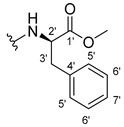	5	74	COMU	~100.0
X1AELTryp **16**	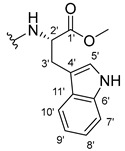	1	57	COMU	~100.0
X1AEDTryp **17**	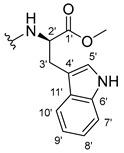	2	77	COMU	~100.0
X1AELT **18**	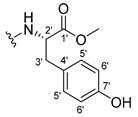	4	49	COMU	95.9
X1AEDT **19**	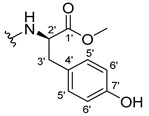	0.5	59	COMU	97.7
X1AELSer **20**	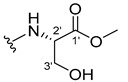	27	50	TBTU	97.9
X1AEDSer **21**	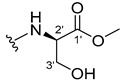	27	53	TBTU	98.9
X1AELAspA. **22**	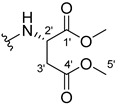	30	56	COMU	~100.0
X1AEDAspA. **23**	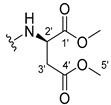	23	63	COMU	~100.0
X1AELGlutA. **24**	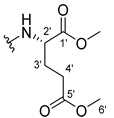	24	75	COMU	~100.0
X1AEDGlutA. **25**	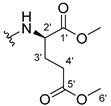	30	67	TBTU	~100.0
X1AELThreo **26**	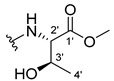	24	44	COMU	~100.0
X1AEDThreo **27**	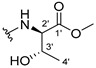	24	48	COMU	~100.0
X1AELCyst **28**	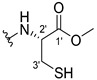	19	92	COMU	~100.0
X1AEDCyst **29**	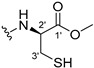	19	91	COMU	~100.0
X1AELMet **30**	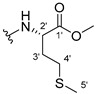	25	50	COMU	~100.0
X1AEDMet **31**	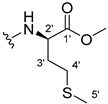	25	55	COMU	~100.0

* Not chiral; ** product in the form of oil, crystallization was not possible. COMU: (1-cyano-2-ethoxy-2-oxoethylidenaminooxy)dimethylamino-morpholino-carbenium hexafluorophosphate; TBTU: *O*-(benzotriazol-1-yl)-*N*,*N*,*N*’,*N*’-tetramethyluronium tetrafluoroborate.; e.r.: enantiomeric ratio.

**Table 2:** Reaction time (h), yield (%), and enantiomeric ratio (e.r.) of the synthesis of CDXs of amino acids (**32**–**61**).

	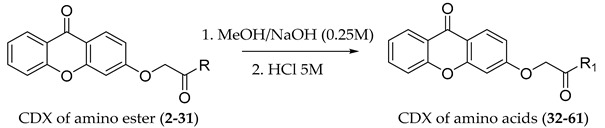				
CDX of Amino Acids	R	R_1_	Reaction Time (h)	Yield (%)	e.r (%)
X1AALAla * 32	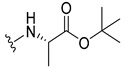	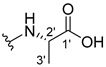	20	70	94.8
X1AADAla * 33	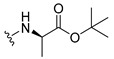	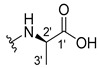	20	67	94.8
X1AAGli ** 34	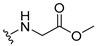	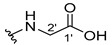	0.1667	~99	-
X1AALIsoLeu **35**	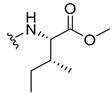	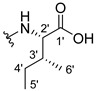	2	41	-
X1AALLeu **36**	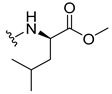	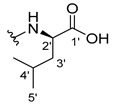	1	68	99.2
X1AADLeu **37**	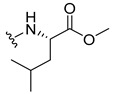	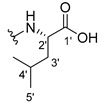	1	64	99.7
X1AALProl **38**	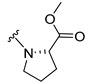	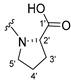	1	50	100.0
X1AADProl **39**	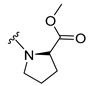	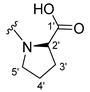	1	47	99.8
X1AALVal **40**	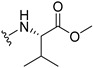	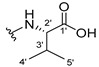	24	59	79.8
X1AADVal **41**	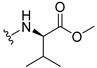	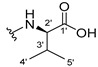	5	67	99.5
X1AALPG **42**	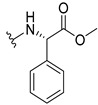	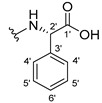	2	56	60.3
X1AADPG **43**	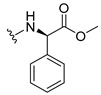	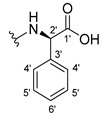	2	61	55.5
X1AALPA **44**	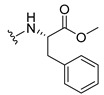	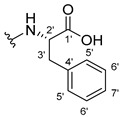	1	62	93.7
X1AADPA **45**	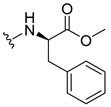	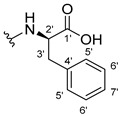	4	75	97.1
X1AALTryp **46**	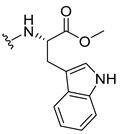	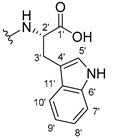	2	71	99.9
X1AADTryp **47**	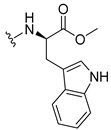	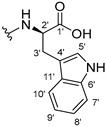	1	64	99.5
X1AALT **48**	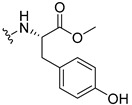	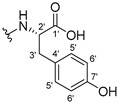	4	65	98.8
X1AADT **49**	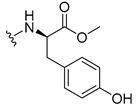	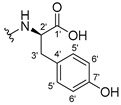	4	69	97.0
X1AALSer **50**	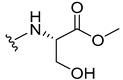	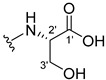	2	72	71.3
X1AADSer **51**	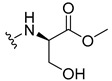	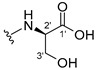	2	71	91.1
X1AALAspA. **52**	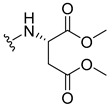	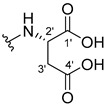	0.0833	70	-
X1AADAspA. **53**	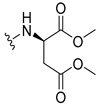	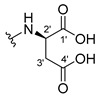	0.0833	38	-
X1AALGlutA. **54**	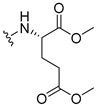	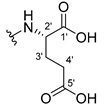	1	84	95.1
X1AADGlutA. **55**	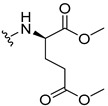	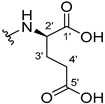	1	43	97.4
X1AALThreo **56**	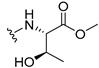	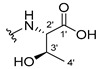	0.5	~99 *	100.0
X1AADThreo **57**	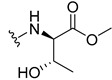	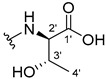	0.5	~99 *	99.8
X1AALCyst **58**	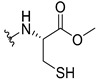	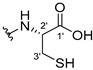	10	76	-
X1AADCyst **59**	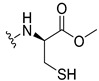	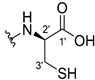	10	70	-
X1AALMet **60**	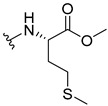	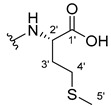	0.5	78	96.3
X1AADMet **61**	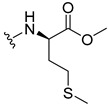	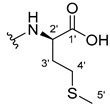	0.5	71	96.2

* Required an additional 1 mL of NaOH (1 M); ** not chiral; e.r.: enantiomeric ratio.

## Data Availability

The data presented in this study are available on request from the corresponding author.

## Supplementary Materials

The following supporting information can be downloaded at: https://www.mdpi.com/article/10.3390/ijms241210357/s1.

## References

[1] Limsuwan S., Trip E., Kouwen T., Piersma S., Hiranrat A., Mahabusarakam W., Voravuthikunchai S., Dijl J., Kayser O. (2009). Rhodomyrtone: A new candidate as natural antibacterial drug from Rhodomyrtus tomentosa. Phytomedicine.

[2] Fernandes C., Carraro M.L., Ribeiro J., Araujo J., Tiritan M.E., Pinto M.M.M. (2019). Synthetic Chiral Derivatives of Xanthones: Biological Activities and Enantioselectivity Studies. Molecules.

[3] Araújo J., Fernandes C., Pinto M., Tiritan M. (2019). Chiral Derivatives of Xanthones with Antimicrobial Activity. Molecules.

[4] Dutta T., Das T., Gopalakrishnan A.V., Saha S.C., Ghorai M., Nandy S., Kumar M., Radha, Ghosh A., Mukerjee N. (2023). Mangiferin: The miraculous xanthone with diverse pharmacological properties. Naunyn Schmiedebergs Arch. Pharmacol..

[5] Lee S.Y., Mojulat M.B.C., Thangaperagasam G.J.C., Surugau N., Tan S.A., John O.D. (2023). A Review on the Cytotoxic and Antimicrobial Properties of Xanthones from Cratoxylum cochinchinense. J. Trop. Life Sci..

[6] Fernandes C., Masawang K., Tiritan M.E., Sousa E., de Lima V., Afonso C., Bousbaa H., Sudprasert W., Pedro M., Pinto M. (2014). New chiral derivatives of xanthones: Synthesis and investigation of enantioselectivity as inhibitors of growth of human tumor cell lines. Bioorg. Med. Chem..

[7] Li R., Inbaraj B.S., Chen B.H. (2023). Quantification of Xanthone and Anthocyanin in Mangosteen Peel by UPLC-MS/MS and Preparation of Nanoemulsions for Studying Their Inhibition Effects on Liver Cancer Cells. Int. J. Mol. Sci..

[8] Koh J.-J., Lin S., Aung T.T., Lim F., Zou H., Bai Y., Li J., Lin H., Pang L.M., Koh W.L. (2015). Amino acid modified xanthone derivatives: Novel, highly promising membrane-active antimicrobials for multidrug-resistant Gram-positive bacterial infections. J. Med. Chem..

[9] Lu Y., Guan T., Wang S., Zhou C., Wang M., Wang X., Zhang K., Han X., Lin J., Tang Q. (2023). Novel xanthone antibacterials: Semi-synthesis, biological evaluation, and the action mechanisms. Bioorg. Med. Chem..

[10] Fernandes C., Palmeira A., Ramos I.I., Carneiro C., Afonso C., Tiritan M.E., Cidade H., Pinto P., Saraiva M., Reis S. (2017). Chiral Derivatives of Xanthones: Investigation of the Effect of Enantioselectivity on Inhibition of Cyclooxygenases (COX-1 and COX-2) and Binding Interaction with Human Serum Albumin. Pharmaceuticals.

[11] Yan Y., Li Y., Sa K., Sun D., Li H., Chen L. (2023). Xanthones and Phenylpropanoids from the Whole Herb of Swertia pseudochinensis and Their Anti-Inflammatory Activity. Chem. Biodivers..

[12] Kalick L.S., Khan H.A., Maung E., Baez Y., Atkinson A.N., Wallace C.E., Day F., Delgadillo B.E., Mondal A., Watanapokasin R. (2023). Mangosteen for malignancy prevention and intervention: Current evidence, molecular mechanisms, and future perspectives. Pharmacol. Res..

[13] Tatiya-aphiradee N., Chatuphonprasert W., Jarukamjorn K. (2019). Anti-inflammatory effect of Garcinia mangostana Linn. pericarp extract in methicillin-resistant Staphylococcus aureus-induced superficial skin infection in mice. Biomed. Pharmacother..

[14] Pinto M.M.M., Palmeira A., Fernandes C., Resende D.I.S.P., Sousa E., Cidade H., Tiritan M.E., Correia-Da-silva M., Cravo S. (2021). From natural products to new synthetic small molecules: A journey through the world of xanthones. Molecules.

[15] Nguyen L.A., He H., Chuong P.H. (2006). Chiral Drugs: An Overview. IJBS.

[16] Tiritan M.E., Ribeiro A.R., Fernandes C., Pinto M.M.M. (2016). Chiral Pharmaceuticals.

[17] Coelho M.M., Fernandes C., Remião F., Tiritan M.E. (2021). Enantioselectivity in drug pharmacokinetics and toxicity: Pharmacological relevance and analytical methods. Molecules.

[18] Smith S.W. (2009). Chiral toxicology: It’s the same thing…only different. Toxicol. Sci..

[19] Committee on Proprietary Medicinal Products (1994). Investigation of Chiral Active Substances; Directive 75/318/EEC as Amended. https://www.ema.europa.eu/en/investigation-chiral-active-substances-human-scientific-guideline.

[20] Hancu G., Modroiu A. (2022). Chiral Switch: Between Therapeutical Benefit and Marketing Strategy. Pharmaceuticals.

[21] Calcaterra A., D’Acquarica I. (2018). The market of chiral drugs: Chiral switches versus de novo enantiomerically pure compounds. J. Pharm. Biomed. Anal..

[22] Carraro M.L., Marques S., Silva A.S., Freitas B., Silva P.M.A., Pedrosa J., De Marco P., Bousbaa H., Fernandes C., Tiritan M.E. (2020). Synthesis of New Chiral Derivatives of Xanthones with Enantioselective Effect on Tumor Cell Growth and DNA Crosslinking. ChemistrySelect.

[23] Phyo Y.Z., Teixeira J., Gonçalves R., Palmeira A., Tiritan M.E., Bousbaa H., Pinto M.M.M., Fernandes C., Kijjoa A. (2021). Chiral derivatives of xanthones and benzophenones: Synthesis, enantioseparation, molecular docking, and tumor cell growth inhibition studies. Chirality.

[24] Fernandes C., Oliveira L., Tiritan M.E., Leitao L., Pozzi A., Noronha-Matos J.B., Correia-de-Sa P., Pinto M.M. (2012). Synthesis of new chiral xanthone derivatives acting as nerve conduction blockers in the rat sciatic nerve. Eur. J. Med. Chem..

[25] Durães F., Cravo S., Freitas-Silva J., Szemerédi N., Martins-Da-costa P., Pinto E., Tiritan M.E., Spengler G., Fernandes C., Sousa E. (2021). Enantioselectivity of chiral derivatives of xanthones in virulence effects of resistant bacteria. Pharmaceuticals.

[26] Jastrzebska-Wiesek M., Czarnecki R., Marona H. (2008). The anticonvulsant, local anesthetic and hemodynamic properties of some chiral aminobutanol derivatives of xanthone. Acta Pol. Pharm..

[27] Szkaradek N., Rapacz A., Pytka K., Filipek B., Zelaszczyk D., Szafranski P., Sloczynska K., Marona H. (2015). Cardiovascular activity of the chiral xanthone derivatives. Bioorg. Med. Chem..

[28] Marona H., Szkaradek N., Karczewska E., Trojanowska D., Budak A., Bober P., Przepiorka W., Cegla M., Szneler E. (2009). Antifungal and antibacterial activity of the newly synthesized 2-xanthone derivatives. Arch. Pharm. Chem. Life Sci..

[29] Marona H., Librowski T., Cegła M., Erdoğan C., Sahin N. (2008). Antiarrhythmic and antihypertensive activity of some xanthone derivatives. Acta Pol. Pharm..

[30] Rajtar G., Zolkowska D., Kleinrok Z., Marona H. (1999). Antiplatelets activity of some xanthone derivatives. Acta Pol. Pharm..

[31] Marona H., Szkaradek N., Kubacka M., Bednarski M., Filipek B., Cegla M., Szneler E. (2008). Synthesis and Evaluation of Some Xanthone Derivatives for Anti-Arrhythmic, Hypotensive Properties and Their Affinity for Adrenergic Receptors. Arch. Pharm..

[32] Francik R., Szkaradek N., Zelaszczk D., Marona H. (2016). Antioxidant activity of xanthones derivatives. Acta Pol. Pharm..

[33] Li H.L., Li X.M., Liu H., Meng L.H., Wang B.G. (2016). Two New Diphenylketones and a New Xanthone from Talaromyces islandicus EN-501, an Endophytic Fungus Derived from the Marine Red Alga Laurencia okamurai. Mar. Drugs.

[34] Szkaradek N., Sypniewski D., Waszkielewicz A.M., Gunia-Krzyzak A., Galilejczyk A., Galka S., Marona H., Bednarek I. (2016). Synthesis and in vitro Evaluation of the Anticancer Potential of New Aminoalkanol Derivatives of Xanthone. Anti Cancer Agents Med. Chem..

[35] Rech J., Sypniewski D., Żelaszczyk D., Szkaradek N., Rogóż W., Waszkielewicz A., Marona H., Bednarek I. (2021). Novel xanthone derivatives impair growth and invasiveness of colon cancer cells in vitro. Biomolecules.

[36] Chen X., Leng J., Rakesh K.P., Darshini N., Shubhavathi T., Vivek H.K., Mallesha N., Qin H.-L. (2017). Synthesis and molecular docking studies of xanthone attached amino acids as potential antimicrobial and anti-inflammatory agents. MedChemComm.

[37] Marx D., Wingen L.M., Schnakenburg G., Muller C.E., Scholz M.S. (2019). Fast, Efficient, and Versatile Synthesis of 6-amino-5-carboxamidouracils as Precursors for 8-Substituted Xanthines. Front. Chem..

[38] Montalbetti C., Falque V. (2005). Amide bond formation and peptide coupling. Tetrahedron.

[39] SwissADME. http://www.swissadme.ch/.

[40] Vieira S.F., Ferreira H., Neves N.M. (2020). Antioxidant and anti-inflammatory activities of cytocompatible salvia officinalis extracts: A comparison between traditional and soxhlet extraction. Antioxidants.

[41] Olival A., Vieira S.F., Gonçalves V.M.F., Cunha C., Tiritan M.E., Carvalho A., Reis R.L., Ferreira H., Neves N.M. (2022). Erythrocyte-derived liposomes for the treatment of inflammatory diseases. J. Drug Target..

[42] Loureiro D.R.P., Magalhães Á.F., Soares J.X., Pinto J., Azevedo C.M.G., Vieira S., Henriques A., Ferreira H., Neves N., Bousbaa H. (2020). Yicathins B and C and Analogues: Total Synthesis, Lipophilicity and Biological Activities. ChemMedChem.

[43] Guedes M., Vieira S.F., Reis R.L., Ferreira H., Neves N.M. (2021). Fishroesomes as carriers with antioxidant and anti-inflammatory bioactivities. Biomed. Pharmacother..

