# Supplementary material for: Synthesis and Anti-Inflammatory Evaluation of a Library of Chiral Derivatives of Xanthones Conjugated with Proteinogenic Amino Acids
Source: Int J Mol Sci. 2023 Jun 19;24(12):10357. doi: 10.3390/ijms241210357 (PMC10299275; doi:10.3390/ijms241210357)
Supplement: Supplementary file 1 [file ijms-24-10357-s001.zip › ijms-2390879-supplementary.pdf]

Synthesis and Anti-Inflammatory Evaluation of a Library of Chiral Derivatives of Xanthones Conjugated with Proteinogenic Amino Acids

Sara F. Vieira [1,2,†], Joana Araújo [3,†], Virgínia M. F. Gonçalves [4,5], Carla Fernandes [3,6], Madalena Pinto [3,6], Helena Ferreira [1,2,*], Nuno M. Neves [1,2] and Maria Elizabeth Tiritan [3,4,5,6,*]

1 3B's Research Group, I3BS—Research Institute on Biomaterials, Biodegradables and Biomimetics, University of Minho, Headquarters of the European Institute of Excellence on Tissue Engineering and Regenerative Medicine, AvePark, Parque de Ciência e Tecnologia, Zona Industrial da Gandra, Barco, 4805-017 Guimarães, Portugal; sara.vieira@i3bs.uminho.pt (S.F.V.); nuno@i3bs.uminho.pt (N.M.N.)

2 ICVS/3B's–PT Government Associate Laboratory, Braga, 4806-909 Guimarães, Portugal

3 Laboratório de Química Orgânica e Farmacêutica, Departamento de Ciências Químicas, Faculdade de Farmácia da Universidade do Porto, Rua Jorge de Viterbo Ferreira 228, 4050-313 Porto, Portugal; m.araujo@gmail.com (J.A.); cfernandes@ff.up.pt (C.F.); madalenakijjoa@gmail.com (M.P.)

4 TOXRUN—Toxicology Research Unit, University Institute of Health Sciences, CESPU, CRL, 4585-116 Gandra, Portugal; virginia.goncalves@cespu.pt

5 UNIPRO—Oral Pathology and Rehabilitation Research Unit, University Institute of Health Sciences (IUCS), CESPU, CRL, 4585-116 Gandra, Portugal

6 Interdisciplinary Centre of Marine and Environmental Research (CIIMAR), University of Porto, Terminal de Cruzeiros do Porto de Leixões, Avenida General Norton de Matos, S/N, 4450-208 Matosinhos, Portugal

* Correspondence: helenaferreira@i3bs.uminho.pt (H.F.); beth@ff.up.pt (M.E.T.)

† These authors contributed equally to this work.

1. ENANTIOMERIC PURITY

The normal-phase mode LC evaluation was carried out in a commercially available CSP Lux® 3 μm Cellulose-2 (150 x 4.6 mm), at room temperature. The mobile phase consisted in a mixture of 0.01% trifluoracetic acid in n-hexane:ethanol (50:50 *v/v*) at a flow rate of 1 mL/min in isocratic mode. The sample injections (10 μL) were carried out in duplicate, and the diode array detection was at 264 nm. The dead time (t_0) was considered to be the front solvent peak and was taken from each run. The stock solutions of all the CDXs were prepared in ethanol at the concentration of 1 mg/mL.

The retention factor (k) was calculated using equation S1, where t_R is the retention time.

$$k = \frac{[t_R - t_0]}{t_0} \text{ (S1)}$$

The separation factor (α) was calculated using equation S2.

$$\alpha = \frac{K_2}{K_1} \text{ (S2)}$$

The resolution factor (Rs) was calculated using equation S3, where t_{R1} and t_{R2} are the retention times of the first and second eluted enantiomers, respectively, and $1/2W_1$ and $1/2W_2$ are the corresponding peak width measured on half height.

$$Rs = 1.18 * \frac{[t_{R2} - t_{R1}]}{\left[\frac{1}{2}W_1 * \frac{1}{2}W_2\right]} \text{ (S3)}$$

The formula used for the calculation of the percentage of % e.r. was the following:, where E1 and E1 are the first and the second and eluted enantiomer, respectively.

$$\%\, e.r. = \frac{[E1]}{[E1]+[E2]} * 100 \text{ (S4)}$$

Before the enantiomeric purity evaluation of the synthesised CDXs, it was necessary to determine the chromatographic parameters of each enantiomeric mixture. For that, a stock solutions of enantiomeric mixtures of CDX in ethanol were prepared with equal aliquots of each enantiomer and analyzed at room temperature. The obtained chromatographic parameters of CDX of amino esters and peak purity are displayed in **Table S1.**

Table S1: cLC data of the enantioseparation of the of amino esters of CDXs in enantiomeric mixtures.

Enantiomeric Mixtures	RT (min) D	RT (min) L	Peak Purity D	Peak Purity L	k_1 D	k_2 L	Rs	α
X1AE_Ala	9.34	6.40	1.0000	1.0000	3.94	2.39	6.34	1.65
X1AE_Leu	9.22	7.14	1.0000	1.0000	4.15	2.99	4.24	1.39
X1AE_Prol	27.09	17.76	0.9995	1.0000	13.34	8.40	4.59	1.59
X1AE_Val	12.10	5.32	1.0000	1.0000	5.40	1.96	12.45	2.76

X1AE_PG	16.00	12.99	1.0000	1.0000	7.47	5.87	3.62	1.27
X1AE_PA	23.84	13.16	1.0000	1.0000	12.32	6.35	9.86	1.94
X1AE_Tryp	17.12	12.57	0.9997	1.0000	8.56	6.03	4.65	1.42
X1AE_T	12.27	8.44	0.9999	1.0000	5.49	3.47	5.48	1.59
X1AE_Ser	19.71	17.68	1.0000	1.0000	10.01	8.88	1.87	1.13
X1AE_AspA.	30.34	24.31	0.9999	0.9999	15.95	12.58	3.87	1.27
X1AE_GlutA.	21.54	19.22	0.9998	0.9998	11.03	9.74	1.96	1.13
X1AE_Threo	12.34	8.18	1.0000	1.0000	5.89	3.57	6.25	1.65
X1AE_Cys	15.28	12.64	1.0000	0.9998	7.08	5.69	3.36	1.25
X1AE_Met	15.92	12.69	1.0000	1.0000	7.89	6.09	3.91	1.30

RT: Retention Time; k: Retention Factor; Alfa (α): Enantioselectivity; Rs: Resolution.

The same evaluation was performed with amino acids of the CDXs of , that are presented in **Table S2**.

Table S2: cLC data of the enantioseparation of the amino acids of CDXs in enantiomeric mixtures.

**Enantiomeric Mixtures**	**RT** (min) D	**RT** (min) L	**Peak Purity** D	**Peak Purity** L	**K** D	**K** L	**Rs**	**α**
X1AA_Ala	6.68	5.46	1.0000	1.0000	2.73	2.05	2.88	1.34
X1AA_Leu	7.63	5.57	1.0000	1.0000	3.26	2.11	4.19	1.54
X1AA_Prol	11.27	9.00	1.0000	1.0000	5.30	4.03	1.72	1.31
X1AA_Val	5.73	4.77	1.0000	1.0000	2.20	1.67	2.40	1.32
X1AA_PG	8.83	7.68	1.0000	1.0000	3.67	3.06	1.93	1.20
X1AA_PA	8.83	7.16	1.0000	0.9999	3.93	3.00	3.00	1.31
X1AA_Tryp	11.80	8.98	1.0000	1.0000	5.59	4.02	3.57	1.39
X1AA_Tyr	8.40	6.77	1.0000	1.0000	3.69	2.78	2.60	1.33
X1AA_Ser	10.48	7.76	0.9999	0.9999	4.85	3.34	3.75	1.46

X1AA_AspA	7.20	6.56	0.9998	1.0000	3.02	2.66	1.10	1.13
X1AA_GlutA	6.78	5.44	1.0000	1.0000	2.59	1.88	2.43	1.38
X1AA_Threo	10.63	5.59	1.0000	1.0000	4.94	2.12	6.42	2.33
X1AA_Met	8.20	6.75	1.0000	1.0000	3.58	2.77	2.78	1.30

RT: Retention Time; K: Retention Factor; Alfa (α): Enantioselectivity; Rs: Resolution.

^{1}H and ^{13}C NMR spectra of CDXs

As the spectra of the enantiomers are identical, the spectra of only one of each enantiomeric pair was selected (Figure S1):

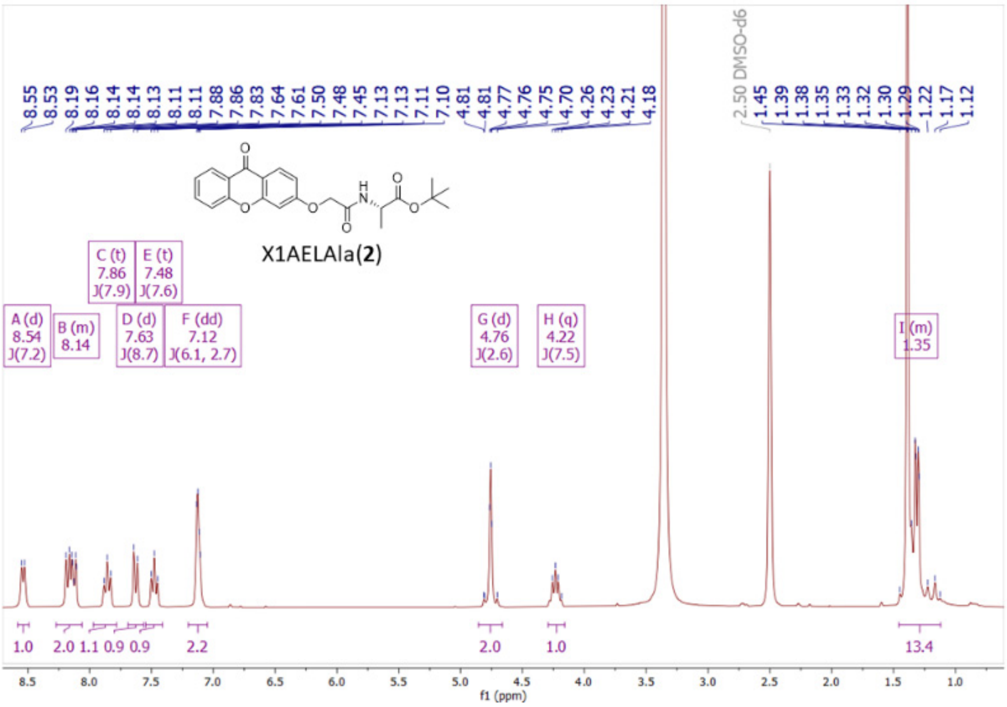

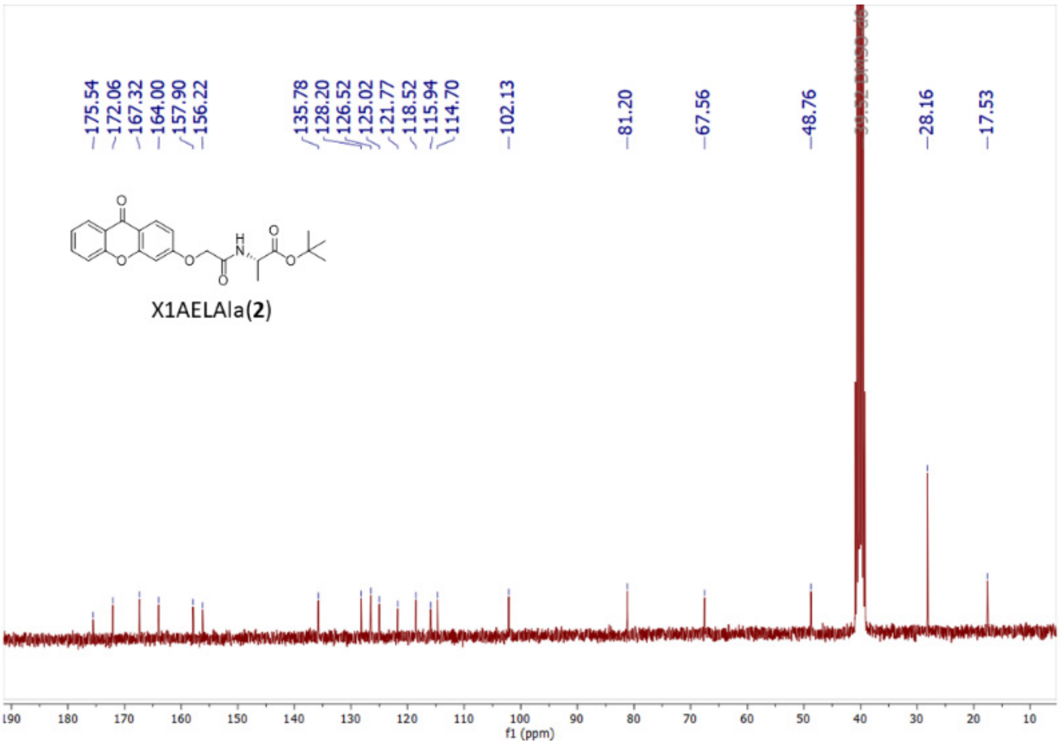

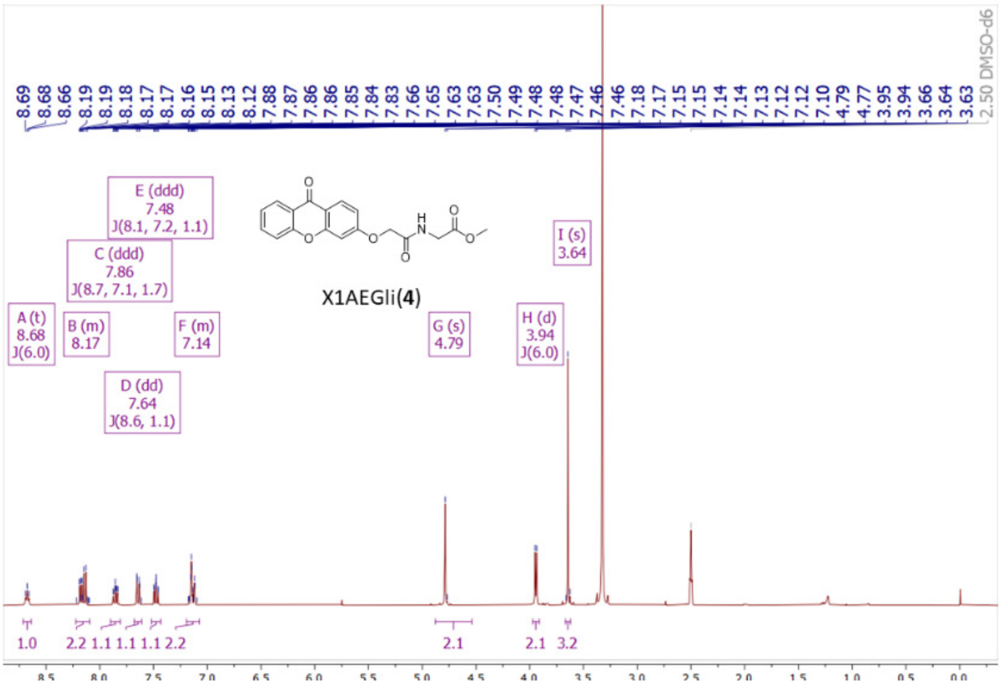
8.69 8.68 8.66 8.19 8.19 8.18 8.17 8.17 8.16 8.15 8.13 8.12 7.88 7.87 7.86 7.86 7.85 7.84 7.83 7.66 7.65 7.63 7.63 7.50 7.49 7.48 7.48 7.47 7.46 7.46 7.18 7.17 7.15 7.15 7.14 7.14 7.13 7.12 7.12 7.10 4.79 4.77 3.95 3.94 3.66 3.64 3.63
2.50 DMSO-d6
E (ddd) 7.48 J(8.1, 7.2, 1.1)
C (ddd) 7.86 J(8.7, 7.1, 1.7)
A (t) 8.68 J(6.0)
B (m) 8.17
F (m) 7.14
D (dd) 7.64 J(8.6, 1.1)
G (s) 4.79
H (d) 3.94 J(6.0)
I (s) 3.64
X1AEGli(**4**)
1.0 2.2 1.1 1.1 1.1 2.2 2.1 2.1 3.2
8.5 8.0 7.5 7.0 6.5 6.0 5.5 5.0 4.5 4.0 3.5 3.0 2.5 2.0 1.5 1.0 0.5 0.0

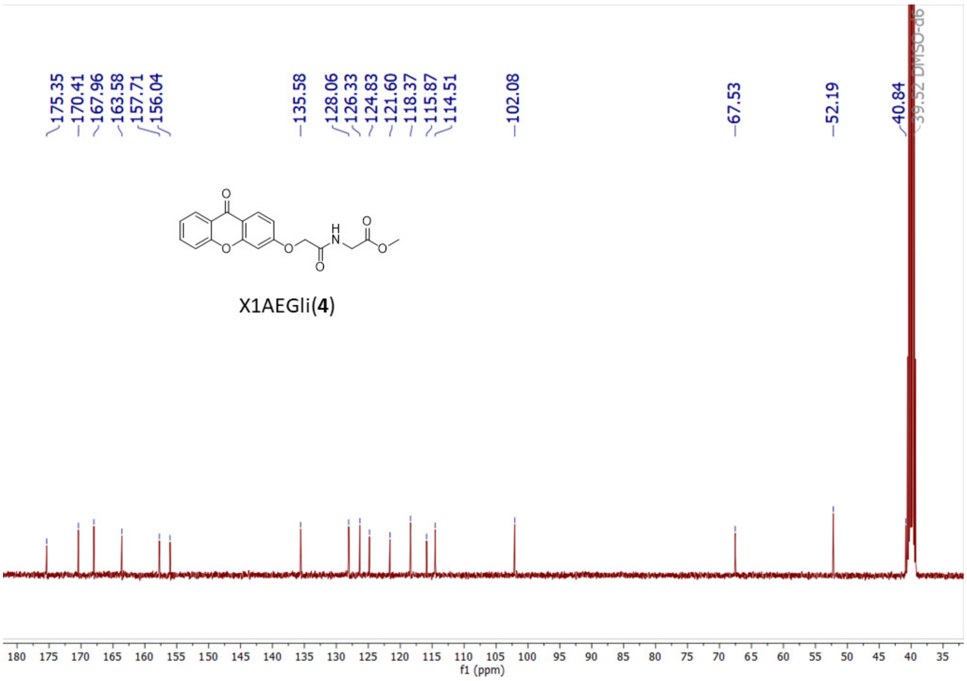
175.35 170.41 167.96 163.58 157.71 156.04 135.58 128.06 126.33 124.83 121.60 118.37 115.87 114.51 102.08 67.53 52.19 40.84
39.52 DMSO-d6
X1AEGli(**4**)
180 175 170 165 160 155 150 145 140 135 130 125 120 115 110 105 100 95 90 85 80 75 70 65 60 55 50 45 40 35
f1 (ppm)

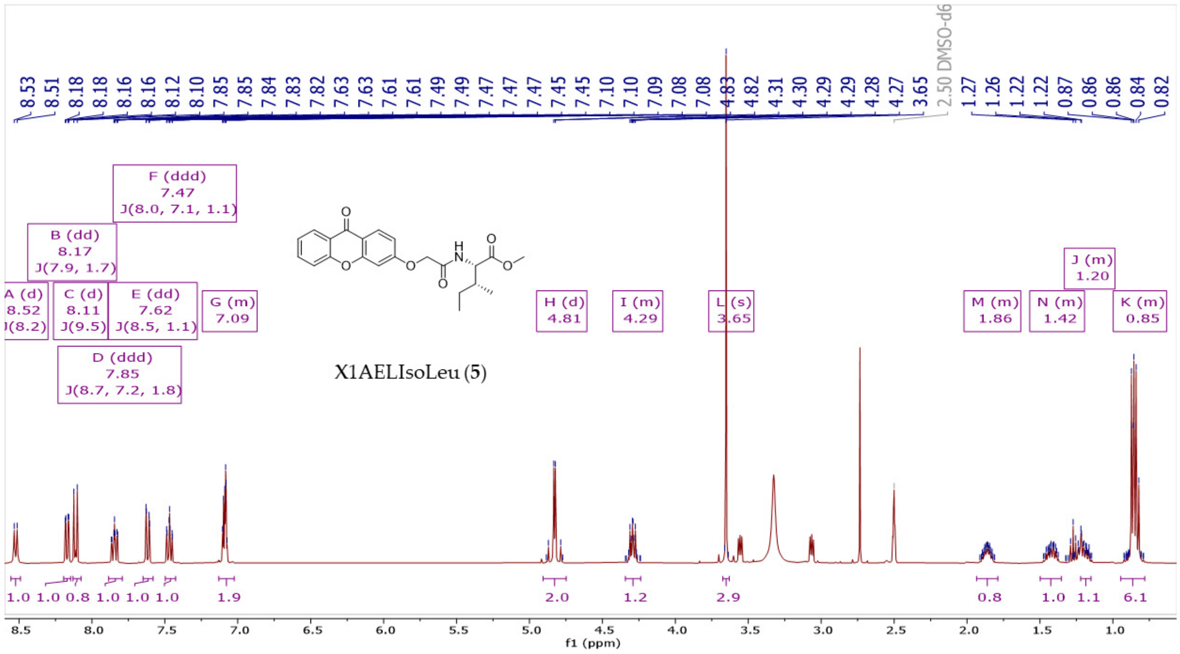
8.53 8.51 8.18 8.18 8.16 8.16 8.12 8.10 7.85 7.85 7.84 7.83 7.82 7.63 7.63 7.61 7.61 7.49 7.49 7.47 7.47 7.47 7.45 7.45 7.10 7.10 7.09 7.08 7.08 4.83 4.82 4.31 4.30 4.29 4.29 4.28 4.27 3.65
2.50 DMSO-d6
1.27 1.26 1.22 1.22 0.87 0.86 0.86 0.84 0.82
F (ddd) 7.47 J(8.0, 7.1, 1.1)
B (dd) 8.17 J(7.9, 1.7)
A (d) 8.52 J(8.2)
C (d) 8.11 J(9.5)
E (dd) 7.62 J(8.5, 1.1)
G (m) 7.09
D (ddd) 7.85 J(8.7, 7.2, 1.8)
H (d) 4.81
I (m) 4.29
L (s) 3.65
M (m) 1.86
N (m) 1.42
J (m) 1.20
K (m) 0.85
X1AELIsoLeu (5)
1.0 1.0 0.8 1.0 1.0 1.0 1.9 2.0 1.2 2.9 0.8 1.0 1.1 6.1
8.5 8.0 7.5 7.0 6.5 6.0 5.5 5.0 4.5 4.0 3.5 3.0 2.5 2.0 1.5 1.0
f1 (ppm)

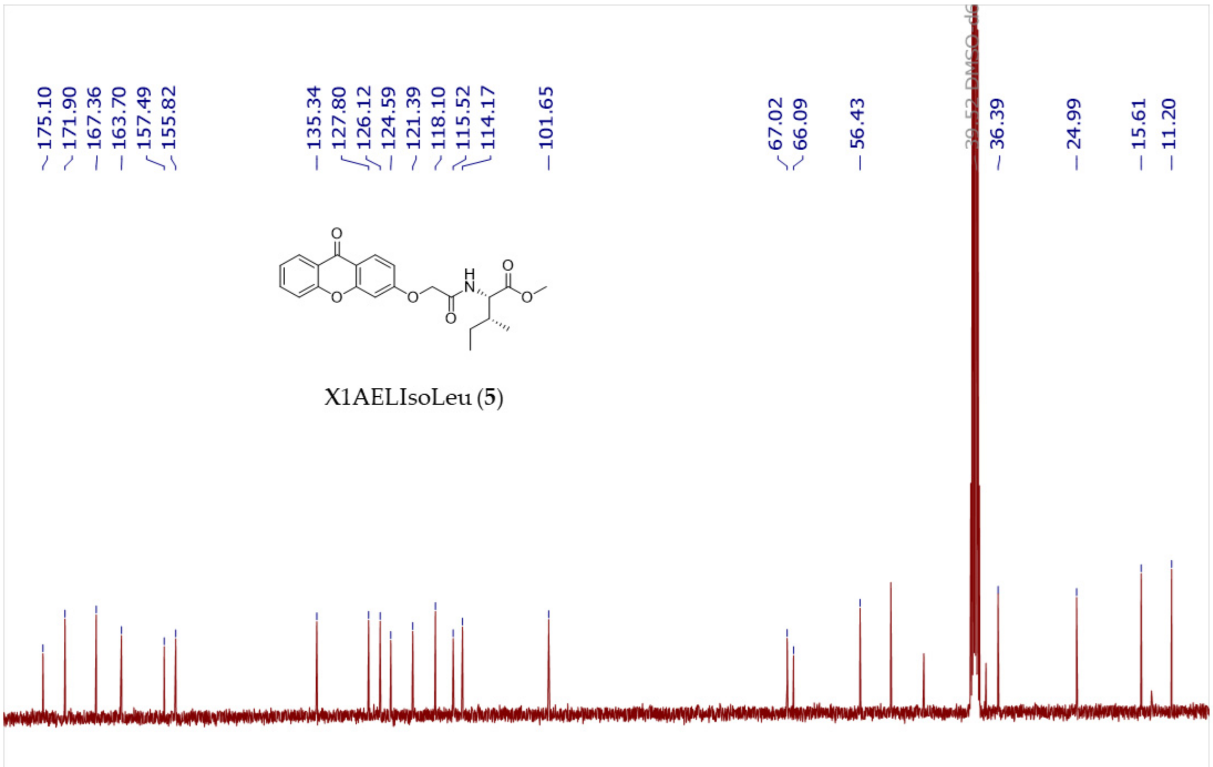
175.10 171.90 167.36 163.70 157.49 155.82
135.34 127.80 126.12 124.59 121.39 118.10 115.52 114.17
101.65
67.02 66.09
56.43
DMSO-d6
36.39
24.99
15.61 11.20
X1AELIsoLeu (5)

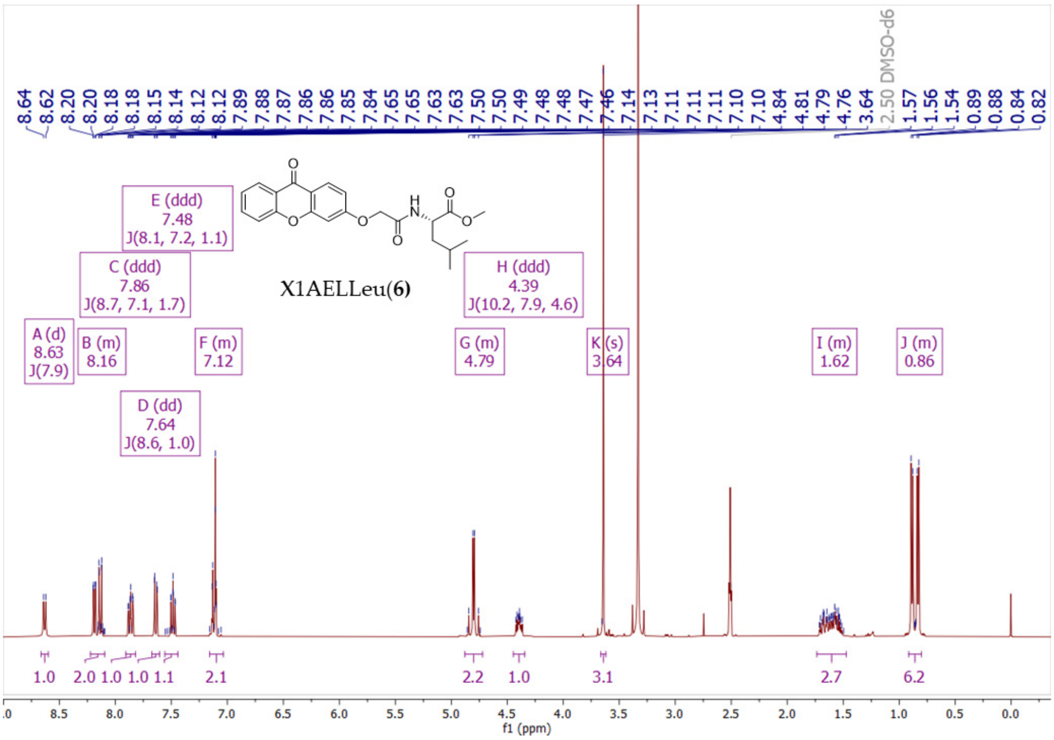
X1AELLeu(6)
A (d) 8.63 J(7.9)
B (m) 8.16
C (ddd) 7.86 J(8.7, 7.1, 1.7)
D (dd) 7.64 J(8.6, 1.0)
E (ddd) 7.48 J(8.1, 7.2, 1.1)
F (m) 7.12
G (m) 4.79
H (ddd) 4.39 J(10.2, 7.9, 4.6)
K (s) 3.64
I (m) 1.62
J (m) 0.86
2.50 DMSO-d6
f1 (ppm)

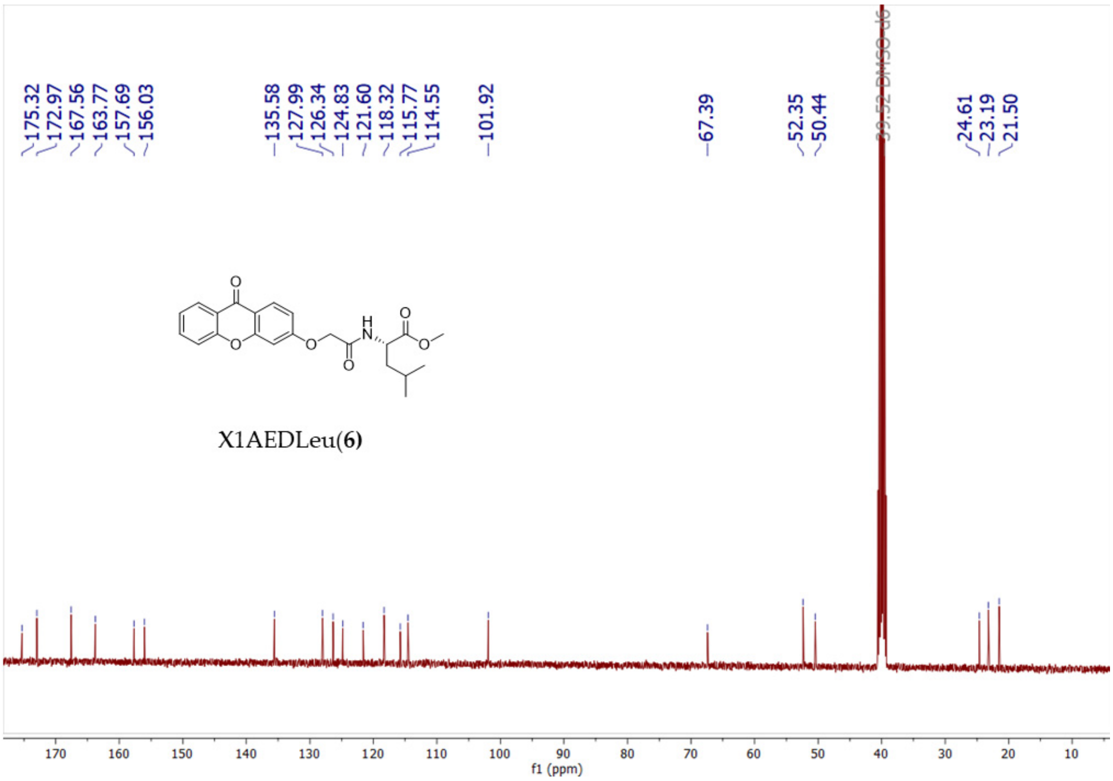
X1AEDLeu(6)
175.32
172.97
167.56
163.77
157.69
156.03
135.58
127.99
126.34
124.83
121.60
118.32
115.77
114.55
101.92
67.39
52.35
50.44
24.61
23.19
21.50
f1 (ppm)

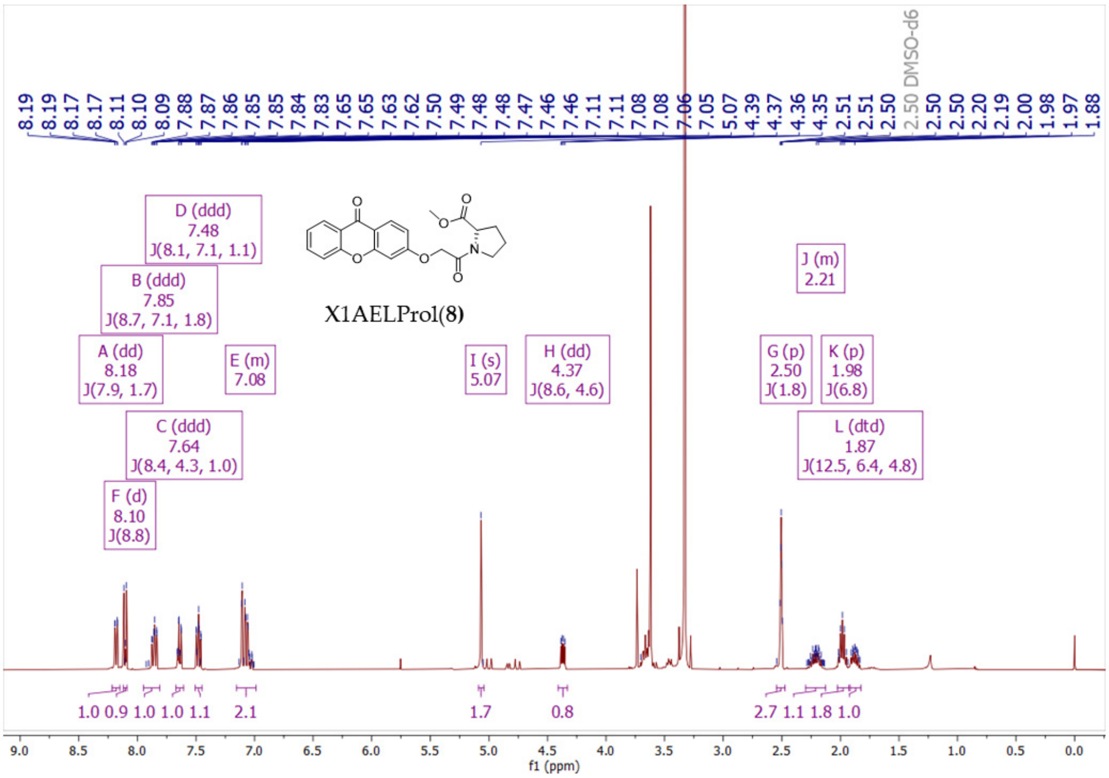
8.19
8.19
8.17
8.17
8.11
8.10
8.09
7.88
7.87
7.86
7.85
7.85
7.84
7.83
7.65
7.65
7.63
7.62
7.50
7.49
7.48
7.48
7.47
7.46
7.46
7.11
7.11
7.08
7.06
7.05
5.07
4.39
4.37
4.36
4.35
2.51
2.51
2.50
2.50 DMSO-d6
2.50
2.50
2.20
2.19
2.00
1.98
1.97
1.88
D (ddd) 7.48 J(8.1, 7.1, 1.1)
B (ddd) 7.85 J(8.7, 7.1, 1.8)
A (dd) 8.18 J(7.9, 1.7)
E (m) 7.08
C (ddd) 7.64 J(8.4, 4.3, 1.0)
F (d) 8.10 J(8.8)
I (s) 5.07
H (dd) 4.37 J(8.6, 4.6)
J (m) 2.21
G (p) 2.50 J(1.8)
K (p) 1.98 J(6.8)
L (dtd) 1.87 J(12.5, 6.4, 4.8)
X1AELProl(8)
1.0 0.9 1.0 1.0 1.1 2.1 1.7 0.8 2.7 1.1 1.8 1.0
9.0 8.5 8.0 7.5 7.0 6.5 6.0 5.5 5.0 4.5 4.0 3.5 3.0 2.5 2.0 1.5 1.0 0.5 0.0
f1 (ppm)

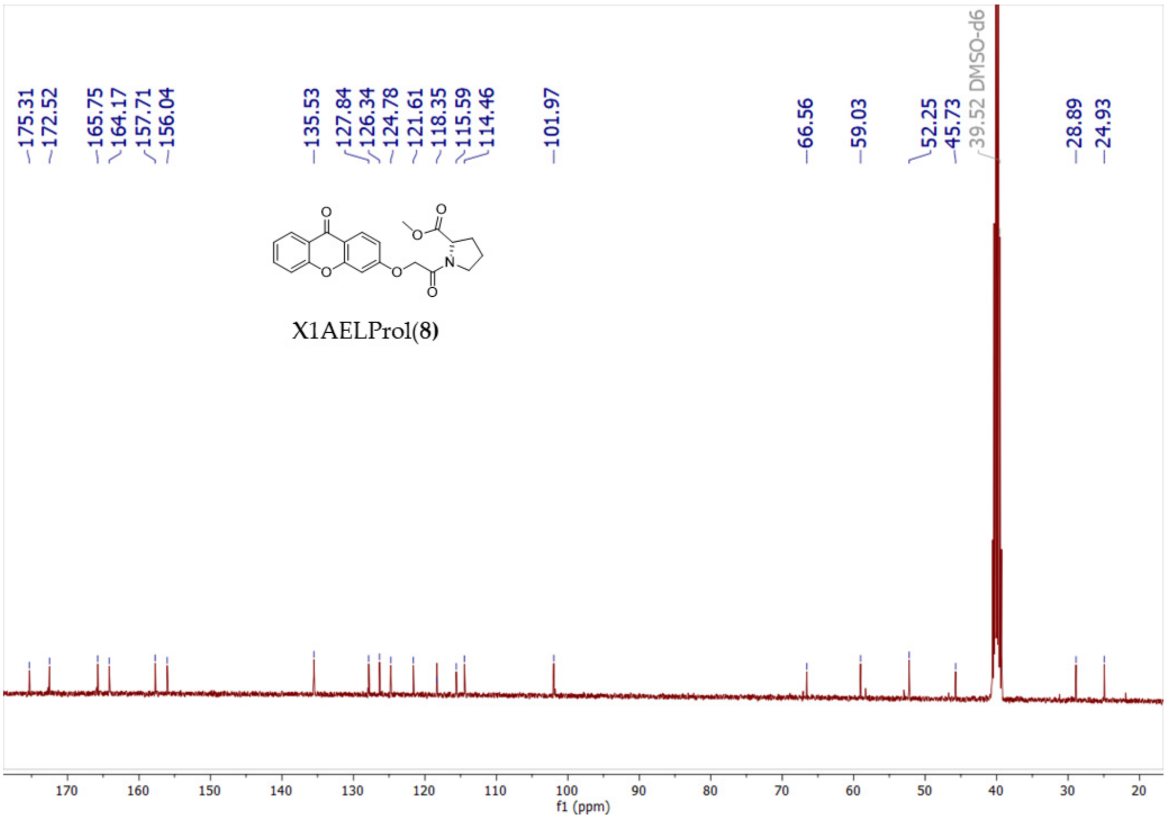
175.31
172.52
165.75
164.17
157.71
156.04
135.53
127.84
126.34
124.78
121.61
118.35
115.59
114.46
101.97
66.56
59.03
52.25
45.73
39.52 DMSO-d6
28.89
24.93
X1AELProl(8)
170 160 150 140 130 120 110 100 90 80 70 60 50 40 30 20
f1 (ppm)

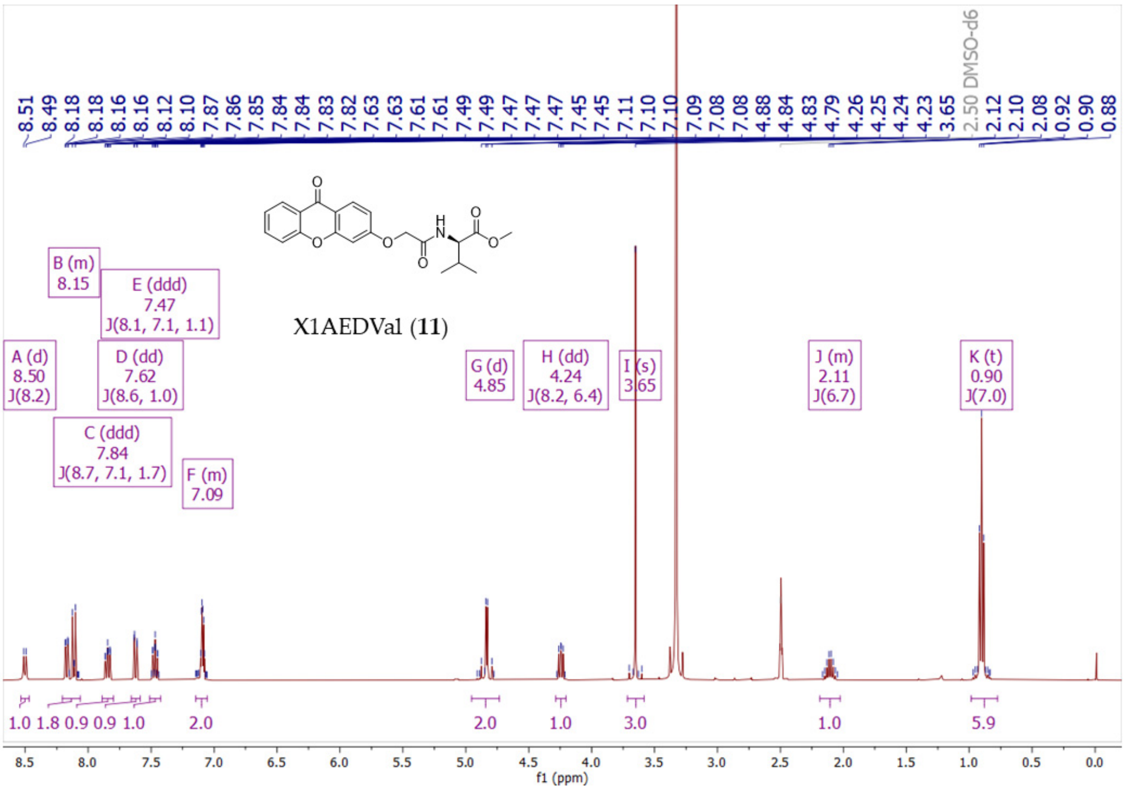
8.51 8.49 8.18 8.18 8.16 8.16 8.12 8.10 7.87 7.86 7.85 7.84 7.84 7.83 7.82 7.63 7.63 7.61 7.61 7.49 7.49 7.47 7.47 7.47 7.45 7.45 7.11 7.10 7.10 7.09 7.08 7.08 4.88 4.84 4.83 4.79 4.26 4.25 4.24 4.23 3.65 2.50 DMSO-d6 2.12 2.10 2.08 0.92 0.90 0.88
X1AEDVal (11)
A (d) 8.50 J(8.2)
B (m) 8.15
C (ddd) 7.84 J(8.7, 7.1, 1.7)
D (dd) 7.62 J(8.6, 1.0)
E (ddd) 7.47 J(8.1, 7.1, 1.1)
F (m) 7.09
G (d) 4.85
H (dd) 4.24 J(8.2, 6.4)
I (s) 3.65
J (m) 2.11 J(6.7)
K (t) 0.90 J(7.0)
1.0 1.8 0.9 0.9 1.0 2.0 2.0 1.0 3.0 1.0 5.9
f1 (ppm)

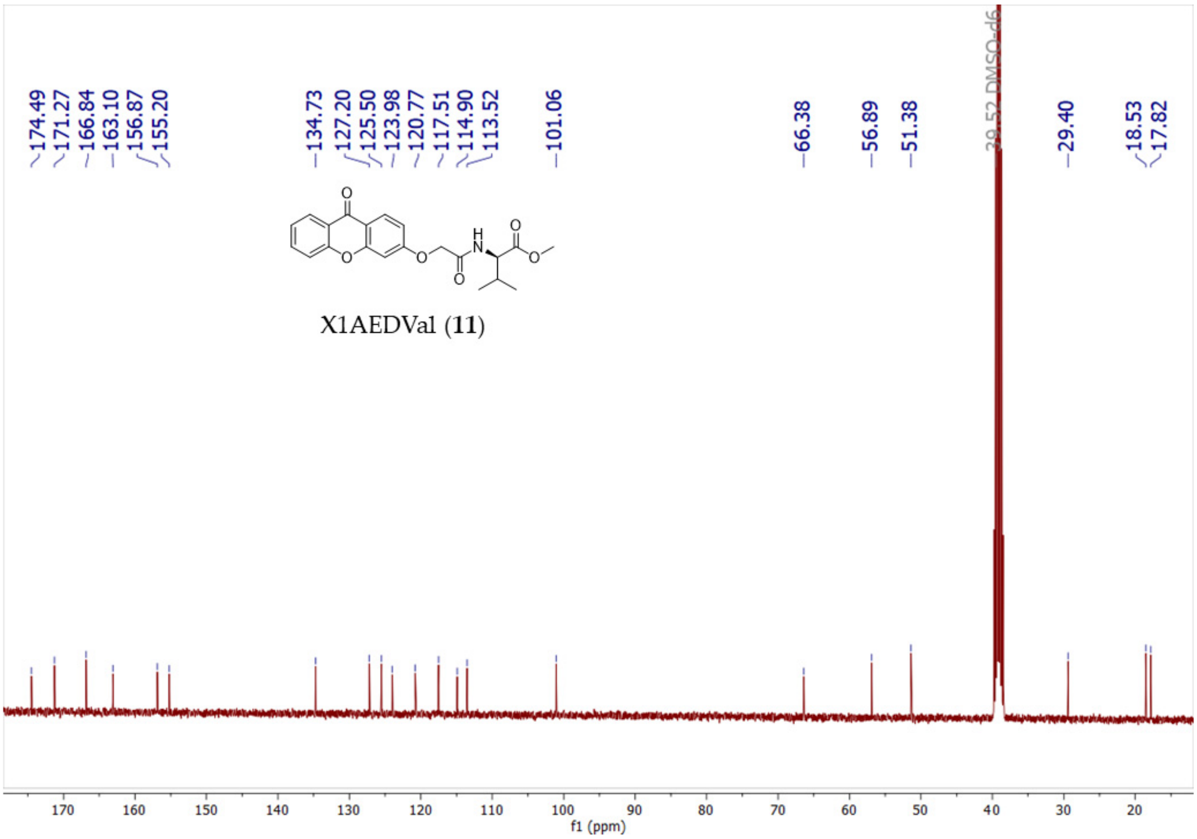
174.49 171.27 166.84 163.10 156.87 155.20 134.73 127.20 125.50 123.98 120.77 117.51 114.90 113.52 101.06 66.38 56.89 51.38 39.52 DMSO-d6 29.40 18.53 17.82
X1AEDVal (11)
f1 (ppm)

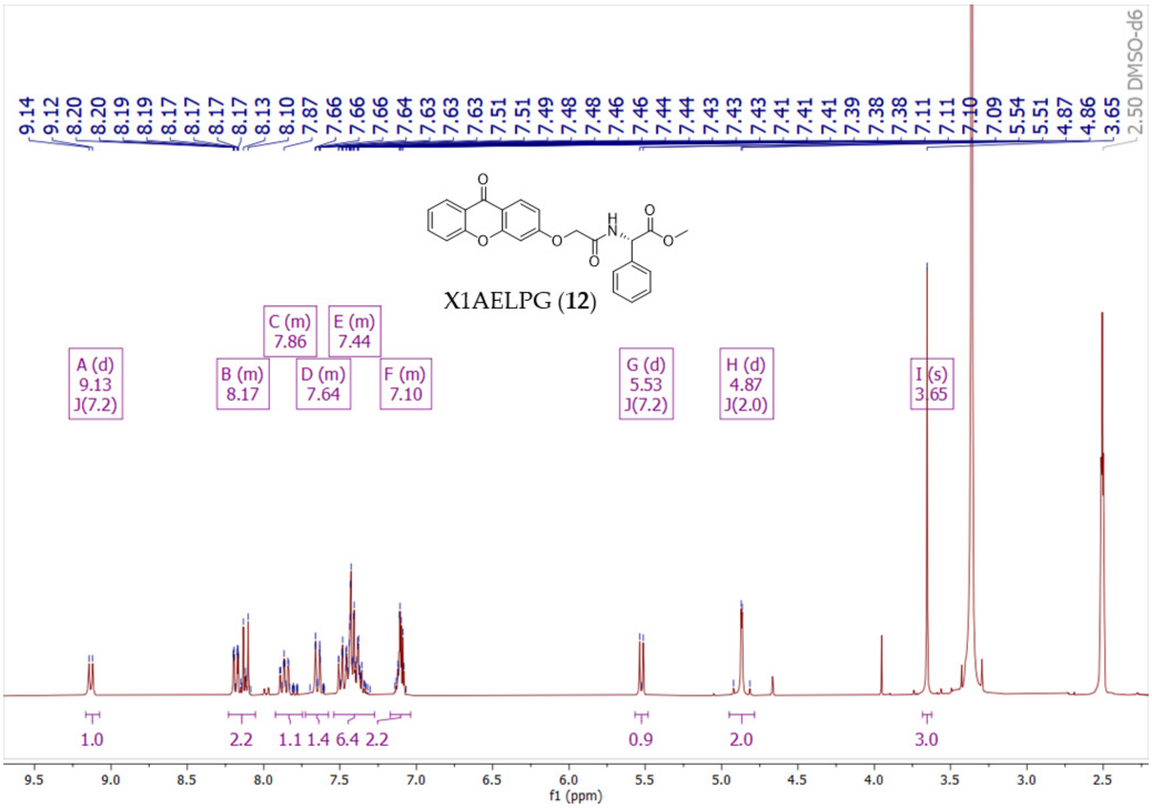

9.14
9.12
8.20
8.20
8.19
8.19
8.17
8.17
8.17
8.17
8.13
8.10
7.87
7.66
7.66
7.66
7.64
7.63
7.63
7.63
7.51
7.51
7.49
7.48
7.48
7.46
7.46
7.44
7.44
7.44
7.43
7.43
7.43
7.41
7.41
7.41
7.39
7.38
7.38
7.11
7.11
7.10
7.09
5.54
5.51
4.87
4.86
3.65
2.50 DMSO-d6
X1AELPG (12)
A (d)
9.13
J(7.2)
B (m)
8.17
C (m)
7.86
D (m)
7.64
E (m)
7.44
F (m)
7.10
G (d)
5.53
J(7.2)
H (d)
4.87
J(2.0)
I (s)
3.65
1.0
2.2
1.1
1.4
6.4
2.2
0.9
2.0
3.0
f1 (ppm)

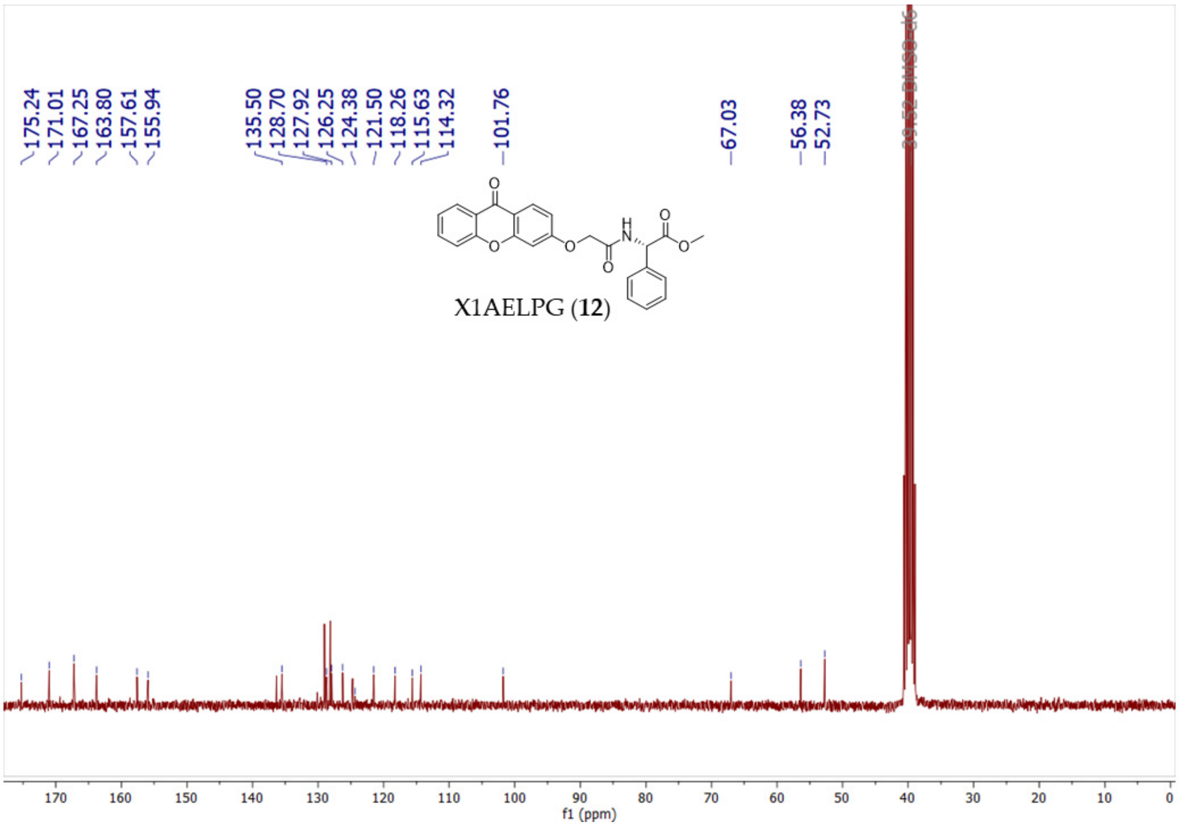

175.24
171.01
167.25
163.80
157.61
155.94
135.50
128.70
127.92
126.25
124.38
121.50
118.26
115.63
114.32
101.76
67.03
56.38
52.73
X1AELPG (12)
f1 (ppm)

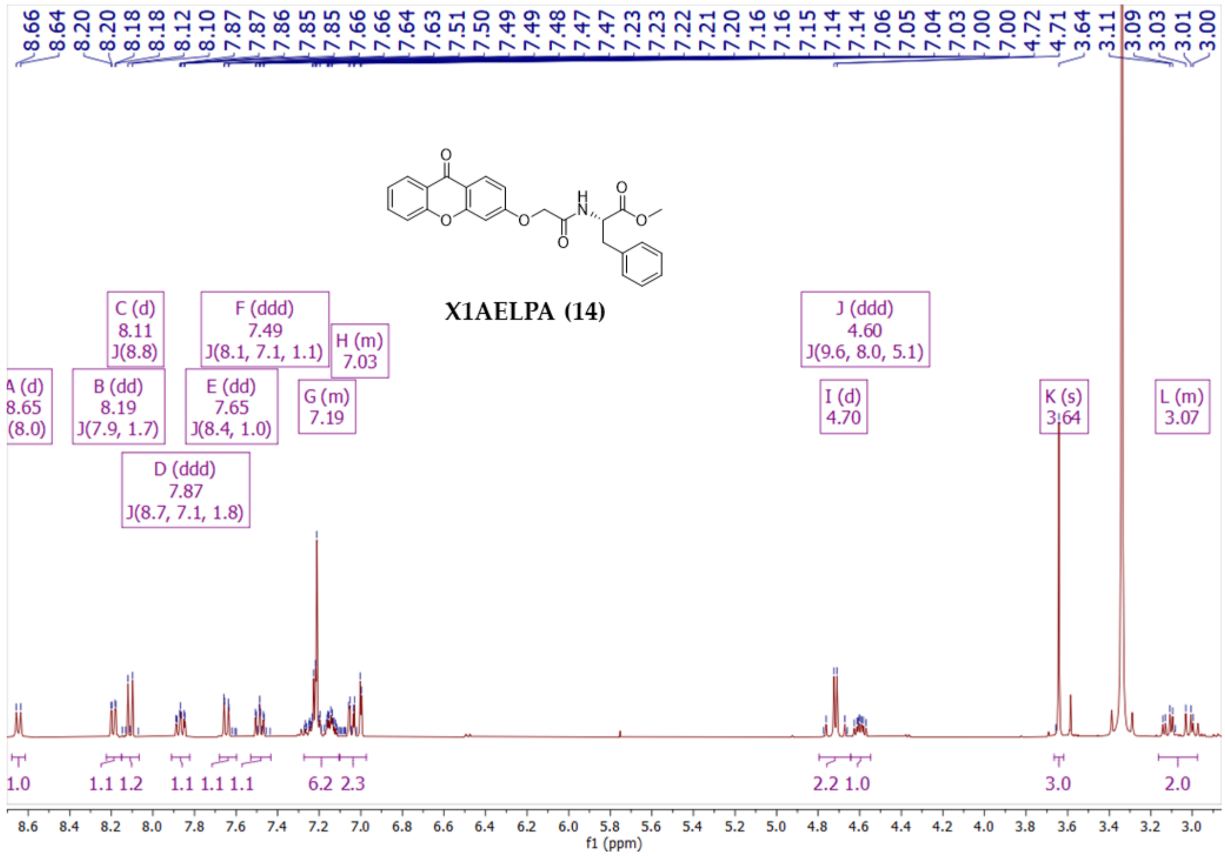
X1AELPA (14)
A (d) 8.65 J(8.0)
B (dd) 8.19 J(7.9, 1.7)
C (d) 8.11 J(8.8)
D (ddd) 7.87 J(8.7, 7.1, 1.8)
E (dd) 7.65 J(8.4, 1.0)
F (ddd) 7.49 J(8.1, 7.1, 1.1)
G (m) 7.19
H (m) 7.03
I (d) 4.70
J (ddd) 4.60 J(9.6, 8.0, 5.1)
K (s) 3.64
L (m) 3.07
f1 (ppm)

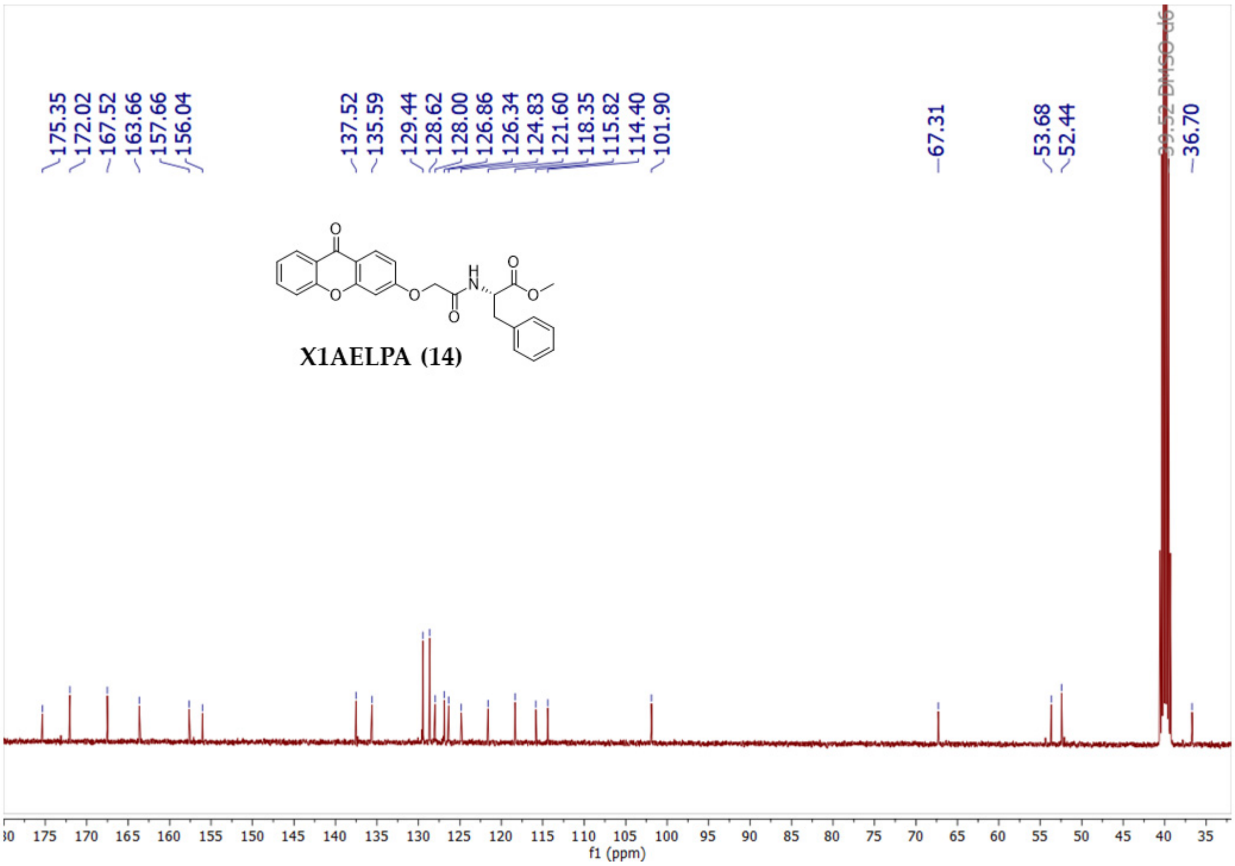
X1AELPA (14)
175.35
172.02
167.52
163.66
157.66
156.04
137.52
135.59
129.44
128.62
128.00
126.86
126.34
124.83
121.60
118.35
115.82
114.40
101.90
67.31
53.68
52.44
36.70
f1 (ppm)

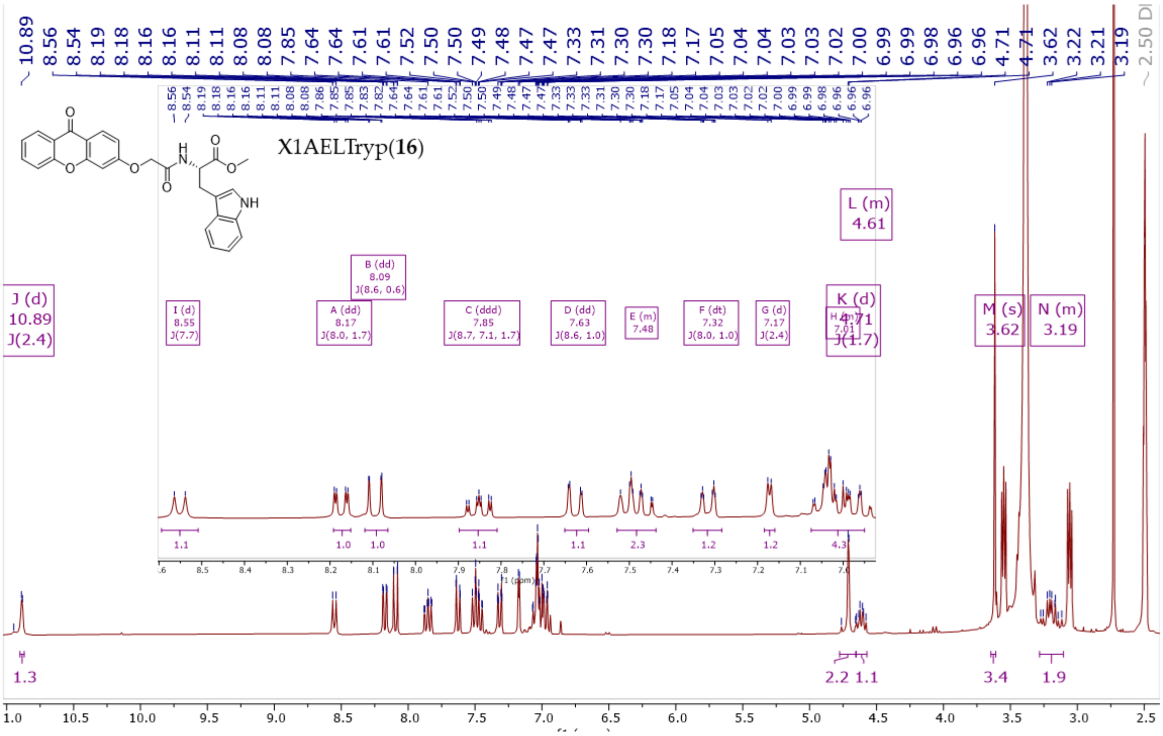
10.89 8.56 8.54 8.19 8.18 8.16 8.16 8.11 8.11 8.08 8.08 7.85 7.64 7.61 7.61 7.52 7.50 7.50 7.49 7.48 7.47 7.47 7.33 7.31 7.30 7.30 7.18 7.17 7.05 7.04 7.04 7.03 7.03 7.02 7.00 6.99 6.99 6.98 6.96 6.96 4.71 4.71 3.62 3.22 3.21 3.19 2.50
X1AELTryp(16)
L (m) 4.61
B (dd) 8.09 J(8.6, 0.6)
J (d) 10.89 J(2.4)
I (d) 8.55 J(7.7)
A (dd) 8.17 J(8.0, 1.7)
C (ddd) 7.85 J(8.7, 7.1, 1.7)
D (dd) 7.63 J(8.6, 1.0)
E (m) 7.48
F (dt) 7.32 J(8.0, 1.0)
G (d) 7.17 J(2.4)
K (d) 4.71 J(1.7)
M (s) 3.62
N (m) 3.19
1.3
2.2 1.1
3.4
1.9

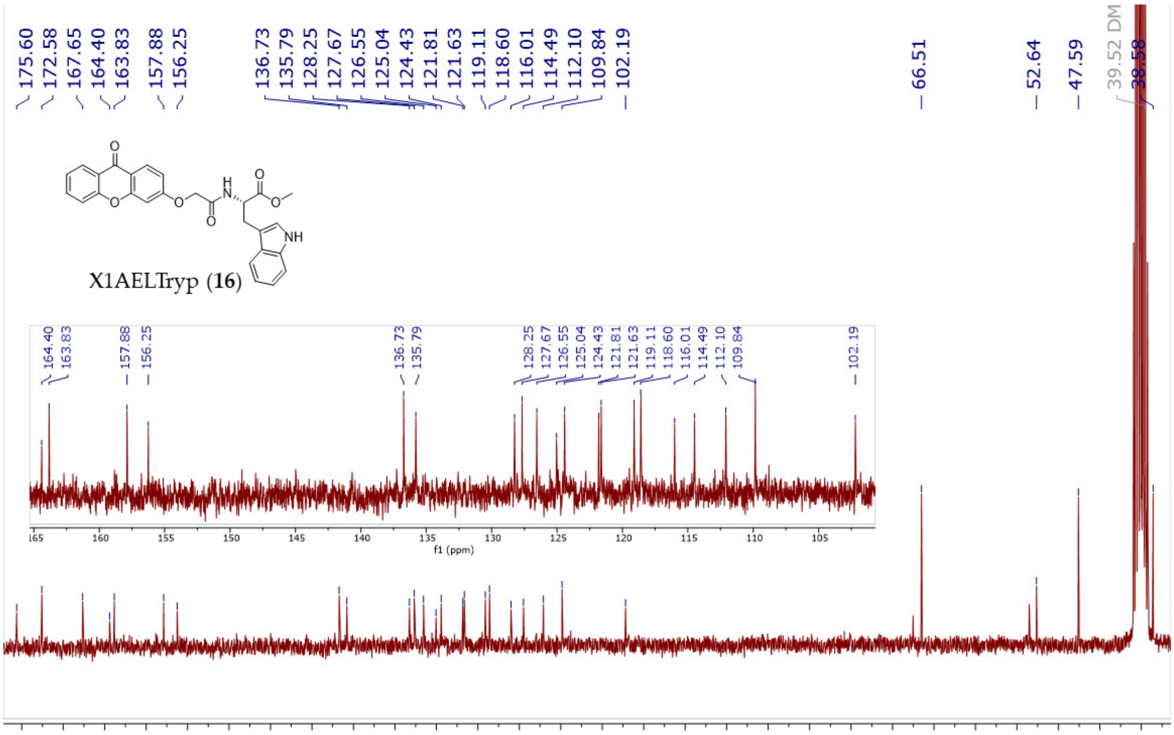
175.60 172.58 167.65 164.40 163.83 157.88 156.25 136.73 135.79 128.25 127.67 126.55 125.04 124.43 121.81 121.63 119.11 118.60 116.01 114.49 112.10 109.84 102.19 66.51 52.64 47.59 39.52 38.58
X1AELTryp (16)
f1 (ppm)

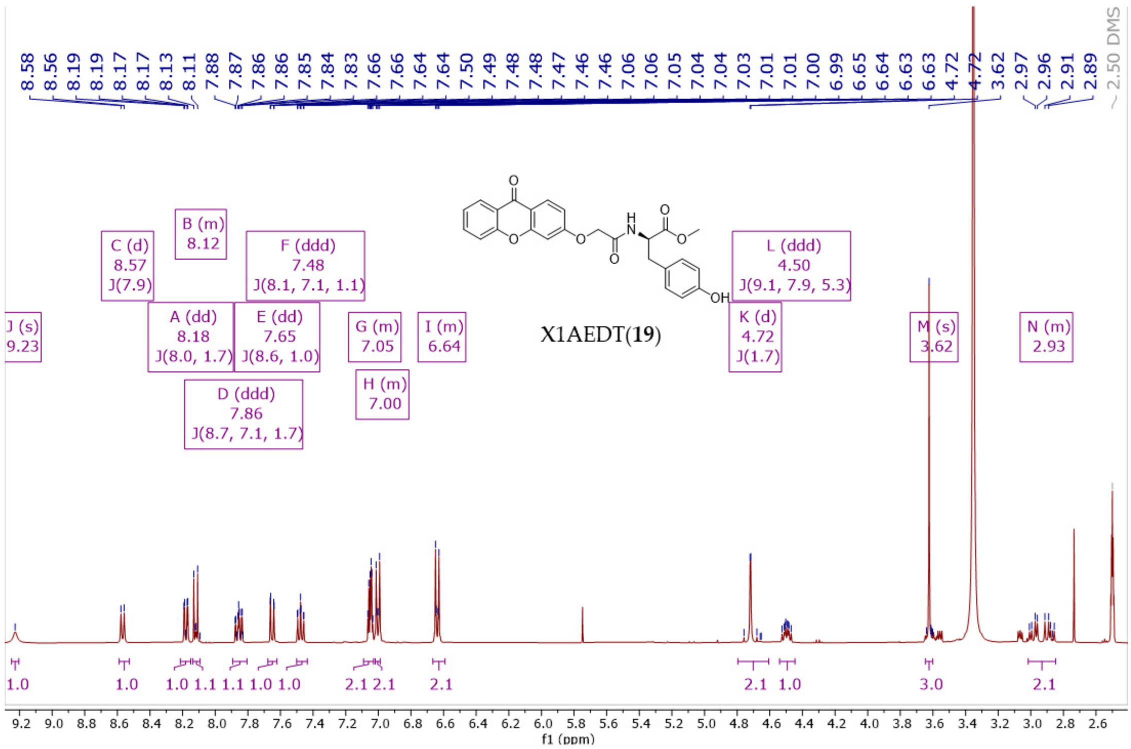
8.58 8.56 8.19 8.19 8.17 8.17 8.13 8.11 7.88 7.87 7.86 7.86 7.85 7.84 7.83 7.66 7.66 7.64 7.64 7.50 7.49 7.48 7.48 7.47 7.46 7.46 7.06 7.06 7.05 7.04 7.04 7.03 7.01 7.01 7.00 6.99 6.65 6.64 6.63 6.63 4.72 4.72 3.62 2.97 2.96 2.91 2.89 2.50 DMS
X1AEDT(19)
J (s) 9.23
C (d) 8.57 J(7.9)
B (m) 8.12
A (dd) 8.18 J(8.0, 1.7)
E (dd) 7.65 J(8.6, 1.0)
F (ddd) 7.48 J(8.1, 7.1, 1.1)
D (ddd) 7.86 J(8.7, 7.1, 1.7)
G (m) 7.05
H (m) 7.00
I (m) 6.64
L (ddd) 4.50 J(9.1, 7.9, 5.3)
K (d) 4.72 J(1.7)
M (s) 3.62
N (m) 2.93
1.0 1.0 1.0 1.1 1.1 1.0 1.0 2.1 2.1 2.1 2.1 1.0 3.0 2.1
f1 (ppm)

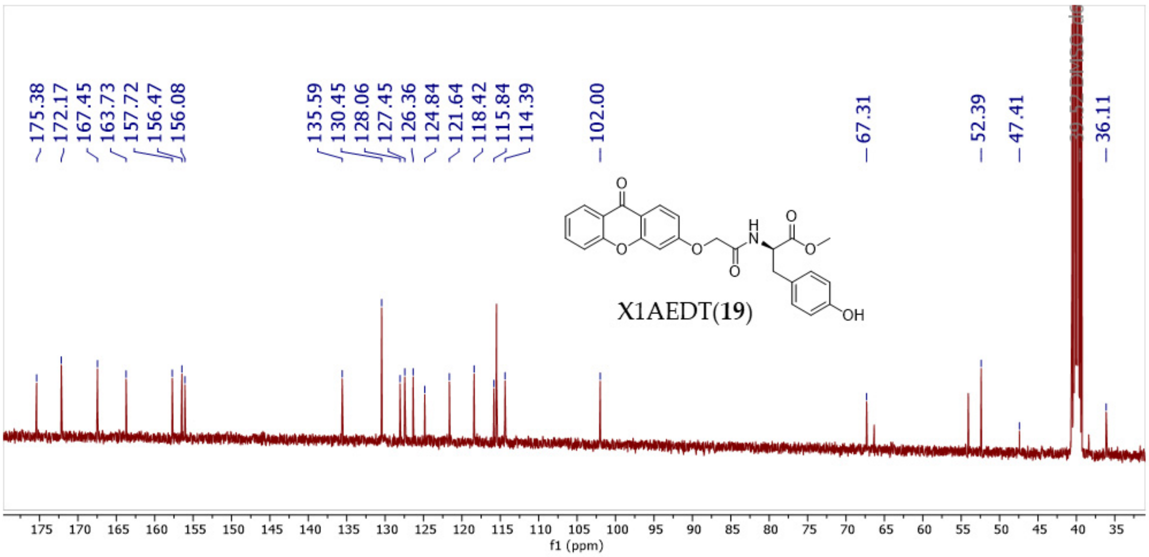
175.38 172.17 167.45 163.73 157.72 156.47 156.08 135.59 130.45 128.06 127.45 126.36 124.84 121.64 118.42 115.84 114.39 102.00 67.31 52.39 47.41 36.11
X1AEDT(19)
f1 (ppm)

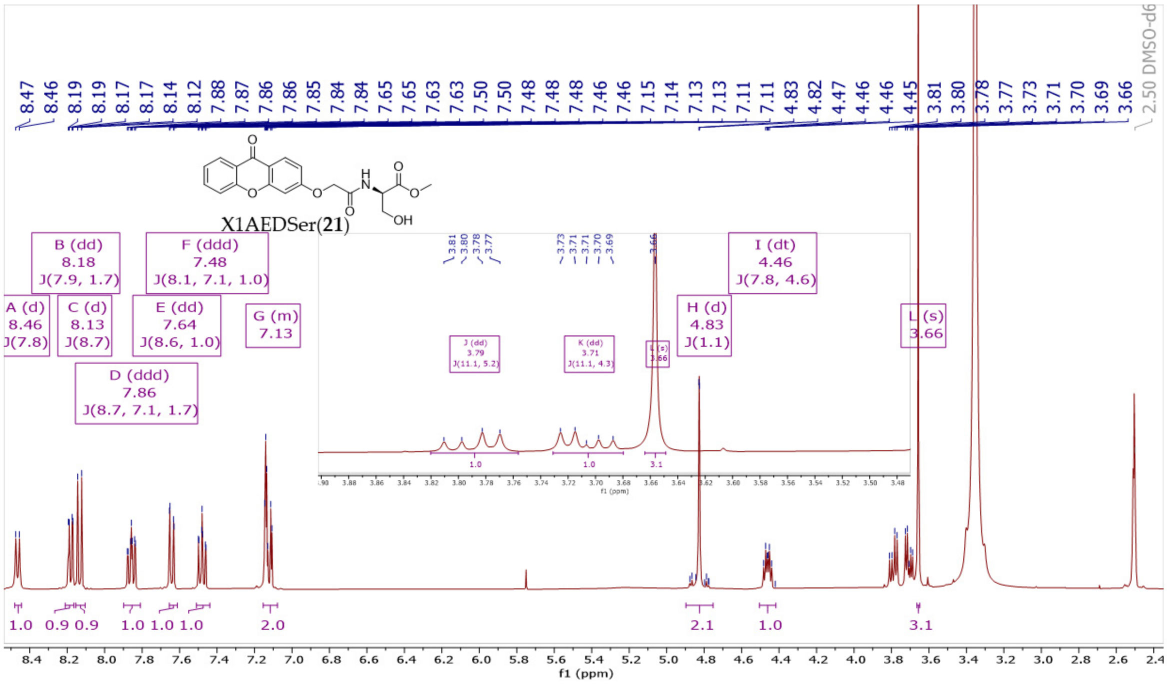
X1AEDSer(21)
A (d) 8.46 J(7.8)
B (dd) 8.18 J(7.9, 1.7)
C (d) 8.13 J(8.7)
D (ddd) 7.86 J(8.7, 7.1, 1.7)
E (dd) 7.64 J(8.6, 1.0)
F (ddd) 7.48 J(8.1, 7.1, 1.0)
G (m) 7.13
H (d) 4.83 J(1.1)
I (dt) 4.46 J(7.8, 4.6)
L (s) 3.66
f1 (ppm)

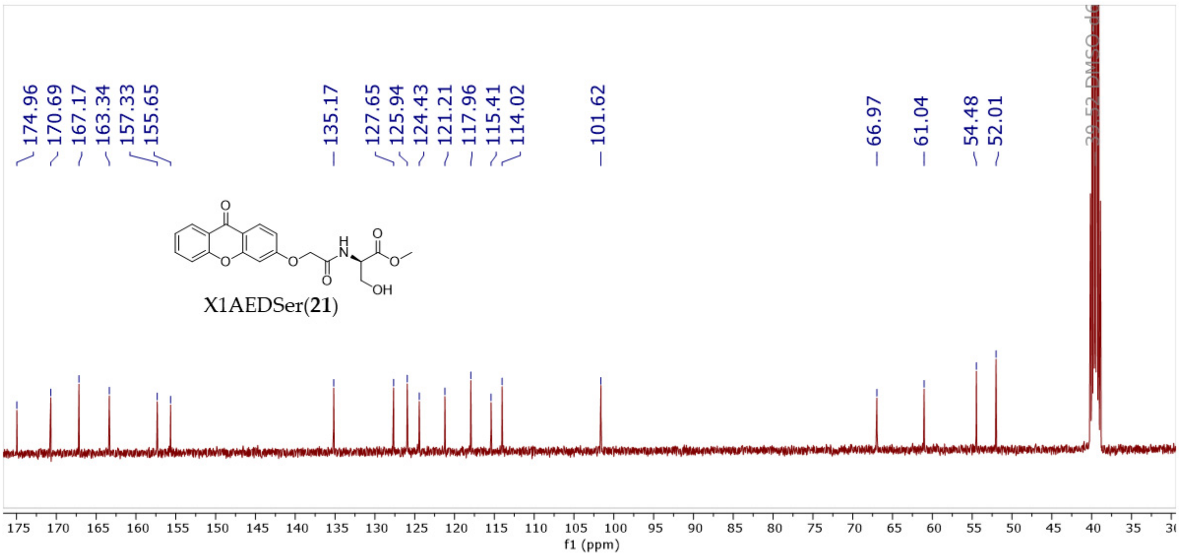
174.96
170.69
167.17
163.34
157.33
155.65
135.17
127.65
125.94
124.43
121.21
117.96
115.41
114.02
101.62
66.97
61.04
54.48
52.01
X1AEDSer(21)
f1 (ppm)

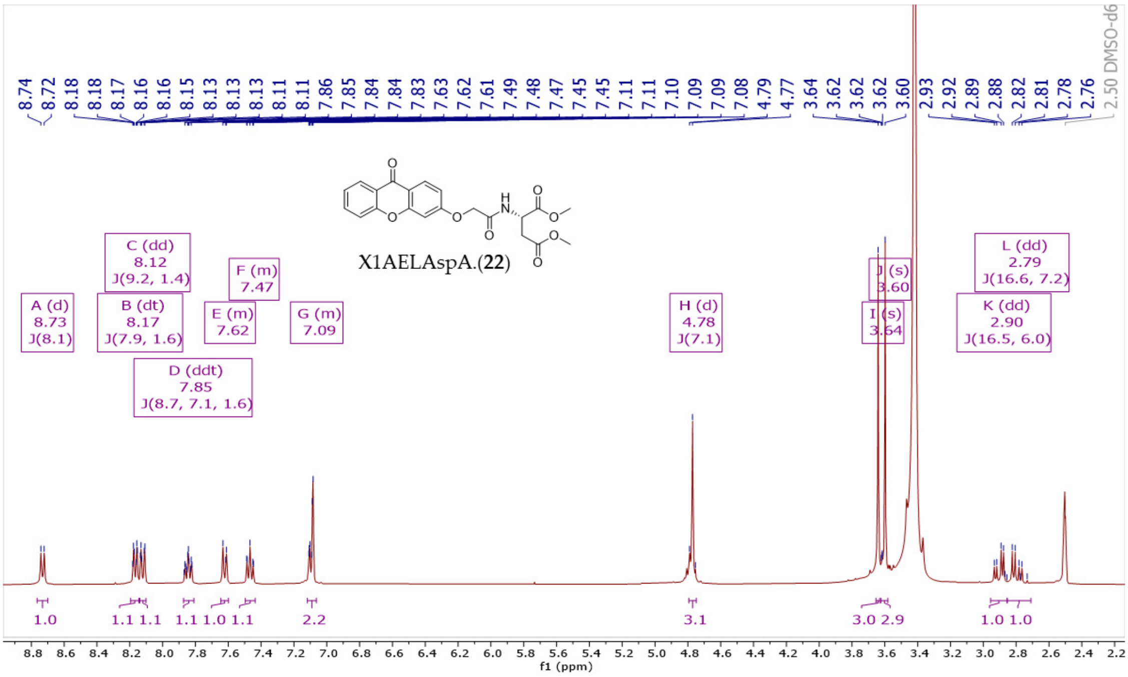
8.74 8.72 8.18 8.18 8.17 8.16 8.16 8.15 8.13 8.13 8.13 8.11 7.86 7.85 7.84 7.84 7.83 7.63 7.62 7.61 7.49 7.48 7.47 7.45 7.45 7.11 7.11 7.10 7.09 7.09 7.08 4.79 4.77 3.64 3.62 3.62 3.62 3.60 2.93 2.92 2.89 2.88 2.82 2.81 2.78 2.76
2.50 DMSO-d6
X1AELAspA.(22)
A (d) 8.73 J(8.1)
B (dt) 8.17 J(7.9, 1.6)
C (dd) 8.12 J(9.2, 1.4)
D (ddt) 7.85 J(8.7, 7.1, 1.6)
E (m) 7.62
F (m) 7.47
G (m) 7.09
H (d) 4.78 J(7.1)
I (s) 3.64
J (s) 3.60
K (dd) 2.90 J(16.5, 6.0)
L (dd) 2.79 J(16.6, 7.2)
1.0 1.1 1.1 1.1 1.0 1.1 2.2 3.1 3.0 2.9 1.0 1.0
f1 (ppm)

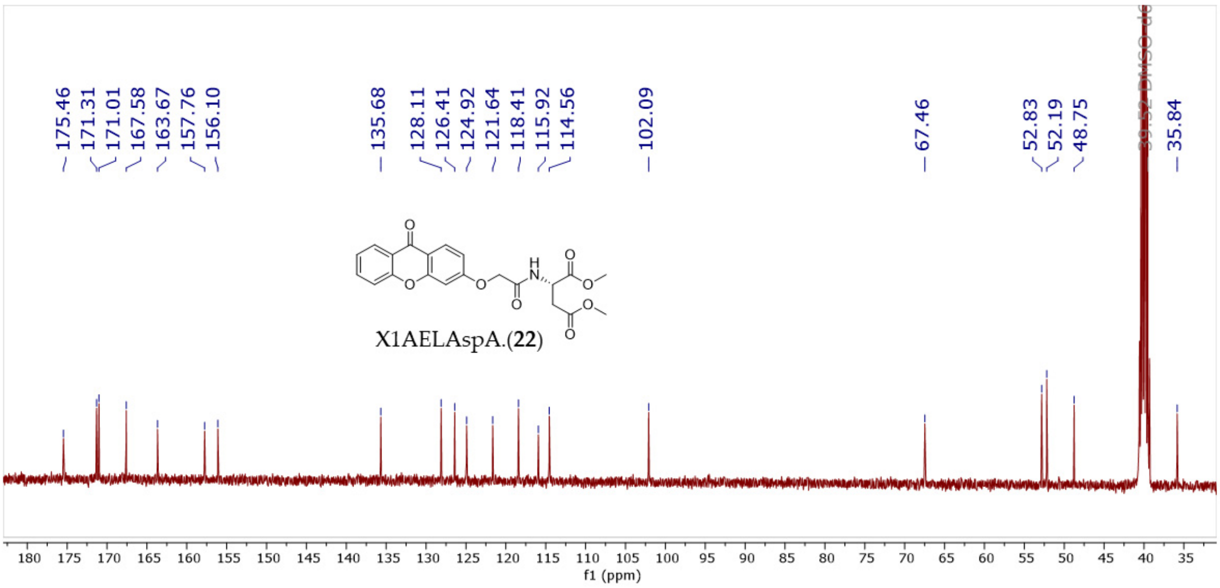
175.46 171.31 171.01 167.58 163.67 157.76 156.10 135.68 128.11 126.41 124.92 121.64 118.41 115.92 114.56 102.09 67.46 52.83 52.19 48.75 35.84
X1AELAspA.(22)
f1 (ppm)

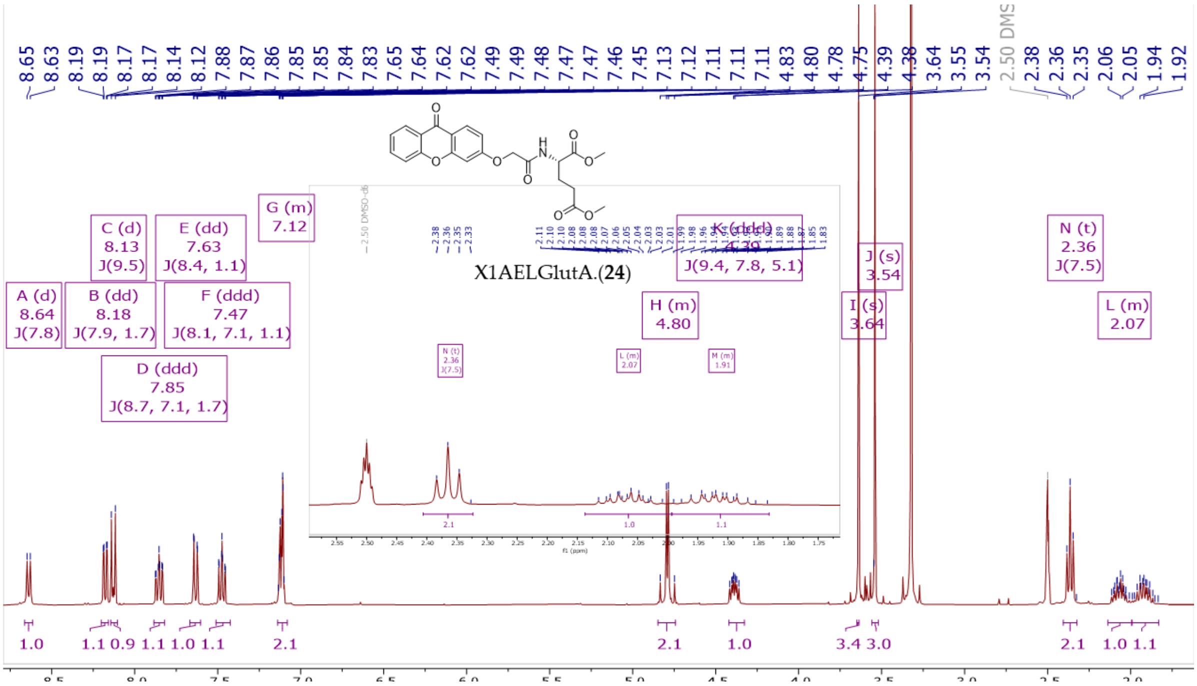
X1AELGlutA.(**24**)

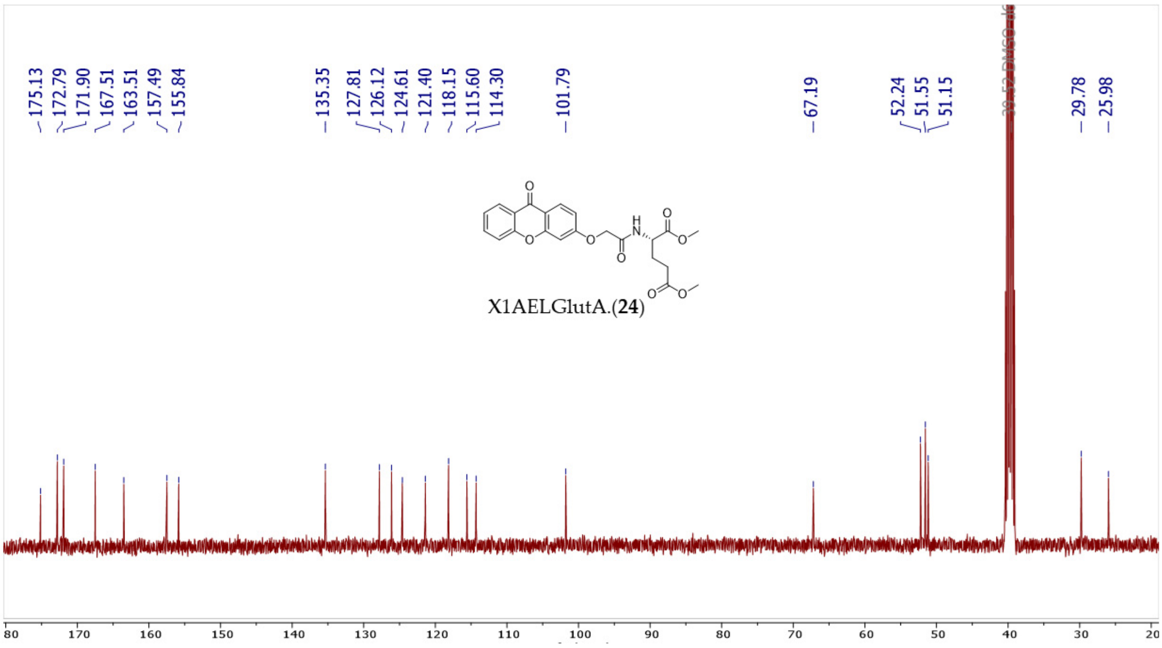
X1AELGlutA.(**24**)

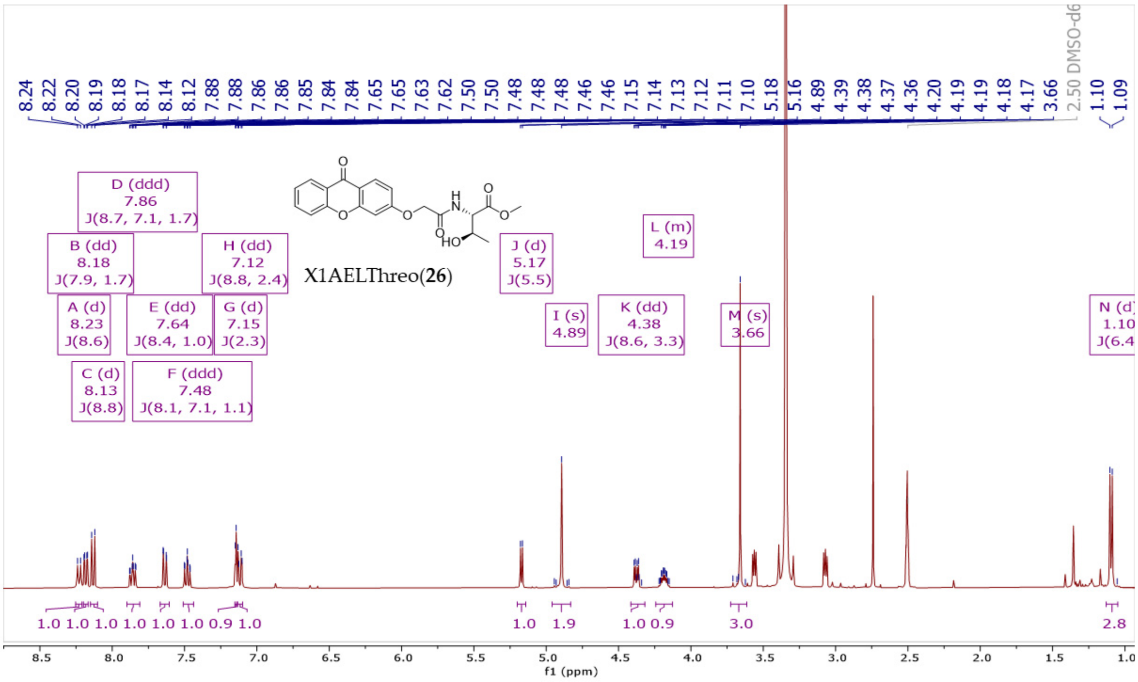
8.24 8.22 8.20 8.19 8.18 8.17 8.14 8.12 7.88 7.88 7.86 7.86 7.85 7.84 7.84 7.65 7.65 7.63 7.62 7.50 7.50 7.48 7.48 7.48 7.46 7.46 7.15 7.14 7.13 7.12 7.11 7.10 5.18 5.16 4.89 4.39 4.38 4.37 4.36 4.20 4.19 4.19 4.18 4.17 3.66
2.50 DMSO-d6
1.10 1.09
D (ddd) 7.86 J(8.7, 7.1, 1.7)
B (dd) 8.18 J(7.9, 1.7)
H (dd) 7.12 J(8.8, 2.4)
A (d) 8.23 J(8.6)
E (dd) 7.64 J(8.4, 1.0)
G (d) 7.15 J(2.3)
C (d) 8.13 J(8.8)
F (ddd) 7.48 J(8.1, 7.1, 1.1)
J (d) 5.17 J(5.5)
I (s) 4.89
L (m) 4.19
K (dd) 4.38 J(8.6, 3.3)
M (s) 3.66
N (d) 1.10 J(6.4
X1AELThreo(26)
1.0 1.0 1.0 1.0 1.0 1.0 0.9 1.0 1.0 1.9 1.0 0.9 3.0 2.8
f1 (ppm)

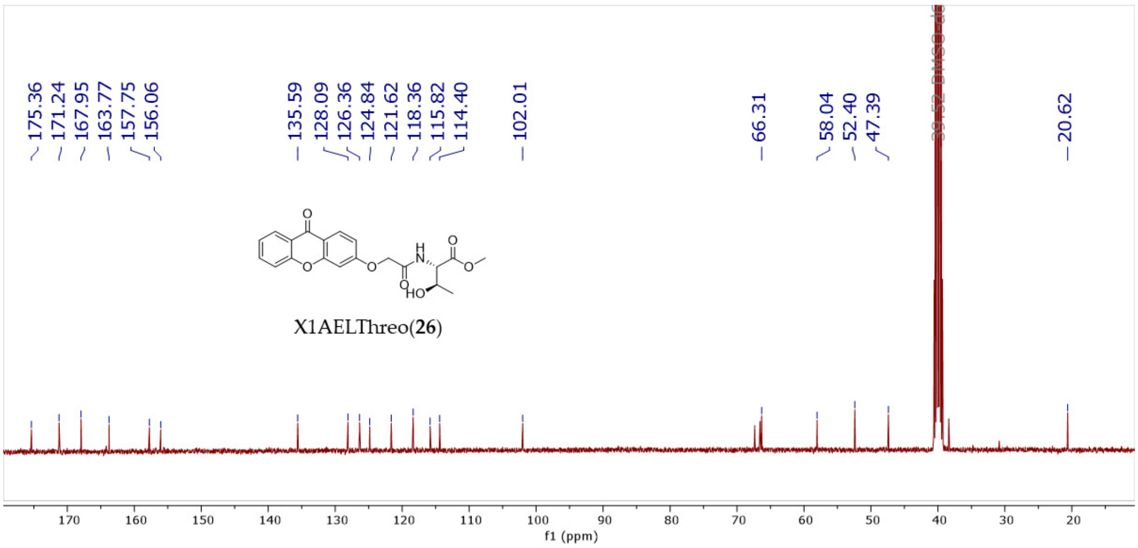
175.36 171.24 167.95 163.77 157.75 156.06 135.59 128.09 126.36 124.84 121.62 118.36 115.82 114.40 102.01 66.31 58.04 52.40 47.39 20.62
X1AELThreo(26)
f1 (ppm)

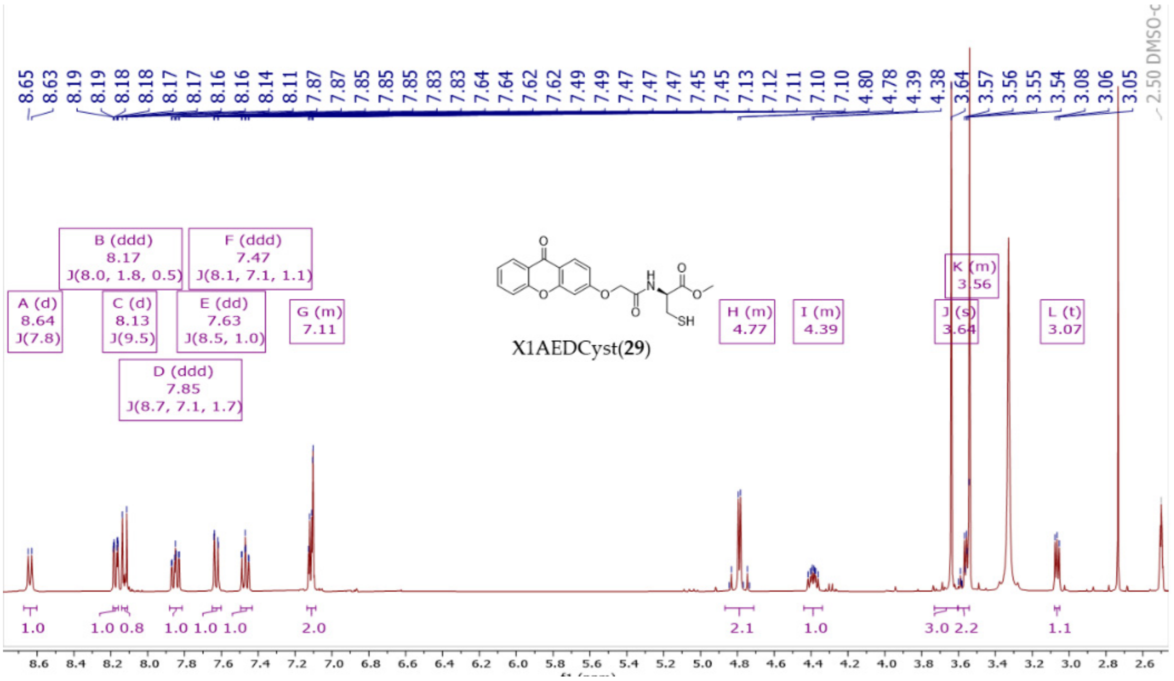
8.65 8.63 8.19 8.19 8.18 8.18 8.17 8.17 8.16 8.16 8.14 8.11 7.87 7.87 7.85 7.85 7.85 7.83 7.83 7.64 7.64 7.62 7.62 7.49 7.49 7.47 7.47 7.47 7.45 7.45 7.13 7.12 7.11 7.10 7.10 4.80 4.78 4.39 4.38 3.64 3.57 3.56 3.55 3.54 3.08 3.06 3.05
2.50 DMSO-d
A (d) 8.64 J(7.8)
B (ddd) 8.17 J(8.0, 1.8, 0.5)
C (d) 8.13 J(9.5)
D (ddd) 7.85 J(8.7, 7.1, 1.7)
E (dd) 7.63 J(8.5, 1.0)
F (ddd) 7.47 J(8.1, 7.1, 1.1)
G (m) 7.11
H (m) 4.77
I (m) 4.39
J (s) 3.64
K (m) 3.56
L (t) 3.07
X1AEDCyst(29)
1.0 1.0 0.8 1.0 1.0 1.0 2.0 2.1 1.0 3.0 2.2 1.1
8.6 8.4 8.2 8.0 7.8 7.6 7.4 7.2 7.0 6.8 6.6 6.4 6.2 6.0 5.8 5.6 5.4 5.2 5.0 4.8 4.6 4.4 4.2 4.0 3.8 3.6 3.4 3.2 3.0 2.8 2.6

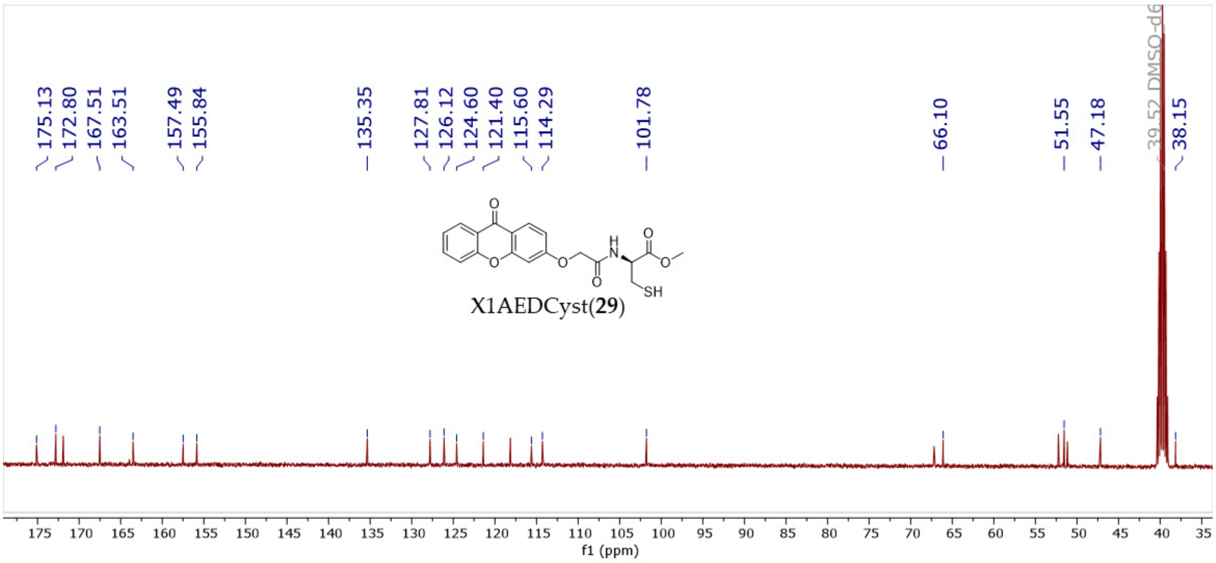
175.13 172.80 167.51 163.51 157.49 155.84 135.35 127.81 126.12 124.60 121.40 115.60 114.29 101.78 66.10 51.55 47.18 39.52 DMSO-d6 38.15
X1AEDCyst(29)
175 170 165 160 155 150 145 140 135 130 125 120 115 110 105 100 95 90 85 80 75 70 65 60 55 50 45 40 35
f1 (ppm)

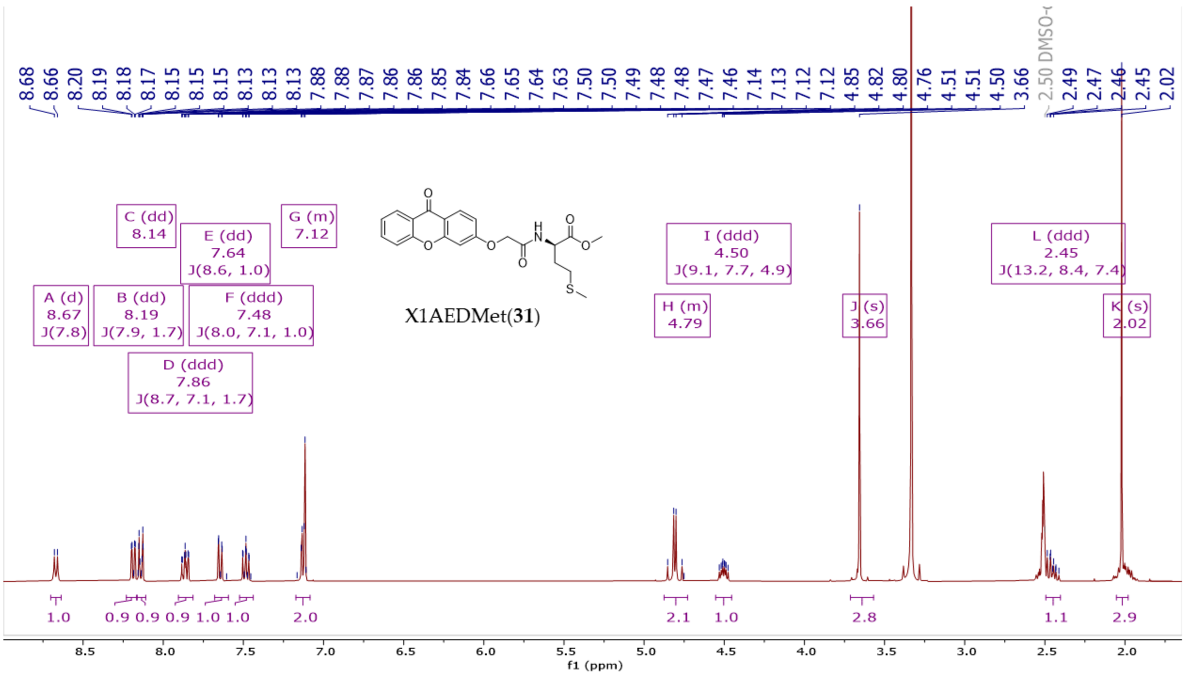
X1AEDMet(31)
A (d) 8.67 J(7.8)
B (dd) 8.19 J(7.9, 1.7)
C (dd) 8.14
D (ddd) 7.86 J(8.7, 7.1, 1.7)
E (dd) 7.64 J(8.6, 1.0)
F (ddd) 7.48 J(8.0, 7.1, 1.0)
G (m) 7.12
H (m) 4.79
I (ddd) 4.50 J(9.1, 7.7, 4.9)
J (s) 3.66
K (s) 2.02
L (ddd) 2.45 J(13.2, 8.4, 7.4)
2.50 DMSO-d
1.0 0.9 0.9 0.9 1.0 1.0 2.0 2.1 1.0 2.8 1.1 2.9
f1 (ppm)

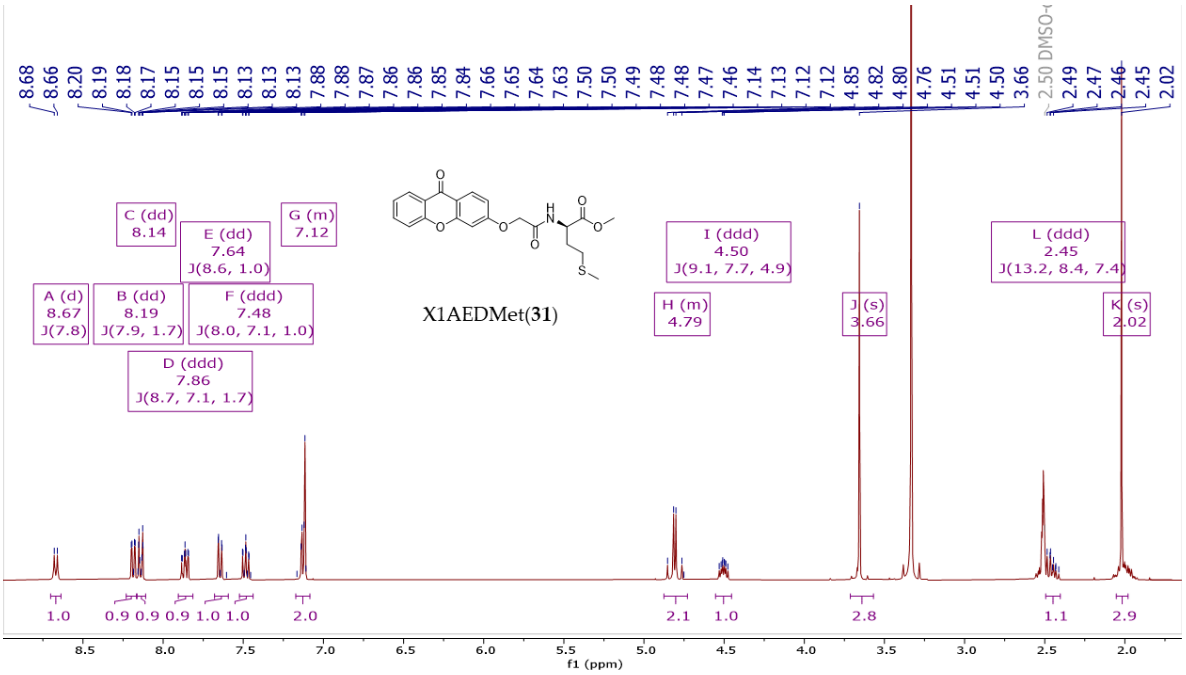
X1AEDMet(31)
A (d) 8.67 J(7.8)
B (dd) 8.19 J(7.9, 1.7)
C (dd) 8.14
D (ddd) 7.86 J(8.7, 7.1, 1.7)
E (dd) 7.64 J(8.6, 1.0)
F (ddd) 7.48 J(8.0, 7.1, 1.0)
G (m) 7.12
H (m) 4.79
I (ddd) 4.50 J(9.1, 7.7, 4.9)
J (s) 3.66
K (s) 2.02
L (ddd) 2.45 J(13.2, 8.4, 7.4)
2.50 DMSO-d
1.0 0.9 0.9 0.9 1.0 1.0 2.0 2.1 1.0 2.8 1.1 2.9
f1 (ppm)

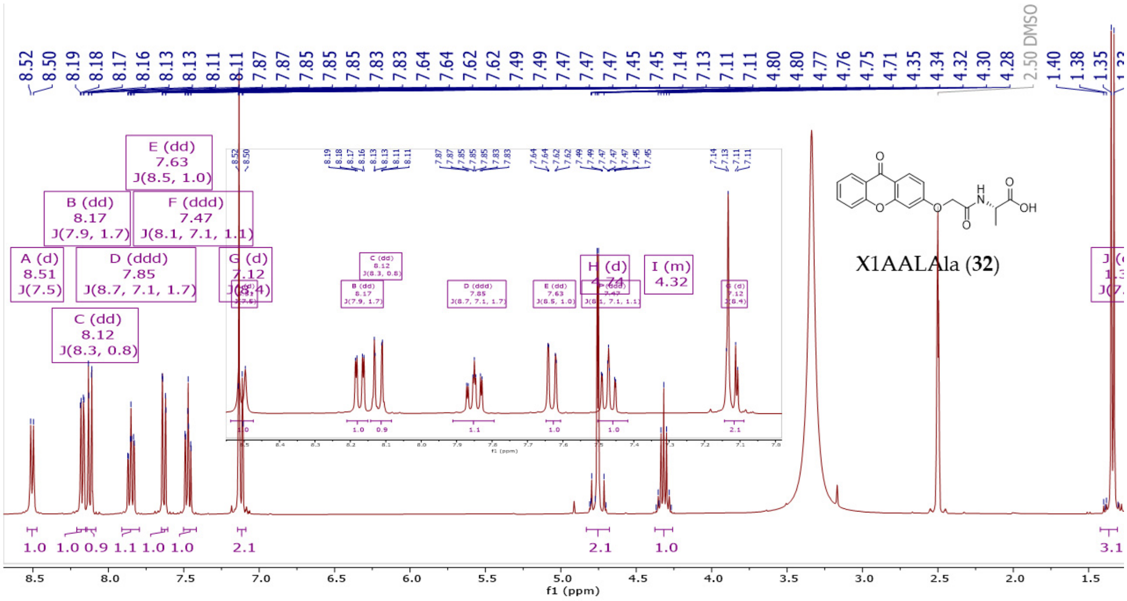
X1AALAla (32)
A (d) 8.51 J(7.5)
B (dd) 8.17 J(7.9, 1.7)
C (dd) 8.12 J(8.3, 0.8)
D (ddd) 7.85 J(8.7, 7.1, 1.7)
E (dd) 7.63 J(8.5, 1.0)
F (ddd) 7.47 J(8.1, 7.1, 1.1)
G (d) 7.12
H (d) 4.74
I (m) 4.32
2.50 DMSO
f1 (ppm)

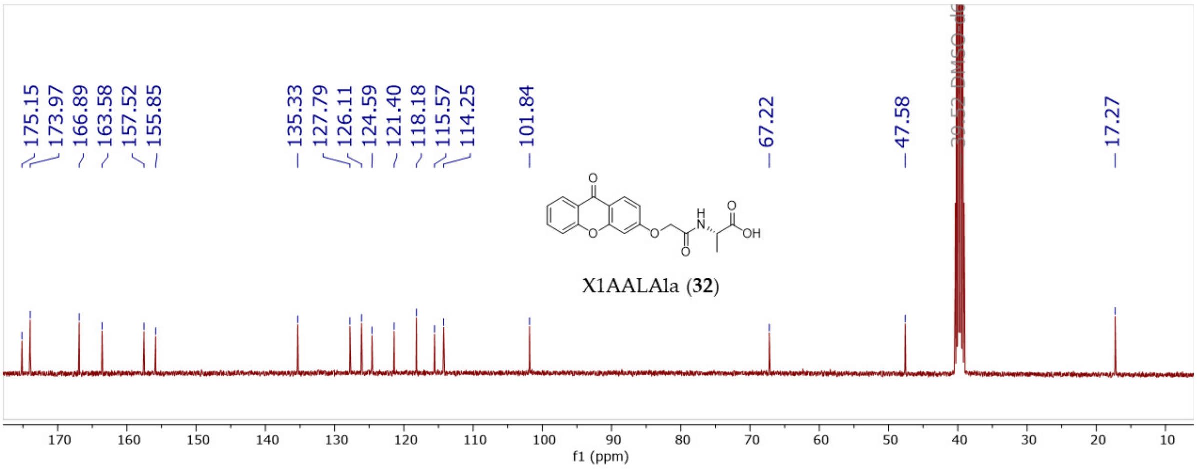
175.15
173.97
166.89
163.58
157.52
155.85
135.33
127.79
126.11
124.59
121.40
118.18
115.57
114.25
101.84
67.22
47.58
17.27
X1AALAla (32)
f1 (ppm)

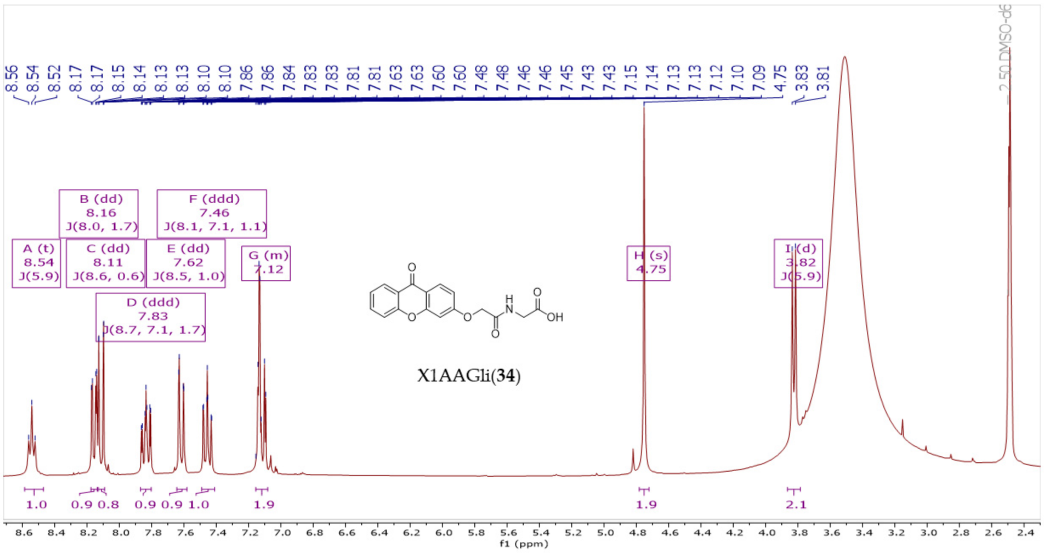
8.56 8.54 8.52 8.17 8.17 8.15 8.14 8.13 8.13 8.10 8.10 7.86 7.86 7.84 7.83 7.83 7.81 7.81 7.63 7.63 7.60 7.60 7.48 7.48 7.46 7.46 7.45 7.43 7.43 7.15 7.14 7.13 7.13 7.12 7.10 7.09 4.75 3.83 3.81
2.50 DMSO-d6
A (t) 8.54 J(5.9)
B (dd) 8.16 J(8.0, 1.7)
C (dd) 8.11 J(8.6, 0.6)
D (ddd) 7.83 J(8.7, 7.1, 1.7)
E (dd) 7.62 J(8.5, 1.0)
F (ddd) 7.46 J(8.1, 7.1, 1.1)
G (m) 7.12
H (s) 4.75
I (d) 3.82 J(5.9)
X1AAGli(34)
1.0 0.9 0.8 0.9 0.9 1.0 1.9 1.9 2.1
f1 (ppm)

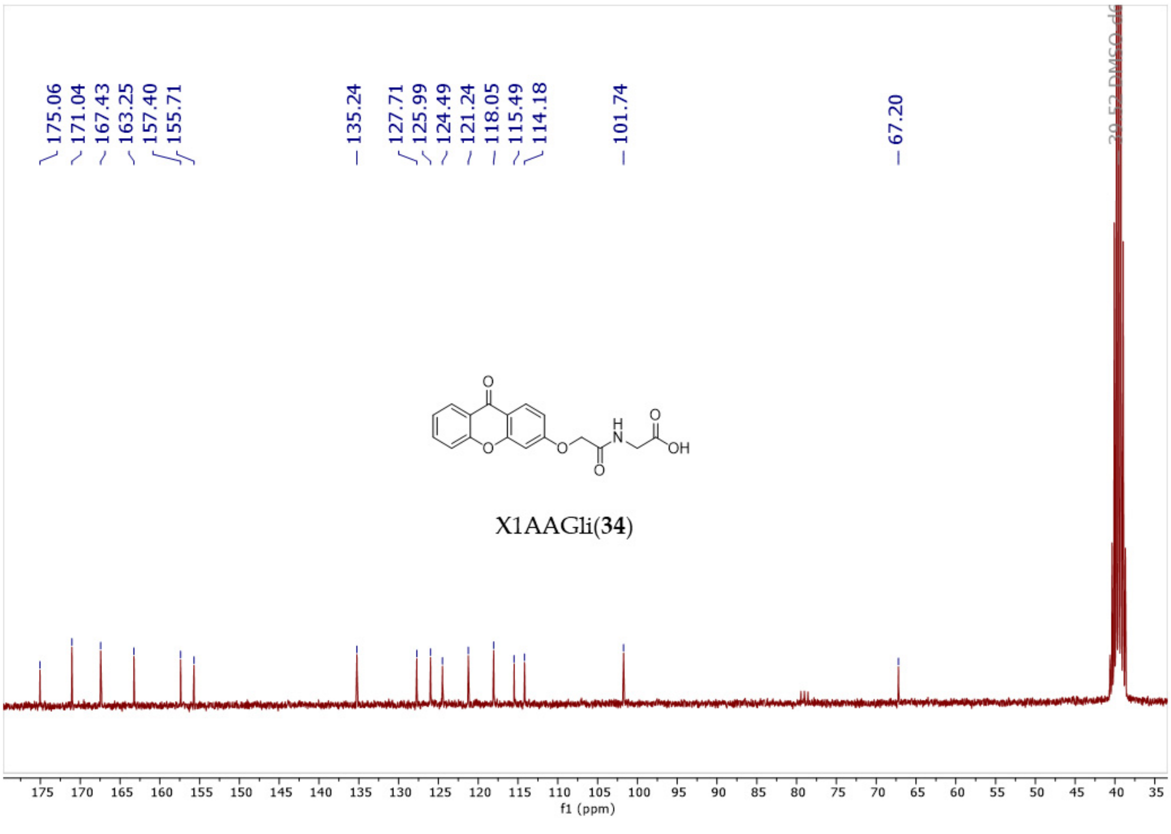
175.06 171.04 167.43 163.25 157.40 155.71 135.24 127.71 125.99 124.49 121.24 118.05 115.49 114.18 101.74 67.20
X1AAGli(34)
f1 (ppm)

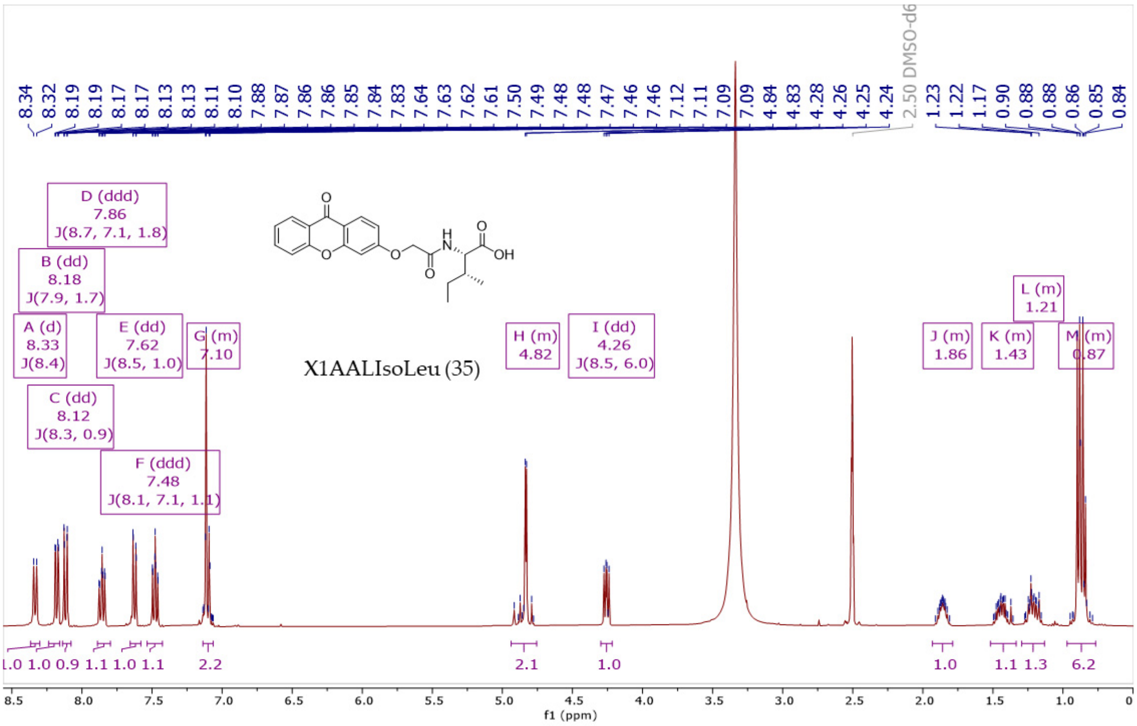

X1AALIsoLeu (35)
A (d) 8.33 J(8.4)
B (dd) 8.18 J(7.9, 1.7)
C (dd) 8.12 J(8.3, 0.9)
D (ddd) 7.86 J(8.7, 7.1, 1.8)
E (dd) 7.62 J(8.5, 1.0)
F (ddd) 7.48 J(8.1, 7.1, 1.1)
G (m) 7.10
H (m) 4.82
I (dd) 4.26 J(8.5, 6.0)
J (m) 1.86
K (m) 1.43
L (m) 1.21
M (m) 0.87
2.50 DMSO-d6
f1 (ppm)

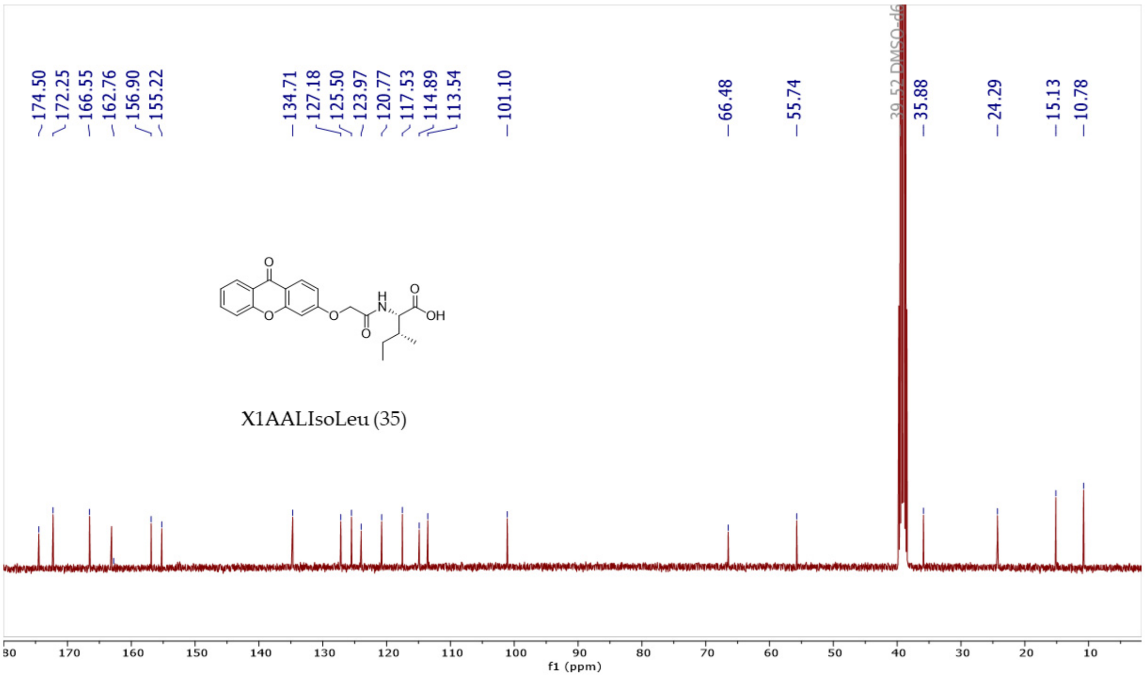

174.50
172.25
166.55
162.76
156.90
155.22
134.71
127.18
125.50
123.97
120.77
117.53
114.89
113.54
101.10
66.48
55.74
35.88
24.29
15.13
10.78
X1AALIsoLeu (35)
f1 (ppm)

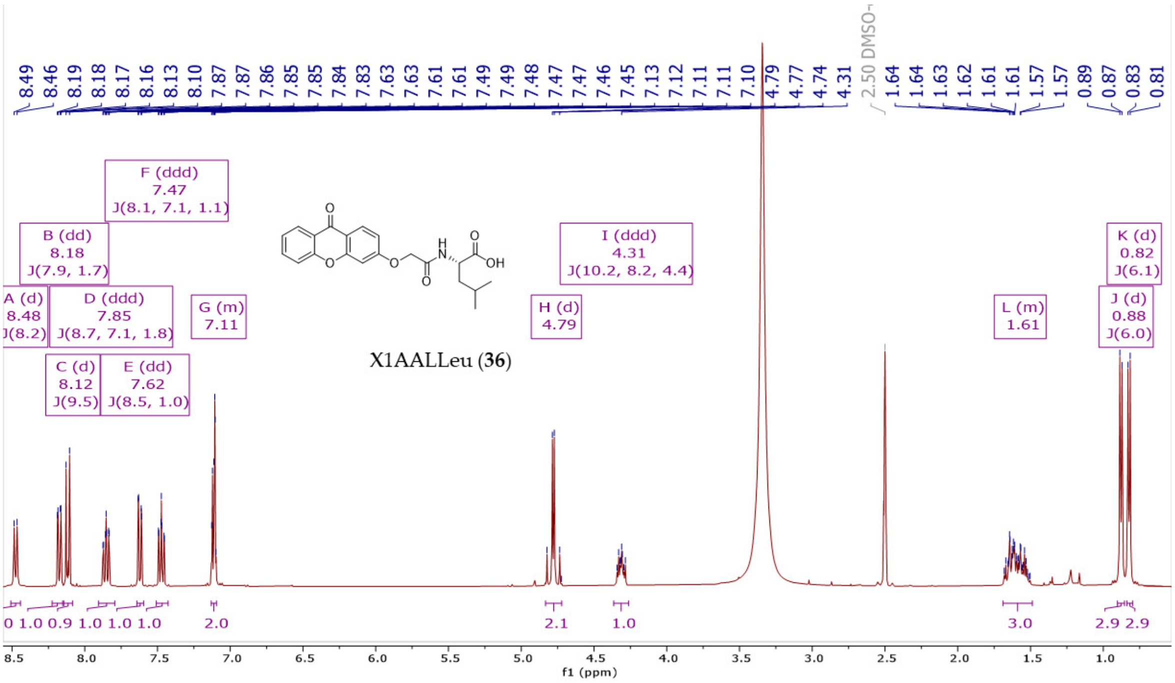
X1AALLeu (36)
A (d) 8.48 J(8.2)
B (dd) 8.18 J(7.9, 1.7)
C (d) 8.12 J(9.5)
D (ddd) 7.85 J(8.7, 7.1, 1.8)
E (dd) 7.62 J(8.5, 1.0)
F (ddd) 7.47 J(8.1, 7.1, 1.1)
G (m) 7.11
H (d) 4.79
I (ddd) 4.31 J(10.2, 8.2, 4.4)
J (d) 0.88 J(6.0)
K (d) 0.82 J(6.1)
L (m) 1.61
2.50 DMSO-
f1 (ppm)

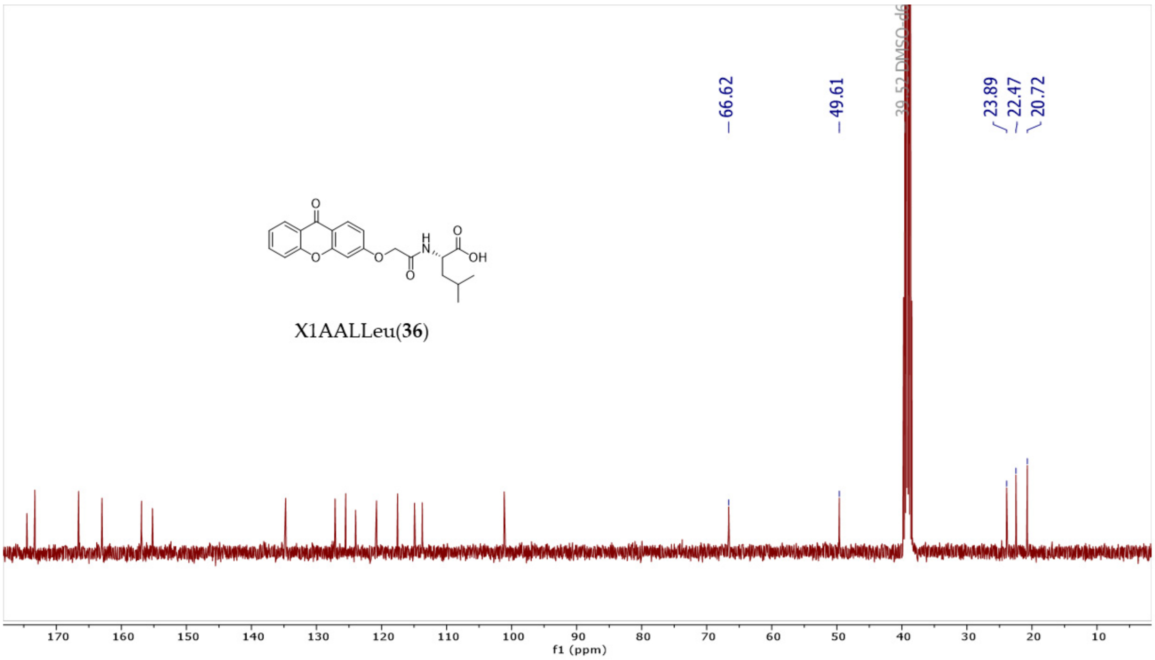
X1AALLeu(36)
66.62
49.61
39.52 DMSO-d6
23.89
22.47
20.72
f1 (ppm)

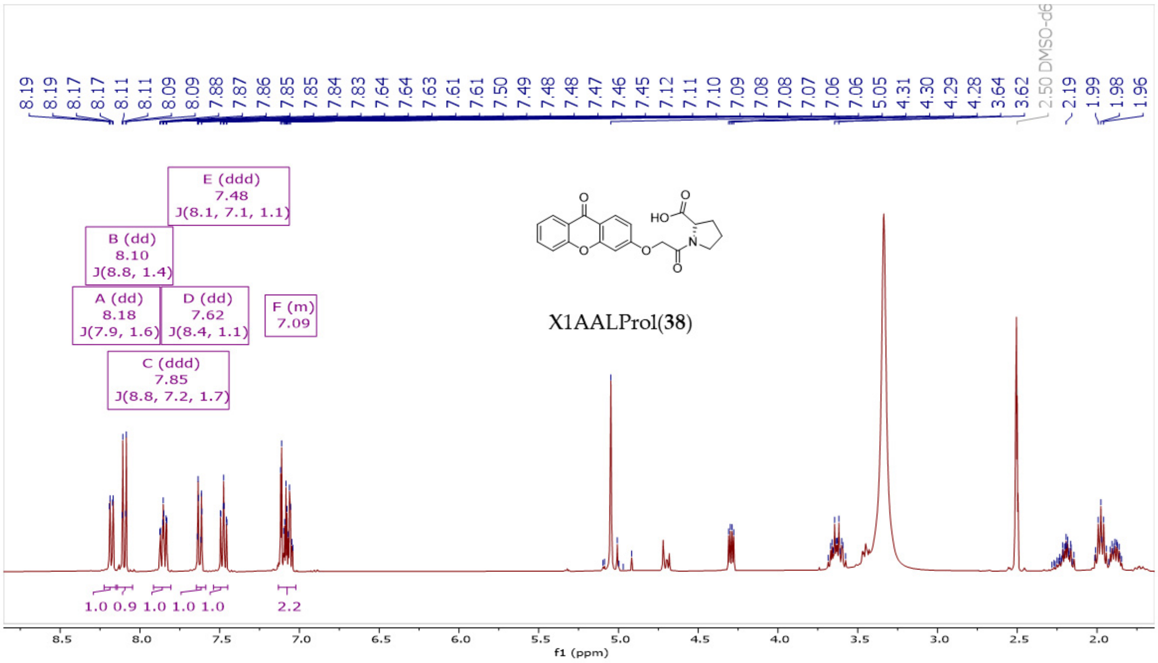
8.19
8.19
8.17
8.17
8.11
8.11
8.09
8.09
7.88
7.87
7.86
7.85
7.85
7.84
7.83
7.64
7.64
7.63
7.61
7.61
7.50
7.49
7.48
7.48
7.47
7.46
7.45
7.12
7.11
7.10
7.09
7.08
7.08
7.07
7.06
7.06
5.05
4.31
4.30
4.29
4.28
3.64
3.62
2.50 DMSO-d6
2.19
1.99
1.98
1.96
E (ddd) 7.48 J(8.1, 7.1, 1.1)
B (dd) 8.10 J(8.8, 1.4)
A (dd) 8.18 J(7.9, 1.6)
D (dd) 7.62 J(8.4, 1.1)
F (m) 7.09
C (ddd) 7.85 J(8.8, 7.2, 1.7)
X1AALProl(38)
1.0 0.9 1.0 1.0 1.0
2.2
f1 (ppm)

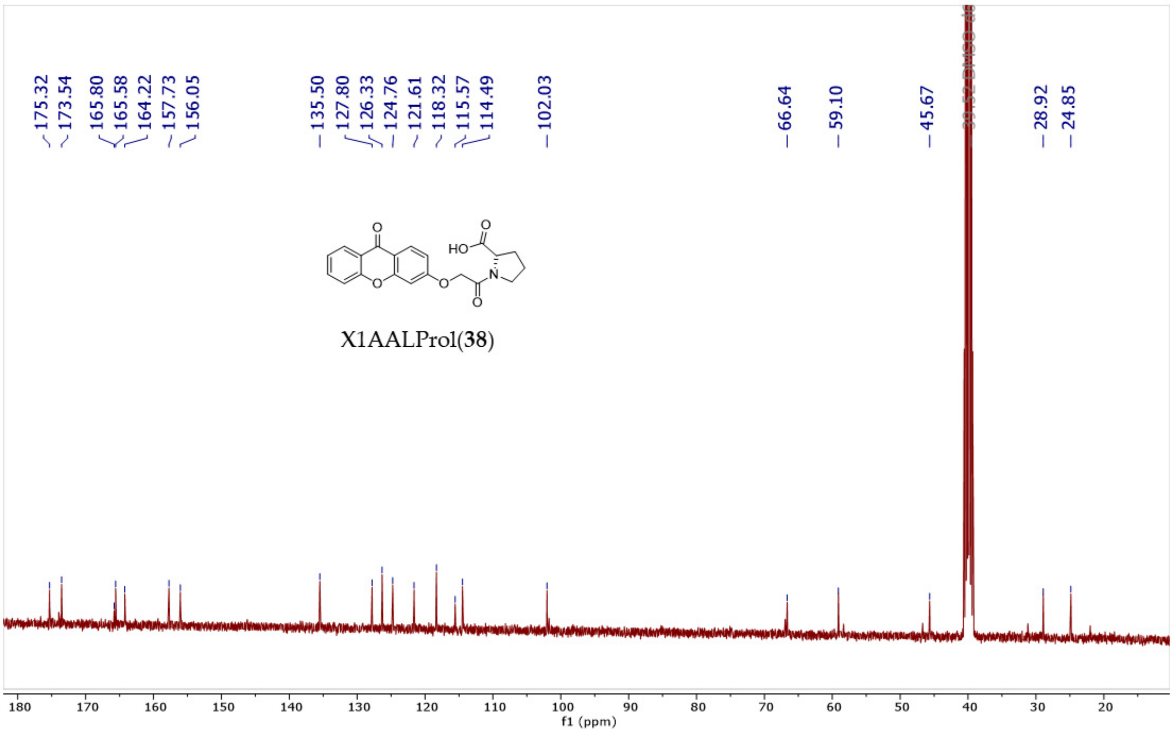
175.32
173.54
165.80
165.58
164.22
157.73
156.05
135.50
127.80
126.33
124.76
121.61
118.32
115.57
114.49
102.03
66.64
59.10
45.67
28.92
24.85
X1AALProl(38)
f1 (ppm)

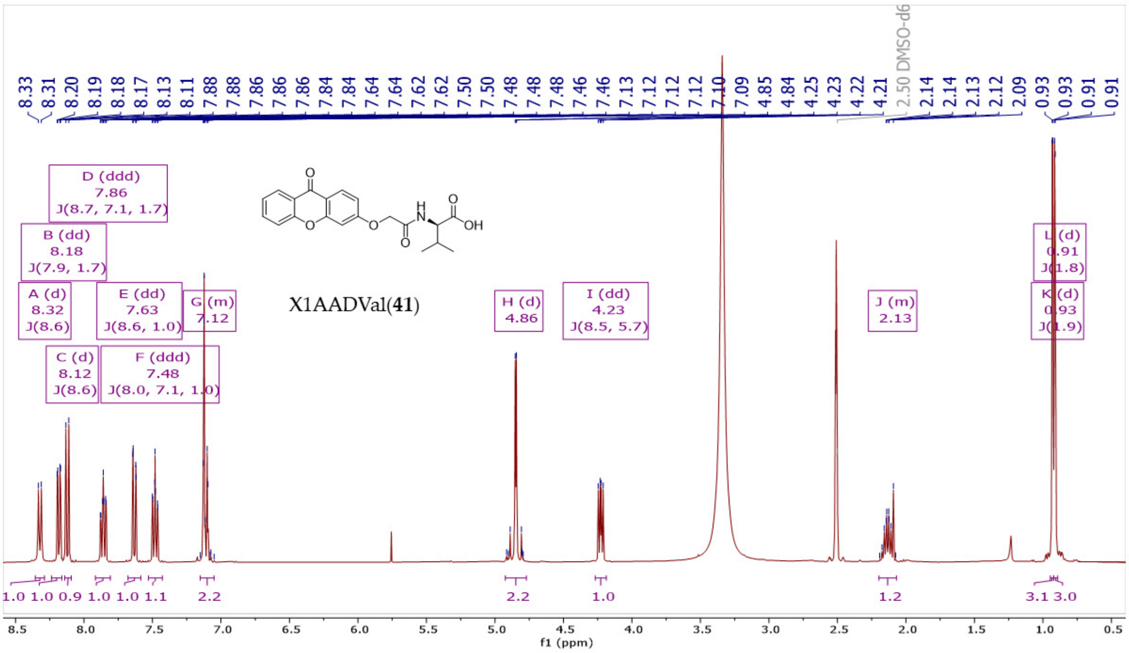
8.33 8.31 8.20 8.19 8.18 8.17 8.13 8.11 7.88 7.88 7.86 7.86 7.86 7.84 7.84 7.64 7.64 7.62 7.62 7.50 7.50 7.48 7.48 7.48 7.46 7.46 7.13 7.12 7.12 7.12 7.10 7.09 4.85 4.84 4.25 4.23 4.22 4.21 2.50 DMSO-d6 2.14 2.14 2.13 2.13 2.12 2.09 0.93 0.93 0.91 0.91
D (ddd) 7.86 J(8.7, 7.1, 1.7)
B (dd) 8.18 J(7.9, 1.7)
A (d) 8.32 J(8.6)
E (dd) 7.63 J(8.6, 1.0)
G (m) 7.12
C (d) 8.12 J(8.6)
F (ddd) 7.48 J(8.0, 7.1, 1.0)
X1AADVal(41)
H (d) 4.86
I (dd) 4.23 J(8.5, 5.7)
J (m) 2.13
L (d) 0.91 J(1.8)
K (d) 0.93 J(1.9)
1.0 1.0 0.9 1.0 1.0 1.1 2.2 2.2 1.0 1.2 3.1 3.0
f1 (ppm)

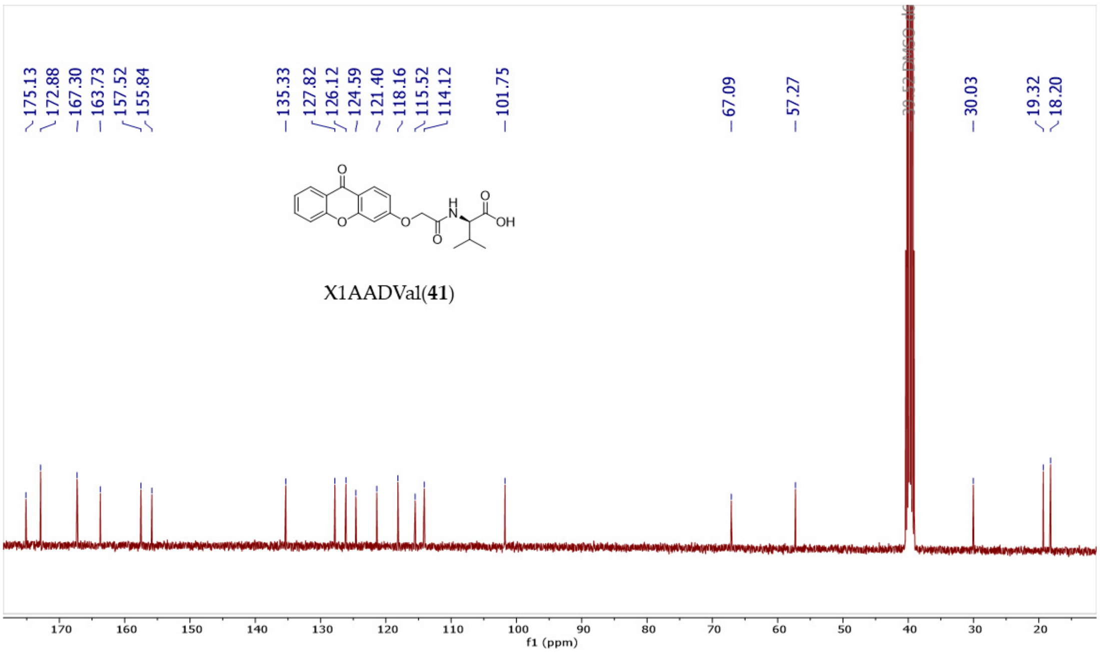
175.13 172.88 167.30 163.73 157.52 155.84 135.33 127.82 126.12 124.59 121.40 118.16 115.52 114.12 101.75 67.09 57.27 30.03 19.32 18.20
X1AADVal(41)
f1 (ppm)

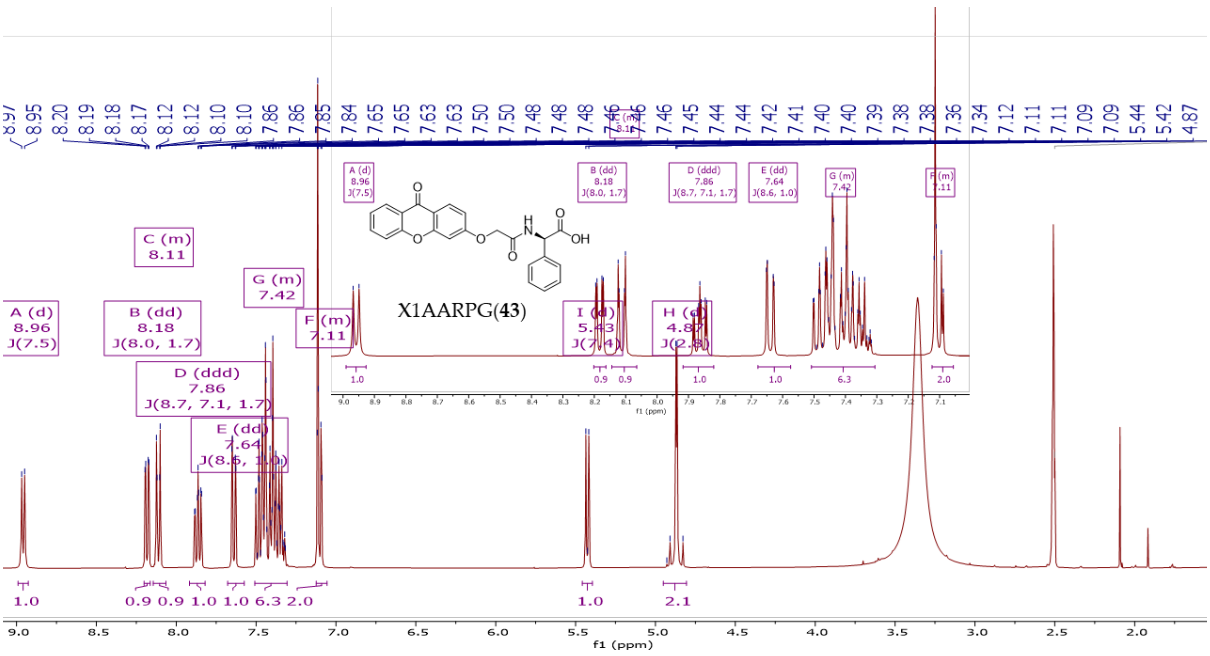
X1AARPG(43)
A (d) 8.96 J(7.5)
B (dd) 8.18 J(8.0, 1.7)
C (m) 8.11
D (ddd) 7.86 J(8.7, 7.1, 1.7)
E (dd) 7.64 J(8.6, 1.0)
F (m) 7.11
G (m) 7.42
I (d) 5.43 J(7.4)
H (d) 4.87 J(2.8)
1.0
0.9
0.9
1.0
1.0
6.3
2.0
1.0
2.1
f1 (ppm)

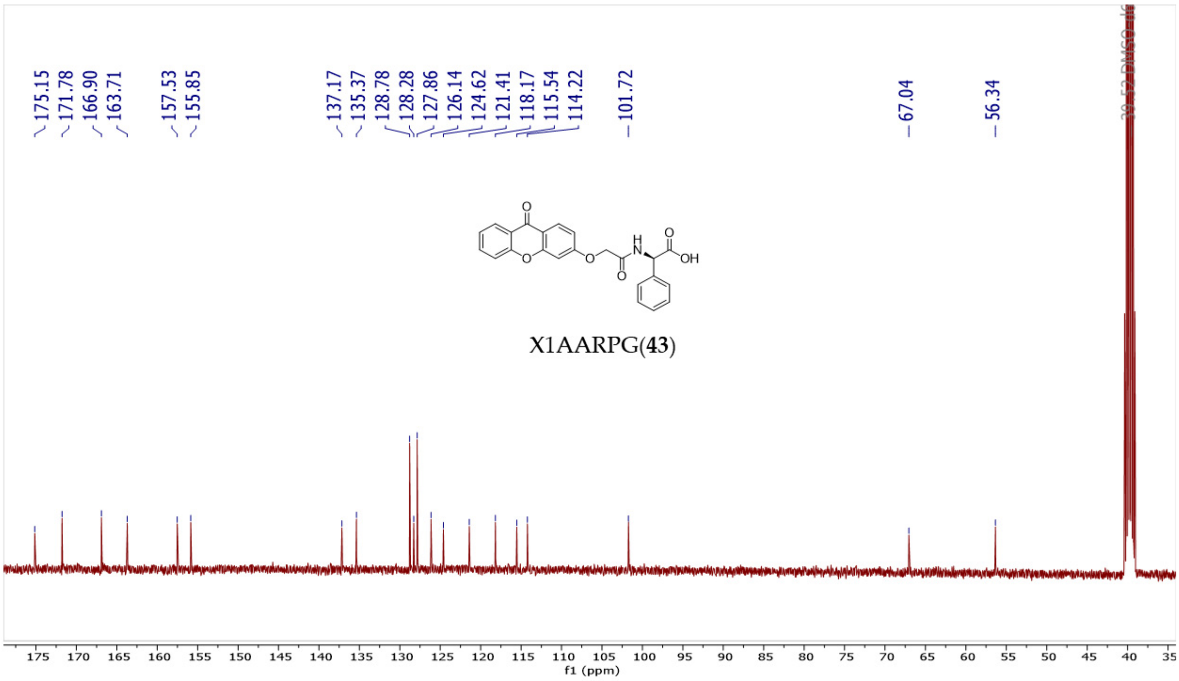
175.15
171.78
166.90
163.71
157.53
155.85
137.17
135.37
128.78
128.28
127.86
126.14
124.62
121.41
118.17
115.54
114.22
101.72
67.04
56.34
X1AARPG(43)
f1 (ppm)

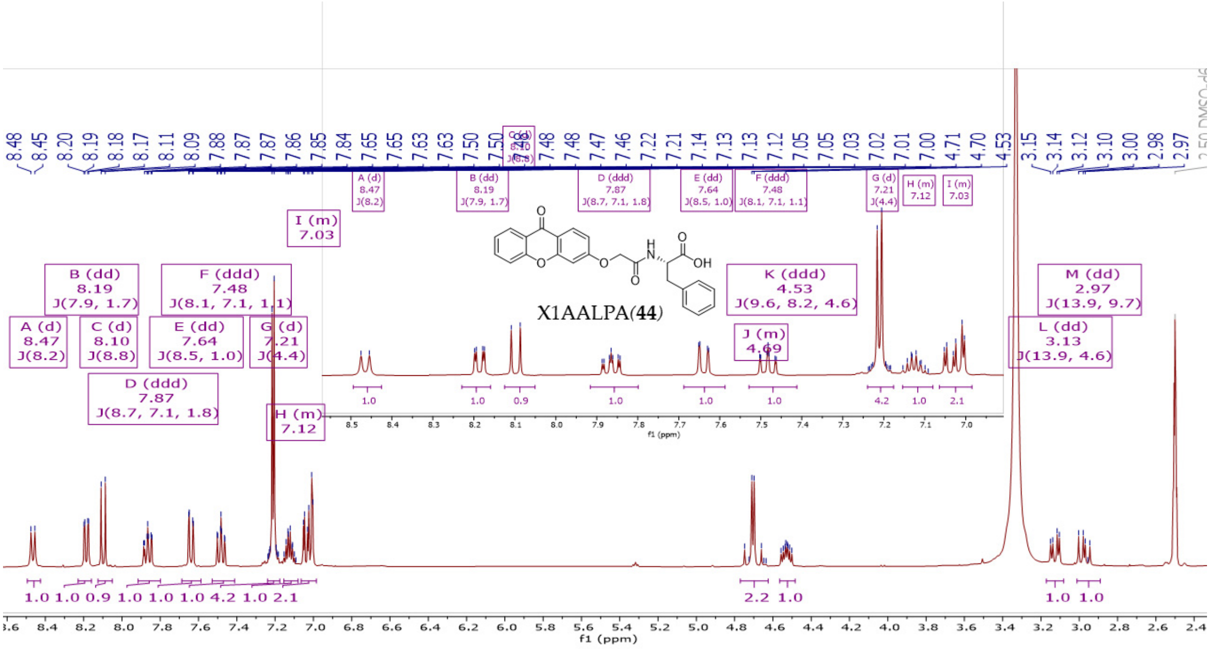
8.48
8.45
8.20
8.19
8.18
8.17
8.11
8.09
7.88
7.87
7.87
7.86
7.85
7.84
7.65
7.65
7.63
7.63
7.50
7.48
7.48
7.47
7.46
7.22
7.21
7.14
7.13
7.13
7.12
7.05
7.05
7.03
7.02
7.01
7.00
4.71
4.70
4.53
3.15
3.14
3.12
3.10
3.00
2.98
2.97
A (d) 8.47 J(8.2)
B (dd) 8.19 J(7.9, 1.7)
C (d) 8.10 J(8.8)
D (ddd) 7.87 J(8.7, 7.1, 1.8)
E (dd) 7.64 J(8.5, 1.0)
F (ddd) 7.48 J(8.1, 7.1, 1.1)
G (d) 7.21 J(4.4)
H (m) 7.12
I (m) 7.03
J (m) 4.69
K (ddd) 4.53 J(9.6, 8.2, 4.6)
L (dd) 3.13 J(13.9, 4.6)
M (dd) 2.97 J(13.9, 9.7)
X1AALPA(44)
1.0 1.0 0.9 1.0 1.0 1.0 4.2 1.0 2.1
2.2 1.0
1.0 1.0
f1 (ppm)

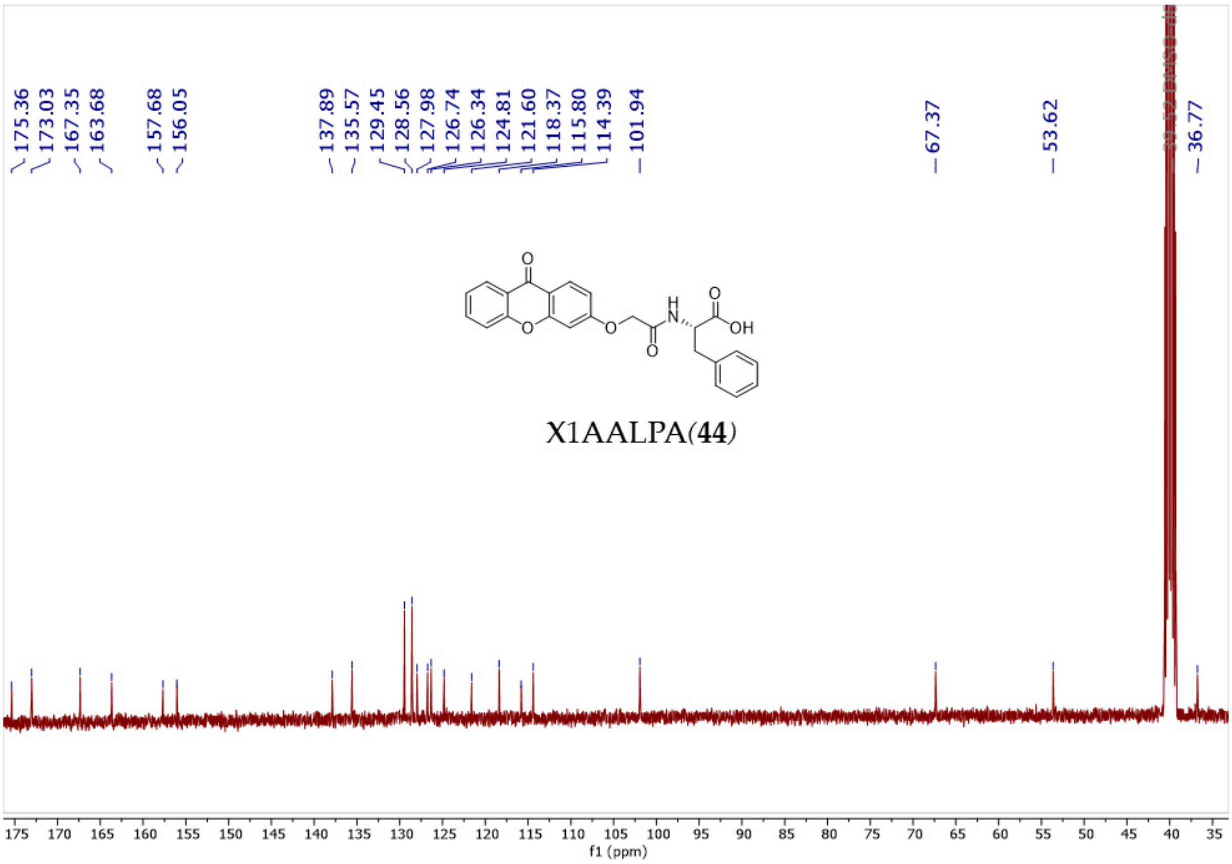
175.36
173.03
167.35
163.68
157.68
156.05
137.89
135.57
129.45
128.56
127.98
126.74
126.34
124.81
121.60
118.37
115.80
114.39
101.94
67.37
53.62
36.77
X1AALPA(44)
f1 (ppm)

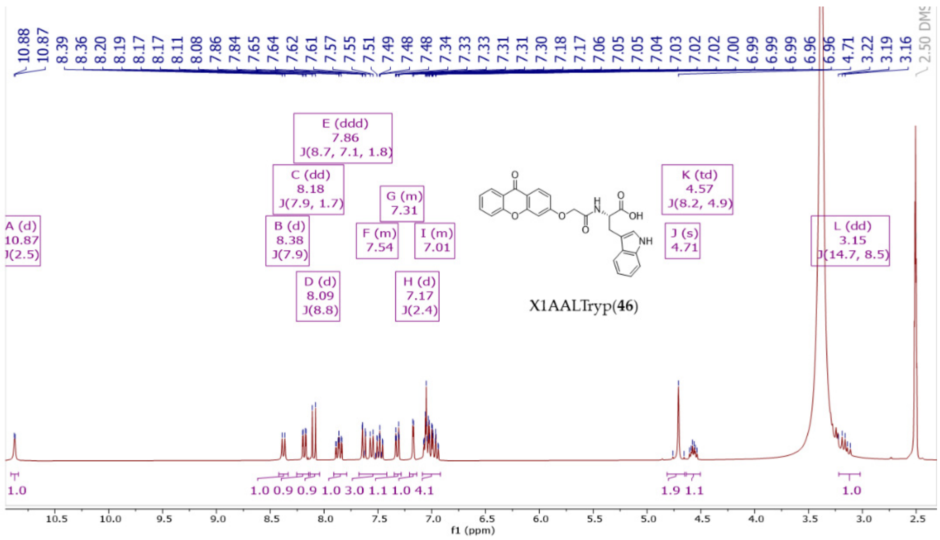
A (d) 10.87 J(2.5)
B (d) 8.38 J(7.9)
C (dd) 8.18 J(7.9, 1.7)
D (d) 8.09 J(8.8)
E (ddd) 7.86 J(8.7, 7.1, 1.8)
F (m) 7.54
G (m) 7.31
H (d) 7.17 J(2.4)
I (m) 7.01
J (s) 4.71
K (td) 4.57 J(8.2, 4.9)
L (dd) 3.15 J(14.7, 8.5)
X1AALTryp(46)
f1 (ppm)

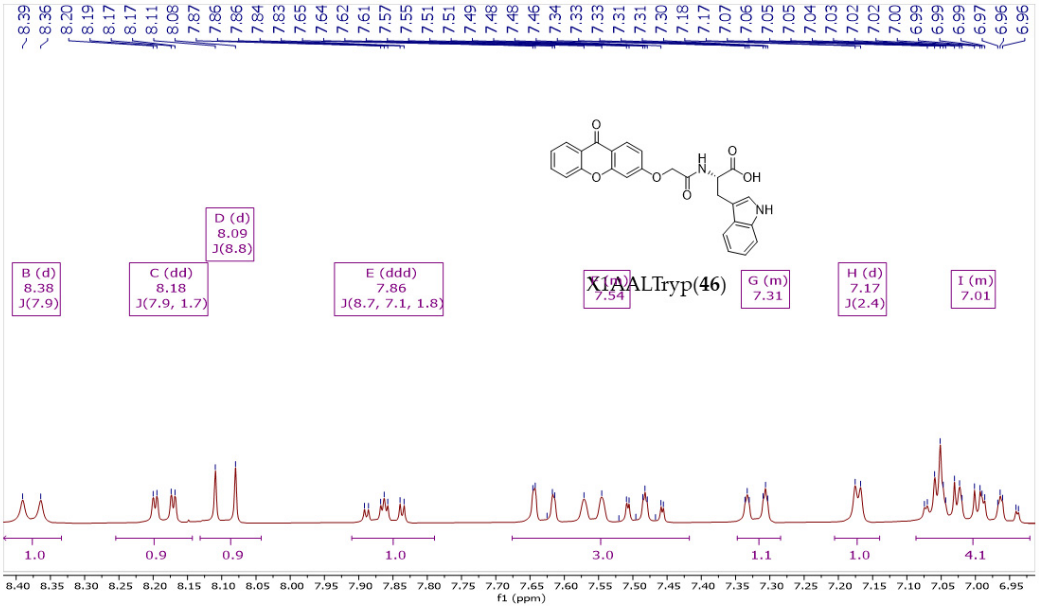
B (d) 8.38 J(7.9)
C (dd) 8.18 J(7.9, 1.7)
D (d) 8.09 J(8.8)
E (ddd) 7.86 J(8.7, 7.1, 1.8)
7.54
X1AALTryp(46)
G (m) 7.31
H (d) 7.17 J(2.4)
I (m) 7.01
f1 (ppm)

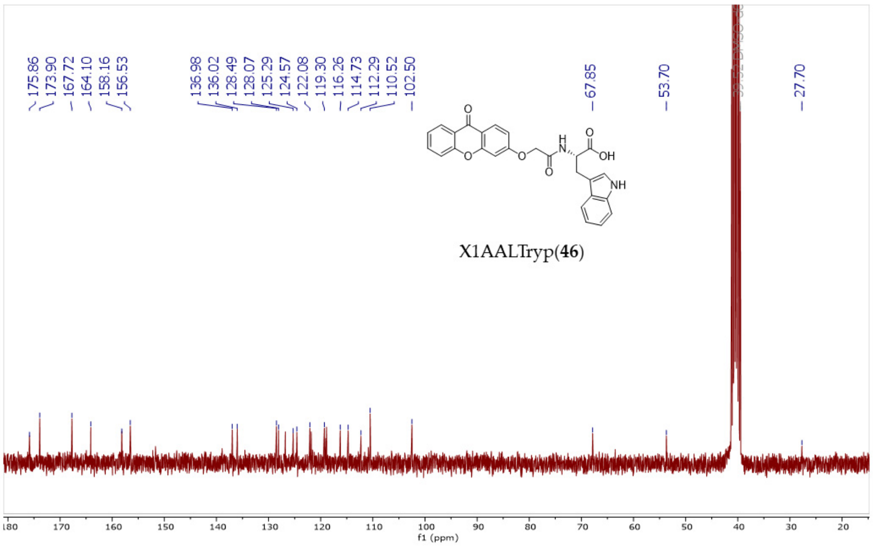
175.86
173.90
167.72
164.10
158.16
156.53
136.98
136.02
128.49
128.07
125.29
124.57
122.08
119.30
116.26
114.73
112.29
110.52
102.50
67.85
53.70
27.70
X1AALTryp(46)
f1 (ppm)

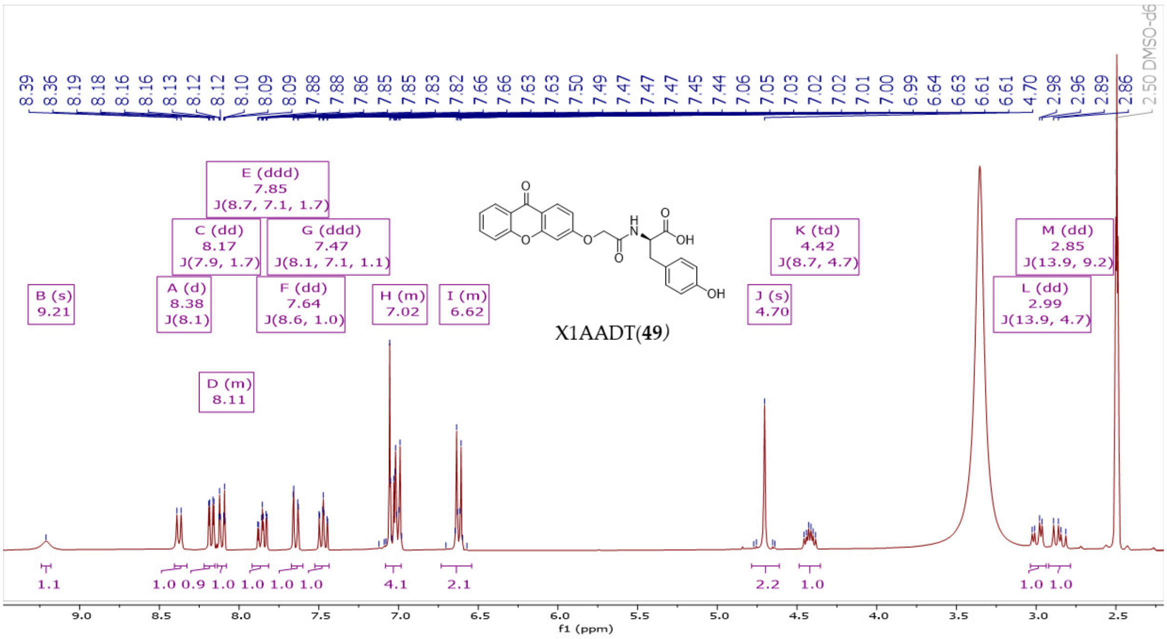
2.50 DMSO-d6
B (s) 9.21
A (d) 8.38 J(8.1)
C (dd) 8.17 J(7.9, 1.7)
D (m) 8.11
E (ddd) 7.85 J(8.7, 7.1, 1.7)
F (dd) 7.64 J(8.6, 1.0)
G (ddd) 7.47 J(8.1, 7.1, 1.1)
H (m) 7.02
I (m) 6.62
J (s) 4.70
K (td) 4.42 J(8.7, 4.7)
L (dd) 2.99 J(13.9, 4.7)
M (dd) 2.85 J(13.9, 9.2)
X1AADT(49)
f1 (ppm)

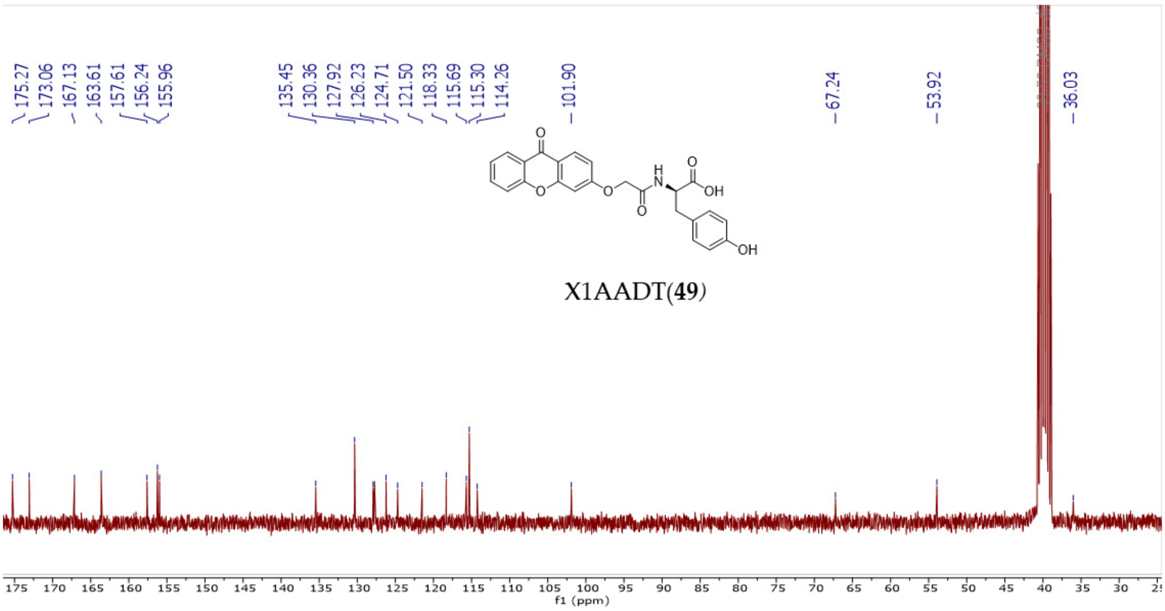
175.27
173.06
167.13
163.61
157.61
156.24
155.96
135.45
130.36
127.92
126.23
124.71
121.50
118.33
115.69
115.30
114.26
101.90
67.24
53.92
36.03
f1 (ppm)

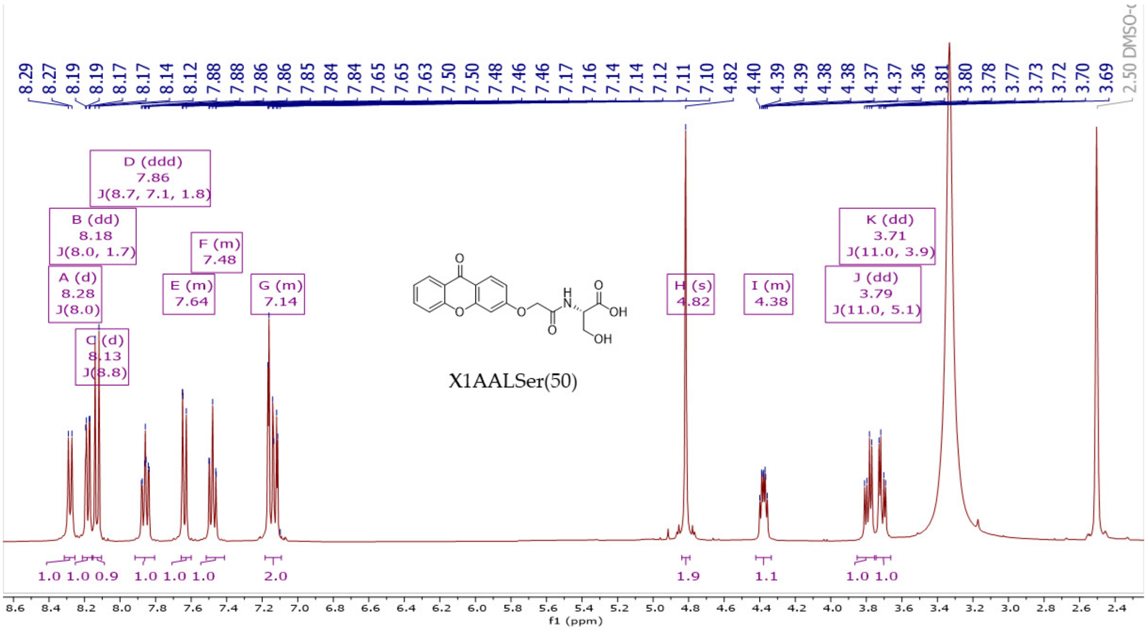
X1AALSer(50)
A (d) 8.28 J(8.0)
B (dd) 8.18 J(8.0, 1.7)
C (d) 8.13 J(8.8)
D (ddd) 7.86 J(8.7, 7.1, 1.8)
E (m) 7.64
F (m) 7.48
G (m) 7.14
H (s) 4.82
I (m) 4.38
J (dd) 3.79 J(11.0, 5.1)
K (dd) 3.71 J(11.0, 3.9)
2.50 DMSO-
f1 (ppm)

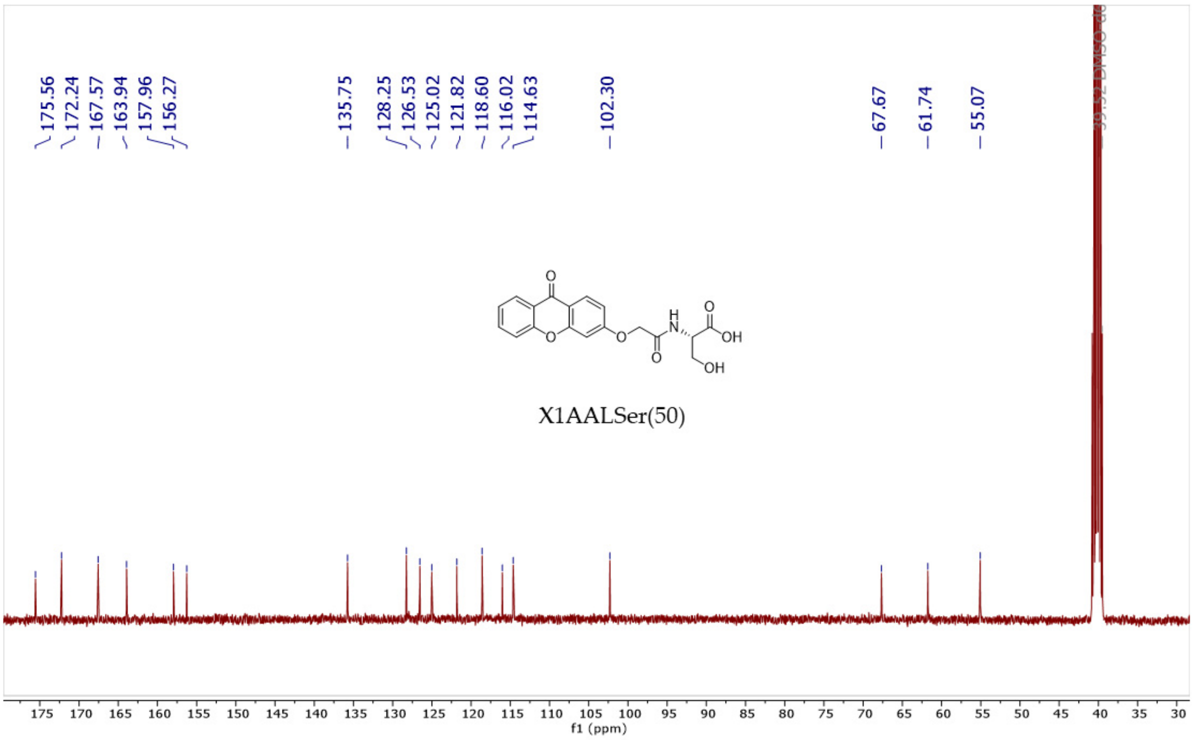
175.56
172.24
167.57
163.94
157.96
156.27
135.75
128.25
126.53
125.02
121.82
118.60
116.02
114.63
102.30
67.67
61.74
55.07
X1AALSer(50)
f1 (ppm)

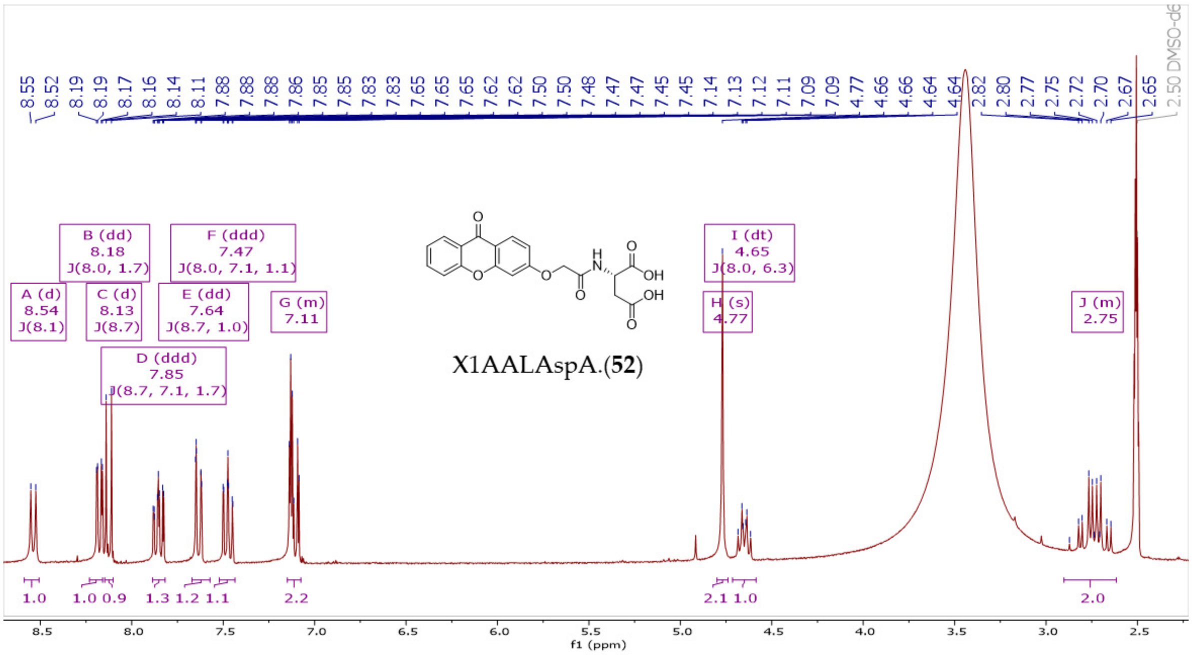
X1AALAspA.(52)
A (d) 8.54 J(8.1)
B (dd) 8.18 J(8.0, 1.7)
C (d) 8.13 J(8.7)
D (ddd) 7.85 J(8.7, 7.1, 1.7)
E (dd) 7.64 J(8.7, 1.0)
F (ddd) 7.47 J(8.0, 7.1, 1.1)
G (m) 7.11
H (s) 4.77
I (dt) 4.65 J(8.0, 6.3)
J (m) 2.75
2.50 DMSO-d6
f1 (ppm)

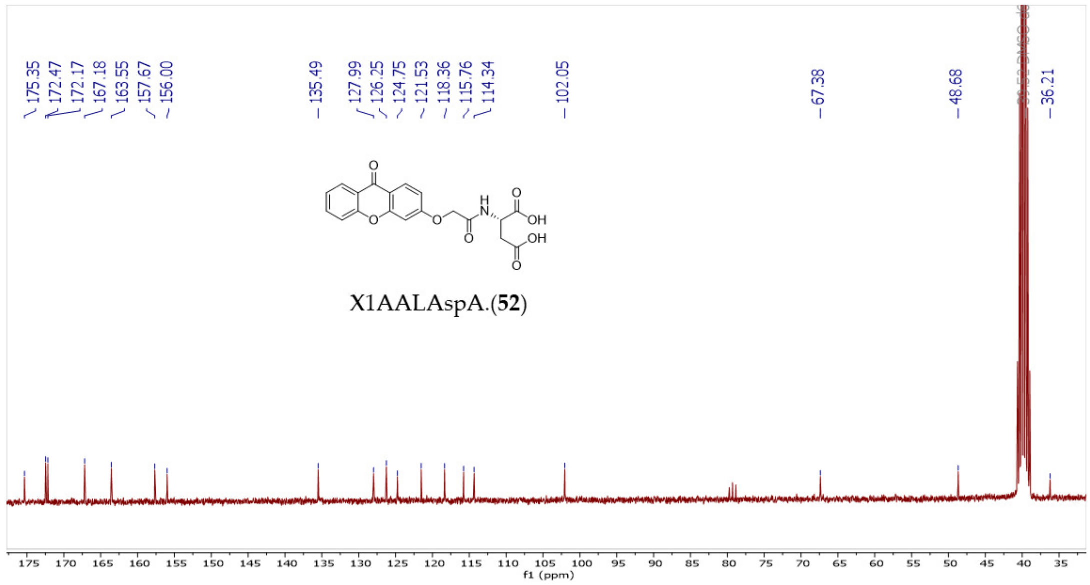

X1AALAspA.(**52**)

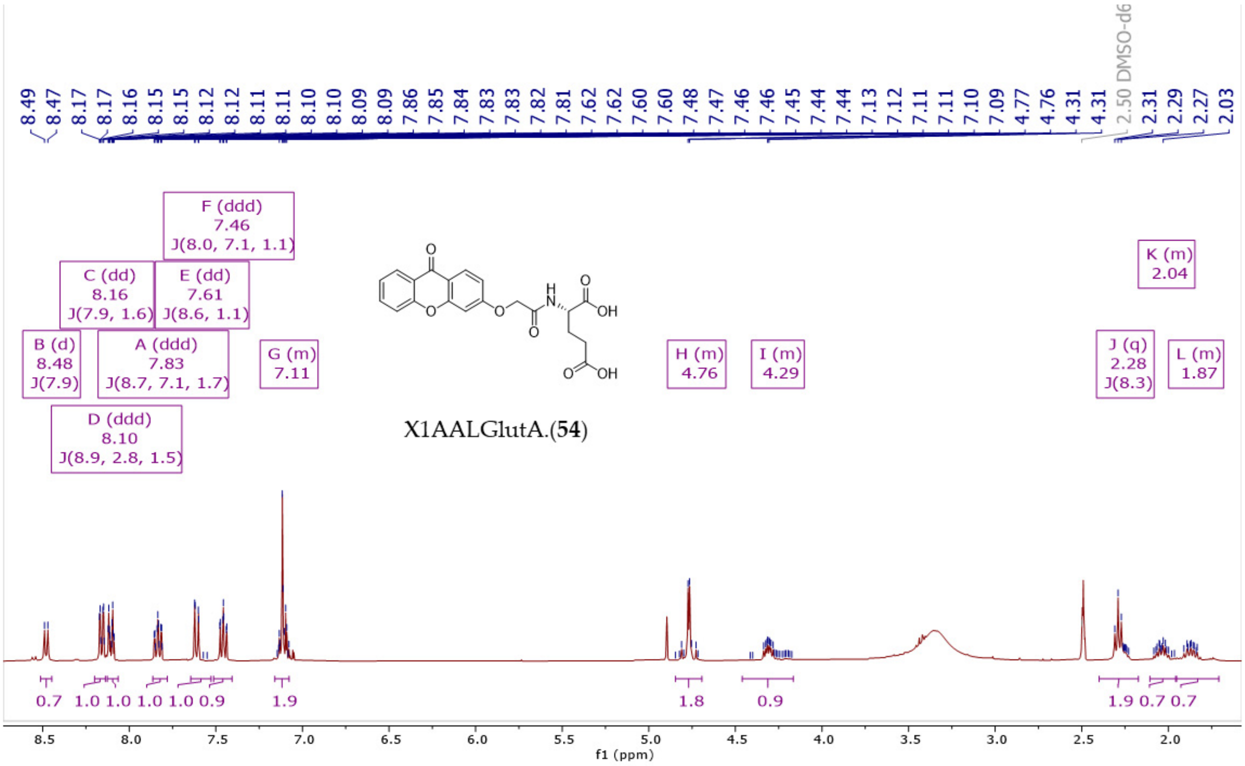

X1AALGlutA.(**54**)

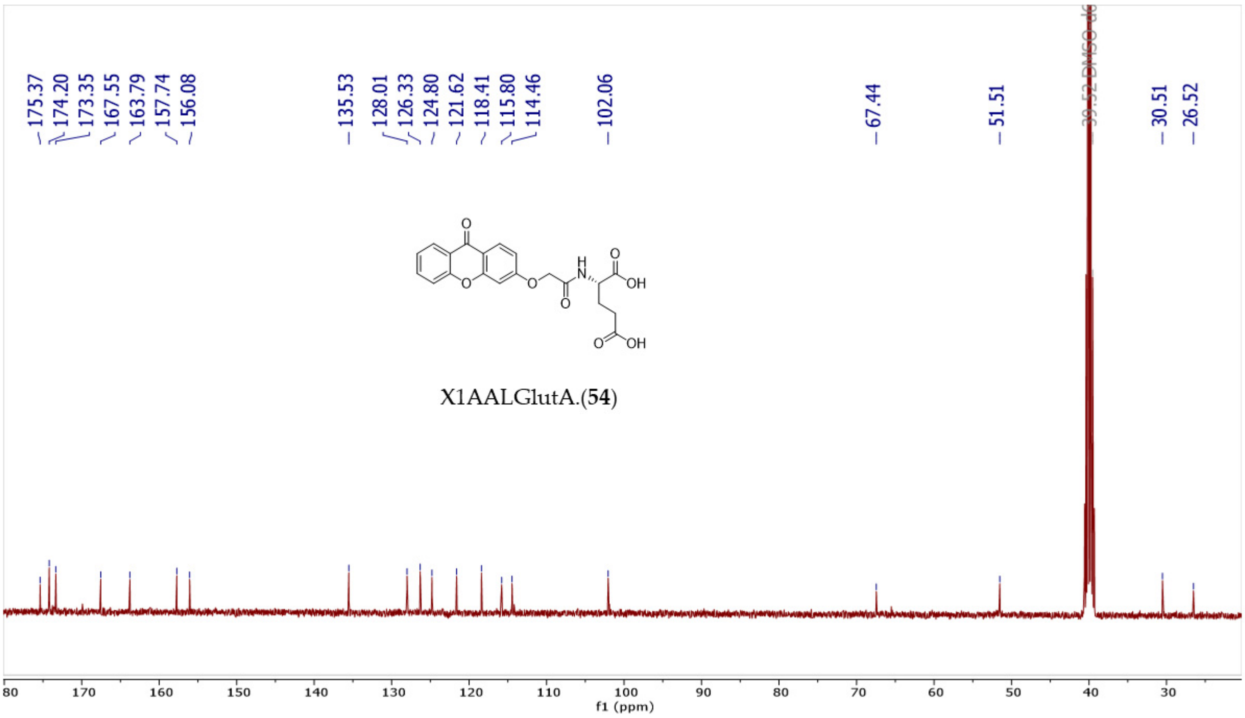
175.37
174.20
173.35
167.55
163.79
157.74
156.08
135.53
128.01
126.33
124.80
121.62
118.41
115.80
114.46
102.06
67.44
51.51
39.52 DMSO-d6
30.51
26.52
X1AALGlutA.(54)
f1 (ppm)

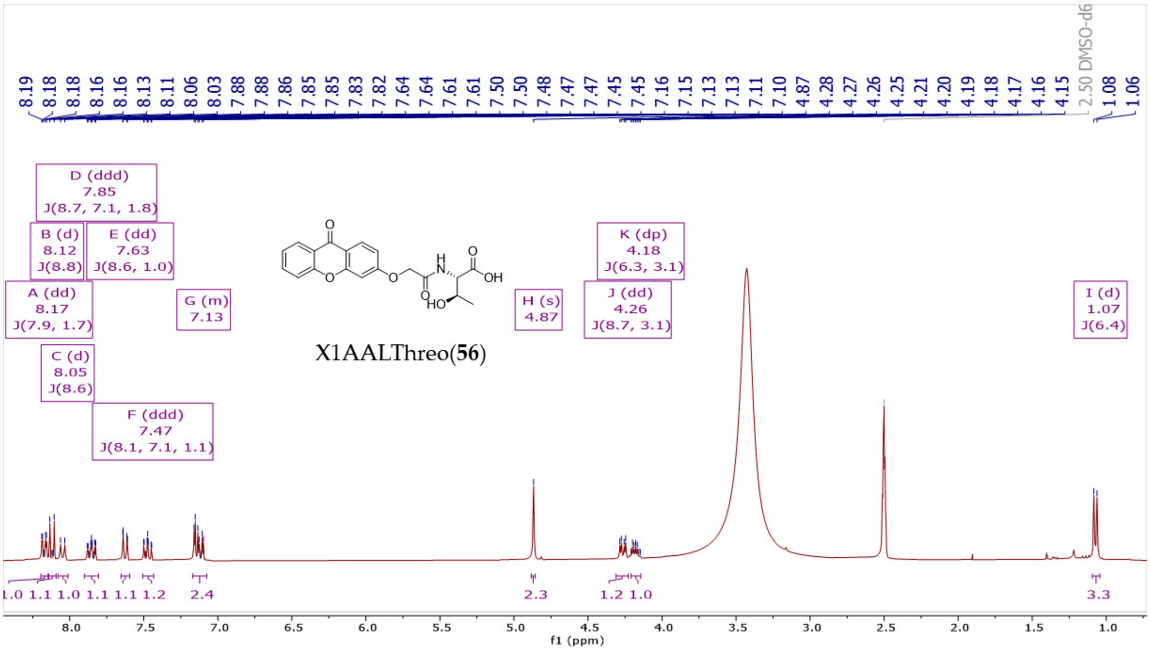
2.50 DMSO-d6
A (dd) 8.17 J(7.9, 1.7)
B (d) 8.12 J(8.8)
C (d) 8.05 J(8.6)
D (ddd) 7.85 J(8.7, 7.1, 1.8)
E (dd) 7.63 J(8.6, 1.0)
F (ddd) 7.47 J(8.1, 7.1, 1.1)
G (m) 7.13
H (s) 4.87
J (dd) 4.26 J(8.7, 3.1)
K (dp) 4.18 J(6.3, 3.1)
I (d) 1.07 J(6.4)
X1AALThreo(56)
f1 (ppm)

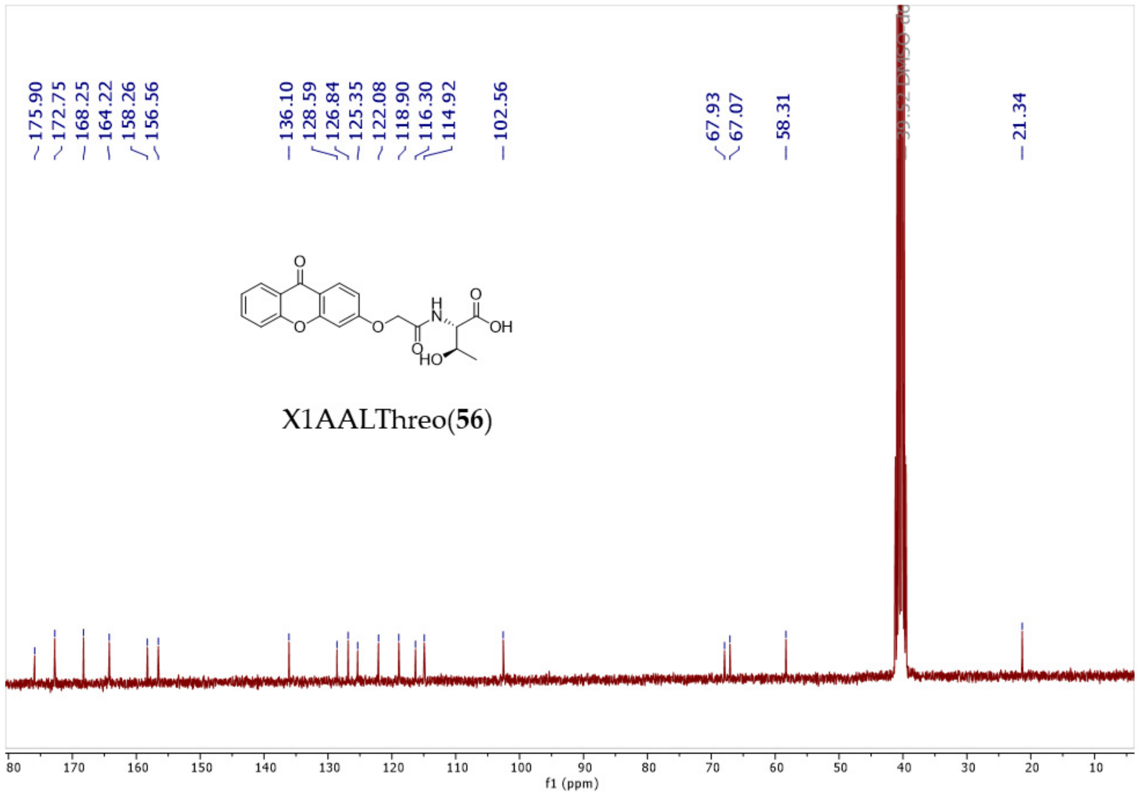
175.90
172.75
168.25
164.22
158.26
156.56
136.10
128.59
126.84
125.35
122.08
118.90
116.30
114.92
102.56
67.93
67.07
58.31
39.52 DMSO-d6
21.34
X1AALThreo(56)
f1 (ppm)

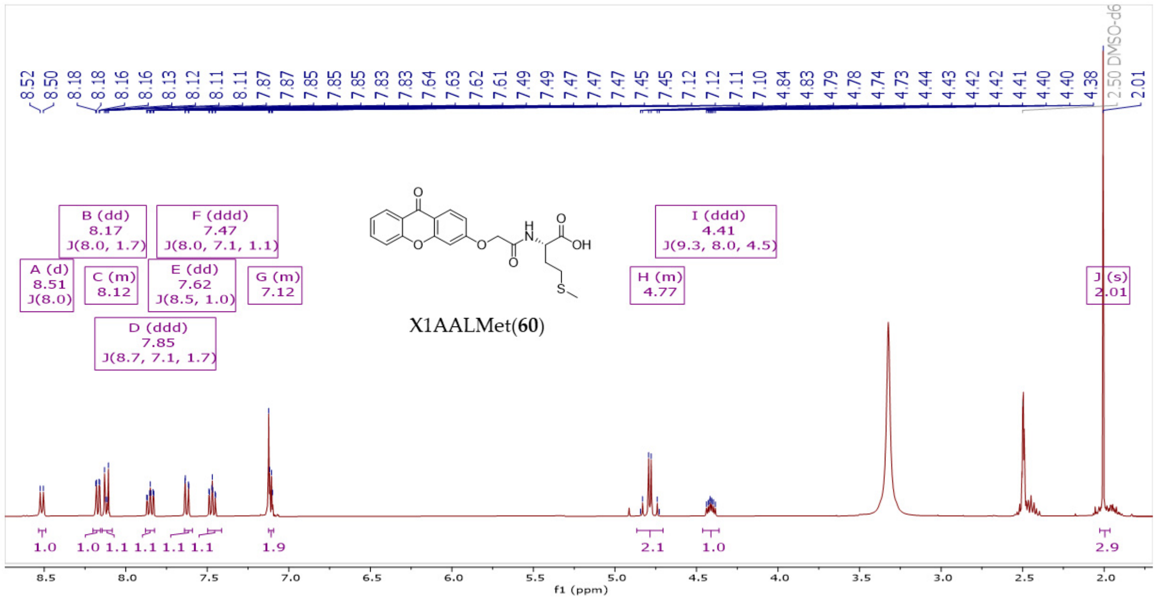
8.52
8.50
8.18
8.18
8.16
8.16
8.13
8.12
8.11
8.11
7.87
7.87
7.85
7.85
7.85
7.83
7.83
7.64
7.63
7.62
7.61
7.49
7.49
7.47
7.47
7.47
7.45
7.45
7.12
7.12
7.11
7.10
4.84
4.83
4.79
4.78
4.74
4.73
4.44
4.43
4.42
4.42
4.41
4.40
4.40
4.38
2.50 DMSO-d6
2.01
A (d) 8.51 J(8.0)
B (dd) 8.17 J(8.0, 1.7)
C (m) 8.12
D (ddd) 7.85 J(8.7, 7.1, 1.7)
E (dd) 7.62 J(8.5, 1.0)
F (ddd) 7.47 J(8.0, 7.1, 1.1)
G (m) 7.12
H (m) 4.77
I (ddd) 4.41 J(9.3, 8.0, 4.5)
J (s) 2.01
X1AALMet(60)
1.0 1.0 1.1 1.1 1.1 1.1 1.9 2.1 1.0 2.9
f1 (ppm)

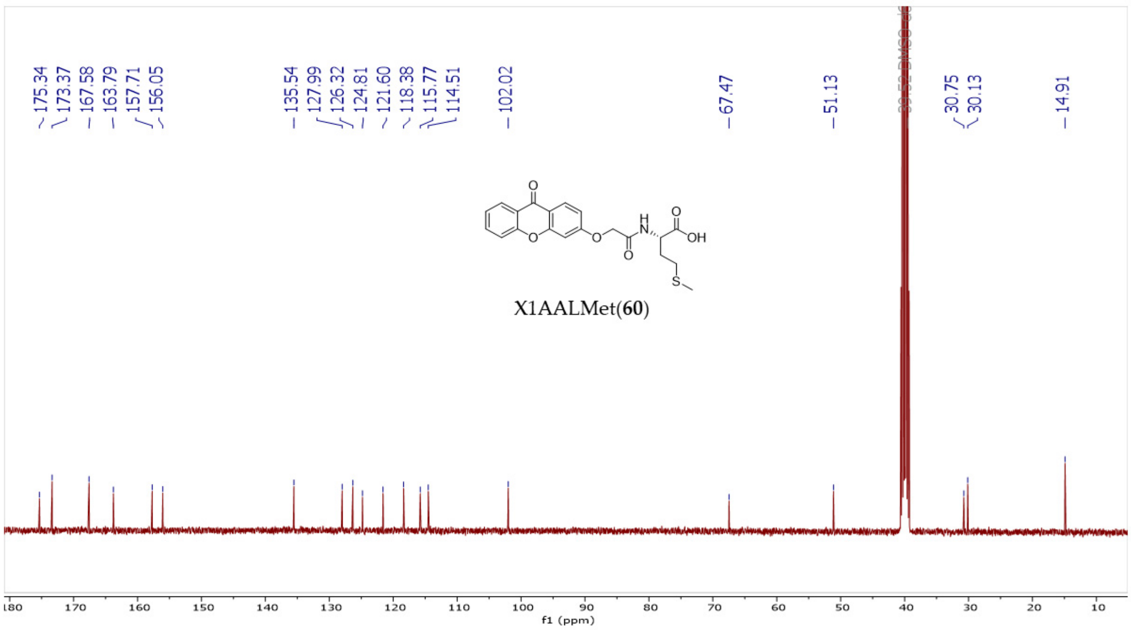

Figure S1. ^{1}H and ^{13}C NMR spectra of CDXs.

Table S3. Physicochemical descriptors and predicted ADME parameters, pharmacokinetic properties, and drug-like nature for CDXs of amino ester and amino acids.

CDXs	Molecular weight (g/mol)	TPSA (Å²)	Log $P_{o/w}$ (iLOGP)	GI absorption	BBB permeant	P-gp substrate	CYP3A4 inhibitor	Lipinski	Veber	Bioavailability Score
X1AELAla **2**	397.42	94.84	3.48	High	No	No	Yes	Yes; 0 violation	Yes	0.55
X1AEGli **4**	341.31	94.84	2.67	High	No	No	Yes	Yes; 0 violation	Yes	0.55
X1AELIsoLeu **5**	397.42	94.84	3.30	High	No	No	Yes	Yes; 0 violation	Yes	0.55
X1AELLeu **6**	397.42	94.84	3.50	High	No	No	Yes	Yes; 0 violation	Yes	0.55
X1AELProl **8**	381.38	86.05	3.24	High	No	No	Yes	Yes; 0 violation	Yes	0.55
X1AELVal **10**	383.39	86.05	3.33	High	No	No	Yes	Yes; 0 violation	Yes	0.55
X1AESPG **12**	417.41	86.05	3.40	High	No	No	Yes	Yes; 0 violation	Yes	0.55
X1AELPA **14**	431.44	86.05	3.31	High	No	No	Yes	Yes; 0 violation	Yes	0.55
X1AELTryp **16**	470.47	110.63	3.38	High	No	Yes	Yes	Yes; 0 violation	Yes	0.55
X1AELT **18**	447.44	115.07	2.84	High	No	No	Yes	Yes; 0 violation	Yes	0.55
X1AELSer **20**	371.34	115.07	2.96	High	No	No	Yes	Yes; 0 violation	Yes	0.55
X1AELAspA. **22**	413.38	121.14	3.32	High	No	No	Yes	Yes; 0 violation	Yes	0.55
X1AELGlutA. **24**	427.40	121.14	3.15	High	No	No	Yes	Yes; 0 violation	Yes	0.55

X1AELThreo **26**	385.37	115.07	2.75	High	No	No	Yes	Yes; 0 violation	Yes	0.55
X1AELCyst **28**	387.41	133.64	2.79	High	No	No	Yes	Yes; 0 violation	Yes	0.55
X1AELMet **30**	415.46	120.14	3.13	High	No	No	Yes	Yes; 0 violation	Yes	0.55
X1AALAla **32**	341.31	105.84	1.97	High	No	No	No	Yes; 0 violation	Yes	0.56
X1AAGli **34**	327.29	105.84	1.82	High	No	No	No	Yes; 0 violation	Yes	0.56
X1AALIsoLeu **35**	383.39	105.84	2.82	High	No	No	Yes	Yes; 0 violation	Yes	0.56
X1AALLeu **36**	383.39	105.84	2.64	High	No	No	Yes	Yes; 0 violation	Yes	0.56
X1AALProl **38**	367.35	97.05	2.30	High	No	No	No	Yes; 0 violation	Yes	0.56
X1AALVal **40**	369.37	105.84	2.52	High	No	No	Yes	Yes; 0 violation	Yes	0.56
X1AALPG 42	403.38	105.84	2.64	High	No	No	Yes	Yes; 0 violation	Yes	0.56
X1AALPA 44	417.41	105.84	2.56	High	No	No	Yes	Yes; 0 violation	Yes	0.56
X1AALTryp 46	456.45	121.63	2.53	High	No	No	Yes	Yes; 0 violation	Yes	0.56
X1AALTyr 48	433.41	126.07	2.08	High	No	No	No	Yes; 0 violation	Yes	0.56
X1AALSer 50	357.31	126.07	1.74	High	No	No	No	Yes; 0 violation	Yes	0.56
X1AALAspA. 52	385.32	143.14	1.67	High	No	No	No	Yes; 0 violation	Yes	0.56
X1AALGlutA. 54	399.35	143.14	1.41	High	No	No	No	Yes; 0 violation	Yes	0.56
X1AALThreo 56	371.34	126.07	2.08	High	No	No	No	Yes; 0 violation	Yes	0.56

X1AALCyst 58	373.38	144.64	2.00	Low	No	No	No	Yes; 0 violation	No; 1 TPSA>140	0.56
X1AALMet 60	401.43	131.14	2.19	High	No	No	Yes	Yes; 0 violation	Yes	0.56

Table S4: Results of HRMS-ESI in positive and negative ions mode

CDXs	Calculate	Polarit	ESI/MS^n	
	d	y	[M–H]$^-$	[M+H]$^+$
**X1AEDAla**	$C_{22}H_{23}NO_6$	-	396.1455	
(3)	397,1525	+		398.1612
**X1AELLeu**	$C_{22}H_{23}NO_6$	-	396.1469	
(6)	397,1525	+		398.1622
**X1AEDLeu**	$C_{22}H_{23}NO_6$	-	396.1466	
(7)	397,1525	+		398.1616
**X1AEDVal**	$C_{21}H_{21}NO_6$	-	382.1310	
(11)	383.1369	+		384.1453
**X1AELPG**	$C_{24}H_{19}NO$	-	416.1149	
(12)	$_6$	+		418.1297
**X1AELTryp**	$C_{27}H_{22}N2$	-	469.1408	
(16)	O_6	+		471.1566
**X1AELSer**	$C_{21}H_{19}NO_8$	-	370.0936	
(20)	371,1005	+		372.1084
**X1AEDAspA**	$C_{21}H_{19}NO_8$	-	412.1052	
.	413.1111	+		414.1207
**X1AAGli**	$C_{17}H_{13}NO_6$	-	326.0686	
(34)	327.0743	+		328.0838
**X1AALIsoLe**	$C_{21}H_{21}NO_6$	-	382.1325	
**u**	383.1369	+		384.1464
**X1AALLeu**	$C_{21}H_{21}NO_6$	-	382.1319	
(36)	383.1369	+		384.1443
**X1AADVal**	$C_{20}H_{19}NO_6$	-	368.1166	
(41)	369.1212	+		370.1296
**X1AADPG**	$C_{23}H_{17}NO_6$	-	402.1010	
(43)	403.3900	+		404.1137
**X1AALPA**	$C_{24}H_{19}NO_6$	-	416.1165	
(44)	417.1212	+		418.1292
**X1AALTryp**	$C_{26}H_{20}N_2O$	-	455.1279	
(46)	$_6$	+		457.1409
		-	356.0799	

**X1AALSer**	$C_{18}H_{15}NO_7$	+		358.0955
**X1AALAspA**	$C_{19}H_{15}NO_8$	-	384.0737	
**.**	385.0798	+		386.0879
**X1AALGluA**	$C_{20}H_{17}NO_8$	-	398.0908	
**.**	399.0954	+		400.1048
**X1AADThre**	$C_{19}H_{17}NO_7$	-	370.0957	
**o**	371.1005	+		372.1086

2. CYTOTOXICITY AND ANTI-INFLAMMATORY ACTIVITY

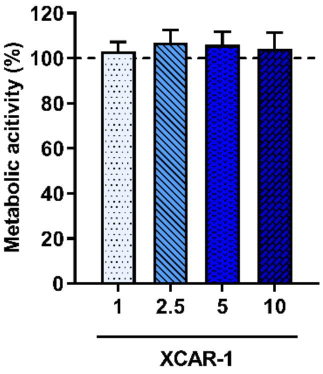

Figure S2. *Metabolic activity of LPS-activated macrophages cultured in the presence of XCAR-1 at different concentrations (µM) for 22 h of culture. No statistically significant differences were observed.*

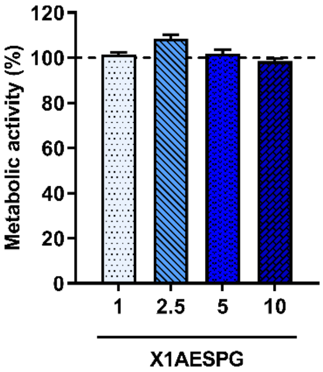

Figure S3. *Metabolic activity of LPS-activated macrophages cultured in the presence of X1AESPG at different concentrations (µM) for 22 h of culture. No statistically significant differences were observed.*

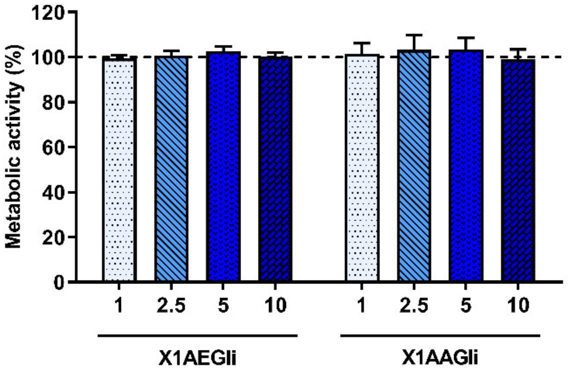

Figure S4. *Metabolic activity of LPS-activated macrophages cultured in the presence of X1AEGli and X1AAGli at different concentrations (µM) for 22 h of culture. No statistically significant differences were observed.*

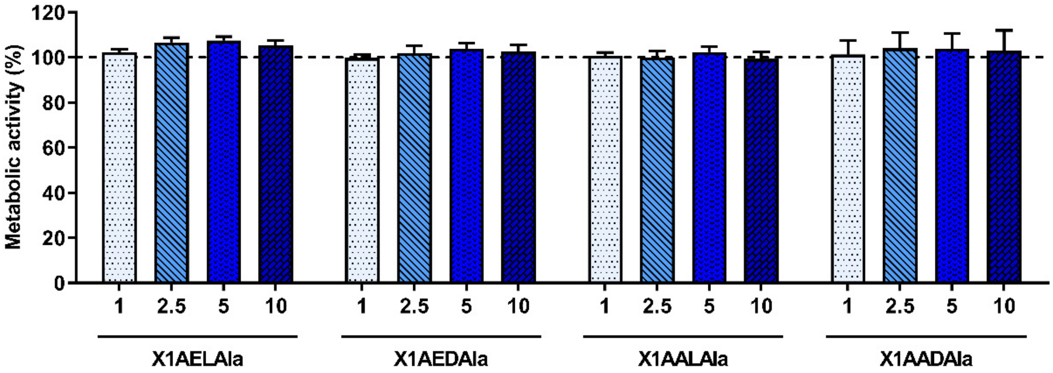

Figure S5. *Metabolic activity of LPS-activated macrophages cultured in the presence of X1AELAla, X1AEDAla, X1AALAla, and X1AADAla at different concentrations (µM) for 22 h of culture. No statistically significant differences were observed.*

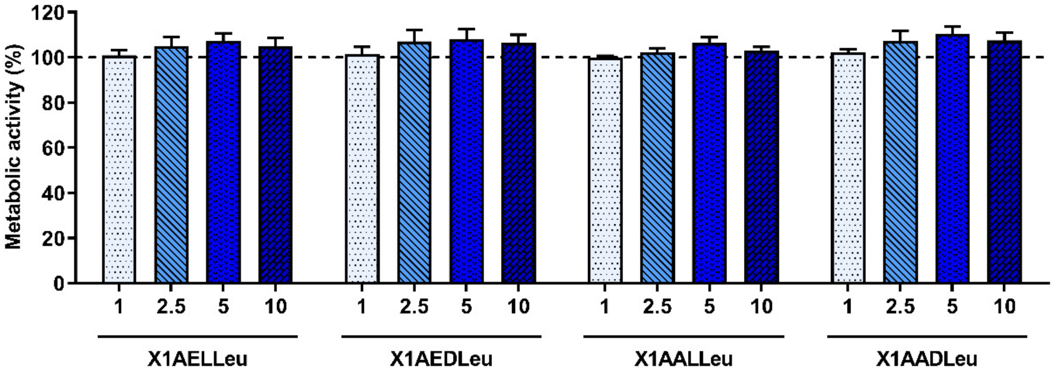

Figure S6. *Metabolic activity of LPS-activated macrophages cultured in the presence of X1AELLeu, X1AEDLeu, X1AALLeu, and X1AADLeu at different concentrations (µM) for 22 h of culture. No statistically significant differences were observed.*

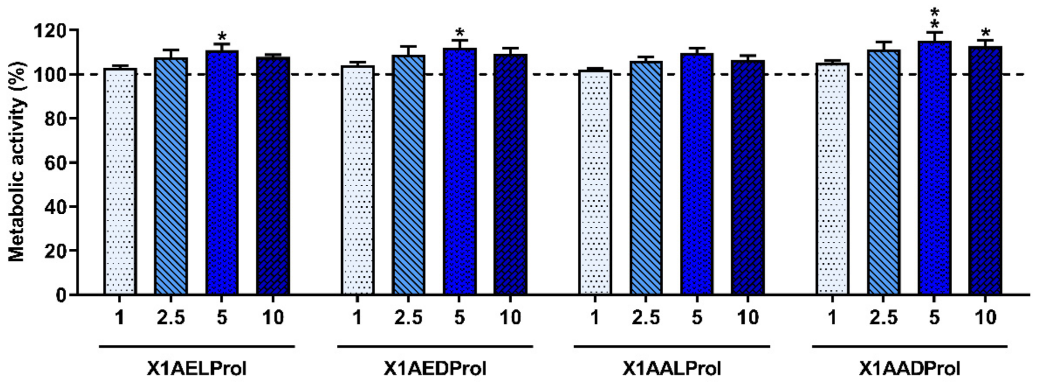

*Figure S7. Metabolic activity of LPS-activated macrophages cultured in the presence of X1AELProl, X1AEDProl, X1AALProl, and X1AADProl at different concentrations (µM) for 22 h of culture. Statistically significant differences are * ($p < 0.0435$) and ** ($p < 0.084$) in comparison with non-treated LPS-stimulated macrophages.*

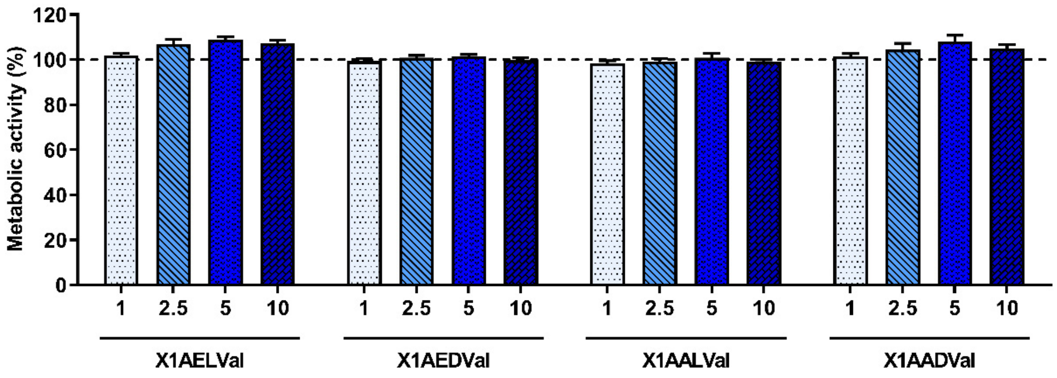

Figure S8. Metabolic activity of LPS-activated macrophages cultured in the presence of X1AELVal, X1AEDVal, X1AALVal, and X1AADVal at different concentrations (µM) for 22 h of culture. No statistically significant differences were observed.

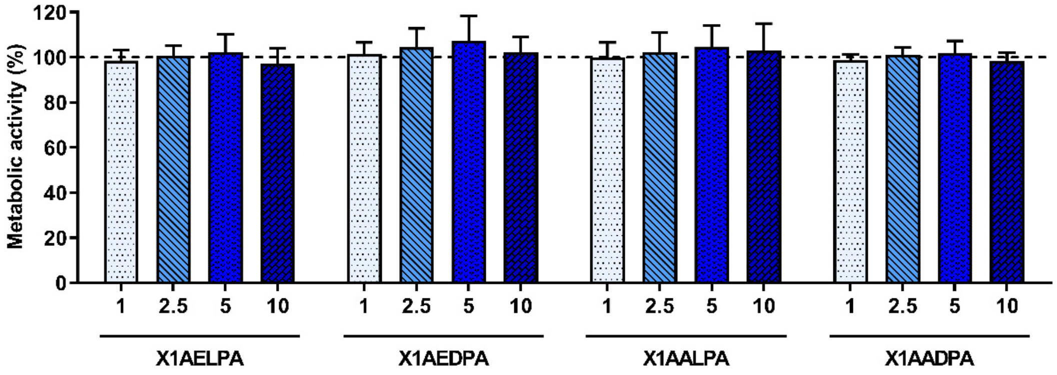

Figure S9. Metabolic activity of LPS-activated macrophages cultured in the presence of X1AELPA, X1AEDPA, X1AALPA, and X1AADPA at different concentrations (µM) for 22 h of culture. No statistically significant differences were observed.

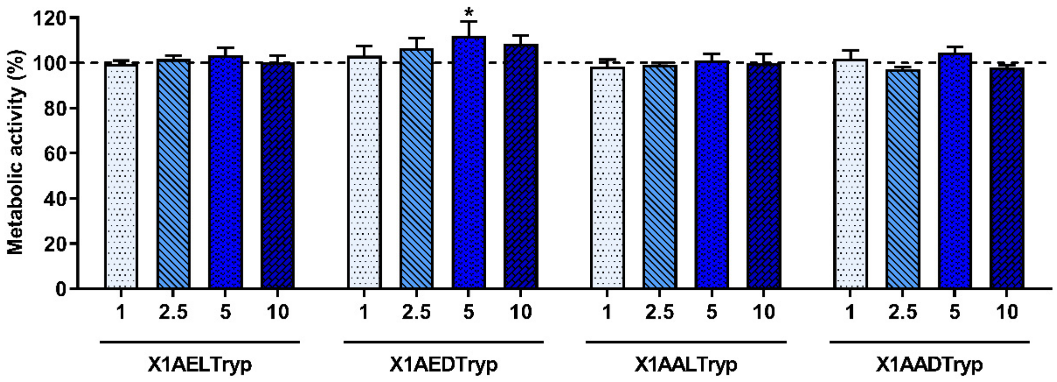

Figure S10. Metabolic activity of LPS-activated macrophages cultured in the presence of X1AELTryp, X1AEDTryp, X1AALTryp, and X1AADTryp at different concentrations (μM) for 22 h of culture. Statistically significant differences are * ($p < 0.0238$) in comparison with non-treated LPS-stimulated macrophages.

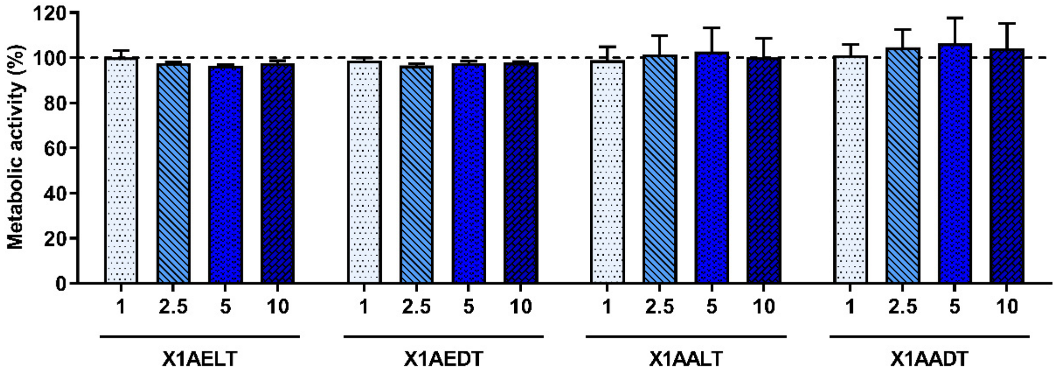

Figure S11. Metabolic activity of LPS-activated macrophages cultured in the presence of X1AELT, X1AEDT, X1AALT, and X1AADT at different concentrations (μM) for 22 h of culture. No statistically significant differences were observed.

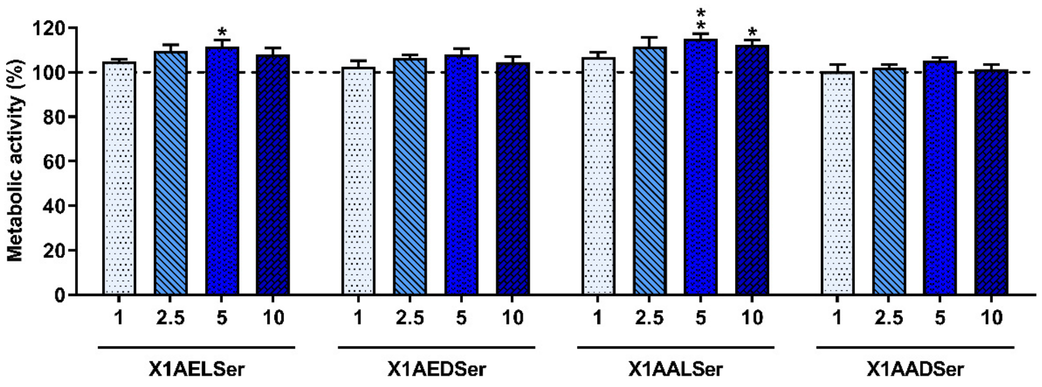

Figure S12. Metabolic activity of LPS-activated macrophages cultured in the presence of X1AELSer, X1AEDSer, X1AALSer, and X1AADSer at different concentrations (μM) for 22 h of culture. Statistically significant differences are * ($p < 0.0435$) and ** ($p < 0.084$) in comparison with non-treated LPS-stimulated macrophages.

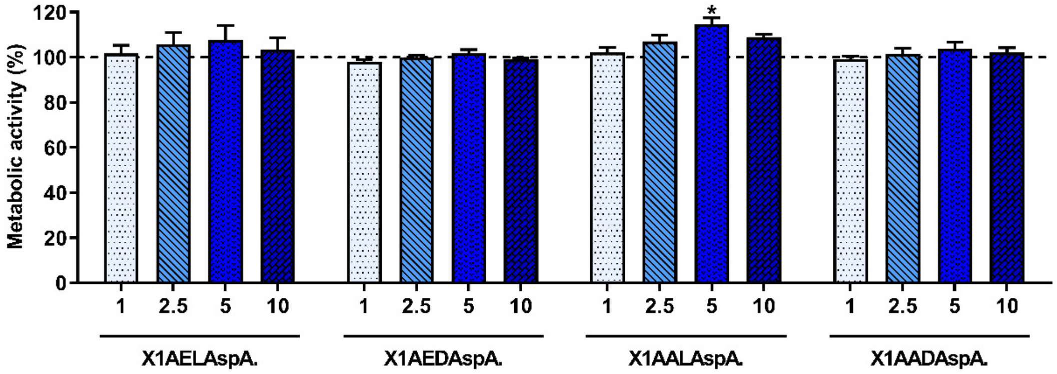

*__Figure S13.__ Metabolic activity of LPS-activated macrophages cultured in the presence of X1AELAspA., X1AEDAspA., X1AALAspA., and X1AADAspA. at different concentrations (µM) for 22 h of culture. Statistically significant differences are * (p < 0.0435) in comparison with non-treated LPS-stimulated macrophages.*

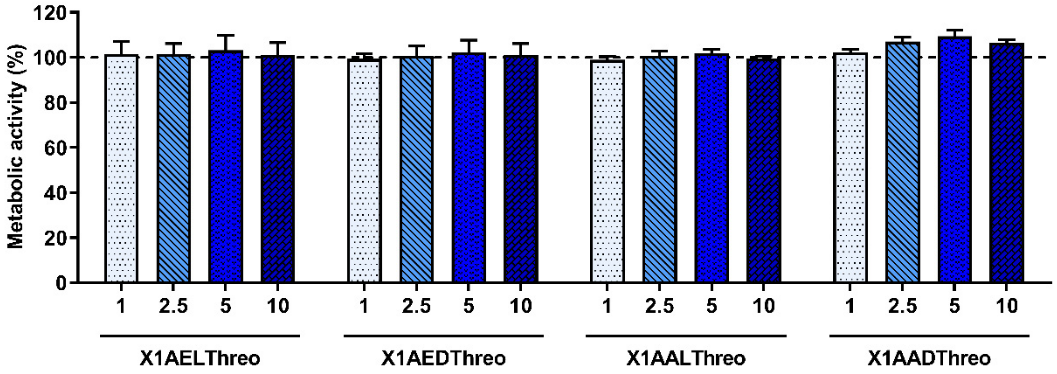

__Figure S14.__ Metabolic activity of LPS-activated macrophages cultured in the presence of X1AELThreo, X1AEDThreo, X1AALThreo, and X1AADThreo at different concentrations (µM) for 22 h of culture. No statistically significant differences were observed.

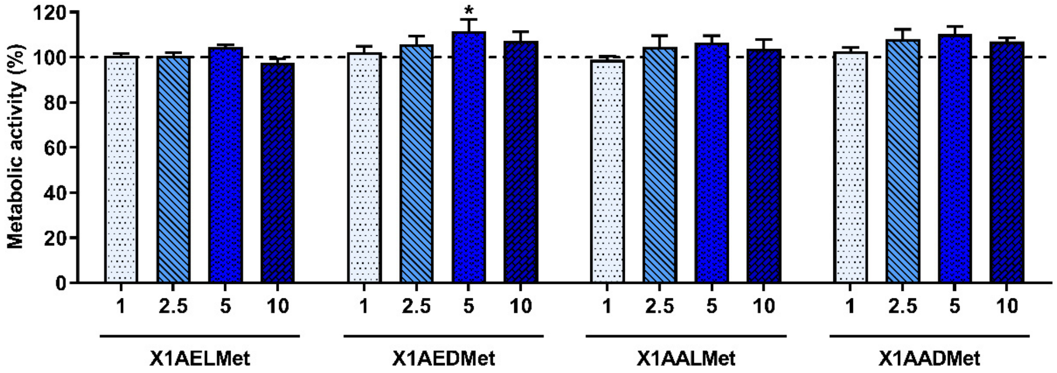

*__Figure S15.__ Metabolic activity of LPS-activated macrophages cultured in the presence of X1AELMet, X1AEDMet, X1AALMet, and X1AADMet at different concentrations (µM) for 22 h of culture. Statistically significant differences are * (p < 0.0238) in comparison with non-treated LPS-stimulated macrophages.*

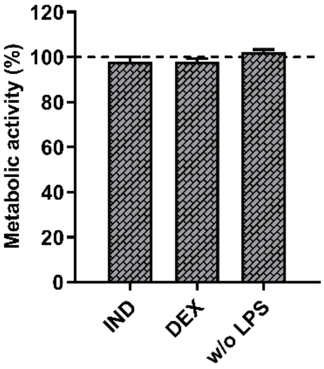

Figure S16. *Metabolic activity of LPS-activated macrophages cultured in the presence of clinically used anti-inflammatory drugs (indomethacin – INDO – and dexamethasone – DEX –, at 10 µM), and non-stimulated macrophages (w/o LPS), for 22 h of culture. No statistically significant differences were observed.*

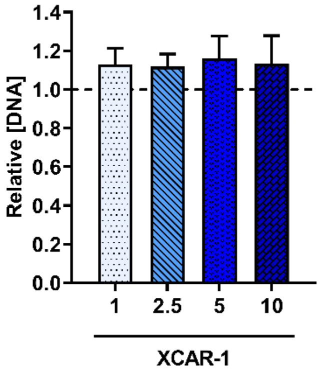

Figure S17. *DNA relative concentration of LPS-activated macrophages cultured in the presence of XCAR-1 at different concentrations (µM) for 22 h of culture. No statistically significant differences were observed.*

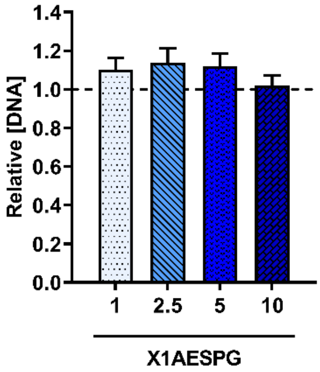

Figure S18. *DNA relative concentration of LPS-activated macrophages cultured in the presence of X1AESPG at different concentrations (µM) for 22 h of culture. No statistically significant differences were observed.*

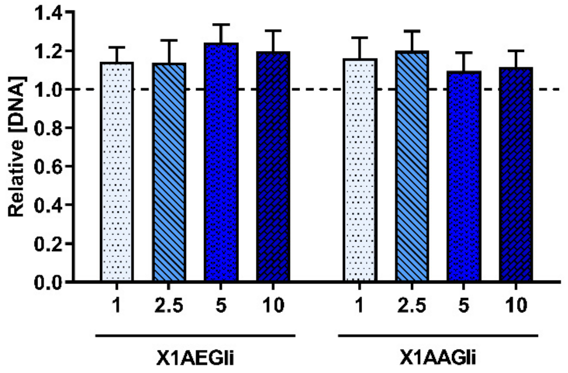

__Figure S19.__ DNA relative concentration of LPS-activated macrophages cultured in the presence of X1AEGli and X1AAGli at different concentrations (μM) for 22 h of culture. No statistically significant differences were observed.

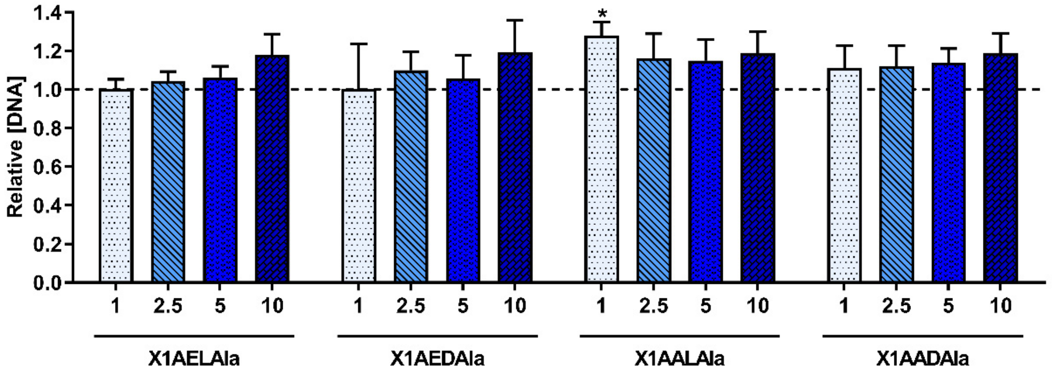

*__Figure S20.__ DNA relative concentration of LPS-activated macrophages cultured in the presence of X1AELAla, X1AEDAla, X1AALAla, and X1AADAla at different concentrations (μM) for 22 h of culture. Statistically significant differences are * ($p < 0.0140$) in comparison with non-treated LPS-stimulated macrophages.*

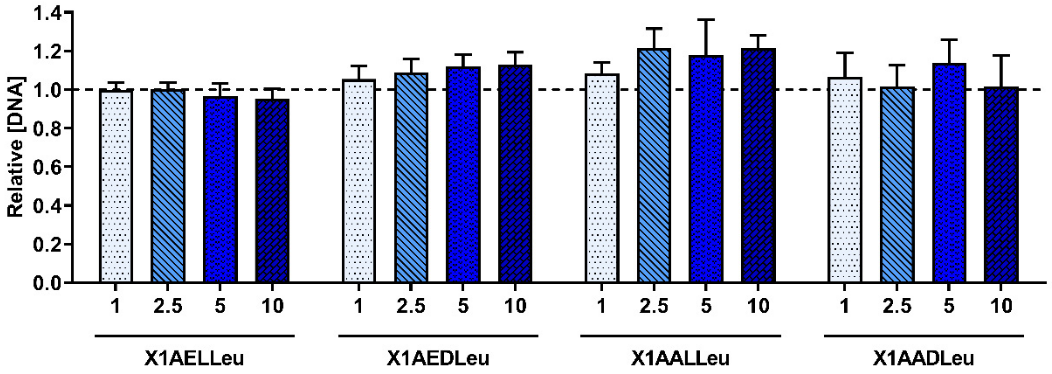

__Figure S21.__ DNA relative concentration of LPS-activated macrophages cultured in the presence of X1AELLeu, X1AEDLeu, X1AALLeu, and X1AADLeu at different concentrations (μM) for 22 h of culture. No statistically significant differences were observed.

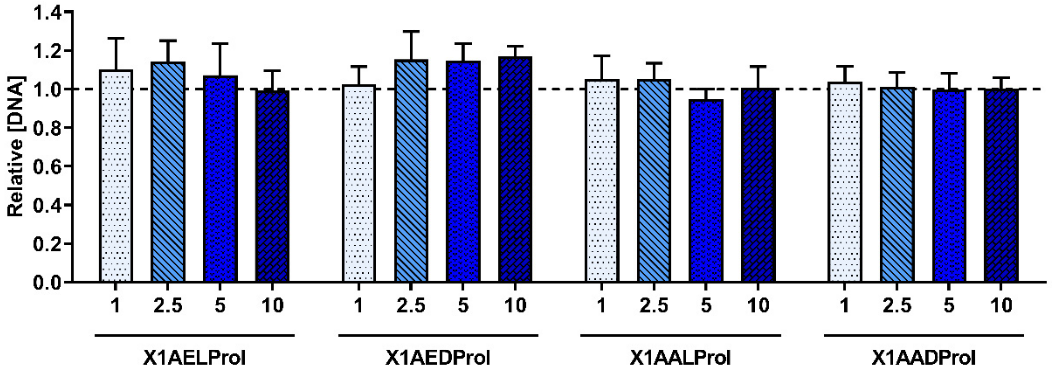

Figure S22. *DNA relative concentration of LPS-activated macrophages cultured in the presence of X1AELProl, X1AEDProl, X1AALProl, and X1AADProl and at different concentrations (μM) for 22 h of culture. No statistically significant differences were observed.*

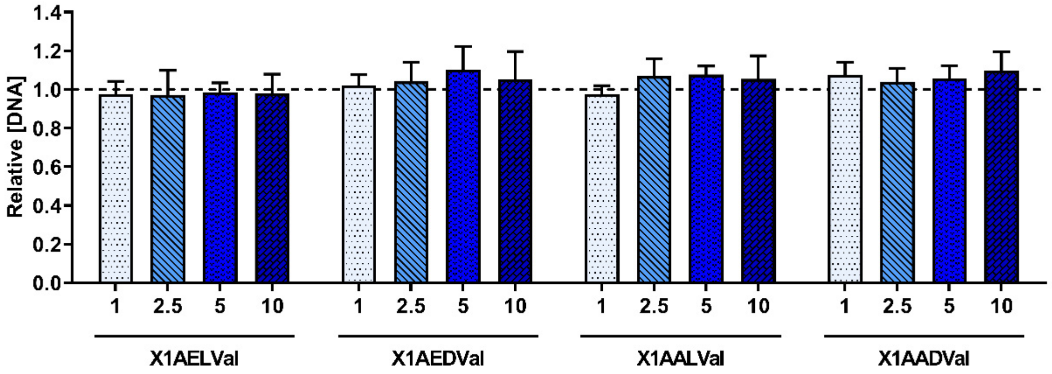

Figure S23. *DNA relative concentration of LPS-activated macrophages cultured in the presence of X1AELVal, X1AEDVal, X1AALVal, and X1AADVal at different concentrations (μM) for 22 h of culture. No statistically significant differences were observed.*

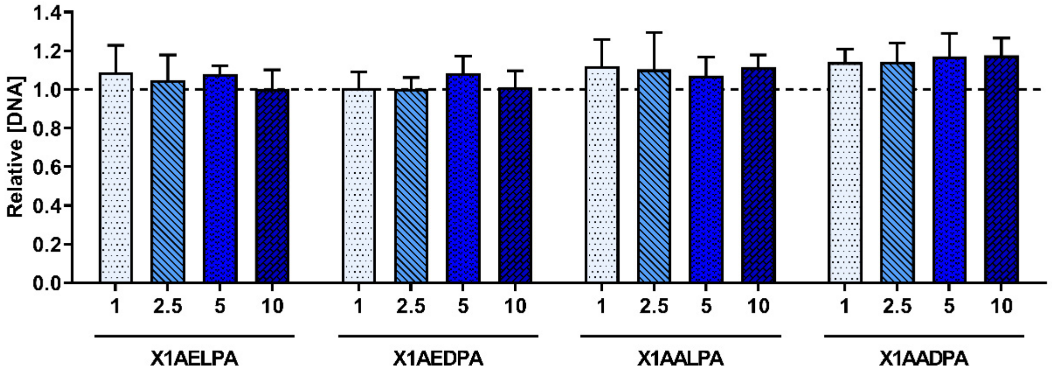

Figure S24. *DNA relative concentration of LPS-activated macrophages cultured in the presence of X1AELPA, X1AEDPa, X1AALPA, and X1AADPA at different concentrations (μM) for 22 h of culture. No statistically significant differences were observed.*

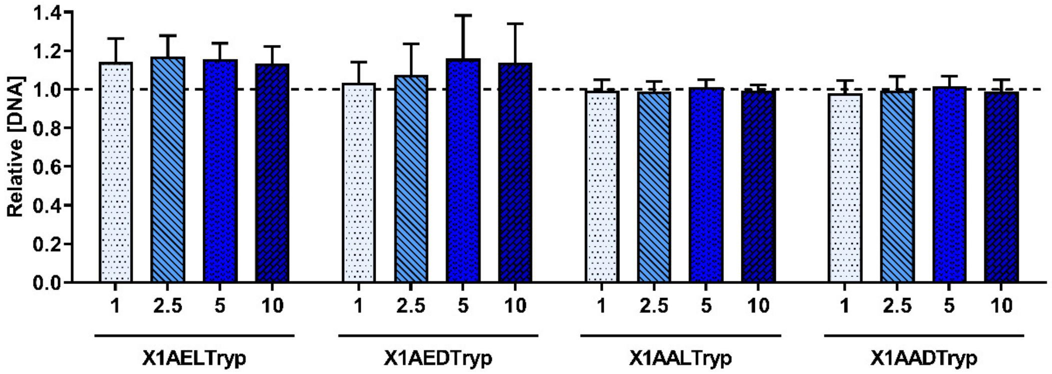

Figure S25. DNA relative concentration of LPS-activated macrophages cultured in the presence of X1AELTryp, X1AEDTryp, X1AALTrp, and X1AADTrp at different concentrations (μM) for 22 h of culture. No statistically significant differences were observed.

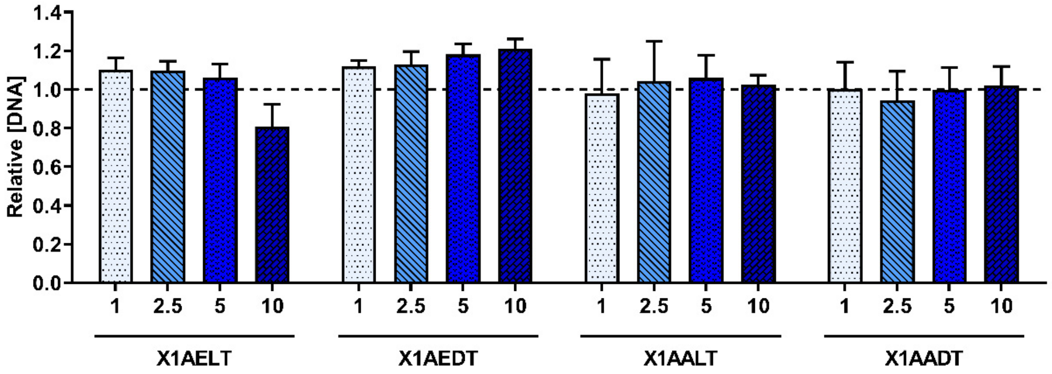

Figure S26. DNA relative concentration of LPS-activated macrophages cultured in the presence of X1AELT, X1AEDT, X1AALT, and X1AADT at different concentrations (μM) for 22 h of culture. No statistically significant differences were observed.

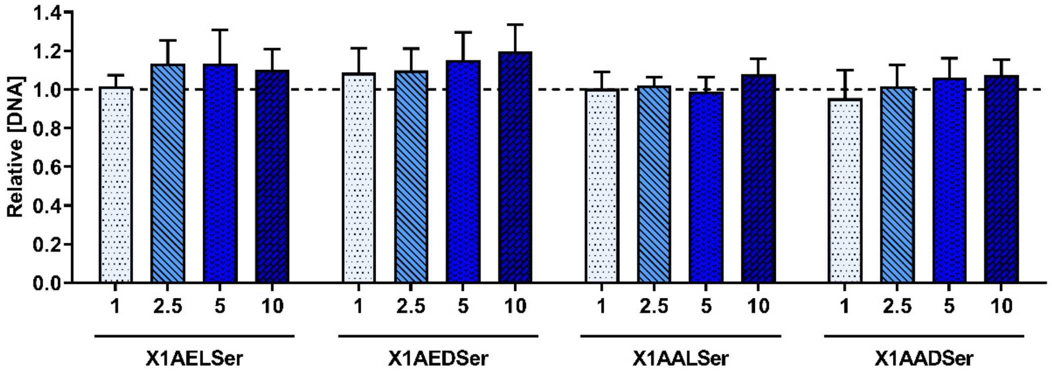

Figure S27. DNA relative concentration of LPS-activated macrophages cultured in the presence of X1AELSer, X1AEDSer, X1AALSer, and X1AADSer at different concentrations (μM) for 22 h of culture. No statistically significant differences were observed.

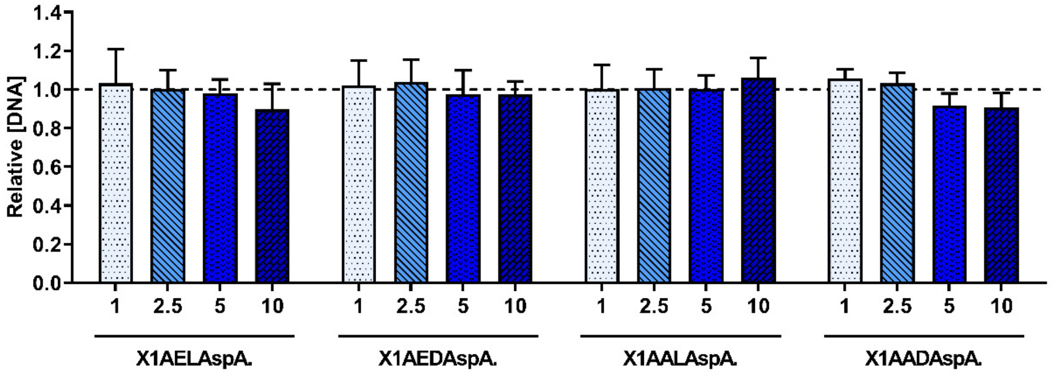

Figure S28. *DNA relative concentration of LPS-activated macrophages cultured in the presence of X1AELAspA., X1AEDAspA., X1AALAspA., and X1AADAspA. at different concentrations (μM) for 22 h of culture. No statistically significant differences were observed.*

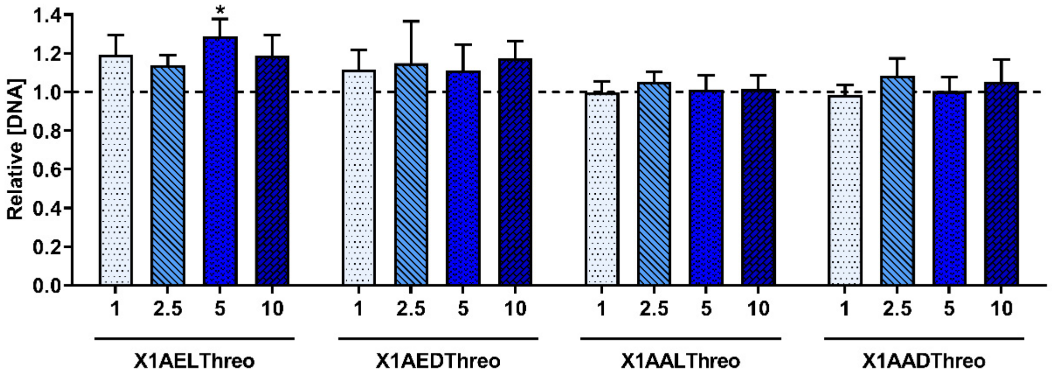

Figure S29. *DNA relative concentration of LPS-activated macrophages cultured in the presence of X1AELThreo, X1AEDThreo, X1AALThreo, and X1AADThreo at different concentrations (μM) for 22 h of culture. Statistically significant differences are * (p < 0.0322) in comparison with non-treated LPS-stimulated macrophages.*

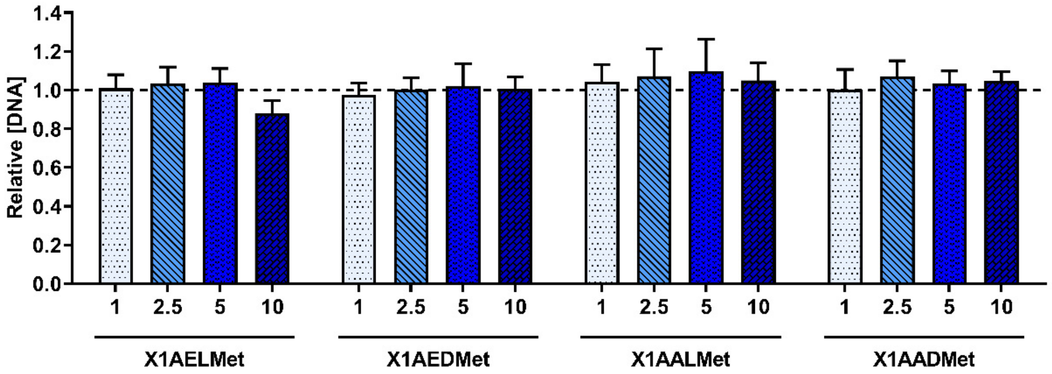

Figure S30. *DNA relative concentration of LPS-activated macrophages cultured in the presence of X1AELMet, X1AEDMet, X1AALMet, and X1AADMet at different concentrations (μM) for 22 h of culture. No statistically significant differences were observed.*

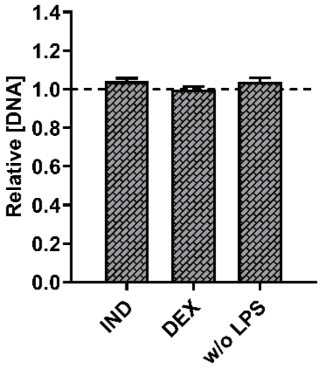

Figure S31. *DNA relative concentration of LPS-activated macrophages cultured in the presence of clinically used anti-inflammatory drugs (indomethacin – INDO – and dexamethasone – DEX –, at 10 μM), and non-stimulated macrophages (w/o LPS), for 22 h of culture. No statistically significant differences were observed.*

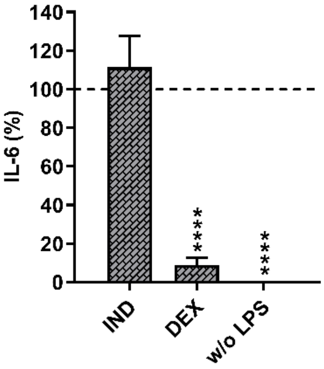

Figure S32. *IL-6 percentage of LPS-activated macrophages cultured in the presence of clinically used anti-inflammatory drugs (indomethacin – INDO – and dexamethasone – DEX, at 10 μM), and non-stimulated macrophages (w/o LPS), for 22 h of culture. Statistically significant differences are **** (p < 0.0001) in comparison with non-treated LPS-stimulated macrophages.*
